# Supplementary material for: Questioning approaches to consent in time critical obstetric trials: findings from a mixed-methods study
Source: BMJ Open. 2024 Feb 10;14(2):e081874. doi: 10.1136/bmjopen-2023-081874 (PMC10862288; doi:10.1136/bmjopen-2023-081874)
Supplement: Supplementary data [file bmjopen-2023-081874supp002.pdf]

COPE
The Carboprost or Oxytocin Postpartum haemorrhage Effectiveness Study

NHS

<Trust address 1>
<Trust address 2>
<Trust address 3>
<postcode>

<telephone number>

COPE: Carboprost vs Oxytocin as the First Line Treatment of Primary Postpartum Haemorrhage; A phase IV, double-blind, double-dummy, randomised controlled trial.

Emergency Patient Information Sheet and Consent Form

Version 1.0: 12-07-2018

Contents

Contents	Page
1) Why am I being asked after I was given the medicine?	2
2) Why are we doing the study?	2
3) What happens if I agree for you to use my information?	2
4) Do I have to take part?	3
5) How was it decided which medicine I received?	3
6) Timeline of visits	3
7) What are the alternatives for treatment?	3
8) What are the benefits and risk of taking part?	4
9) Will my participation be kept confidential?	4
10) What if there is a problem?	4
11) What will happen to the results of the study?	4
12) What are my electronic hospitalisation records?	4
13) Who is running the study?	5

Contact

Principal Investigator: **<PI NAME>**
Research Midwife: **<RM NAME>**
Telephone: **<number>**

Or visit the website: www.copestudy.uk

Information about the study

Excessive bleeding after childbirth (also known as postpartum haemorrhage or PPH) is a common problem which affects 1 in 20 women.

COPE is a research study to compare two drugs currently used to treat PPH, to decide which is better. The two drugs are **carboprost** and **oxytocin**.

Before you decide if you want to give your permission for your information to be used in this study, it is important for you to understand why the study is being done and what is involved.

Please take time to read the following information carefully and discuss it with friends or relatives if you wish. Taking part is voluntary. If you don't want to take part then you don't need to give a reason.

Your decision will not affect how the doctors and nurses will look after you.

Please ask a member of your clinical team if there is anything that is not clear, or if you would like more information.

Important things that you need to know

PPH is a medical emergency and needs to be treated without delay; it is important to prevent excessive bleeding as quickly as possible.

After you had given birth, you experienced a PPH. As this is a medical emergency, the priority is to treat the PPH and there is not time to properly discuss whether you would like to take part in COPE before giving treatment for PPH.

The ethical committee and medicines regulator have approved that women can be treated as part of the COPE research study and then approached after the medical emergency has passed to see if you are happy for us to collect some information for the research study.

Deja E, *et al. BMJ Open* 2024; 14:e081874. doi: 10.1136/bmjopen-2023-081874

Thank you for taking the time to read this information sheet. We hope you will find this information helpful.

Why am I being asked after I was given the medicine rather than before?

After you had given birth, you experienced a PPH.

PPH is a medical emergency and needed to be treated without delay; it was important to prevent excessive bleeding as quickly as possible. As there wasn't time to properly discuss the COPE research study we have approval from the ethical committee and medicines regulator to treat women as part of the COPE research study and then to ask for consent afterwards. This is research without prior consent (sometimes called deferred consent).

As part of the COPE study, when you experienced a PPH you were given two injections – one into the muscle and one into the vein. You were given either carboprost and placebo or oxytocin and placebo. A placebo is a salt solution containing no active drug – acting as a "dummy drug". Carboprost is injected into the muscle and oxytocin injected into the vein, therefore including a placebo as well as carboprost or oxytocin, in this way, means that neither you nor your doctor know what treatment you were given. This ensures a fair test.
We have therefore come to talk to you about the study as soon as possible after the medical emergency to ask for your permission to use information we have collected about your hospital stay.

Why are we doing the COPE study?

It is important to stop excessive bleeding after birth (PPH) as quickly as possible, as the blood loss can leave women feeling extremely weak and tired. They can then find it hard to care for their newborn baby. This hospital is one of about 40 hospitals in the UK involved in the COPE Study. About 4000 women will take part. We want to know if it is better to use carboprost or oxytocin as the first drug for treatment if a woman has a PPH.

The results from COPE will help doctors know which treatment is better for women with PPH.

What happens if I agree that you can use my information?

A member of the clinical team will talk to you in detail and you will be able to ask any questions that you have. If you have had all of your questions answered and are happy for your information to be collected then you will be asked to sign a consent form to confirm this. You will be given a copy of the consent form and the information sheet to keep.

We will collect some information about your health and we will ask you some questions about your experience in the study, your experience of childbirth and your general well-being. These questions will be asked before you leave the hospital and then again at four weeks after the birth. We will contact you via telephone, email or text to follow-up.

<u>Embedded study: Your views on how we ask people to take part in COPE are important</u>

You and your birth partner (if applicable) may be asked to take part in a telephone interview or face to face interview if you live in the North West and/or complete a questionnaire. Interview discussions will last about 60 minutes and will include how you felt about the COPE study. This study will inform how we ask other parents/ relatives about COPE and future related research. Again, it is up to you whether you take part or not, and your care will not be affected if you do not take part.

Do I have to take part?

No. Taking part is voluntary. If you choose to take part you can also choose to stop at any time without giving a reason. The standard of care you receive now and in the future will be the same whether you take part or not.

Deja E, *et al. BMJ Open* 2024; 14:e081874. doi: 10.1136/bmjopen-2023-081874

If at any point you decide to stop taking part in the study, you will receive the care usually offered by your hospital following PPH.

We will use any study information collected up until the time you stop taking part; this information will be pseudo-anonymised, meaning that identifying details, (e. g. your name) will only be accessed by people working on the study.

How was it decided which medicine I received?

In research studies we often split people up into groups to look at how different treatments work. People in one group get a different treatment than patients in another group. In the COPE study there are two treatment groups:

Group A: Women will receive oxytocin and placebo

Group B: Women will receive carboprost and placebo

It is really important that each group in the COPE study has a similar mix of patients in it so we know that if one group of patients does better than the other it is because of the treatment and not because there are differences in the types of women in each group.

We use a computer programme that puts women 'at random' into one of the groups – you might hear this described as 'randomisation' or 'random allocation', but they all mean the same thing. So, neither you nor your doctor chose your treatment. In the COPE study, you are equally as likely to be in the group receiving oxytocin as you are in the group receiving carboprost.

COPE is a 'blinded study' which means neither you nor your doctor will know what your treatment is. In the event of an emergency, the name of your treatment can be found out quickly.

Timeline of visits

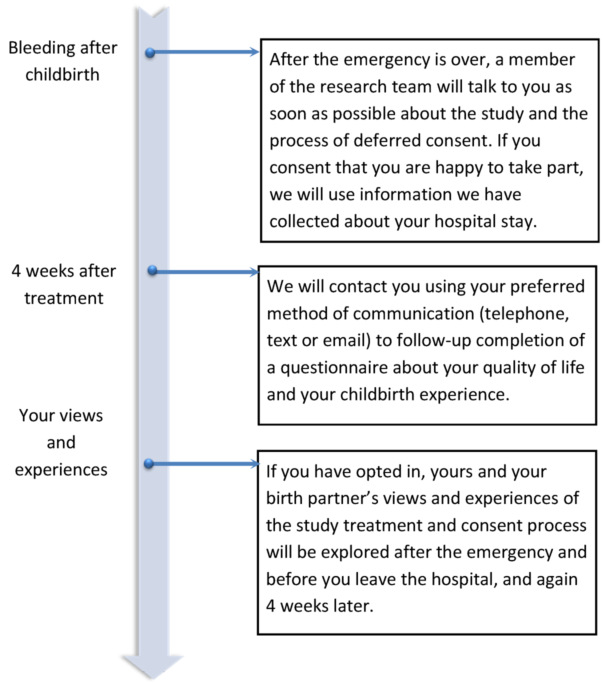

What are the alternatives for treatment?

If you were not treated as part of COPE, you would have usually been given oxytocin first and then carboprost if the bleeding continued. Both are common treatments for PPH, but we do not know which is better.

What are the benefits and risks of taking part?

The results from the study will help doctors know what treatment is best to use first for PPH in the future.

The common side effects of both treatments are as follows:

**Oxytocin**	**Carboprost**
Headache	Diarrhoea
Nausea (feeling sick)	Vomiting
Vomiting	High temperature
Low blood pressure	Increased blood pressure
Water Retention	

Deja E, *et al. BMJ Open* 2024; 14:e081874. doi: 10.1136/bmjopen-2023-081874

COPE The Carboprost or Oxytocin Postpartum haemorrhage Effectiveness Study

NHS

Will my participation be kept confidential?

Yes. We will follow strict ethical and legal practice so that all information collected about you during the study is kept strictly confidential. With your permission, we will send a letter to your GP to let them know you are taking part and use your NHS number to access anonymised data about your hospital admissions.

Any information with your name on it (such as the consent form) that has to be transferred to the coordinating centre in Liverpool, will be sent securely and only accessed by people working on the study or working to ensure the study is being run correctly.

When you agree to take part in a research study, the information about your health and care may be provided to researchers running other research studies in this organisation and in other organisations. These organisations may be universities, NHS organisations or companies involved in health and care research in this country or abroad. Your information will only be used by organisations and researchers to conduct research in accordance with the UK Policy Framework for Health and Social Care Research.

This information will not identify you and will not be combined with other information in a way that could identify you. The information will only be used for the purpose of health and care research, and cannot be used to contact you or to affect your care. It will not be used to make decisions about future services available to you, such as insurance.

What if there is a problem?

If you have any concerns about this study, you should ask to speak with one of your research team who will do their best to answer your questions.

If you remain unhappy and wish to complain formally, you can do this through the NHS Complaints Procedure. Details can be obtained from your hospital.

Every care will be taken in the course of this research study. However, in the unlikely event that you are injured as a result of the managing organisation (University of Liverpool), compensation may be available and you may have to pay your related legal costs. Your hospital where you receive your treatment has a duty of care to you whether or not you agree to participate in the trial and the University of Liverpool accepts no liability for negligence on the part of your hospital's employees. If you are harmed and this is due to someone's negligence, then you may have grounds for a legal action for compensation against the NHS Trust where you are being treated, but you may have to pay for your legal costs.

What will happen to the results of the study?

Our results will guide clinical practice for PPH. We plan to present the results of the study at conferences and publish them in medical journals so that we can explain to the medical community what our research results have shown. Confidentiality will be ensured at all times and you will not be identified in any publication.

What are my electronic hospitalisation records?

The NHS organisations routinely collect information about your inpatient, outpatient and any Accident & Emergency hospital care. In England and Wales the data are referred to as 'Hospital Episode Statistics' or HES data, whilst in Scotland the data are referred to as 'Scottish Morbidity Records' or SMR data; these are regarded as a special category of information.

The study team will retrieve information from your electronic hospitalisation records held by NHS Digital (https://digital.nhs.uk) for patients in England; NHS Wales Informatics Service Information Services (http://www.infoandstats.wales.nhs.uk/) for patients in Wales; and the electronic Data Research and Innovation Service (http://www.isdscotland.org/Products-and-Services/EDRIS/) for patients in Scotland.

Researchers at Bangor University, who are part of the COPE study, want to use your HES and SMR data to

calculate the overall costs of care. These data will be collected for the period from three months prior to the start of the study and for the duration of the study. To retrieve your HES records, information to identify you including your name, date of birth, NHS (or CHI) Number and COPE Study Number, will be securely transferred by the COPE research team to each of the organisations listed above. Towards the end of the study, they will in turn, securely transfer your pseudo-anonymised HES or SMR records to Bangor University using an encrypted electronic transfer system. The data is referred to as 'pseudo-anonymised' data because whilst all your identifying details (name, date of birth, postcode, NHS (or CHI) Number) will have been removed, there is still a unique COPE Study Number present in the data should COPE researchers need to refer back to your identifying details and contact you for any reason.

Your electronic HES and SMR records will be stored on secure Bangor University computer servers which meet NHS data security standards. Researchers at Bangor University will not be able to access your identifying details and will not keep the pseudo-anonymised HES or SMR data longer than 12 months. It will then be returned to the University of Liverpool for long term storage.

Who is running the study?

The University of Liverpool is the sponsor for this study based in the United Kingdom. The University is responsible for managing this study whilst the University's Clinical Trials Research Centre (CTRC) runs it on a day-to-day basis. This study is funded by the National Institute for Health Research (NIHR)'s Health Technology Assessment (HTA) programme.

The study has been reviewed by the funder and has been approved by the Health Research Authority, National Research Ethics Service Committee (<MAIN REC>) and the Medicines and Healthcare products Regulatory Agency.

The University of Liverpool will be using information from you and your medical records in order to undertake this study and will act as the data controller for this study. This means that we are responsible for looking after your information and using it properly. The University of Liverpool will keep identifiable information about you for 25 years after the study has finished.

Your rights to access, change or move your information are limited, as we need to manage your information in specific ways in order for the research to be reliable and accurate. If you withdraw from the study, we will keep the information about you that we have already obtained. To safeguard your rights, we will use the minimum personally-identifiable information possible.

You can find out more about how we use your information at www.copestudy.uk

Your NHS hospital will collect information from you and your medical records for this research study in accordance with our instructions.

Your NHS hospital will use your name, NHS number and contact details to contact you about the research study, and make sure that relevant information about the study is recorded for your care, and to oversee the quality of the study. Individuals from the University of Liverpool and regulatory organisations may look at your medical and research records to check the accuracy of the research study. Your NHS hospital will pass these details to the University of Liverpool along with the information collected from you and your medical records. The only people in the University of Liverpool who will have access to information that identifies you will be people who need to contact you to in relation to the study or audit the data collection process. The people who analyse the information will not be able to identify you and will not be able to find out your name, NHS number or contact details.

Your NHS hospital will keep identifiable information about you from this study for 25 years after the study has finished.

Thank you for reading this information sheet.

Contacts for further information

If you would like more information or have any questions about the COPE study please talk to:

Principal Investigator: **<PI NAME>**
Research Midwife: **<RM NAME>**

Telephone: **<number>**
Or visit the website: www.copestudy.uk

If you wish to discuss the study with someone independent of the research team you can contact the local NHS Patient Advice and Liaison Service (PALS) on: <telephone number>

COPE
The Carboprost or Oxytocin Postpartum haemorrhage Effectiveness Study

NHS

Consent Form

To be completed by the Researcher:

Site Name:	
Participant Study Number	
Participant Initials:	Participant DOB: / /
NHS / CHI Number	
Postcode	

To be completed by the patient:

	Tick	Initial
**Once you have read and understood each statement please tick (✓) and initial.**<br>Example: I confirm that I have read and understand the Participant Information Sheet.	✓	JS
1. I have read and understood the information sheet for this study. I have had the opportunity to ask questions and have these answered satisfactorily.		
2. I understand that I have already received the study treatment for PPH.		
3. I understand that participation is voluntary and that I am free to withdraw from the study at any time, without giving a reason, and without my care or legal rights being affected.		
4. I understand that my data will be retained for a maximum of 25 years by the site and by the Clinical Trials Research Centre (CTRC), part of the University of Liverpool, and that they will be stored in a confidential manner.		
5. I understand that relevant written and electronically-recorded sections of my medical notes, records and any data collected during the study may be looked at by authorised individuals from the research team and those listed under 'Who is running the study?' section above, NHS Trust and Regulatory Authorities where it is relevant to my taking part in research. I give permission for these individuals to have access to these notes, my records and data.		
6. I understand that the data collected for this study may be used in a pseudo-anonymised form for related research.		
7. I give permission for a copy of this consent form which will include my name, date of birth and NHS number to be sent to the CTRC (where it will be kept in a secure location), to allow confirmation that my consent was given.		
8. I agree to my GP being informed of my participation in the study.		
9. I agree for my name, date of birth, NHS (or CHI) Number, COPE Study Number and a copy of this consent form to be shared with NHS Digital or their UK devolved equivalent so they can link this information to Hospital Episode Statistics (HES), or Scottish Morbidity Records (SMR) containing my details and provide members of the COPE study team working on the trial with HES and SMR data regarding my hospital attendances.		
10. I agree to take part in the above study.<br>My preferred method of communication for follow – up is (please tick as appropriate):<br>**Telephone** ☐ **Email** ☐ **Text** ☐		

Consent Form

To be completed by the Researcher:

Site Name:	
Participant Study Number	
Participant Initials:	Participant DOB: / /
NHS / CHI Number	
Postcode	

**Contact details:**	
**Telephone number:**	
**Email address:**	

Below are embedded study statements:

11. I agree to being contacted to take part in an embedded study interview.
12. I agree to completing an embedded study questionnaire.
13. I agree for the audio recording of my consent discussion with the COPE midwife/doctor to be used in the embedded study.

Below are optional study statements:

14. I agree that I may be contacted in the future in relation to this or other relates studies.

Your **full** name (please print):			
Your signature:		Date:	dd / mm / yyyy

To be completed by the Researcher:			
Researcher **full** name (please print):			
Researcher signature:		Date:	dd / mm / yyyy

To be completed by the Translator (if used):			
Translator **full** name (please print):			
Translator signature:		Date:	

Deja E, *et al. BMJ Open* 2024; 14:e081874. doi: 10.1136/bmjopen-2023-081874
