# Supplementary material for: Questioning approaches to consent in time critical obstetric trials: findings from a mixed-methods study
Source: BMJ Open. 2024 Feb 10;14(2):e081874. doi: 10.1136/bmjopen-2023-081874 (PMC10862288; doi:10.1136/bmjopen-2023-081874)
Supplement: Supplementary data [file bmjopen-2023-081874supp003.pdf]

Trial number: ☐☐ ☐☐

COPE Study: postnatal questionnaire

Carboprost vs Oxytocin as the First Line Treatment of Primary Postpartum Haemorrhage.

The following questions are about the COPE study discussion and consent process that you/your partner took part in. We refer to people agreeing to take part in research as ‘consenting’. We are interested in when you/ your partner were approached to discuss COPE and what you thought of the information you were given.

a. Date of birth: ☐☐/☐☐/☐☐☐☐

b. Today’s date: ☐☐/☐☐/☐☐☐☐

c. First part of your postcode: ___ ___ ___ ___

d. Are you:

Male ☐ or female ☐

Patient (Mum) ☐ or Birth partner ☐ Please describe your relationship to the patient in the box below (e.g. husband, wife, partner, mother, friend)

☐

e. Have you/your partner had a postpartum haemorrhage in a previous pregnancy

Yes ☐ No ☐ I don’t know ☐ Not applicable ☐

f. When did a doctor or midwife first speak to you about COPE? (Please tick all that apply)

1) During an antenatal appointment ☐
2) When I was bleeding before treatment was given ☐
3) After I had have been given treatment for bleeding ☐
4) I can’t remember ☐

COPE Woman and birth partner postnatal questionnaire version 1.0 19/01/2018

1. *Please indicate how strongly you agree or disagree with the following statements by placing a circle around the answer that best fits your opinion*

**Statements**	**Agree**	**Neither agree nor disagree**	**Disagree**
a. The doctor or midwife checked that it was a convenient time to discuss research before discussing COPE	1	2	3
b. The information I received about COPE was clear and straightforward to understand	1	2	3
c. I had enough opportunity to ask questions about COPE	1	2	3
d. I was satisfied with the consent process for COPE	1	2	3
e. It was difficult to take in the information I was given about COPE	1	2	3
f. It was difficult to make a decision about COPE	1	2	3
g. I made this decision	1	2	3
h. It was a joint decision (e.g. between patient and birth partner)	1	2	3
i. Someone took this decision away from me	1	2	3
j. I was not in control of this decision	1	2	3
k. The decision about the research was inappropriately influenced by others	1	2	3

If the answer to this question is ‘Agree’, please state who you think influenced the decision about the research:

____

Deja E, *et al. BMJ Open* 2024; 14:e081874. doi: 10.1136/bmjopen-2023-081874

2. *Did you/your partner consent for your participation in COPE?*

◯ Yes (Go to Question 3) ◯ No (Go to Question 4)

3. *What were your reasons for providing consent for your participation in COPE?*

Please tick all that apply <u>and then circle your main reason</u> (e.g. ✔)

a. To help me/my partner ☐

b. To help other women in the future ☐

c. I felt that medical studies like COPE are important ☐

d. Because I trusted the doctor or midwife who explained COPE ☐

e. The treatment had already been given to me/my partner ☐

f. The treatment worked ☐

g. I didn't feel comfortable saying no to the doctor or midwife who explained the study ☐

h. Other (Please state): ☐

____

4. *If you did not provide consent, please provide your reasons for deciding that you/your partner would not take part in COPE*
(If you do not wish to do so, please leave this space blank)

5. *Please tell us any comments or suggestions you have to improve the recruitment and consent process for COPE:*

We would like to thank you for taking the time to complete this questionnaire.

Please place the questionnaire in the envelope provided, <u>seal it and give it back to the doctor or study midwife.</u>

Deja E, *et al. BMJ Open* 2024; 14:e081874. doi: 10.1136/bmjopen-2023-081874
