# Supplementary material for: Questioning approaches to consent in time critical obstetric trials: findings from a mixed-methods study
Source: BMJ Open. 2024 Feb 10;14(2):e081874. doi: 10.1136/bmjopen-2023-081874 (PMC10862288; doi:10.1136/bmjopen-2023-081874)
Supplement: Supplementary data [file bmjopen-2023-081874supp004.pdf]

Version 1.0 19.01.2018
IRAS ID: 235254

COPE: Interview Topic Guide- Patient

Introduce self, thank participant for taking part and confirm happy to take part in an interview. **Remind participants of broad aims: We are interviewing women and their partners who have been asked to participation in the COPE study to find out experiences of women, their birth partners and practitioners involved in recruitment and consent in COPE. Findings from interviews will be used to inform how we conduct the rest of the trial.**

Section 1: Introduction, setting ground rules

Discuss the following issues:

- Review the nature and purpose of the research.
- No right or wrong answers, aim to understand experiences of recruitment and consent.
- Confidentiality, use of data.
- Explain the use of data recorder, transcription, use of pseudonym (invite to choose), use of verbatim quotes, will be taking field notes.
- Researcher aware that discussion might bring up difficult memories, explain can decline to answer any question or prompt; can ask to stop at any time if feels need to.
- Expected duration of interview.
- Check consent form signed
- Ask if any questions.

Bereaved mothers: I have some idea about your circumstances, if there is anything that you find difficult to talk about please don't feel that you have to. If you want to stop the interview at any point, then please let me know.

I will start with some questions about you if that's ok and then I will ask you about your experience of being invited to take part in the COPE study?

Check recorder working

Introduce and switch on tape recorder

Section 2: demographics

To start we have a couple of general questions about you if that's ok?

1. Could you please tell me your age?
2. Before your maternity leave were you in employed or unemployed? If employed, what was your occupation?

Deja E, *et al. BMJ Open* 2024; 14:e081874. doi: 10.1136/bmjopen-2023-081874

3. What is the first part of post code?
4. What would you describe as your ethnic background?
5. How many children do you have?

Section 3: Antenatal awareness and COPE recruitment

1. **When did you first hear about the COPE study?**

2. **Were you provided with information about the COPE study during an antenatal visit?**

-IF NO

- **Did you see any leaflets or posters about the COPE Study during your pregnancy?** Explore location, views on the information; whether they requested further details, who from, what questions did you have, where they answered sufficiently.

Go to Section 4

IF YES

- Who discussed COPE with you (role)
- Could you tell me what they explained about the COPE study?
- Did they go through any of the potential risks or benefits of taking part in the study at that point? If yes, how did they describe these?
- Was there anything that you found: a) unclear b) surprising?
- Is there anything else that sticks out in your mind about the discussion?
- (*Prompt- if study aims not described by participant in response to the second point above*) This next question is not a test. Just so we can gage whether the trial is being explained clearly to women. Could you tell me what you understood the study was about?
- Do you have any suggestions about how this discussion could be improved in the future? If yes how?
- Could you tell me about any written information you were given about the COPE study? Explore whether they were given the information leaflet – short version and/or the full patient information sheet
- When did you receive this information? (Prompt: explore written and verbal and time point)
- Did you read the information leaflet/sheet? (Prompt: If they read the short information PIS leaflet or the full information PIS)
- What did you think about the information leaflet/sheet?
- Was there anything that you found: a) unclear? b) surprising?

Deja E, *et al. BMJ Open* 2024; 14:e081874. doi: 10.1136/bmjopen-2023-081874

- Could the information leaflet be improved in any way? (Prompt: If so, how?)

Section 4: birth and PPH experience (for bereaved mothers introduce this section and restate that if there is anything that you find difficult to talk about please don't feel that you have to).

1. Please could you tell me about your labour and birth? (Explore: mode of delivery- vaginal birth, caesarean birth, emergency, planned, water birth, general anaesthetic etc.)
2. How did you feel during labour and birth?
3. After birth were you aware that you were losing a lot of blood.
   - *Explore what alerted you to the problem?*
   - *What were you told, by whom about the loss of blood.*
   - *Could you tell me what happened?, how long after birth?*
   - *How did you feel, any worries or fears, how did you manage your emotions?*
4. Can you remember if you were given any treatments to stop the bleeding? *(If can recall explore awareness of what drug, if it did stop the bleeding, other treatments given)*
5. (if applicable) Did you have a PPH with other births?

Section 5. COPE first approach (birth)

1. **Could you tell me at what point did someone first mention the COPE study? For example, did a midwife or doctor briefly speak to you about COPE before a treatment was given to help stop your bleeding?**

IF YES (e.g. verbal consent at time of PPH)

- Could you tell me a bit more about that? *Explore who and when,* did they speak to you or (partner name) or was this a joint discussion?
- Could you tell me how were you feeling at that point in time?
- Can you remember how you responded? Explore if consented, declined, did you ask any questions?
- Is there anything that sticks out in your mind about the discussion?
- Do you think that this was the best time to discuss the study with you?
- If not, when would you have preferred to have the discussion about COPE? *Explore views on seeking consent after the trial drugs have been given.*
- *Did someone come and speak to you a second time about COPE after your birth?*

IF NO (e.g. consent within 24 hours or can't remember)

- How would you have felt if the doctor or midwife had spoken to you at that point in time? (*Explore acceptability of seeking verbal consent during PPH, would you have been able to make a decision about taking part in COPE at that point in time?)*
- When do you remember a midwife or doctor first speaking to you about the COPE study?

  (Explore who discussed COPE and whether person familiar to participant, whether it

matters to the participant who approached her, timing of approach, whether this was an appropriate time and if timing was checked).

Section 6. COPE full recruitment conversation

1. **Could you tell me what they explained to you about the COPE study? (explore level of involvement in trial discussions) If already consented antenatally explore level of discussion- this could have just been a checking consent so questions below may be too detailed).**
2. **Did they go through any of the potential risks or benefits of taking part in the study at that point?** *If yes, how did they describe these?*
3. **Was there anything that you found: a) unclear b) surprising?**
4. **Is there anything else that sticks out in your mind about the discussion?**
5. **This next question is not a test. Just so we can gage whether the trial is being explained clearly to women. Could you tell me what you understood the study was about?**
6. **Do you have any suggestions about how this discussion could be improved in the future?** *If yes how?*
7. **Did you discuss the decision to be part of COPE with anyone else?** *Explore who, did their views influence your decision? Shared decision making*

Section 7. Written information (as above, if antenatally see if information given again)

1. **Could you tell me about any written information you were given about the COPE study?** *Explore whether they were given the information leaflet – short version and/or the full patient information sheet*
2. **When did you receive this information?** *(Prompt: explore written and verbal and time point)*
3. **Did you read the information leaflet/sheet?** (*Prompt:* If they read the short information PIS leaflet or the full information PIS)
4. **What did you think about the information leaflet/sheet?**
5. **Was there anything that you found: a) unclear? b) surprising?**
6. **Could the information leaflet be improved in any way?** *(Prompt: If so, how?)*

Section 8. Research without prior consent

Explain: Some women were not provided with COPE study information and invited to consent during an antenatal visit as they were not anticipated to be at high risk of a PPH. This meant that when they had a PPH they were included in the study without having a full discussion about the trial and invited to provide consent. This is research without prior consent, also known as deferred consent. Legislation allows research without prior consent in emergency situations as there is no time to have a discussion about the research and that actually having that discussion might delay vital treatment.

1. **What do you think of the use of research without prior content in emergency situations?**
2. **What do you think of the use of RWPC in the COPE study?**

ONLY IF RWPC approach used

3. **How long did you get to think about whether you wanted your information to be used in COPE?** *Do you think this was long enough?*
4. **Did you have the opportunity to ask any other questions about the study?**
5. **Did you ask any?** *What questions did you ask?, if not, why not*
6. **How long do you think people should be given to think about consenting to the use of their information in the trial?**
7. **In making the decision about your participation in COPE, what sort of things went through your mind?**
8. **Was there anything you found particularly helpful in making up your mind?**
9. **Was there anything you found unhelpful?**
10. **How hard was this decision?**
11. **Was there anything specific that influenced your decision**?
12. **Would you mind telling me what were your reasons for (providing consent/not providing consent)?**
13. **How do you feel about participating in this research?**
    - *Explore experiences of the research process e.g. recruitment, completing questionnaires, contact with research team*
    - *Ask how they feel about participating in this interview*

    Is there anything else you might want to add?

At the close of the interview briefly summarise the main points to confirm interpretation with the participant. Ask if they wish to expand any responses or add anything else to the discussion.

- *Ensure participant has contact details for the local research team should they wish to discuss any aspect of the study.*
- *Complete reflexive diary/field notes.*

Deja E, *et al. BMJ Open* 2024; 14:e081874. doi: 10.1136/bmjopen-2023-081874
