# Supplementary material for: Questioning approaches to consent in time critical obstetric trials: findings from a mixed-methods study
Source: BMJ Open. 2024 Feb 10;14(2):e081874. doi: 10.1136/bmjopen-2023-081874 (PMC10862288; doi:10.1136/bmjopen-2023-081874)
Supplement: Author's manuscript [file bmjopen-2023-081874.draft_revisions.pdf]

BMJ Open

BMJ Open is committed to open peer review. As part of this commitment we make the peer review history of every article we publish publicly available.

When an article is published we post the peer reviewers' comments and the authors' responses online. We also post the versions of the paper that were used during peer review. These are the versions that the peer review comments apply to.

The versions of the paper that follow are the versions that were submitted during the peer review process. They are not the versions of record or the final published versions. They should not be cited or distributed as the published version of this manuscript.

BMJ Open is an open access journal and the full, final, typeset and author-corrected version of record of the manuscript is available on our site with no access controls, subscription charges or pay-per-view fees (http://bmjopen.bmj.com).

If you have any questions on BMJ Open's open peer review process please email info.bmjopen@bmj.com

BMJ Open

Questioning approaches to consent in time critical obstetric trials: findings from a mixed methods study

Journal:	*BMJ Open*
Manuscript ID	bmjopen-2023-081874
Article Type:	Original research
Date Submitted by the Author:	08-Nov-2023

Complete List of Authors:	Deja, Elizabeth; University of Liverpool, Department of Public Health, Policy and Systems<br>Weeks, Andrew; University of Liverpool, Department of Women's and Children's Health<br>Van Netten, Charlotte; University of Liverpool, Liverpool Clinical Trials Centre<br>Gamble, Carrol; University of Liverpool, Health Data Science<br>Meher, Shireen; Birmingham Women's Hospital<br>Gyte, Gillian; National Childbirth Trust,<br>Lavender, Tina; Liverpool School of Tropical Medicine Department of Clinical Sciences,<br>Woolfall, Kerry; University of Liverpool,

Keywords:	Postpartum Women < Postpartum Period, OBSTETRICS, QUALITATIVE RESEARCH, Clinical Trial

SCHOLARONE™
Manuscripts

I, the Submitting Author has the right to grant and does grant on behalf of all authors of the Work (as defined in the below author licence), an exclusive licence and/or a non-exclusive licence for contributions from authors who are: i) UK Crown employees; ii) where BMJ has agreed a CC-BY licence shall apply, and/or iii) in accordance with the terms applicable for US Federal Government officers or employees acting as part of their official duties; on a worldwide, perpetual, irrevocable, royalty-free basis to BMJ Publishing Group Ltd ("BMJ") its licensees and where the relevant Journal is co-owned by BMJ to the co-owners of the Journal, to publish the Work in this journal and any other BMJ products and to exploit all rights, as set out in our licence.

The Submitting Author accepts and understands that any supply made under these terms is made by BMJ to the Submitting Author unless you are acting as an employee on behalf of your employer or a postgraduate student of an affiliated institution which is paying any applicable article publishing charge ("APC") for Open Access articles. Where the Submitting Author wishes to make the Work available on an Open Access basis (and intends to pay the relevant APC), the terms of reuse of such Open Access shall be governed by a Creative Commons licence – details of these licences and which Creative Commons licence will apply to this Work are set out in our licence referred to above.

Other than as permitted in any relevant BMJ Author's Self Archiving Policies, I confirm this Work has not been accepted for publication elsewhere, is not being considered for publication elsewhere and does not duplicate material already published. I confirm all authors consent to publication of this Work and authorise the granting of this licence.

For peer review only

Questioning approaches to consent in time critical obstetric trials: findings from a mixed methods study

Deja, E., Weeks, AD. Van Netten, C., Gamble, C., Meher, S., Gyte, G., Lavender, T and Woolfall, K.

Author details:

Dr Elizabeth Deja, PhD, Research Associate, University of Liverpool, Department of Public Health, Policy and Systems

Weeks, AD. MD FRCOG, Professor of International Maternal Health, Department of Women's and Children's Health, University of Liverpool and Liverpool Women's NHS Foundation Trust, members of Liverpool Health Partners.

Van Netten, C., Trial Manager, Liverpool Clinical Trials Centre, University of Liverpool.

Gamble, Carrol., Professor of Medical Statistics, Health Data Science, University of Liverpool.

Meher, S., MD, MRCOG, Consultant in Maternal Fetal Medicine, Birmingham Women's Hospital, Birmingham, UK

Gyte, G., BSc, MPhil. NCT PPI Consultant. NCT, Brunel House, 11 The Promenade, Clifton Down, Bristol. BS8 3NG

Lavender, T, PhD, Professor of Maternal and Newborn Health, Department of International Public Health, Liverpool School of Tropical Medicine, Pembroke Place, Liverpool L3 5QA.

Dr Woolfall, K., PhD, Reader in Health Research Methodology, University of Liverpool, Department of Public Health, Policy and Systems

Twitter

Kerry Woolfall @kerry_woolfall; Andrew Weeks @adweeks; Elizabeth Deja @BethDeja; Tina Lavender @DameTina1; COPE trial account @COPETrial; Carrol Gamble @Carrol_Gamble; LCTC @livuni_LCTC; IPHS @livuniIPH

Acknowledgements Special thanks to all the families who took part in the research and to our patient partners for their contribution to the design, conduct and study findings

For peer review only

Author contributions

KW led the study, which was embedded within the COPE Trial led by AW. ED, CG, GG, LT and AW were involved in the design of the study; or the acquisition, analysis or interpretation of data. ED collected the data. ED analysed the data with oversight from KW. ED and KW drafted the article and AW, LT, and CG critically revised it for important intellectual content. All authors gave approval for the version to be published. KW and ED agree to act as guarantors and to be accountable for all aspects of the work in ensuring that questions related to the accuracy or integrity of any part of the work are appropriately investigated and resolved.

Funding: The COPE Trial, including this embedded study, was funded by The National Institute for Health Research (NIHR) Health Technology Assessment (HTA) (Project number 16/16/06) and coordinated by the University of Liverpool.

Disclaimer: The views and opinions expressed herein are those of the authors and do not necessarily reflect those of the HTA Programme, NIHR, NHS or the Department of Health. The funders had no role in the collection, analysis and interpretation of data or in the decision to submit this article for publication.

Competing interests: NIHR, HTA Programme grant payments were made to the institutions of all authors to support the conduct of this study. ED and CVN employment roles were funded by the NIHR HTA programme grant payments made to their institutions.

Patient and public involvement: Patients and/or the public were involved in the design, conduct, reporting, or dissemination plans of this research. Refer to the Methods section for further details.

Ethics approval: This study involves human participants and was approved by West Midlands - Coventry & Warwickshire Research Ethics Committee (18/WM/0227)

Provenance and peer review: Not commissioned; Grant externally peer-reviewed.

Data availability statement: No data are available. Although data relevant to the study are included in the article and are uploaded as online supplemental information, no raw data are available. The datasets generated and analysed for this study are not publicly available due to participants being from a small, specialised population, which creates ethical and privacy concerns about being too identifiable.

ORDID IDs

Elizabeth Deja https://orcid.org/0000-0002-3626-4927

Andrew Weeks https://orcid.org/0000-0002-1909-337X

Kerry Woolfall https://orcid.org/0000-0002-5726-5304

Gill Gyte https://orcid.org/0000-0003-4266-3585

Shireen Meher https://orcid.org/0000-0002-9866-7932

Carrol Gamble https://orcid.org/0000-0002-3021-1955

For peer review only

Conflicts of interest

ED None

KW None

SM None

CVN None

ADW Is the inventor of the PPH Butterfly a device to facilitate uterine compression for the first-line management of PPH. The patent is held by the University of Liverpool, but ADW would receive a share of any royalties if commercialised.

CG None

Key words

Postpartum haemorrhage, Consent, Trials, Labour, Mixed-methods

For peer review only

ABSTRACT

Objective: Trial legislation enables research to be conducted without prior consent (RWPC) in emergency situations, yet this approach has rarely been used in time-critical obstetric trials. This study explored views and experiences of antenatal recruitment and consent and RWPC in an emergency intrapartum randomised clinical trial.

Design: Embedded, Mixed methods study within a trial, involving questionnaires, recorded recruitment discussions, interviews and focus groups in the first year of trial recruitment (12/2020-01/2022).

Setting: COPE is a double-blind randomised clinical trial, comparing the effectiveness of Carboprost and Oxytocin as first-line treatment of postpartum haemorrhage.

Participants: Two hundred and eighty-six people (190 women/ 96 birth partners), linked to 198/380 (52.1%) COPE recruits participated in the embedded study. Of these, 272 completed a questionnaire (178 women/ 94 birth partners), 22 were interviewed (19 women/ 3 birth partners) and 12 consent discussions were recorded. Twenty-seven staff took part in three focus groups and 9 staff were interviewed.

Results: Participants recommended that information about the study should be more accessible antenatally for those who wish to be informed. Most women and staff did not think it would be appropriate to seek consent during pregnancy or early labour as it may cause 'unnecessary panic' and lead to research waste, as most women would not become eligible. There was support for the use of RWPC as COPE interventions are used in standard clinical practice and viewed as low risk. Women who were approached about the trial whilst having a postpartum haemorrhage also supported RWPC as they could not recall research discussions.

Conclusions: Findings support the use of RWPC for time-critical interventions, and raise questions about the appropriateness of other commonly used consent pathways, including antenatal consent and verbal assent.

For peer review only

Strengths and limitations of this study

Strengths

- A comprehensive mixed methods design including questionnaires and in depth interviews from key stakeholders including trial participants, birth partners and staff; this allowed for triangulation of data and depth of insight.
- Insight was gained from women and birth partners who experienced informed consent through antenatal recruitment, as well as verbal assent during PPH and RWPC through emergency recruitment.
- Data were collected until there was thematic saturation and to the point of information power, which is the point when data is deemed to address the study aims; sample specificity, such as experience relevant to the study aims, and sample diversity.

Limitations

- The consent rate for the COPE trial was high (n= 363/380, 96%) which limited opportunities to gain insight into the views and experiences of women who declined to take part.
- Interviews were limited to participants who could speak English.

The original protocol for the study, see supplementary material 1.

For peer review only

INTRODUCTION

The Royal College of Obstetricians and Gynaecologists (RCOG) provides guidance on obtaining consent in time-critical perinatal research and recommends that the approach should vary according to the individual underlying risk of being eligible for participation (1). However, there is no evidence to support this strategy, or the quoted levels of risk, which means the design of clinical trial consent processes may not be in line with the expectations or priorities of women and stakeholders.

Recruiting women to intrapartum research studies is complex (2, 3). Several strategies have been used to balance the need to gain truly informed consent with the desire not to unnecessarily distress or over-medicalise a natural process (4). Predominantly, informed consent is sought from women before trial enrolment, however, this may not always be possible in intrapartum research for example if a woman completely lacks capacity (e.g. is unconscious). Legislation makes provisions for an alternative decision maker to be involved in enrolment decisions. This is usually a relative (e.g. legal representative) or doctor who is independent of the research. However, involving an alternative decision-maker requires time for information exchange and discussion and thereby this is not possible where there is an urgent need to treat within a medical emergency. In such urgent care situations, women or their representative may have very little time or capacity to consider study information and make an informed decision about participating in research. Research has shown staff may have ethical and legal concerns about enrolling women into a trial without written informed consent (5). Some studies include an antenatal consent process to help offset such concerns. For uncommon conditions or events, this means approaching potentially large numbers of women who may never become eligible. This can be costly and may overburden women with information or cause unnecessary worry for women who may never experience the trial condition (6, 7).

Houghton et al (2018) conducted a qualitative study exploring women's views and experiences of consent in an obstetric emergency in The WOMAN Trial, which explored treatments for postpartum haemorrhage (PPH) (8). The WOMAN trial included options for informed consent and to randomise women without their prior consent if a relative or doctor was not present to consent on their behalf. Regardless of the consent approach, and whether or not they consented or declined, women struggled to recall recruitment conversations and were often unable to differentiate between clinical and research discussions. No single consent pathway was preferred. Findings may have been limited by poor recall due to a stressful birth experience and interviews being conducted more than a year after trial recruitment took place.

Trials legislation enables research to be conducted without prior consent (RWPC, also known as deferred consent) when there is an urgent need to treat a patient for the purposes of the trial (9). This involves administering trial interventions to eligible patients without delay and then, once the emergency situation has passed, researchers seek consent for ongoing involvement in the study (10, 11). Although research has shown support for RWPC in paediatric and neonatal settings (12-15) there has been limited use of this approach in time critical obstetric trials. One pilot study (ACROBAT) (7) explored women's views and experiences of RWPC within a postpartum cluster trial and showed support for the approach in a small sample of women

COPE is a double-blind, randomised controlled trial (RCT) comparing the effectiveness of carboprost and oxytocin as first line treatments of PPH. As part of an embedded study, our research aimed to explore women, birth partner and clinician views on the various approaches to recruitment and consent to inform the ongoing RCT and future time critical obstetric trials.

For peer review only - http://bmjopen.bmj.com/site/about/guidelines.xhtml

Methods

Setting

The COPE Trial was designed with two consent processes: researchers could seek prospective antenatal consent for women at increased risk of PPH, or women could be recruited without prior consent during a PPH (see Figure 1). The latter method is unusual in a labour setting and required assessment of feasibility and patient acceptability.

[Insert Figure 1. COPE RCT consent process]

Study Design

We conducted an embedded mixed-methods study in all sites open during the first year of COPE Trial recruitment (12/2020- 01/2022). We used previous research (16-20) to develop participant information sheets (Supplementary material 2), questionnaires (Supplementary Material 3) and topic guides (Supplementary Material 4).

We collected questionnaires and conducted interviews with women and birth partners approached for participation in COPE. The questionnaire followed the same format as those used in similar studies (9, 11, 13) consisting of demographic questions, Likert scale questions from the Decision Making Control Instrument (14) and free text responses, taking on average five minutes to complete. Interview topic guides included questions on birth experiences, COPE information, approaches to recruitment, willingness to participate and views on trial acceptability

For clinical and research staff we undertook focus groups and interviews and recorded trial consent discussions with patients and birth partners (if applicable). The Staff focus group and interview topic guide explored experiences of recruitment and consent during the first year of recruitment, acceptability of the COPE trial, site training, screening, administering the COPE intervention, documentation, medication and logistics of running the trial.

Patient and public involvement

Before finalizing the research protocol, the consent process went through extensive consultation with parents and public both in person and online with consumer groups. A trial consumer panel was formed, led by an experienced consumer representative and Trial Management Group member (GG), who provided critical input and represented the participants' views throughout the study.

Women and birth partner recruitment and conduct

All women and their birth partners approached to take part in the COPE trial during its first year were eligible to take part in the embedded study if they spoke English. This included those who declined trial participation. When the COPE trial was broached by site staff, women and birth partners were informed of the embedded study and written consent was sought to complete a questionnaire and/ or take part in a telephone/ online interview with the COPE researcher (ED) at a convenient time within approximately a month. Two pilot sites also sought consent to audio record consent discussions which took place on a hospital ward within 24 hours of randomisation.

For peer review only - http://bmjopen.bmj.com/site/about/guidelines.xhtml

Eleven English sites recruited to COPE during the first year. Staff at all sites provided women and birth partners with questionnaires to complete immediately following the consent discussion with pre-addressed envelopes to be returned to ED (PhD, female, Psychologist, Research Associate). ED contacted those who consented to interview in sequential order (by receipt of a consent form), stratifying by site, and role (women/ birth partner). Priority was given to those who had declined consent for the COPE trial and/ or agreed to audio recordings. Based on previous studies (21-23), we anticipated that 20-25 interviews would be needed to reach thematic saturation, where additional data does not lead to any new major themes identified during analysis. Researchers were also looking for high levels of 'information redundancy' (24-26) and information power, the point when data is deemed to address the study aims; sample specificity, such as experience relevant to the study aims, and sample diversity (24, 27).

Due to COVID-19 restrictions, all interviews took place over the telephone. Respondent validation was used to add unanticipated topics to the topic guide as interviewing and analysis progressed [16]. A £30 shopping voucher was given to participants after the interview to thank them for their time.

Staff recruitment and conduct

Initial findings from women and birth partner interviews and questionnaires were used to develop staff focus groups and interview topic guides (see supplementary material). Co-investigators at the first four open sites were contacted regarding the capacity to hold an online focus group using Microsoft Teams or Zoom. The research nurse and co- investigators disseminated invitations to all staff involved in the conduct of the trial, including Participant Information Sheets and consent forms to be completed before the focus group. Staff members that could not make the focus groups and/or were at other sites were invited to take part in an online interview.

Analysis

Digital audio recordings were transcribed verbatim by a professional transcription company (UK Transcription, Brighton) and anonymised. Qualitative analysis of interviews, focus groups, audio-recorded recruitment discussion data and open response questionnaire data were interpretive and iterative (28) utilising a reflexive thematic analysis approach (29, 30). NVivo 12 software (QSR International Pty Ltd., Melbourne, Australia) was used to assist in the organisation and coding of data. Data from the parent and staff questionnaires were cleaned and entered into SPSS Version 24.0 (IBM Corp., Armonk, NY, US). Descriptive statistics are presented with percentages. ED and KW (PhD, female, social scientist, Reader) analysed and synthesised the data, drawing on the constant comparative approach (31, 32).

For peer review only

Results

Participants

During the first year of COPE Trial recruitment, 382 Women were screened for trial participation. A total of 366 individuals (n=260 women and 106 birth partners) gave consent to at least one part of the embedded study. Of these, 191 women and 96 birth partners provided data, linked to 199/ 380 (52.1%) COPE recruits at 9/11 (75%) sites (see Figure 2). Within these, 16 consent discussions with 12 women (mean 9 minutes per woman, range 4.1-19.6) on the emergency pathway were recorded, 272 questionnaires (178 women/ 94 birth partners) and 22 telephone interviews (19 women/ 3 birth partners) (mean of 31.6 minutes, SD 9.3, range 17 -49) were competed.

The eleven sites opened at different times across the 12 months of data collection. Four sites were open during the first six months, with the others opening between months seven and twelve, two in the last month. To prevent over-representation, the initial two sites were closed to the embedded study after seven months as thematic saturation had been reached for both questionnaires and interviews. The other sites remained open to ensure information power was achieved.

The first four open sites took part in the staff component of the embedded study. A total of 36 staff self-identified as research midwives (n=10), midwives (n=4), anaesthetists (n=2), obstetricians (n=14), principal Investigators (n=2), ward managers (n=1) and research leads (n=3). Of these, 27 took part in three focus groups at two sites (mean 28 minutes, range 31-52) and 9 in interviews (mean 50 minutes, range 38-103).

[Instert Figure 2: Embeded study recruitment]

For peer review only

Antenatal recruitment - the importance of awareness

Women, birth partners and practitioners had mixed views on the use of antenatal recruitment to the COPE trial. All of the women and birth partners who were recruited antenatally (n=16) and/ or indicated in the questionnaire that they *"first heard about COPE during an antenatal appointment"* (questionnaire item) (n=8) stated they were *"satisfied with the consent process"* (questionnaire item, 24/24, 100%).

The majority of women who were recruited in the emergency pathway, as well as staff involved in recruitment, believed that antenatal COPE recruitment may: "*be a bit overwhelming and cause mams to panic unnecessarily*" (P05, Women, interview) for women who do not have a PPH and those that do:

> "No, definitely after. Because you don't need that extra worry on top. And isn't it only 5% of women that haemorrhage anyway? It's such a low percentage. Even if you're telling the high-risk women, they're already concerned. Yes, I would say afterwards."

P12, Women, Interview

> "We didn't want to worry the women about PPH before the event because it might not happen to them. I think there was that fear of causing alarm about what might happen before it actually happens. You want her to look forward to the birth of her baby and not be worried about it. So, I think we were a little bit concerned that it might cause a bit of upset in the women unnecessarily"

P01, Staff, Interview

Staff highlighted that alongside the potential emotional burden, antenatal recruitment was practically and conceptually difficult. A couple of the staff described how the definition of high risk for PPH varied between hospitals, with conflicting definitions considered to be confusing and a barrier for antenatal recruitment:

> "Some of our ladies that were classed as high-risk for a PPH weren't on your list as being high-risk for antenatal approaching. So, that caused a bit of confusion sometimes"

P01, Staff, Interview

Practical issues to antenatal recruitment also included the COVID-19 pandemic restrictions limiting the: "*number of face-to-face consultations… it decreases our chances of meeting people*" (P01, Focus Group 1), as well as being "*really time-consuming*" (P05, Staff, Interview) and wasteful as recruitment may not lead to randomisation as: "*actually they did not have postpartum haemorrhage"* (P02, Staff, Focus group 1). They were also concerned with the possibility, that for those who did have a PPH, there may not be a COPE trained member of staff available to administer the drugs. These challenges were reflected in trial recruitment data (see Figure 2) with 22 antenatal consents resulting in just two randomisations.

Some staff were concerned about the possibility of accidentally recruiting a woman through the RWPC route who had previously declined consent antenatally. Despite processes to mitigate this risk being in place within the protocol, this possibility made some staff reluctant to recruit antenatally:

> "Because you could accidentally randomise someone who maybe they will be a decline, but that person maybe hasn't quite realised that, several months down the line, or weeks or whatever, when they then come to the Labour Ward."

For peer review only

P06, Staff, interview

Both women and staff reflected that the main benefit of antenatal recruitment: "*is that it increases awareness for the women*" (P04, Staff, Interview). Women recruited through the emergency route felt that raising awareness of the COPE trial antenatally would be sufficient rather than consent being sought at that point in time. Both women and staff felt that this could be achieved through parent-facing advertising: "*whether it's just posters and things like that*" (P04, Staff, Focus group 2) or an "*information leaflet on Badger* [electronic notes]" (P09, Staff, Interview). As the information would provide: "*the opportunity to know about it..[..].. they could read up more into for themselves.*" (P17, Women, Interview) for those that wish to do so.

Assent discussion during PPH - situational incapacity

Some staff discussed broaching the COPE trial with women and/or birth partners during PPH when they felt it was appropriate, despite verbal assent not being specified within the COPE protocol. A total of 42/272 (15.4%) women and birth partners indicated in the questionnaire that 'a doctor or midwife first spoke to them about COPE while they were bleeding before treatment was given'. When describing their experience of labour and PPH, women's experiences varied greatly based on levels of blood loss, trauma, medical interventions, physical health, sedation and consciousness. All of these impacted their level of situational capacity and in turn their view on seeking verbal assent in COPE:

> "I think I lost two and a half pints of blood. I just remember being very cold, very uncomfortable and frankly a little bit scared. Then I pretty much woke up four hours later and she [the baby] was already here."

P07, Women, Interview

> "I was nervous beforehand, but the staff were great. They just put my mind at rest and, yes, it just felt like it went really quickly. Everything seemed to go well. Yes, it was a really calm and positive experience."

P15, Women, Interview

During interviews, the few women who recalled the COPE trial being briefly discussed during their PPH were largely indifferent to the research discussion. Due to the consequences of going through labour and having just given birth, they often stated they had not taken in any study information due to lack of capacity:

> "Yes, I was fine with it. [being told about COPE] it was more beneficial that they came round after rather than explaining it fully in the [Caesarean] section room because in the theatre I wasn't thinking straight, so I couldn't tell you, I couldn't relay anything that they said in there.."

P13, Women, interview

In contrast, one woman who stated she wanted to be 'in control of her body' clearly recalled asking questions about the research and felt reassured to have all the information about what was going on, including information about COPE:

> "For me personally, I ask questions because I like to feel a little bit in control of body, I like to know exactly what's happening. So, me asking questions and them being able to explain it

For peer review only

there and then, that helped me come to terms with it, that helped calm me down really because I like to know step by step what is exactly happening."

P05, Women, interview

Women who did not hear about the COPE trial during their PPH were asked their views on seeking verbal assent at that point in time. Most felt that discussing research would be inappropriate, particularly if there was a lot of blood lost or in the context of a traumatic birth. As the quotations below illustrate, one woman felt that she was dying during her PPH. Most stated they would have been emotionally and physically unable to understand or retain research information and make an informed decision:

> "There was one point in theatre when I thought, "I actually am going to die." But aside from that they were amazing. Everyone in the theatre was cheering me on, being like, "Push. I dare you. Push." It was a bit like being at a football match."

P20, Women, Interview

> "In my opinion, labour is one of the most unplannable events that you can have. You can be completely low risk and end up requiring litres of blood. I think nobody is going to be retain that information at the time if they are haemorrhaging or having somebody place metal forceps in their vagina."

P18, Women, Interview

Not all women were aware that they had a PPH and some felt that broaching the trial to seek verbal assent could negatively affect women's birth experiences and cause panic by drawing attention to the bleeding:

> "I was completely unaware that I was having a bleed, whereas I think if they had made me aware in theatre that I was actually having a bleed and asked for my consent, I think I would have started panicking. I think that would have completely changed that really nice, calm memory I have got of giving birth. I think it is a good thing that they didn't ask for my consent right there and then, and then put me onto it."

P15, Women, Interview

Seeking consent when the emergency situation has passed

The majority (351/373, 94%) of all COPE participants during the first year were recruited through the RWPC route. In our sample, 96.3% of women and birth partners indicated they were satisfied (n= 144/161) or indifferent (n=11/161) with 'the consent process for COPE' (Questionnaire Item). They described how it: "*made sense when I read the information why it was done that way*." (P21, women, interview) as they would not have been able to take in the information at the time and on the whole would prefer not to have it mentioned to them to before, or during labour. This support for RWPC appeared to be underpinned by their: "*trust in the people that are looking after you*" (P19, Women, Interview), that staff: "*wouldn't do it if it's going to put you at risk or anything, so it's fine*." (P01, Women Interview). RWPC in COPE was also viewed as acceptable because both treatment arms were: "*approved in practice in its own right*" (P18, Women, Interview).

Whilst all those interviewed were happy with the use of RWPC, interview and audio-recorded recruitment discussion data indicated that at the time of consent, commonly within 24 hours after

birth, they were: "*shattered*" (P10, women, recruitment discussion recording), "*I'm really tired. I've just come over all tired*" (P12, Women, recruitment discussion recording); "*My whole body feels really weak and lethargic*." (P05, Women, recruitment discussion recording) and: "*in quite a lot of pain still*" (P16, Women, Interview). Alongside their physical condition: "*there was just too much happening*" (P10, Women, Interview) with people: "*just popping in to make sure you're okay* (P02, staff, recruitment discussion recording), women and baby checks, clinical care and looking after the baby. Consequently, they commented on how they had asked fewer questions than they might have done in another context. This was reflected in the audio recordings, which were short in length (mean 9 minutes, range 4.1-19.6) and predominantly consisted of the staff talking.

Despite the personal discomfort experienced at the point that the COPE trial was broached, during interviews women accurately recalled the purpose of the COPE trial and stated they had made an informed decision about taking part, with multiple opportunities to ask staff questions if they had wished. Recruitment discussion data indicated those who did ask questions were focused on clarifying details of their PPH: "*How much blood loss compared to…*? (P05, Women, recruitment discussion recording) and whether or not they had only received a placebo. During interviews, women reflected that their interactions with staff were clear and well-handled:

> "She came back and we still hadn't had a chance to read it. So, she left and then she came back later that day. She was great. It was all very clear. And she was very conscientious and understanding of the situation, just to give us space and time."

P12, Women, Interview

After discussing all the possible types of consent with women and birth partners during interviews, many concluded that if there had been an option between antenatal and RWPC recruitment for COPE, their preference was for the RWPC/ emergency route where: *"there was no consent given and they just gave it to me*" (P15, Women, Interview) due to the reasons outlined above.

Of the 6/161 (3.7%) who stated they were "not satisfied" with the use of RWPC in the questionnaire, four were birth partners and two were mothers. They used the open response section of the questionnaire to explain that they would have preferred to give consent in advance because: "*Seeking consent after the fact is disconcerting*" (P60, Women, Matched, Questionnaire) and: "*consent takes away some of the good will we have towards the programme*" (P101, Birth Partner, Questionnaire). One birth partner felt that: "*it didn't seem an emergency and more of a 'just in case' treatment* (P60, Birth partner, Matched, questionnaire) therefore not meeting the criteria for RWPC. One mother was not happy to have received an unnecessary intervention (the placebo): "*Tell people what is going in their bodies, don't get me wrong it helped but do not like the dummy needle*" (P199, Women, Questionnaire).

Staff described how they initially had concerns about the use of RWPC as their: "*knee-jerk response was the ethics of how can you give drugs to somebody without their consent?"* (P01, Staff, Interview). It was also considered: "*a really daunting approach for us because we really didn't know how women would take it*" (P04, Staff, Interview). However, experience with this approach appeared to change opinions about the acceptability of RWPC in this setting: "*I think the more we got through the trial, I think, the better we all have felt about it*." (P03, Staff, Focus group 3). Staff found RWPC acceptable in COPE because: "*the fact that the women are okay with it, puts to bed any worries that I had about how it would be perceived."* (P09, Staff, Interview). As the trial progressed, staff also concluded that giving the PPH treatment without prior consent was closer to standard practice as they do not ask for consent when administering PPH drugs in emergency situations:

For peer review only

> "If you have got a situation as a PPH, you don't go through all the different drugs. You don't say to a woman, 'We are giving this drug and this does this and that does that.' You don't go into great detail. You just say you are giving some medication and debrief them afterwards because, in that emergency situation, you haven't got time" (P08, Staff, Interview)

As also highlighted by women, Staff described how RWPC was acceptable in the COPE trial because it involved approved medications and actively treating the PPH: "*it is not a placebo medication, they are getting an active medication as well*" (P01, Staff, Focus group 1). Therefore: "*we not exposing the patient to unnecessary harm because of the trial*" (P01, Staff, Focus group 3) and: "*In essence, they are going to get their usual care*" (P11, Staff, Focus group 1).

The main issue staff experienced with RWPC was timing. They needed to obtain consent for the 24-hour main COPE trial questionnaire as close to that time point as possible. This was only If women were not busy and had the capacity to do so.

Trial screening logs indicated that the 17 women who declined consent were due to 'not receiving treatment for PPH' (n=2); women 'not wishing to take part in research' (n=7), no translation services being available (n=2), too much paper work' (n=1) and 'being too tired' (n=1). Or women providing verbal agreement, however, leaving hospital before written consent being obtained and then not returning the consent forms (n=4);'

Discussion

Establishing the most appropriate consent pathway for intrapartum research with women who may be vulnerable, have fluctuating capacity and need emergency treatment for PPH is ethically and practically challenging (2-4, 6-8). This mixed methods study provides insight into the experiences of women, birth partners and clinician and their views on approaches to recruitment and consent in the COPE trial, including the use of RWPC, which is novel in obstetric trials.

Our findings suggest that seeking informed consent antenatally was considered acceptable to those women and birth partners who experienced this pathway in COPE. Despite the COPE trial being designed in accordance with RCOG guidance (1), staff involved in recruitment, and women randomised to the trial through the emergency consent pathway questioned the ethics of consenting women who may never become eligible for the trial. They were concerned about causing unnecessary anxiety and information burden for women by asking them to consider something going wrong with their pregnancy, particularly for those that do not go on to have a PPH. This finding was also reported by Alvarez et al (33), who raised concerns about how a desire to not cause distress may lead to selection bias, with staff choosing who to approach. Women and birth partners in our study prioritised information provision, such as leaflets or posters, about the trial in antenatal settings over an informed consent process. Providing such information to women at increased risk of PPH would provide the opportunity for consideration by those who wish to do so (6) and therefore, in line with the bioethical principle of autonomy, protecting their right to make their own decision about research participation (34, 35). Staff were also concerned about the potential for research waste, a concern validated when the first 22 research consent discussions in COPE led to just two randomisations. After the first year of the COPE trial, taking into consideration the findings of the embedded study and recruitment rates, the antenatal consent pathway route was removed from the COPE protocol.

Despite verbal assent not being specified as part of the recruitment process in the COPE protocol, we found some clinicians did seek verbal assent for participation in the trial when women were

having a PPH. The majority of women stated they were not in a position to have, or understand research discussions at that point in time. As reported by Houghton et al 2018 (8), recall of such recruitment discussions was poor. We found that due to situational incapacity, verbal assent at the time of PPH was only viewed as acceptable in a minority of cases when women are seeking out information and appeared to have capacity. However, assessing such capacity is extremely difficult (2, 3) and, as our data suggests, clinicians did not always correctly judge if a woman had the capacity to discuss research. Women in our study expressed a clear preference to not be approached about research at this time.

To our knowledge, this is the first study to explore experiences of RWPC in an obstetric RCT. RWPC was viewed by women and birth partners as being logical, acceptable and appropriate as COPE interventions were considered low-risk and time critical. This is in line with views of RWPC in paediatric emergency situations (13, 21, 23) and the ACROBAT Pilot trial (7). Staff had some initial apprehension about the use of RWPC. However, they were reassured by women's positive responses. Roper et al's (36) seven-step framework to enhance practitioner explanations and parental understandings of RWPC in paediatric emergency and critical care trials highlights the importance of explaining the condition before the consent discussion. This is particularly relevant to obstetric trials where women may have fluctuating, or no capacity, and therefore not be aware of what has happened during birth. Indeed, there were both women and birth partners in our study who were unaware that a PPH had occurred when COPE was broached by staff. In line with guidance on RWPC (37), we recommend that research staff should check the appropriate timing of the research discussion with the bedside staff and ensure the patient is aware of their clinical condition and history during childbirth before explaining how they were entered into a clinical trial. Research staff should also explain the reasons why consent could not have been sought prospectively.

Strengths and limitations of this study

The study was strengthened by its mixed methods design including data collection methods at various time points from all key stakeholders including birth partners. The study included all sites open within the first year which provided sample variance (75% of sites) and data from all sites that opened in the first 11 months of COPE retirement. Giving insight into views on consent processes from a large proportion of COPE participants and birth partners linked to half (52%) of the COPE recruits.

This study has several limitations. Firstly, we had restricted insight into the views and experiences of women who declined to take part in the COPE trial. However, 100% of women approached antenatally proved consent and 97.5% of women recruited via the emergency pathway provided consent, so there was limited opportunity to access the minority who declined. Screening logs indicated that reasons for declining were not related to the approach to recruitment and more about not wanting to take part in any research. An exception was one birth partner, whose questionnaire indicated they had declined due to not being satisfied with the RWPC process. Secondly, audio-recorded recruitment conversations were restricted to two sites. Although limited in quantity, the insight gained from the data we did obtain suggested they were often very brief conversations led by clinicians and corroborated the accounts given by both women and staff. These data further highlight the importance of assessing appropriate timing of RWPC discussion with women who have just had a traumatic childbirth. Thirdly, there were no interviews with antenatal consent pathway recruits because of delays in obtaining and/or processing consent due to staffing shortages during the initial phase of the COVID-19 pandemic. Questionnaire data linked to 14 COPE recruits 14/22 (63%) who experienced this pathway provided insight into their satisfaction with the

For peer review only - http://bmjopen.bmj.com/site/about/guidelines.xhtml

recruitment process they experienced, but this did not provide the depth of understating that would be gained through an interview.

Conclusion

Recruiting women to intrapartum research studies is practically and ethically challenging. Our findings support the use of RWPC for time-critical, low-risk interventions and it raises questions about the appropriateness of other commonly used consent pathways, including seeking informed consent antenatally and verbal assent during an obstetric emergency.

For peer review only

Figures and supplementary Materials

Figure 1. COPE RCT consent process

Figure 2: Embedded study recruitment

SM 1 COPE Protocol

SM 2 Example Information Sheet and Consent Form - Emergency Patient

SM 3 Example Embedded Study Questionnaire- Postnatal

SM 4 Example Embedded Study topic guide - patient interview

References

1. RCOG. Obtaining Valid Consent to Participate in Perinatal Research Where Consent is Time Critical. Royal College of Obstetrics and Gynaecologists2016.
2. Vernon G, Alfirevic Z, Weeks A. Issues of informed consent for intrapartum trials: a suggested consent pathway from the experience of the Release trial [ISRCTN13204258]. Trials. 2006;7(1):13.
3. Russell AM, Shepherd V, Woolfall K, Young B, Gillies K, Volkmer A, et al. Complex and alternate consent pathways in clinical trials: methodological and ethical challenges encountered by underserved groups and a call to action. Trials. 2023;24(1):151.
4. Patel D, Nasir S, Elati A, Vernon G, Weeks A. Historical trends in the timing of informed consent for research into intrapartum complications. BJOG: An International Journal of Obstetrics & Gynaecology. 2012;119(3):361-5.
5. Lawton J, Hallowell N, Snowdon C, Norman J, Carruthers K, Denison F. Written versus verbal consent: a qualitative study of stakeholder views of consent procedures used at the time of recruitment into a peripartum trial conducted in an emergency setting. BMC Medical Ethics. 2017;18(1):1-13.
6. Lawton J, Snowdon C, Morrow S, Norman JE, Denison FC, Hallowell N. Recruiting and consenting into a peripartum trial in an emergency setting: a qualitative study of the experiences and views of women and healthcare professionals. Trials. 2016;17:195.
7. Sweeney L, Lanz D, Daru J, Rasijeff AM, Khanom F, Thomas A, et al. Deferred consent in emergency obstetric research: findings from qualitative interviews with women and recruiters in the ACROBAT pilot trial for severe postpartum haemorrhage. BMJ open. 2022;12(5):e054787.
8. Houghton G, Kingdon C, Dower M, Shakur-Still H, Alfirevic Z. What women think about consent to research at the time of an obstetric emergency: a qualitative study of the views of a cohort of World Maternal Antifibrinolytic Trial participants. BJOG: An International Journal of Obstetrics & Gynaecology. 2018;125(13):1744-53.
9. The Medicines for Human Use (Clinical Trials) Regulations, (2004).
10. Woolfall K, Frith L, Gamble C, Gilbert R, Mok Q, The CONNECT advisory group, et al. How parents and practitioners experience research without prior consent (deferred consent) for emergency research involving children with life threatening conditions: a mixed method study. BMJ Open *2015*;5(9):e008522
11. Woolfall K, Frith L, Dawson A, Gamble C, Lyttle MD, The CONNECT Advisory Group, et al. 15 minute consultation: an evidence-based approach to research without prior consent (deferred consent) in neonatal and paediatric critical care trials. Archives of disease in childhood - Education & practice edition. 2015;101(1):49-53.
12. Woolfall K, Young B, Frith L, Appleton R, Iyer A, Messahel S, et al. Doing challenging research studies in a patient-centred way: a qualitative study to inform a randomised controlled trial in the paediatric emergency care setting. BMJ Open. 2014;4(5).

For peer review only

13. Deja E, Peters MJ, Khan I, Mouncey PR, Agbeko R, Fenn B, et al. Establishing and augmenting views on the acceptability of a paediatric critical care randomised controlled trial (the FEVER trial): a mixed methods study. BMJ Open. 2021;11(3):e041952.
14. O'Hara CB, Canter RR, Mouncey PR, Carter A, Jones N, Nadel S, et al. A qualitative feasibility study to inform a randomised controlled trial of fluid bolus therapy in septic shock. Archives of Disease in Childhood. 2017.
15. Woolfall K, Frith L, Dawson A, Gamble C, group TCa, Young B. Research without prior consent (deferred consent) in trials investigating the emergency treatmemt of critically ill children: CONNECT study guidance. Liverpool: University of Liverpol; 2015 July 2015.
16. Molyneux S, Njue M, Boga M, Akello L, Olupot-Olupot P, Engoru C, et al. 'The Words Will Pass with the Blowing Wind': Staff and Parent Views of the Deferred Consent Process, with Prior Assent, Used in an Emergency Fluids Trial in Two African Hospitals. PLoS ONE. 2013;8(2):e54894.
17. O'Hara CB, Canter RR, Mouncey PR, Carter A, Jones N, Nadel S, et al. A qualitative feasibility study to inform a randomised controlled trial of fluid bolus therapy in septic shock. Arch Dis Child. 2017:archdischild-2016-312515.
18. Woolfall K, Frith L, Gamble C, Gilbert R, Mok Q, Young B. How parents and practitioners experience research without prior consent (deferred consent) for emergency research involving children with life threatening conditions: a mixed method study. BMJ Open. 2015;5(9):e008522.
19. Peters MJ, Woolfall K, Khan I, Deja E, Mouncey PR, Wulff J, et al. Different temperature thresholds for antipyretic intervention in critically ill children with fever due to infection: the FEVER feasibility RCT. Health Technology Assessment 2019;23(5):1 - 148.
20. Roper L, Lyttle MD, Gamble C, Humphreys A, Messahel S, Lee ED, et al. Planning for success: overcoming challenges to recruitment and conduct of an open-label emergency department–led paediatric trial. Emergency Medicine Journal. 2021;38(3):191-7.
21. Woolfall K, Frith L, Gamble C, Gilbert R, Mok Q, Young B, et al. How parents and practitioners experience research without prior consent (deferred consent) for emergency research involving children with life threatening conditions: a mixed method study. BMJ Open. 2015;5.
22. O'Hara CB, Canter RR, Mouncey PR, Carter A, Jones N, Nadel S, et al. A qualitative feasibility study to inform a randomised controlled trial of fluid bolus therapy in septic shock. Arch Dis Child. 2018;103(1):28-32.
23. Woolfall K FL, Gamble C, Young B. How experience makes a difference: practitioners' views on the use of deferred consent in paediatric and neonatal emergency care trials. BMC Med Ethics. 2013;14(45).
24. Saunders B, Sim J, Kingstone T, Baker S, Waterfield J, Bartlam B, et al. Saturation in qualitative research: exploring its conceptualization and operationalization. Quality & Quantity. 2018;52(4):1893-907.
25. Baker SE, Edwards R. How many qualitative interviews is enough? National Centre for Research Methods (NCRM): . 2012.
26. Mays N, Pope C. Assessing quality in qualitative research. Bmj. 2000;320(7226):50-2.
27. Malterud K, Siersma V, Guassora A. Sample Size in Qualitative Interview Studies. Qualitative Health Research. Sage Journals. 2016;26(13):1753-60.
28. Braun V, Clarke V. What can "thematic analysis''offer health and wellbeing researchers? International journal of qualitative studies on health and well-being. 2014;9:26152.
29. Byrne D. A worked example of Braun and Clarke's approach to reflexive thematic analysis. Quality & Quantity. 2021:1-22.
30. Byrne D. A worked example of Braun and Clarke's approach to reflexive thematic analysis. Quality & Quantity. 2021.
31. Glaser BG. The constant comparative method of qualitative analysis. Social Problems. 1965;12(4):436-45.
32. Stiles WB. Evaluating qualitative research. Evidence-Based Mental Health. 1999;2(4):99-101.

33. Alvarez M, Hotton EJ, Harding S, Ives J, Crofts JF, Wade J. Women's and midwives' views on the optimum process for informed consent for research in a feasibility study involving an intrapartum intervention: a qualitative study. Pilot and Feasibility Studies. 2023;9(1):98.
34. Entwistle VA, Carter SM, Cribb A, McCaffery K. Supporting Patient Autonomy: The Importance of Clinician-patient Relationships. Journal of General Internal Medicine. 2010;25(7):741-5.
35. Kihlbom U. Autonomy and negatively informed consent. Journal of Medical Ethics. 2008;34(3):146-9.
36. Roper L, Lyttle MD, Gamble C, Humphreys A, Messahel S, Lee ED, et al. Seven-step framework to enhance practitioner explanations and parental understandings of research without prior consent in paediatric emergency and critical care trials. Emergency Medicine Journal. 2021;38(3):198-204.
37. Woolfall K, Frith L, Dawson A, Gamble C, Lyttle MD, Young B. Fifteen-minute consultation: an evidence-based approach to research without prior consent (deferred consent) in neonatal and paediatric critical care trials. Archives of disease in childhood Education and practice edition. 2016;101(1):49-53.

For peer review only

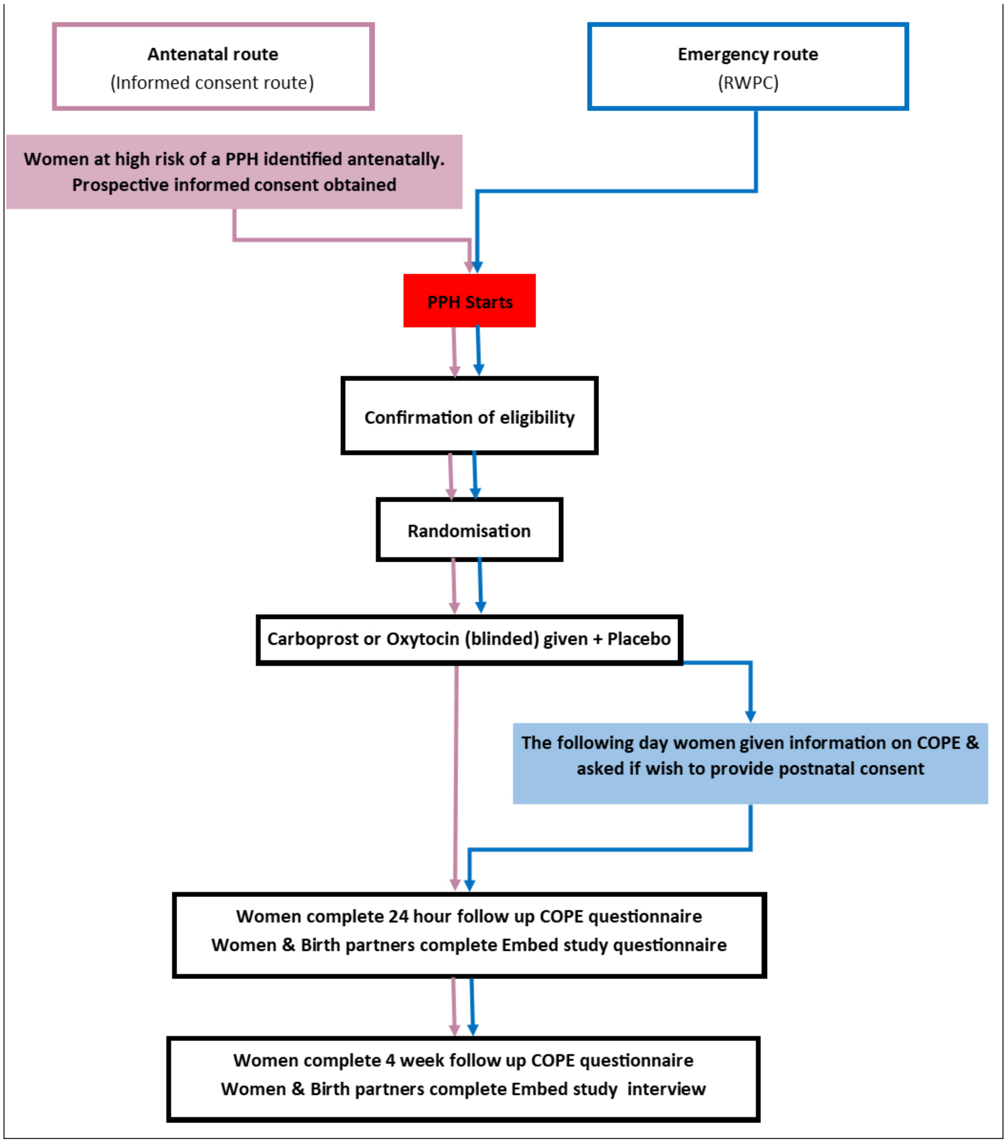

Figure 1. COPE RCT consent process

177x201mm (150 x 150 DPI)

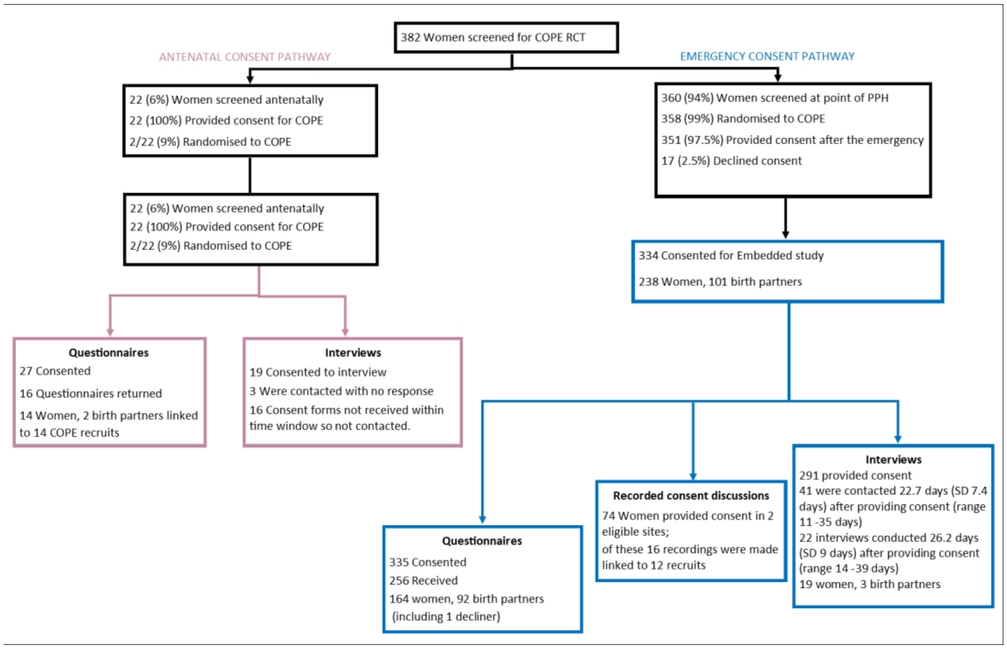

Figure 2: Embedded study recruitment

277x177mm (150 x 150 DPI)

COPE

The **C**arboprost or **O**xytocin **P**ostpartum haemorrhage **E**ffectiveness Study

Full explanatory title: Carboprost vs Oxytocin as the First Line Treatment of Primary Postpartum Haemorrhage. A Phase IV, double-blind, double-dummy, randomised controlled trial.

COPE Protocol v6.0 11/05/2022

Study Sponsor:

University of Liverpool Clinical Directorate,
Thompson Yates Building,
The Quadrangle,
Brownlow Hill,
Liverpool,
L3 5RB

EudraCT number: 2018-001829-11

CTA Reference Number: 04196/0052/001-0001

ISRCTN: 16416766

Research Ethics Ref: 18/WM/0227

Sponsor Ref: UoL001337

During the management of the COPE study, the Clinical Trials Research Centre (CTRC) and the Liverpool Cancer Trials Unit (LCTU) have merged to become the Liverpool Clinical Trials Centre (LCTC). The University of Liverpool is responsible for managing this study whilst the Liverpool Clinical Trials Centre (LCTC) runs it on a day-to-day basis. This study is funded by the National Institute for Health Research (NIHR) Health Technology Assessment (HTA) programme (16/16/06).

For peer review only

For peer review only - http://bmjopen.bmj.com/site/about/guidelines.xhtml

PROTOCOL APPROVAL

I, the undersigned, hereby approve this clinical study protocol:

Authorised by Chief Investigator:

Signature: ______________________________ **Date:** ____________

Prof Andrew Weeks
Professor of International Maternal Health
Sanyu Research Unit, Department of Women's and Children's Health
University of Liverpool
c/o University Department, First Floor
Liverpool Women's Hospital
Crown Street
Liverpool, L8 7SS

For peer review only

For peer review only - http://bmjopen.bmj.com/site/about/guidelines.xhtml

PROTOCOL APPROVAL

I, the undersigned, hereby approve this clinical study protocol:

Authorised on behalf of Sponsor:

Signature: ________________________ **Date:** ____________

Karen Wilding

Senior Clinical Research Governance Manager

University of Liverpool Clinical Directorate

Thompson Yates Building

The Quadrangle, Brownlow Hill,

Liverpool

L3 5RB

For peer review only

For peer review only - http://bmjopen.bmj.com/site/about/guidelines.xhtml

PROTOCOL APPROVAL

I, the undersigned, hereby approve this clinical study protocol:

Authorised by Lead Statistician:

Signature: ____ **Date:** ______________

Prof Carrol Gamble

LCTC Director

Liverpool Clinical Trials Centre

University of Liverpool

2nd Floor, Institute in the Park

Alder Hey Children's NHS Foundation Trust

Liverpool, L12 2AP

For peer review only

General Information

This document describes the COPE trial including detailed information about procedures and recruitment. The protocol should not be used as an aide-memoir or guide for the treatment of other patients; every care was taken in its drafting, but corrections or amendments may be necessary. These will be circulated to the registered investigators in the trial, but centres entering patients for the first time are advised to contact the coordinating centre (Liverpool Clinical Trials Centre, University of Liverpool) to confirm they have the most up to date version. Clinical questions relating to this trial should be referred to the Chief Investigator, Professor Andrew Weeks, via the LCTC.

This protocol defines the participant characteristics required for study entry and the schedule of treatment and follow-up. Participant recruitment will be undertaken in compliance with this document and applicable regulatory and governance requirements and waivers to authorise non-compliance are not permitted.

Incidence of protocol non-compliance, whether reported prospectively (e.g. where a treatment cannot be administered on a scheduled date as a result of public holidays) or retrospectively noted (e.g. as a result of central monitoring) are recorded as protocol deviations, the incidence of which are monitored and reported to trial oversight committees.

The template content structure is consistent with the SPIRIT (22) and has regard for the Health Research Authority guidance (1). Regulatory and ethical compliance information is located in section 12.

Relationship Statements

Roles and responsibilities are fully described in section 15.

The sponsor name is University of Liverpool who will formally delegate specific sponsoring roles to the Chief Investigator and Clinical Trials Unit, but remains legally responsible for the trial.

Clinical Trials Unit (CTU): LCTC at the University of Liverpool in collaboration with the chief investigator, Professor Andrew Weeks, will have overall management responsibility for the trial from a CTU perspective and will be responsible for the co-ordination of centres.

LCTC as part of the Liverpool Clinical Trials Collaborative has achieved full registration by the UK Clinical Research Collaboration (www.ukcrc.org) as their standards and systems were assessed by an international review panel as reaching the highest quality. LCTC has a diverse trial portfolio underpinned by methodological rigour, a GCP compliant data management system, and core standard operating procedures.

For peer review only - http://bmjopen.bmj.com/site/about/guidelines.xhtml

CONTACT DETAILS

Institutions

Sponsor:	Trial Management, Monitoring and Analysis:
The University of Liverpool<br>Clinical Directorate<br>Thompson Yates Building<br>The Quadrangle, Brownlow Hill,<br>Liverpool<br>L3 5RB<br><br>Tel: 00 44 (0) 7717 863747<br>Email: sponsor@liverpool.ac.uk	Liverpool Clinical Trials Centre<br>University of Liverpool<br>2nd Floor, Institute in the Park<br>Alder Hey Children's NHS Foundation Trust<br>Eaton Road<br>Liverpool<br>L12 2AP<br><br>Tel: +44(0)151 795 8760<br>E-mail: cope@liverpool.ac.uk

For peer review only

For peer review only - http://bmjopen.bmj.com/site/about/guidelines.xhtml

CONTACT DETAILS

Individuals

**Individual Authorised to Sign the Protocol and Protocol Amendments on behalf of the Sponsor:**	**Chief Investigator (CI):**
Karen Wilding<br>Senior Clinical Research Governance Manager<br>The University of Liverpool Clinical Directorate<br>Thompson Yates Building<br>The Quadrangle, Brownlow Hill,<br>Liverpool<br>L3 5RB<br><br>Tel: 00 44 (0) 7717 863747T: +44(0)151 794 8373<br>E-mail: sponsor@liverpool.ac.uk	Professor Andrew Weeks<br>Professor of International Maternal Health<br>Sanyu Research Unit, Department of Women's and Children's Health<br>University of Liverpool<br>c/o University Department, First Floor<br>Liverpool Women's Hospital<br>Crown Street<br>Liverpool, L8 7SS<br><br>Tel: +44(0)151 7959578<br>Fax: +44(0)151 7959553<br>E-mail: aweeks@liverpool.ac.uk
**Medical Expert who will Advise on Protocol Related Clinical Queries (If other than CI):**	**Medical Expert who will Evaluate SAE Reports (If other than CI):**
Dr Shireen Meher<br>Consultant in Maternal Fetal Medicine<br>Birmingham Women's & Children's NHS Foundation Trust<br>Birmingham Women's Hospital<br>Mindelsohn Way<br>Edgbaston<br>Birmingham, B15 2TG<br><br>Tel: 07734694660<br>E-mail: smeher@liverpool.ac.uk	Dr Dimitrios Siassakos<br>Honorary Consultant in Obstetrics<br>UCL EGA Institute for Women's Health<br>University College Hospital<br>Medical School Building<br>74 Huntley Street<br>London WC1E 6AU<br><br>Tel: 07930 624 894<br>E-mail: jsiasakos@me.com
**Medical Expert who will Evaluate SAE Reports (If other than CI or cross cover):**	**Statistical Team Lead:**
Mr Kim Hinshaw<br>Consultant Obstetrician & Gynaecologist<br>Obstetrics & Gynaecology<br>City Hospitals Sunderland NHS Foundation Trust<br>Sunderland Royal Hospital<br>Kayll Road<br>Sunderland, SR4 7TP<br><br>Tel: 0191 5410051<br>Fax: 0191 5699792<br>E-mail: kim.hinshaw@chsft.nhs.uk	Professor Carrol Gamble<br>Head of Biostatistics, LCTC Director<br>Liverpool Clinical Trials Centre<br>University of Liverpool<br>2nd Floor, Institute in the Park<br>Alder Hey Children's NHS Foundation Trust<br>Liverpool<br>L12 2AP<br><br>Tel: 0151 794 9759<br>E-mail: c.gamble@liverpool.ac.uk

For peer review only - http://bmjopen.bmj.com/site/about/guidelines.xhtml

**Embedded study:**	**Embedded Study Lead and Consent Lead:**
Professor Dame Tina Lavender<br>Professor of Maternal and Newborn Health<br>Department of International Public Health<br>Liverpool School of Tropical Medicine<br>Pembroke Place<br>Liverpool, L3 5QA<br><br>E-mail: Tina.Lavender@lstmed.ac.uk	Kerry Woolfall, PhD, Dr, Psychological Sciences<br>Senior Lecturer<br>MRC North West Hub for Trials Methodology Research<br>Institute of Psychology, Health and Society<br>Block B, Room B112, 1st Floor Waterhouse Building<br>Liverpool, L69 3GL<br><br>Tel: 0151 794 4634<br>E-mail: woolfall@liverpool.ac.uk
**Lead for Economic Evaluation:**	
Prof Dyfrig Hughes<br>Professor of Pharmacoeconomics<br>Centre for Health Economics and Medicines Evaluation<br>Ardudwy, Normal Site,<br>Bangor University<br>Holyhead Road<br>Bangor, LL57 2PZ<br><br>Tel: 01248 382950<br>E-mail: d.a.hughes@bangor.ac.uk	

Additional Contacts:

The contact details of other individuals involved in the trial are detailed in documents supplementary to the protocol and stored in the Trial Master File (TMF):

Contact	Document Title
Trial Management Group (TMG)<br>Trial Steering Committee (TSC)<br>Independent Data and Safety Monitoring Committee (IDSMC)	COPE Trial Oversight Committee Membership
Principal Investigators (PIs)	COPE Participating Centres

For peer review only

TABLE OF CONTENTS

PROTOCOL APPROVAL **2**
CONTACT DETAILS **6**
TABLE OF CONTENTS **9**
GLOSSARY **12**
1 PROTOCOL SUMMARY **13**
1.1 Schematic of Study Design 15
2 INTRODUCTION **16**
2.1 Background 16
2.2 Rationale 16
2.3 Risk and Benefits 17
2.4 Objectives 17
2.4.1 Primary Objective 17
2.4.2 Secondary Objective(s) 17
2.5 Outcome measures 17
2.5.1 Primary Outcome 17
2.5.2 Secondary Outcome 18
3 TRIAL DESIGN **19**
4 STUDY SETTING AND SELECTION OF CENTRES / CLINICIANS **20**
5 STUDY POPULATION **21**
5.1 Inclusion Criteria 21
5.2 Exclusion Criteria 21
5.3 Co-enrolment Guidelines 21
6 SCREENING, RECRUITMENT AND RANDOMISATION **22**
6.1 Screening 22
6.2 Recruitment 22
6.3 Consent Procedures 22
6.3.1 Information provision and postnatal consent 22
6.3.2 Principles of informed consent 23
6.3.3 Discharge/transfer to another hospital prior to postnatal consent 24
6.3.4 Maternal death prior to postnatal consent 26
6.3.5 Neonatal death prior to postnatal consent 26
6.4 Randomisation Procedures 26
6.5 Who is Blinded to Allocations 27
7 PARTICIPANT TIMELINE, ASSESSMENTS AND PROCEDURES **28**
7.1 Schedule for Follow-up 28
7.2 Procedures for Assessing Efficacy 29
7.3 Procedures for Assessing Safety 29
7.4 Other Assessments 29
7.4.1 Childbirth Experience Questionnaire (CEQ) 29
7.4.2 Health Economics assessments 29
7.5 Patient Transfer and Withdrawal 30
7.5.1 Discontinuation 30
7.5.2 Withdrawal of Consent 30
7.5.3 Participant Transfers 30
7.6 Loss to Follow-up 31

For peer review only - http://bmjopen.bmj.com/site/about/guidelines.xhtml

7.7 Trial Closure 31

8 EMBEDDED MIXED METHODS RESEARCH 32
8.1 Design 32
8.2 Selection of participants 32
8.3 Enrolment and procedure 33
8.3.1 Part A: Audio-recordings 33
8.3.2 Part B: Questionnaires 34
8.3.3 Part C: Interviews with women and their birth partner 34
8.3.4 Part D: Focus groups and/or interviews with the COPE practitioners 35

9 TRIAL TREATMENTS 36
9.1 Introduction 36
9.2 Formulation, Packaging, Labelling, Storage and Stability 36
9.2.1 Formulation 36
9.2.2 Packaging and Labelling 36
9.2.3 Storage and Stability 37
9.3 Distribution, Dispensing, Dosage and Administration of Study Treatment/s 37
9.3.1 Distribution and Dispensing 37
9.3.2 Dosage and Administration 37
9.4 Overdose 39
9.5 Delivery and Accountability of IMP at Trial Sites 39
9.5.1 Delivery 39
9.5.2 Accountability Procedures for Study Treatment/s 39
9.5.3 Expired and unused IMP stock 39
9.6 Concomitant Medications/Treatments 40
9.6.1 Medications Permitted/Not Permitted/ Precautions Required 40
9.6.2 Data on Concomitant Medication 40
9.7 Unblinding 40

10 SAFETY REPORTING 41
10.1 Time Period for Safety Reporting 41
10.2 Reference Safety Information 41
10.3 Flowchart of Adverse Events Reporting Requirements 42
10.4 Terms and Definitions 42
10.5 Notification of deaths 44
10.6 Severity / Grading of Adverse Events 44
10.7 Relationship to Trial Treatment 44
10.8 Expectedness 45
10.9 Follow-up After Adverse Events 45
10.10 Reporting Procedures 45
10.10.1 Non serious AEs 45
10.10.2 Non serious ARs 46
10.10.3 SAEs/ SARs/ SUSARs 46
10.10.4 Maintenance of Blinding 47
10.11 Responsibilities – LCTC 47
10.12 Safety reports 48
10.13 Urgent Safety Measures 48
10.14 Out-of-hours Medical Cover 48

11 STATISTICAL CONSIDERATIONS 49
11.1 Introduction 49
11.2 Method of Randomisation 49
11.3 Sample Size calculation 49
11.3.1 Feasibility (attaining recruitment targets) 50
11.4 Interim Monitoring and Analyses 50

For peer review only

11.5 Analysis Plan 50
11.5.1 Health Economic Analysis 50
11.5.2 Qualitative data analysis 51

12 REGULATORY AND ETHICAL APPROVALS 52
12.1 Statement of Compliance 52
12.2 Regulatory Approval 52
12.3 Ethical Approval 52
12.3.1 Ethical Considerations 52
12.4 Health Research Authority (HRA) Approval 53
12.4.1 Capacity and capability assessment 53
12.5 Protocol Deviation and Serious Breaches 53
12.6 Study Discontinuation 53

13 DATA MANAGEMENT AND TRIAL MONITORING 54
13.1 Source Documents 54
13.2 Data Capture Methods 54
13.3 Monitoring 55
13.3.1 Central Monitoring 55
13.3.2 Clinical Site Monitoring 55
13.4 Confidentiality 55
13.4.1 Hospital Episode Statistics (HES) 56
13.5 Quality Assurance and Control 56
13.6 Records Retention 56

14 INDEMNITY 58

15 ROLES AND RESPONSIBILITIES 59
15.1 Role of Study Sponsor and Study Funder 59
15.2 Funding and Support in Kind 59
15.2.1 NHS Research costs 59
15.2.2 NHS Support costs 60
15.2.3 Treatment costs 60
15.3 Protocol Contributors 60
15.4 Trial Committees 61
15.4.1 Trial Management Group (TMG) 61
15.4.2 Trial Steering Committee (TSC) 61
15.4.3 Independent Data and Safety Monitoring Committee (IDSMC) 61

16 PUBLICATION AND DISSEMINATION 62
16.1 Publication Policy 62
16.1.1 Authorship 62
16.2 Dissemination to Key Stakeholders 62
16.3 Data Sharing 62

17 CHRONOLOGY OF PROTOCOL AMENDMENTS 63
17.1 Version 1.0 (10/07/2018) 63
17.2 Version 2.0 (10/09/2018) 63
17.3 Version 3.0 (01/05/2019) 63
17.4 Version 4.0 (13/07/2020) 65
17.5 Version 5.0 (14/04/2021) 68
17.6 Version 6.0 (11/05/2022) 69

18 REFERENCES 71

Appendix 1. COPE Outcomes 73

For peer review only - http://bmjopen.bmj.com/site/about/guidelines.xhtml

GLOSSARY

AE	Adverse Event
AR	Adverse Reaction
CI	Chief Investigator
COS	Core outcome set
CRF	Case Report Form
CTIMP	Clinical Trial of an Investigational Medicinal Product
CTU	Clinical Trials Unit
EUDRACT	European Clinical Trials Database
GP	General Practitioner
HRA	Health Research Authority
IDSMC	Independent Data and Safety and Monitoring Committee
IMP	Investigational Medicinal Product
LCTC	Liverpool Clinical Trials Centre
MHRA	Medicines and Health Care Products Regulatory Agency
NRES	National Research Ethics Service
NIHR CRN	National Institute for Health Research Clinical Research Network
PI	Principal Investigator
QALY	Quality Adjusted Life Years
RCOG	Royal College of Obstetricians and Gynaecologists
R&D	Research & Development
REC	Research Ethics Committee
RM	Research Midwife (Registered)
RSI	Reference Safety Information
RSO	Research Support Office
SAE	Serious Adverse Event
SAR	Serious Adverse Reaction
SDV	Source Data Verification
SMPC	Summary of Product Characteristics
SMR	Scottish Morbidity Records
SOP	Standard Operating Procedure
SSAR	Suspected Serious Adverse Reaction
SUSAR	Suspected Unexpected Serious Adverse Reaction
TMG	Trial Management Group
TSC	Trial Steering Committee
UAR	Unexpected Adverse Reaction
WHO	World Health Organisation

For peer review only

1 PROTOCOL SUMMARY

**Full Title:**	Carboprost vs Oxytocin as the first line treatment of primary postpartum haemorrhage. A Phase IV, double-blind, double-dummy, randomised controlled trial.
**Acronym:**	COPE
**Phase:**	IV
**Target Condition:**	Primary Postpartum haemorrhage (PPH)
**Sample size**	3,948 participants
**Main Inclusion Criteria:**	• ≥ 16 years of age<br>• Requirement for medical treatment for primary PPH
**Main Exclusion Criteria:**	• Known to have opted out of participation antenatally<br>• Known oxytocin or carboprost hypersensitivity<br>• Known active cardiac or pulmonary disease<br>• Known to have previously been treated as part of COPE<br>• Has already received carboprost prophylactically for postpartum haemorrhage<br>• Has already received uterotonic drug treatment for postpartum haemorrhage (this does not include PPH prophylaxis)<br>• Stillbirth
**Study Centres and Distribution:**	NHS hospital maternal units in the UK
**Patient Study Duration:**	Duration per participant: 4 weeks
**Overall Study duration:**	48 months
**Agent/ Intervention:**	**Intervention:** Carboprost 250 micrograms by deep intramuscular injection and 1 ml placebo by slow intravenous injection over 2 minutes<br><br>**Control:** Oxytocin (5 international units (IU) for caesarean section or 10 IU for vaginal delivery) by slow intravenous injection over 2 minutes and 1 ml placebo by deep intramuscular injection
**Primary objective:**	To compare carboprost with oxytocin as initial treatments for women with clinically diagnosed PPH after giving birth in UK hospitals.

For peer review only

**Secondary objective:**	1. To assess the relative cost-effectiveness of the use of carboprost and oxytocin as initial treatments for women with clinically diagnosed PPH.<br>2. To explore the views of participants and their carers about their experiences of the two treatments and the consent process.
**Primary outcome measures:**	Blood transfusion defined as "Any red blood cell transfusion or cell salvage of ≥300mls commenced any time between randomisation and 48 hours after randomisation (or hospital discharge if earlier than 48 hrs)"
**Secondary outcome measures:**	1. Volume of blood transfusion<br>2. Use of a further uterotonic drug<br>3. Composite outcome of any organ dysfunction based on WHO near-miss approach for maternal health (2)<br>4. Hysterectomy<br>5. Blood loss<br>6. Blood loss ≥ 1000 mls<br>7. Haemoglobin<br>8. Shock<br>9. Maternal death<br>10. Non pharmacological approach to treat or investigate bleeding<br>11. Manual removal of placenta<br>12. Adverse reactions of particular interest (as listed in section 10.1)<br>13. 'Skin to skin' care with baby within the first hour after birth<br>14. Separation from new-born in first hour after birth<br>15. Breastfeeding<br>16. Childbirth Experience Questionnaire (CEQ)<br>17. Resource use
**Embedded Mixed Methods Research**	For at least the first 9 months of recruitment, we will conduct qualitative research to explore the views and experiences of women recruited to the trial, their partners, and practitioners involved in recruitment and consent in COPE.

For peer review only - http://bmjopen.bmj.com/site/about/guidelines.xhtml

1.1 Schematic of Study Design

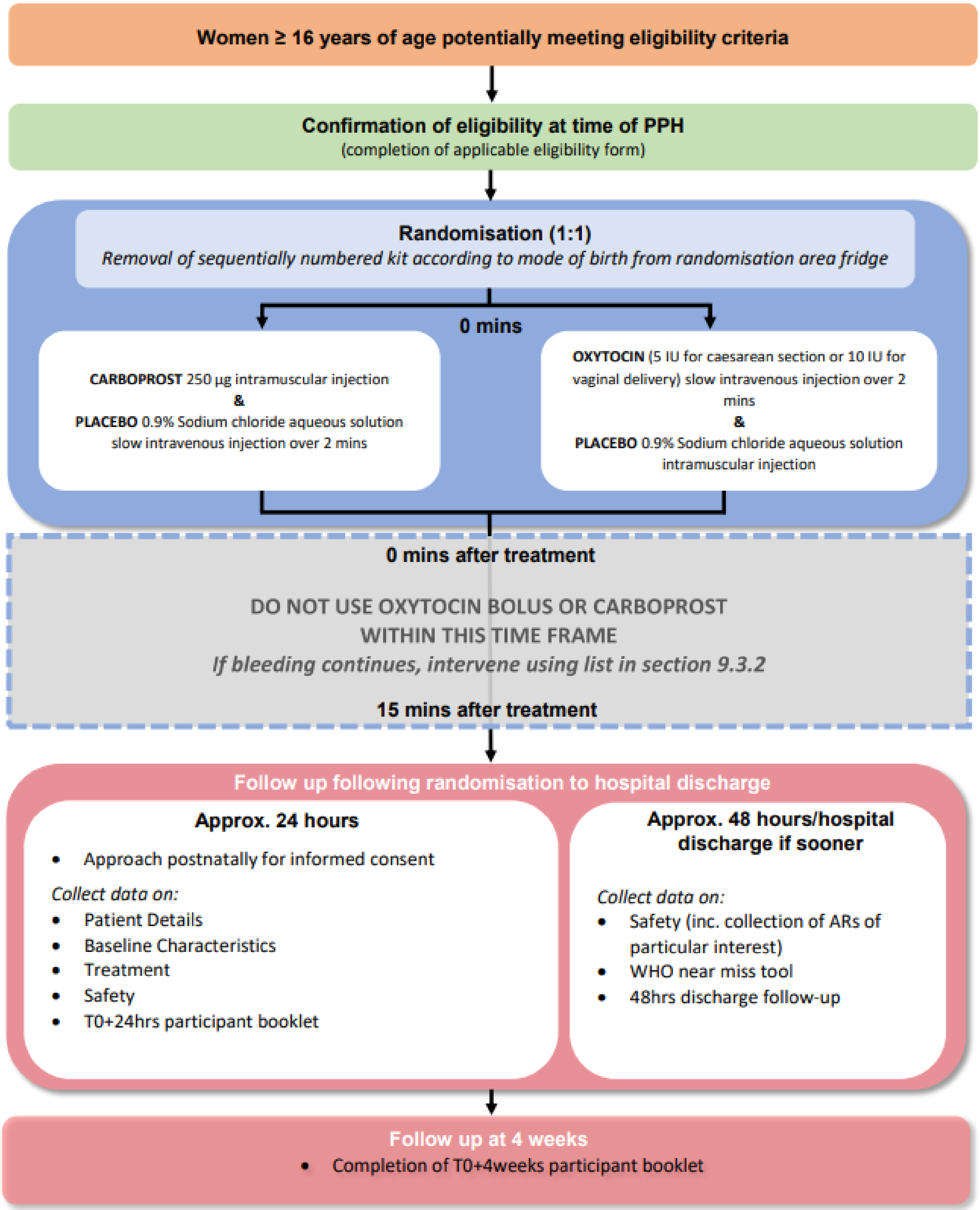

2 INTRODUCTION

2.1 Background

Bleeding after childbirth (postpartum haemorrhage, PPH) occurs after around 5% of births (depending on definition) and causes the death of 75,000 women worldwide each year (3). It is the second leading cause of direct maternal deaths in the UK (19 deaths between 2013-2015) (4). Significant maternal morbidity may also result from weakness secondary to anaemia, delayed recovery, psychological trauma, difficulties with breastfeeding and poor bonding with the newborn. Available treatments, including drugs with known side effect profiles, blood transfusion and invasive or surgical procedures such as hysterectomy can have substantial negative impact on the woman's recovery, long-term health and sense of wellbeing.

PPH is a clinical emergency. The bleeding in PPH is unpredictable and difficult to quantify, and so most clinicians treat early, as soon as they are unhappy with the bleeding. As a result of the unpredictability of PPH and difficulties in gaining emergency consent, there are few randomised trials of PPH treatments. The evidence used in guidelines is therefore based largely on studies of prophylaxis and small observational studies (5). The National Institute for Health Research (NIHR) Health Technology Assessment (HTA) programme has therefore called for research into PPH treatments as one of its research priorities. Recent advances in emergency intrapartum consent pathways, developed in part by applicants in partnership with consumer groups, have facilitated recruitment (6-9).

2.2 Rationale

As uterine atony is the most common cause, intravenous (i.v.) oxytocin is universally recommended as first line therapy. However, the recommended dosage varies. Whilst the National Institute for Health and Care Excellence (NICE) recommends 10 international units i.v.; the Royal College of Obstetricians and Gynaecologists (RCOG) guidelines and *Mothers and Babies: Reducing Risk through Audits and Confidential Enquiries across the UK* (MBRRACE-UK) suggest 5 international units (5, 10, 11). There are no direct comparisons of the two doses. Although studies suggest that 3 international units is adequate (12, 13), we will use a higher dose in this study (5-10IU) to prevent criticism of inadequate dosing. The only risk of this is hypotension that occurs with rapid injection, however this was not seen in a recent randomised controlled trial in which 517 women were given 10 international units of oxytocin as an i.v. bolus over 1 minute (14). The need for slow intravenous injection will be emphasised in training (see section 2.3 below). In women who have a caesarean section (who usually have intravenous oxytocin prophylaxis, have usually just had a spinal anaesthetic inserted and who have greater blood loss) we will give only 5IU IV in line with anaesthetic recommendations.

The PPH rate is increasing, and this may be partially related to the overuse of oxytocics in labour (11). Laboratory studies on myometrium suggests that repeated use of oxytocics leads to the saturation of oxytocin receptors and reduced efficacy of oxytocin as a therapy (15). Attention has therefore turned to the use of prostaglandins as an alternative mechanism for improving the strength of uterine contractions in the event of uterine atony. Misoprostol is a prostaglandin E1 analogue, which is convenient for both storage and administration. It has been studied extensively for both prophylaxis and treatment of PPH, and found to be less effective than oxytocin (3) and of no additional benefit when given in addition to standard drugs for PPH treatment (16). Carboprost is a prostaglandin F2a analogue that is given intramuscularly (i.m.). There are 13 small studies of carboprost for PPH prophylaxis. They show a reduction in blood loss compared with conventional uterotonics, but diarrhoea,

For peer review only - http://bmjopen.bmj.com/site/about/guidelines.xhtml

vomiting, fever or hypertension affects about 15% of those treated (17). Carboprost is also more expensive than oxytocin. There are no studies of carboprost for PPH treatment (18), but NICE recommends carboprost as a second line treatment. NICE, and others, have suggested the need for a major randomised trial to ascertain both the effectiveness of carboprost and its optimal position in a PPH treatment pathway (3, 5).

2.3 Risk and Benefits

Oxytocin is the standard first line treatment for atonic postpartum haemorrhage. It has been shown to cause effective uterine contraction, is low cost and relatively free from side-effects. Oxytocin can commonly cause headache, nausea and vomiting. The benefit of repeated doses (for example giving 10IU prophylaxis and then 10IU treatment shortly after) has however been questioned (3, 15), especially given that pharmacokinetic studies suggest that the optimal dosage is just 3IU (12). Furthermore, a bolus dose of intravenous oxytocin causes a rapid but transient fall in blood pressure by around 20mmHg (19) and has been implicated in maternal death during PPH (20). Repeated doses of oxytocin also cause water retention. It is therefore of uncertain benefit, and not without risks.

Carboprost, a prostaglandin F2α analogue, is usually reserved for second line management of atonic PPH. A systematic review of 13 small randomised trials comparing the efficacy of carboprost and conventional uterotonics for PPH prophylaxis found that it was associated with less blood loss, but around 15% of women suffered side effects including diarrhoea, vomiting, fever or hypertension (17).

2.4 Objectives

2.4.1 Primary Objective

The aim of the research is to compare intramuscular carboprost 250 micrograms with intravenous oxytocin (5 international units (IU) for caesarean section or 10 IU for vaginal birth) by slow injection over 2 minutes for the initial treatment for women with clinically diagnosed postpartum haemorrhage after giving birth in UK hospitals.

2.4.2 Secondary Objective(s)

1. To assess the relative cost-effectiveness of the use of carboprost and oxytocin as initial treatments for women with clinically diagnosed PPH.
2. To explore the views of participants and their carers about their experiences of the two treatments and the consent process.

2.5 Outcome measures

The outcomes selected for this study include the recently developed Core Outcome Set (COS) for PPH treatment trials (Appendix 1).

2.5.1 Primary Outcome

Blood transfusion defined as 'any red blood cell transfusion or cell salvage of ≥ 300 mls commenced any time between randomisation and 48 hours after randomisation (or hospital discharge if earlier than 48 hours'.

For peer review only - http://bmjopen.bmj.com/site/about/guidelines.xhtml

2.5.2 Secondary Outcome

All secondary outcomes are collected prior to discharge or 4 weeks whichever is earliest unless otherwise specified.

1. Volume of blood transfusion
2. Use of a further uterotonic drug
3. Composite outcome of any organ dysfunction[1]
4. Hysterectomy
5. Blood loss
6. Blood loss ≥ 1000mls
7. Haemoglobin
8. Shock
9. Maternal death
10. Non-pharmacological approach to treat or investigate bleeding[2]
11. Manual removal of placenta
12. Adverse reactions of particular interest (as listed in section 10.1)
13. Skin to skin care with baby within the first hour after birth
14. Separation from new-born in first hour after birth
15. Breastfeeding
16. Childbirth Experience Questionnaire (CEQ)
17. Resource use[3]

For at least the first 9 months of recruitment, we will conduct qualitative research to explore the views and experiences of women recruited to the trial, their partners, and practitioners involved in recruitment and consent in COPE.

[1] Based on definitions within the WHO Maternal 'near-miss' Approach for maternal health (2).

[2] Interventions include: laparotomy, internal uterine tamponade with balloon or packing with gauze, arterial embolisation, removal of retained placenta or placental tissue, examination in theatre under anaesthetic and external uterine compression (bimanual or other).

[3] Number of units of blood transfused will be collected for resource use; transfers to higher care units will be collected within resource use.

For peer review only

3 TRIAL DESIGN

This is a double-blind, double-dummy, randomised controlled trial.

To determine the feasibility of answering the clinical question, an internal pilot examining rates of recruitment and consent, data completeness and sample size assumptions will be carried out. The timing of the internal pilot will be informed by the decision of the IDSMC and correspond to ability to reasonably estimate the primary outcome event rate with reasonable precision.

Criteria for progression from internal pilot to full study are:

Recruitment
Taking into account the pilot study and the ratio of vaginal to caesarean births, we will estimate the total recruitment period required to achieve the target sample size. If this is achievable within the recruitment period agreed with the funders then we will proceed to the main trial. If it exceeds this duration we will introduce ways to reduce this, and seek approval from the funder. If no obvious solutions exist and the funder is unable to support the time extension required then we will abandon the main trial.

Consent
If the rate of informed consent for follow-up and data use is 80% or more, proceed to main trial. If 60% to 80%, then review qualitative data to identify any aspects amenable to change, and proceed. If less than 60%, and no obvious solutions exist, then abandon the main trial.

Completeness of primary outcome data
If primary outcome data are available for over 95% of participants, then proceed. If 75-95%, identify aspects amenable to change and proceed to main trial as amended. If available for less than 75% of participants randomised, and no obvious solutions exist, abandon the main trial.

Sample Size estimation
The Independent Data Safety and Monitoring Committee (IDSMC) will be asked to review the assumptions made in the sample size calculation. As a result the numbers required may be increased but not decreased. If an increase in sample size is recommended, then this will be discussed with funders and informed by ability to deliver within time and budget.

For peer review only

4 STUDY SETTING AND SELECTION OF CENTRES / CLINICIANS

The study will take place in approximately 40 UK NHS hospital maternity units.

Criteria for the selection of centres will be determined by the Trial Management Group (TMG) and will be described in the supplementary document 'COPE Site Suitability Assessment'.

Initiation of centres will be undertaken in compliance with LCTC Standard Operating Procedures (SOPs). Centres fulfilling the criteria will be selected to be recruitment centres for the COPE trial and will be opened to recruitment upon successful completion of all global (e.g. Research Ethics Committee (REC) and Medicines and Healthcare Products Regulatory Agency (MHRA)) and study-specific conditions (e.g. site personnel training requirements) and once all necessary documents have been returned to LCTC as detailed in the trial 'greenlight' checklist.

Participating centres will be listed in a log, maintained separately to the protocol.

For peer review only

For peer review only - http://bmjopen.bmj.com/site/about/guidelines.xhtml

5 STUDY POPULATION

The requirements of Good Clinical Practice (GCP) are that a doctor should either recruit or supervise the recruitment of individuals to a Clinical Trial of an Investigational Medicinal Product (CTIMP). Thus women giving birth at home or in stand-alone midwifery units will not be eligible to participate.

5.1 Inclusion Criteria

- ≥ 16 years of age
- Requirement for medical treatment for primary PPH

5.2 Exclusion Criteria

- Known to have opted out of participation antenatally
- Known oxytocin or carboprost hypersensitivity
- Known active cardiac or pulmonary disease
- Known to have previously been treated as part of COPE
- Has already received carboprost prophylactically for postpartum haemorrhage
- Has already received uterotonic drug treatment for postpartum haemorrhage (this does not include PPH prophylaxis)
- Stillbirth

5.3 Co-enrolment Guidelines

Individuals who are currently recruited into other intrapartum interventional studies may be approached for the COPE trial upon agreement with the study Chief Investigator. Where recruitment into another trial is considered to be appropriate and without having any detrimental effect on the COPE trial this must first be discussed with the LCTC who will contact the Chief Investigator, Prof Andrew Weeks.

For peer review only

For peer review only - http://bmjopen.bmj.com/site/about/guidelines.xhtml

6 SCREENING, RECRUITMENT AND RANDOMISATION

6.1 Screening

Screening and eligibility information will be collected by completion of participant screening and eligibility forms. These forms will provide important information for monitoring purposes.

6.2 Recruitment

PPH is an unpredictable clinical emergency occurring after around 5% of births. COPE participants will be recruited via the following emergency pathway:

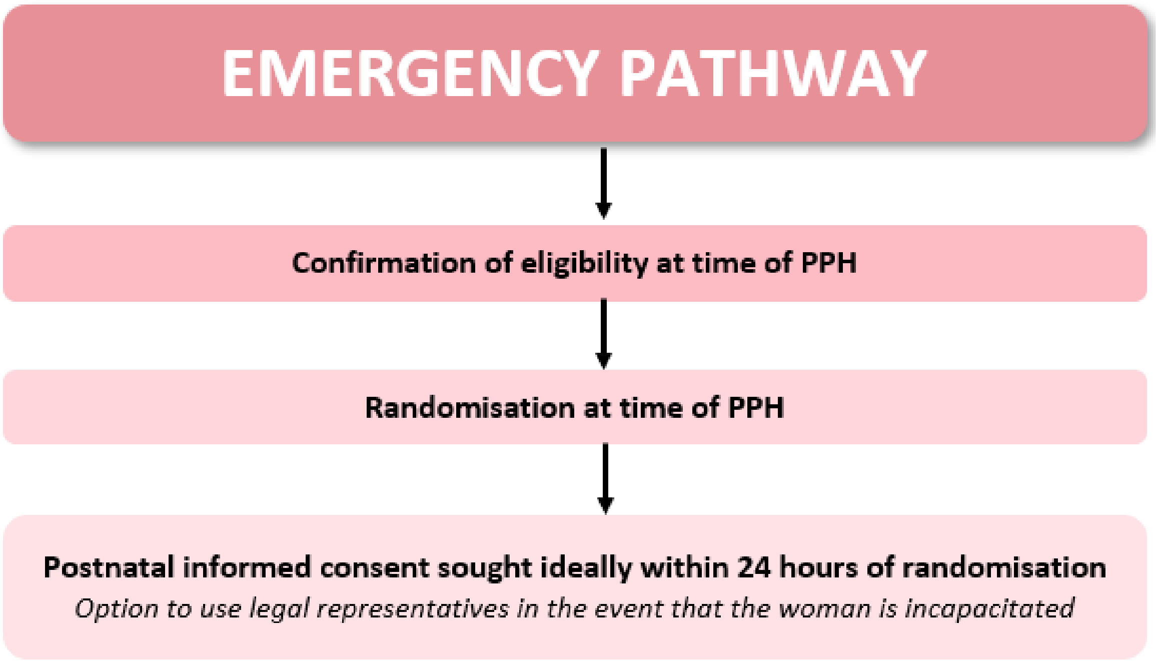

6.3 Consent Procedures

Randomisation and treatment will occur if eligibility criteria are met after child birth. Anonymous data, for the purposes of the recipients, will be sent to the LCTC for processing under public interest to allow monitoring of safety. Personal identifiable information will be subsequently sent to LCTC following written informed consent.

6.3.1 Information provision and postnatal consent

A variety of strategies will be used to advertise the study to raise awareness amongst women antenatally, including: patient posters, antenatal clinical discussions, and social media campaigns. Study advertising will include details of how women can contact the local study team to discuss the study.

Once a woman has been diagnosed with PPH, a critical clinical emergency situation exists. Eligible women have a life threatening condition and risk of death is highest early after delivery.

For peer review only - http://bmjopen.bmj.com/site/about/guidelines.xhtml

Their mental capacity, physical and emotional state may be diminished as a result of blood loss, pain, or by drugs administered during the labour.

Eligible women will be randomised into the trial in the emergency situation where treatment needs to be given urgently and there is no time for prior consent from a Legal Representative. An overview of the emergency pathway and postnatal consent procedure is provided in section 6.2. The treating clinician may briefly explain the study to the woman if it is deemed appropriate. Note that it is not the intention to obtain verbal consent in this emergency situation. For women who are randomised, but they or their legal representatives do not provide consent postnatally, anonymous data, for the purposes of the recipients (LCTC) will be sent for processing under public interest to allow monitoring of safety. The provision of anonymous data from sites to the LCTC is in the line with the General Data Protection Regulations (2018) and Common Law. Where consent is not provided no contact will be made with the woman's GP.

6.3.1.1 Consent provision where capacity is regained promptly

Postnatally, once the woman is stable and ideally within 24 hours or prior to discharge/transfer to another hospital, full study information will be provided to the woman. Before approaching women, the trial recruiter will firstly check whether the timing is appropriate with the clinical team. Permission will be sought for disclosure of identifiable data and continued follow-up. The woman will be asked to sign the "Postnatal (emergency pathway) Patient Information Sheet and Consent Form".

6.3.1.2 Consent provision where capacity is not regained or is delayed

Before approaching women, the trial recruiter will firstly check whether the timing is appropriate with the clinical team. Should it be apparent that incapacity is likely to continue, full study information will be provided as soon as practicable to her Personal Legal Representative (PeR) or Professional Legal Representative (PrR), if PeR is not available. Permission will be sought for disclosure of identifiable data and continued follow-up. The PeR or PrR will be asked to sign the "Postnatal (emergency pathway) Personal Legal Representative Information Sheet and Consent Form" or "Postnatal (emergency pathway) Professional Legal Representative Information Sheet and Consent Form", respectively.

The consent given by an appropriate legal representative on behalf of a woman lacking capacity to do so for themselves shall represent the presumed will of the incapacitated woman and as such it is not mandatory to re-approach women recruited in such a manner should they regain their capacity to give consent during the course of their participation in COPE.

Legal representatives will be made aware that, should this circumstance arise during the trial participation period (4 weeks), the woman will be provided with information about COPE but will not be approached to provide their own written consent. The woman will be made aware that they can withdraw from the trial at any time by revoking the informed consent provided by their legal representative as stated in section 7.5.2.

6.3.2 Principles of informed consent

In obtaining and documenting informed consent, the investigator/delegate should comply with applicable regulatory requirements and should adhere to the principles of GCP and to the ethical principles that have their origin in the Declaration of Helsinki.

At the appropriate time, the objectives, risks and inconveniences of the trial and the conditions under which it is to be conducted are to be provided to the woman or their legal representative by staff with experience in obtaining informed consent. A Patient Information Sheet and

For peer review only - http://bmjopen.bmj.com/site/about/guidelines.xhtml

Consent form, describing in detail the trial interventions, trial procedures and risks will be approved by a REC, and the woman (or their Legal Representative) will be asked to read and review the document. Upon reviewing the document, the investigator will explain the research study to the woman (or their Legal Representative). This information will emphasise that participation in the trial is voluntary and that the woman may withdraw from the trial at any time and for any reason. All women (or their Legal Representative) will be given opportunity to ask any questions that may arise, and should have the opportunity to discuss the study with their friends and family, and time to consider the information prior to agreeing to consent. A contact point where further information about the trial may be obtained will be provided.

Women will have as long as possible to consider participation in COPE. Research staff should aim to meet the woman to seek consent within 24 hours, or prior to discharge or transfer to another hospital. The woman or their Legal Representative will be asked to sign and date the informed consent document. Both the person obtaining consent and the woman (or their Legal Representative) must personally sign and date the form. A copy of the consent form will be given to the woman (or their Legal Representative) for their records. The original copy will be filed in the Investigator Site File, a copy filed in the woman's notes and a further copy should be sent to the LCTC. Following provision of consent, a COPE GP letter will be sent to the woman's GP by the recruiting site.

The woman (or their Legal Representative) may, without being subject to any resulting detriment, withdraw consent for trial participation any time by revoking their informed consent as stated in section 7.5.2. In this circumstance or where the woman (or their Legal Representative) declines to provide consent, the rights and welfare of the woman will be protected and the quality of medical care will not be adversely affected.

6.3.2.1 Definitions of legal representatives

England and Wales:

- Personal Legal Representative i.e. a person not connected with the conduct of the trial who is suitable to act as the legal representative by virtue of their relationship with the adult, and is available and willing to do so. If one is not available:
- Professional Legal Representative i.e. a person not connected with the conduct of the trial who is the doctor primarily responsible for the medical treatment of the adult, or a person nominated by the healthcare provider.

Scotland:

- Personal Legal Representative i.e.
    - Adult's Welfare Guardian or Welfare Attorney, or if not appointed:
    - The adult's nearest relative, if neither are reasonably contactable:
- Professional Legal Representative i.e. a person not connected with the conduct of the trial who is the doctor primarily responsible for the medical treatment of the adult, or a person nominated by the healthcare provider.

6.3.3 Discharge/transfer to another hospital prior to postnatal consent

It is expected that consent will be sought for all women prior to discharge/transfer to another hospital (in the event that the woman dies prior to consent being sought refer to section 6.3.4).

Where consent is not sought prior to discharge/transfer the following should occur:

For peer review only - http://bmjopen.bmj.com/site/about/guidelines.xhtml

The Research Midwife (RM), or other designated member of the research team at site, will call the woman within 5 working days of randomisation to inform them of their involvement in the trial and provide details. If the woman cannot be contacted, then the process for postal consent should be followed.

If the woman is willing to provide consent, the RM will outline the two different ways in which consent could be provided, as per the below, and ask the woman which way they would prefer. If during the conversation the woman is clear that they do not wish to participate then no trial documentation/correspondence or follow up documentation will be sent.

Two methods are provided for confirming consent:

1. **Electronic-consent:** If the woman would prefer to use Electronic-consent then their email address must be provided to the site. The person speaking with the woman must ensure that the woman is fully informed that their email address will be used for this purpose. Full guidance on obtaining consent electronically can be found in COPE Electronic-consent guidance document.

   LCTC will facilitate the collection of validated E-consent. The E-consent request will expire after 4 weeks from the date sent. Once complete, all signatories and LCTC will receive a copy of the final signed consent form.

2. **Postal consent:** the RM, or other designated member of the research team at site, will post the below items to the woman's address within 5 working days:
   - Participant covering letter
   - Participant Information Sheet and Consent form (Home version)

   The covering letter will confirm that the woman has 4 weeks from the date of the letter to return the consent form confirming whether they would like to continue participation in the trial.

   When the consent form is received by the RM, they will sign or organise for an authorised individual to sign the consent form. When signed, the RM will send a copy to the LCTC.

Note: Audio-recordings of telephone discussions are not required.

If no response is received within 2 weeks (14 days), the RM, or other designated member of the research team at site will make a follow up call to the woman to check that they have received the information. Postal consent documents/e-consent email will be re-sent/forwarded, respectively, if the site team member is unable to contact the woman or information has not been received.

If no response is received via E-consent or postal consent within 4 weeks (28 days), the woman's identifiable data will not be included within the trial as confirmed in the telephone conversation/correspondence. Consent is being sought in this scenario for the disclosure of confidential information in order to avoid a breach of the common law duty of confidentiality. The four-week questionnaire will be sent by post with another consent form to give the woman another opportunity to provide consent. If we find that consent in this scenario is not practicable and results in informative missing data (e.g. linked to severity of condition or duration of hospital stay) a future Section 251 application may be made.

For peer review only - http://bmjopen.bmj.com/site/about/guidelines.xhtml

Collection of personal identifiable information is restricted to the data requested on the consent form. All other data are anonymous to the purposes of the recipients (LCTC) and processed under public interest and are necessary for monitoring of safety.

6.3.4 Maternal death prior to postnatal consent

This is likely to be a very rare occurrence. However, in the event that a woman dies before consent has been sought, the attending RM will obtain information from colleagues and bereavement counsellors to establish the most appropriate practitioner to notify the Personal Legal Representative of the research involvement.

Consent can be sought from Personal legal representatives following the death of their relative and prior to the Personal Legal Representative's departure from the hospital. However, it is at the discretion of the site staff to determine if this is appropriate for each individual family. In this situation, the "Bereaved Postnatal (emergency pathway) Personal Legal Representative Information Sheet and Consent Form" would be used.

If consent is not sought prior to the Personal Legal Representative's departure from hospital then the Personal Legal Representative will be notified and consent can be sought by an appropriate practitioner at a bereavement follow-up visit. If it is not possible to obtain Personal Legal Representative consent then Professional Legal Representative consent form can be completed.

In addition, all deaths, irrespective of the consent status, should be reported on a Serious Adverse Event Case Report Form (CRF) and securely emailed to the LCTC within 24 hours of the research team becoming aware (see section 10.5).

6.3.5 Neonatal death prior to postnatal consent

This is likely to be a rare occurrence. However, if a participant experiences the death of their baby before consent has been sought, the attending RM will obtain information from colleagues and bereavement counsellors to establish the most appropriate practitioner to notify the participant of the research involvement.

Consent will be sought if considered appropriate; however, it is at the discretion of the site staff to determine if this is appropriate for each individual participant. In this situation, the usual information sheet and consent form would be used.

6.4 Randomisation Procedures

Participants will be randomised in a 1:1 ratio using random variable block size, stratified by mode of birth (caesarean section or vaginal birth).

Centres will be provided with a series of sequentially numbered, sealed treatment kits to be received by the pharmacy department and distributed to an appropriate secure location within randomisation areas i.e. delivery suite for ready access upon presentation of eligible patients.

Each kit will contain either:

a) An ampoule of carboprost and an ampoule of placebo or
b) An ampoule of oxytocin and an ampoule of placebo.

Departments will be provided with case report forms (CRFs) to be completed promptly at the time of recruitment (to document key screening/eligibility and baseline data).

For peer review only - http://bmjopen.bmj.com/site/about/guidelines.xhtml

After eligibility is confirmed by an authorised medical doctor, the next sequentially numbered treatment kit for the particular mode of birth (vaginal or caesarean section) should be selected and administered. The treatment kit's identification number is the randomisation number for the woman and should be recorded in their medical notes and on the trial CRFs.

All women randomised in to COPE (treatment kit removed from fridge following confirmation of eligibility) who go on to receive the COPE treatment or an alternative medicinal treatment for PPH should be approached for their consent and followed up as a COPE participant as per COPE protocol. If a treatment kit was removed from the fridge but a medicinal treatment for PPH was not administered (COPE treatment kit or other) then consent does not need to be sought and they should not be followed up as a COPE participant.

A member of the trial site team as delegated on the delegation log will periodically check to ensure that there are adequate stocks of trial treatment kits, that the correct number of treatment kits are present and intact and that the sequential numbering system is maintained. Any discrepancies will be immediately reported to the LCTC.

6.5 Who is Blinded to Allocations

This is a double-blind study and all individuals involved in the conduct and delivery of the trial, except for the randomising statistician, or those unblinded to individual cases as a requirement (e.g. for safety reporting), will be blinded to treatment allocations. Statisticians involved in monitoring will be unblinded following determination of participant inclusion within each analysis population.

In case of the need of emergency unblinding, unblinding envelopes will be provided and stored at an agreed location within the site that is readily accessible at time of need. The construction of these envelopes is resistant to accidental damage or tampering and contents cannot be viewed without fully opening.

The site team will periodically check to ensure that the appropriate number of unblinding envelopes are present and intact. Any discrepancies will be immediately reported to the LCTC.

For unblinding procedures see section 9.7.

For peer review only

For peer review only - http://bmjopen.bmj.com/site/about/guidelines.xhtml

7 PARTICIPANT TIMELINE, ASSESSMENTS AND PROCEDURES

7.1 Schedule for Follow-up

Participants will be recruited and followed up to a maximum of 4 weeks postnatally.

		Follow-Up Schedule			
**Procedures**		**Screening**	**Baseline (T0)[1]**	**T0 - 48 hrs or hospital discharge if sooner[3]**	**T0 + 4 wks[4]**
Assessment of Eligibility Criteria		X	X		
Confirmation of Full Eligibility by an authorised medical doctor			X		
Randomisation			X		
Administration of Study Intervention			X		
Signed Postnatal Consent Form[2]				X*	
Baseline Characteristics			X		
Clinical Outcomes	The use and timing of additional uterotonics			X	
	Manual removal of placenta			X	
	Hysterectomy			X	
	Blood loss at birth			X	
	Non-pharmacological approach			X	
	Skin to skin care with baby			X*	
	Separation from new-born in first hour after birth			X*	
	Breastfeeding initiation (first 24hrs)			X*	
	Blood transfusion or cell salvage			X	
	Volume of blood transfusion			X	
	Haemoglobin[3]			(X)*	
	Shock			X	
	Exclusive breastfeeding (at 48hrs/ discharge if sooner)			X	
	Adverse Reactions of particular interest		X	X	
	Outcome of any organ dysfunction			X	(X)
	Maternal death[5]		(X)	(X)	(X)
	Exclusive breastfeeding (at 4 weeks)				X
Assessment of Serious Adverse Events[6]			X	X	(X)
Childbirth Experience Questionnaire					X
EQ-5D-5L questionnaire and EQ-VAS				X*	X
Healthcare Resource Utilisation Questionnaire					X

For peer review only

(X) – As indicated/appropriate.
[1] At baseline, all procedures should be done before study intervention.
[2] Postnatal (emergency pathway) consent is obtained after childbirth ideally within 24 hrs or prior to discharge / transfer to another hospital. If consent is not obtained prior to discharge, the appropriate consent procedures should be followed as per section 6.3.
[3] Haemoglobin (in non-transfused women only) will be ideally obtained postnatally on the day following birth (12-36 hours post birth) or at discharge, whichever is soonest. For more details, see Appendix 1.
[4] Participant to be contacted initially using her favoured mode of communication (letter or telephone), with a letter or telephone reminder if no response within 2 weeks
[5] All deaths collected from the time of randomisation until hospital discharge or 4 weeks, whichever is earlier.

[6]Serious Adverse Events and Serious Adverse Reactions will be actively monitored and reported from the time of randomisation until hospital discharge or 4 weeks, whichever is sooner. ARs of particular interest collected on a separate single timepoint CRF, as per section 10.
* To be completed at 24 hours after randomisation.

7.2 Procedures for Assessing Efficacy

Clinical efficacy outcomes will be collected at 24 hours and at 48 hours or at hospital discharge, if sooner. Details of the outcomes (with the timing of assessment and definitions) are included in Appendix 1. COPE OUTCOMES.

7.3 Procedures for Assessing Safety

The Principal Investigator (PI) or delegated research staff will be monitoring and reporting all relevant adverse events (see section 10) from randomisation until hospital discharge or 4 weeks, whichever is sooner.

7.4 Other Assessments

7.4.1 Childbirth Experience Questionnaire (CEQ)

The CEQ was developed in 2010 to measure the impact of an intervention on a woman's childbirth experience. It includes four main aspects of the childbirth experience: Own capacity, Professional support, Perceived safety and Participation.

In COPE, the UK-validated CEQ will be completed by the participants as a paper or telephone questionnaire at 4 weeks.

7.4.2 Health Economics assessments

7.4.2.1 Resource use and costs

Resource use will be based on:

(1) Entries made in designated sections of participants' CRFs. The CRF will be used to record data on procedures (surgical) and interventions (including units of blood products transfused) as well as dates of patient admission and discharge. This will be completed prior to discharge.

(2) A resource use questionnaire, administered at the 4-week follow-up, using the participant's favoured mode of communication (letter, telephone) with a letter or telephone reminder if no response provided within 2 weeks.

(3) Hospital Episode Statistics (HES) and Scottish Morbidity Records (SMR) data sourced from i) NHS Digital for participants recruited in England; ii) NHS Wales Informatics Service for

For peer review only - http://bmjopen.bmj.com/site/about/guidelines.xhtml

participants recruited in Wales; and iii) the electronic Data Research and Innovation Service for participants recruited in Scotland.

- Data will be requested from the above listed NHS organisations pertaining to outpatient, inpatient, critical care and A&E attendances from 3 months prior to randomisation, to 4 weeks post randomisation (following completion of follow-up). Participant information (postcode, date of birth, NHS (or CHI) number and screening/randomisation number) will be securely transferred to authorised personnel at the NHS organisations and the HES and SMR data with the screening/randomisation number will be securely transferred to health economists at Centre for Health Economics and Medicines Evaluation (CHEME), Bangor University, who will be conducting the economic analysis.
- The only identifier present in the HES / SMR dataset will be the trial randomisation identifier, and health economists at CHEME will not have any access to keys linking this to participant personal data. Electronic HES and SMR records will be stored on secure Bangor University computer servers which meet NHS data security standards. Access will be restricted only to health economists working on the trial.

Unit costs will be obtained from NHS reference costs and other standard NHS sources.

7.4.2.2 Health outcomes

Quality Adjusted Life Years (QALYs) will be estimated from utilities derived from trial participant responses to EQ-5D-5L questionnaires (and applying recommended methods for generating UK relevant utilities) administered for completion at 24 hours and at 4-weeks post randomisation.

7.5 Patient Transfer and Withdrawal

7.5.1 Discontinuation

Upon completion of trial intervention, subsequent treatments and actions will be recorded (see section 9.3.2). This would **not** necessitate the participant discontinuing the study.

7.5.2 Withdrawal of Consent

Generally, follow-up will continue unless the woman/Legal Representative explicitly withdraws consent for follow-up.

The woman/Legal Representative is free to withdraw consent at any time without providing a reason and without being subject to any resulting detriment. The rights and welfare of the woman will be protected by emphasising to them throughout the trial that the quality of medical care will not be adversely affected if they decline to participate in the trial, or withdraw consent.

Where the woman/Legal Representative wishes to withdraw consent, a withdrawal CRF should be completed documenting the reason for withdrawal. Centres should explain the importance of remaining in trial follow-up and of the importance of contributing data. Data collected prior to withdrawal will be analysed and data relevant to safety events will continue to be collected to meet regulatory requirements.

7.5.3 Participant Transfers

A minority of women may be randomised and treated at a COPE site and then transferred to another hospital for further treatment before the 48-hour follow-up period is completed (e.g. the participant needs to be transferred to a hospital with intensive care facilities).

For peer review only - http://bmjopen.bmj.com/site/about/guidelines.xhtml

In these cases, the treating hospital should contact the transfer hospital to collect the follow-up information and consent (where possible).

Note: Only transfer sites that are also a recruiting site for COPE can assist with the consent process, however, regardless of where consent was sought, the participant would be classified as a recruited participant for the treating site only.

7.6 Loss to Follow-up

Follow-up contact will be made via the PI or designated research staff at each centre. After discharge, participants will be contacted via their preferred means of communication for the purposes of the 4-week follow-up.

Non-responders will be contacted again after 2 weeks. Failure to respond to the second contact within another 2 weeks will deem the participant “loss to follow-up”.

Wherever possible, information on the reason for loss to follow-up will be recorded.

7.7 Trial Closure

The end of the trial is defined to be the date on which data for all participants is locked and data entry privileges are withdrawn from the trial database. However, the trial may be closed prematurely by the Trial Steering Committee (TSC), on the recommendation of the Independent Data and Safety Monitoring Committee (IDSMC).

For peer review only

8 EMBEDDED MIXED METHODS RESEARCH

8.1 Design

Recruitment to the embedded mixed methods research has now closed.

For at least the first 9 months of recruitment, we will use interviews, and questionnaires to explore the views and experiences of women recruited to the trial and their birth partners. In the five pilot sites (or alternative site if a pilot site is not open to recruitment within two months of recruitment of the first woman to the trial) we will also conduct focus groups with practitioners and audio-recorded recruitment discussions. This embedded research will explore:

- experience and acceptability of the trial and its interventions;
- antenatal and postnatal information provision;
- practitioner experience of recruitment;
- the interaction and exchange of information about the trial between staff and potential participants;
- emergency recruitment at time of PPH and postnatal consent;
- antenatal consent amongst both those recruited and not recruited;
- postnatal consent for the disclosure of identifiable data and follow-up.

Interim findings from research conducted in the internal pilot will be used to inform approaches to recruitment and consent procedures for the remainder of the COPE trial.

Methods will include:

A: Audio recording of COPE antenatal and postnatal consent discussions between women and their birth partner (if applicable) and trial recruiters (only at the internal pilot sites).

B. A questionnaire with women and their birth partners (if applicable) who consent or decline consent for the COPE trial (all sites).

C: Interviews with women and their birth partners (if applicable) who consent or decline consent for the COPE trial, including bereaved parents (all sites)

D: Focus groups and/or interviews with COPE trial recruiters at each pilot site (only at the internal pilot sites)

8.2 Selection of participants

__**Women and birth partners**__

Inclusion criteria

- Women and their birth partners who are recruited to COPE

Exclusion criteria

- Women and their birth partners who do not speak English or lack the capacity to consent

For peer review only - http://bmjopen.bmj.com/site/about/guidelines.xhtml

COPE practitioners

Inclusion criteria

- Practitioners who are involved in screening, recruiting, randomising and consenting participants/ legal representatives

Exclusion criteria

- None

Consent sought from	Location consent was sought	A: Audio-recording	B: Questionnaire	C: Interview
Woman and birth partner (if present) who do and do not give consent for COPE trial	On-site (antenatal and postnatal following birth)	✔ (at pilot sites only)	✔	✔
Bereaved woman and birth partner (if applicable)	On-site (postnatal)			✔
Woman and birth partner (if applicable)	Home (postnatal)		✔	✔
Bereaved women and birth partner (if applicable)	Home (postnatal)			✔

8.3 Enrolment and procedure

8.3.1 Part A: Audio-recordings

Delegated COPE recruiters at pilot sites will seek permission to audio-record recruitment consultations when they first approach potential women and their birth partners about COPE in postnatal settings. If permission for audio-recording is declined the recruitment consultation will not be recorded. If permission is given, the recruiter will record this on the relevant COPE information sheet and consent form and activate an audio-recorder. If there is more than one trial discussion (e.g. an initial discussion followed by a full trial discussion after woman has considered the trial information) then all discussions should be recorded.

Completed audio-recordings will be uploaded to a secure online file store for professional transcription purposes. The 'Guidance for the management of audio recordings' should be used to ensure that the recordings are correctly stored and uploaded in accordance with the Data Protection Act.

Audio-recording of trial consultations will take place at each of the five pilot sites (or alternative site if a pilot site is not open to recruitment within two months of recruitment of the first woman to the trial) until the sample target (data saturation point) is achieved. Sites will be informed when audio-recordings can stop.

Note: Please refer to section 8.2 for audio recordings applicability.

For peer review only - http://bmjopen.bmj.com/site/about/guidelines.xhtml

8.3.2 Part B: Questionnaires

At postnatal recruitment discussions COPE recruiters will ask all women and their birth partners (including those who decline consent for the COPE trial, but not including bereaved women and their birth partners) to complete the COPE recruitment questionnaire, place it in a sealed stamped addressed envelope and return it to the recruiter to post to qualitative researcher.

If consent has been given to complete a questionnaire within the COPE information sheet and consent form, but a questionnaire has not been received, the questionnaire will be sent via post. In the rare instance that COPE trial consent is not sought prior to discharge/transfer to another hospital, the site team should send the questionnaire by post to women and their birth partners along with the COPE information sheet and consent form to complete. If questionnaires are not returned the embedded study team will ask women and birth partners to complete the questionnaire online at the point of arranging interviews (see below).

Recruitment for questionnaires will be conducted for the internal pilot and for at least the first 9 months of recruitment. We anticipate to collect approximately 500 questionnaires.

Note: Refer to section 8.2 for questionnaire applicability.

8.3.3 Part C: Interviews with women and their birth partner

COPE recruiters will ask women and their birth partners if they wish to take part in a telephone interview or face-to-face interview (if they live in the Northwest of England). Their permission to be contacted to arrange an interview will be recorded on the relevant COPE information sheet and consent form. They will be contacted using their contact details provided. We aim to interview a selection of women and their birth partners, who decline consent and provide consent.

Following consent to do so, the University of Liverpool qualitative researchers will make contact with women and, their birth partner (if applicable) after the birth to arrange remote interviews (telephone or online) or face-to-face interviews (Northwest only) at their home or location of choice (e.g. hospital, University) approximately 2 to 4 weeks after they have given birth. Telephone interviews with women and their birth partners will be conducted separately. Those involved in face-to-face interviews can choose to be interviewed together. Bereaved women will be contacted no less than six weeks after birth. Such calls/interviews will be handled with sensitivity and will be arranged within a timeframe tailored to individual women.

All interviews will be conducted by qualitative researchers who have proven skills in the conduct of research in sensitive settings. Any distress during the interviews will be managed with care and compassion and participants will be free to decline to answer any questions that they do not wish to answer or to stop the interviews at any point. Such participants will be supported in obtaining appropriate help.

Interviews will be conducted until point of sample or data saturation (approximately 15-35), whereby no new themes are emerging in the analysis of data). It is expected that the interviews will take on average between 30 to 60 minutes.

After the interviews are complete, qualitative researchers will send participants a letter and a £30 high street shopping voucher to thank them for their time. All participants who express an

For peer review only - http://bmjopen.bmj.com/site/about/guidelines.xhtml

interest in taking part but are not selected for an interview will be contacted via email by a qualitative researcher (if address provided) to thank them for their interest in the study.

8.3.4 Part D: Focus groups and/or interviews with the COPE practitioners

COPE practitioners (e.g. recruiters, PI or lead RM) at the five pilot sites (or alternative site if a pilot site is not open to recruitment within two months of recruitment of the first woman to the trial) will be sent an email invitation to participate in an online focus group or remote interview (telephone or online via Zoom or Microsoft Teams) (whichever is possible to arrange around clinical practice). Interviews and focus groups will take place between 3 and five months after site opening (depending upon sufficient experience of recruitment and staff availability)

Those who register interest will be sent a practitioner information sheet including details of topics that will be discussed in the focus group or telephone interview including:

- acceptability of the trial;
- experience of recruitment and the approach to consent;
- potential barriers to recruitment and any potential solutions;
- how information about the trial is communicated to women;
- how women respond to trial information and any suggested improvements to trial information and consent processes.

All interviews will be conducted by the qualitative researcher:

Consent will be sought from participants before the focus group or telephone interview begins. This will involve the qualitative researcher explaining the aims of the study to the practitioner and providing an opportunity for questions. For focus groups, PIS and consent forms will be sent to practitioners via email. Practitioners will be asked to complete and return the consent form to the embedded study researcher before the scheduled focus group. For telephone or online interviews, the qualitative researcher will read each aspect of the consent form, including consent for audio recording of the interview and ask whether the participant would like to receive a copy of the findings when the study is complete. The qualitative researcher will tick each box on the consent form when the participant provides verbal consent. Informed consent discussions will be audio recorded for analysis.

Participation will be entirely voluntary and practitioners will be able to withdraw at any time without giving a reason.

After the interviews are complete, site research staff will be sent a thank you letter.

For peer review only

9 TRIAL TREATMENTS

9.1 Introduction

This is a randomised, double-blind, double-dummy trial. The investigational medicinal products (IMPs) in this trial are carboprost and oxytocin. Participants will be randomised to one of the following treatment arms:

Intervention: Carboprost 250 micrograms by deep intramuscular injection and placebo by slow intravenous injection over 2 minutes.

Control: Oxytocin (5 international units (IU) for caesarean section or 10 IU for vaginal delivery) by slow intravenous injection over 2 minutes and placebo by deep intramuscular injection.

9.2 Formulation, Packaging, Labelling, Storage and Stability

9.2.1 Formulation

COPE trial active treatments (carboprost and oxytocin) are market-authorised drugs. The formulations will be supplied in their marketed form and will not be manipulated other than labelling and packaging to maintain double-blind, carried out by clinical supply company.

9.2.1.1 Carboprost

Active carboprost 250 micrograms (Pfizer Limited) is presented as 1 ml of a colourless, sterile, aqueous solution for intramuscular injection.

9.2.1.2 Oxytocin

Active oxytocin 10 international units (Hameln Pharmaceuticals Ltd) is presented as 1 ml of a colourless, sterile, aqueous solution for intravenous injection.

9.2.1.3 Placebo

The matching placebo is presented as 0.9% Sodium Chloride in 1 ml colourless, sterile, aqueous solution for intramuscular injection or intravenous injection.

9.2.2 Packaging and Labelling

IMP supplies will be secured via a clinical services company, who will procure, over-label and package into blinded treatment kits in accordance with regulation 46 SI 2004/1031 and the detailed guidance provided in annex 13 of the EU Good Manufacturing Practice (GMP) guide. Kits will be sequentially numbered and their number will become the participant's randomisation number.

Each kit will contain two ampoules in an outer carton. Both ampoules and the outer carton will be labelled. Each ampoule is intended for a single dose for a single participant.

For peer review only - http://bmjopen.bmj.com/site/about/guidelines.xhtml

9.2.3 Storage and Stability

Treatment kits will be temperature monitored whilst stored at the central manufacturing facility and during shipment to participating centres. Treatment kits will be received and stored by the site pharmacy prior to being distributed in batches to the participating department where they will be stored until randomisation in an appropriate, secure refrigerator (randomisation area). Randomisation represents the point at which a kit is removed from the fridge, following confirmation of woman's eligibility. The fridge temperature should be maintained at 2-8°C. Kits should not be frozen and ampoules should be kept refrigerated and in their outer carton until required.

The temperature of storage locations will be monitored and documented daily in the temperature log. In the event of storage temperatures falling outside the 2-8°C range permitted, stock should be immediately quarantined in a monitored refrigerator and the trial coordinator at the LCTC contacted for further actions. Stock should not be removed from quarantine until instructed to do so by the trial co-ordinator. Stock that falls below 2°C should be quarantined and should not be used in the trial; the trial co-ordinator will confirm when this stock can be destroyed. Some stock that goes above 8°C may be used again with a shortened expiry date (12 weeks from excursion). This stock must remain in quarantine until the trial co-ordinator has confirmed that it can be used again. If the trial co-ordinator confirms that this can be used again then the site must apply a new label to the stock with the new expiry date and batch number (labels will be provided by the LCTC). Full guidance on re-labelling activities can be found in COPE guidance for re-labelling. Re-labelling is to be performed under GMP conditions with exemption 37 for labelling. Two people at site (one of whom should be a pharmacist) will complete re-labelling following the guidance document and record on the COPE re-labelling log when completed. Once re-labelling has been completed the trial co-ordinator will confirm when the stock can be removed from quarantine.

9.3 Distribution, Dispensing, Dosage and Administration of Study Treatment/s

9.3.1 Distribution and Dispensing

IMPs will be supplied as bulk stock from the distributor to pharmacy in kits containing two ampoules. Authorised, trial-trained pharmacy staff should distribute sufficient kits to the designated randomisation area(s) where they should be stored in accordance with section 9.2. Pharmacy staff at site will sign and date an accountability log documenting receipt of IMP and recording the distributed location.

Kits should be dispensed at time of randomisation following confirmation of eligibility. Dispense represents the point at which kits are removed from the randomisation area fridge. Staff at site will sign and date accountability logs documenting receipt in randomisation area and dispense for randomisation.

9.3.2 Dosage and Administration

Study interventions are supplied as ready-for-use, 1 millilitre formulations as single doses for a single participant. Both ampoules in each kit should be administered following randomisation in accordance with COPE protocol and local practice (this includes option to dilute intravenous ampoule contents in 10 millilitres saline to facilitate slow injection):

- Carboprost 250 micrograms by deep intramuscular injection (Pfizer Limited; Hemabate Sterile Solution) and placebo by slow intravenous injection over 2 minutes

For peer review only - http://bmjopen.bmj.com/site/about/guidelines.xhtml

OR

- Oxytocin (5 IU for caesarean section or 10 IU for vaginal delivery) by slow intravenous injection over 2 minutes (Hameln Pharmaceuticals Ltd; Oxytocin 10 IU/ml Concentrate for Solution for Infusion) and placebo by deep intramuscular injection.

Note: To achieve 5 IU oxytocin (caesarean section) draw up 0.5 ml of intravenous ampoule contents and discard the remaining 0.5 ml.

As far as packaging allows, **both** ampoules should be visually inspected for particulate matter and discoloration prior to administration. Should this be observed, the whole kit (two ampoules) should be quarantined, segregated from the other IMP and pharmacy notified. If neither ampoule has been administered the next sequential kit may be selected to administer COPE treatment. If one ampoule has already been administered and a problem is noted with the second ampoule a further COPE kit **should not** be selected. Care should continue as if both ampoules had been administered, with the use of the emergency procedures and / or unblinding as required. Both ampoules should be quarantined, segregated from the other IMP and pharmacy notified.

The quarantined kit/s should be returned to pharmacy as soon as they are next open to await further instruction (pharmacy staff will liaise with LCTC).

Once the IMP is administered, treating clinicians should **not** use further carboprost or oxytocin bolus within 15 minutes, this time represents the half-life of intravenous oxytocin, and is the time interval specified for repeat doses of carboprost. Additional pharmacological treatments, if needed, should be avoided within the 15 minutes. However, if a woman continues to experience heavy bleeding, the managing clinician may use the below treatments during this period:

PHYSICAL

- Empty the bladder, massage the uterus and check for genital tract trauma;
- Put the baby to the breast to stimulate natural oxytocin release;
- Use bimanual compression, or aortic compression.

DRUGS

- Give ergometrine (if not hypertensive);
- Start an oxytocin infusion;
- Administer intravenous tranexamic acid 1g; or
- Give misoprostol 800 micrograms sublingual or per rectum.

After the 15 minute period, further management should be conducted in accordance with NICE guidance and local practice for the management of PPH, including the use of further oxytocin (no more than 10 additional units applied as a bolus) or carboprost (no more than 7 additional doses).

Both of the ampoules in a kit should be administered so that all women are administered a deep intramuscular injection and a slow intravenous injection. For women who are recruited following caesarean section, 0.5 ml of intravenous ampoule contents will be drawn up for injection and the remaining 0.5 ml discarded. This will achieve the 5 IU oxytocin. For women recruited following vaginal birth, the entirety of the 1 ml of intravenous ampoule contents will be drawn up for injection.

For peer review only - http://bmjopen.bmj.com/site/about/guidelines.xhtml

Given the nature of the interventions in this protocol it is not anticipated a further modification in dose will be required or feasible. If, as a last resort, unblinding is necessary, this will be conducted in accordance with section 9.7.

9.4 Overdose

As part of the COPE protocol, women will be administered a single dose of carboprost or oxytocin. If administered in accordance with this protocol, overdose is unlikely. In the event that overdose is suspected, treatment should be symptomatic and supportive and there is the option to unblind if absolutely essential for further management (see also Section 9.7).

Although overdose of medication without signs or symptoms may be excluded from AE reporting this may still require investigation to ensure the protocol and regulatory requirements are met e.g. for IMP management and administration to ensure participant safety. Therefore, in such circumstances site should report immediately (within 24 hours) using the SAE form to LCTC (see section 10) and a root cause investigation should be initiated.

9.5 Delivery and Accountability of IMP at Trial Sites

9.5.1 Delivery

Trial IMP will only be delivered to an investigator site once the site has been initiated by LCTC, acting on behalf of Sponsor to ensure full ethical and regulatory approvals have been granted. The size of the shipments to each site will be pre-determined based on the participant recruitment target for that individual site.

Recruitment will be monitored centrally and drug shipment dates will be tailored accordingly to ensure that pharmacies hold adequate supplies of trial treatment. Pharmacies must document all shipment receipts and will provide copies of this documentation to the LCTC.

IMP stock must be received by a designated member of the pharmacy department and must be stored at 2-8°C with temperature monitoring as described in section 9.2.3.

9.5.2 Accountability Procedures for Study Treatment/s

Drug accountability logs will be maintained throughout (accountability and temperature monitoring records will be maintained in the receiving department within site as well as within pharmacy).

Records of all shipments must be kept in the drug accountability log.

If IMP stock received from the distributor is unexpected, wrong, damaged or out of temperature range, the stock should be quarantined and LCTC contacted for further actions.

9.5.3 Expired and unused IMP stock

Any stock that has expired at the trial site during the trial and any stock remaining at trial closedown must be notified to the LCTC, who will authorise destruction. Stock will be destroyed locally according to site policy and records made in the applicable accountability log.

For peer review only - http://bmjopen.bmj.com/site/about/guidelines.xhtml

9.6 Concomitant Medications/Treatments

9.6.1 Medications Permitted/Not Permitted/ Precautions Required

Once the IMP is administered, treating clinicians should **not** use further carboprost or oxytocin bolus within 15 minutes, this time represents the half-life of intravenous oxytocin, and is the time interval specified for repeat doses of carboprost. Additional pharmacological treatments, if needed, should be avoided within the 15 minutes. However, if a woman continues to experience heavy bleeding, the managing clinician may use other treatments (see section 9.3.2). After the 15 minute period, further management should be conducted in accordance with NICE guidance and local practice for the management of PPH, including the use of further oxytocin (no more than 10 additional units applied as a bolus) or carboprost (no more than 7 additional doses). Subsequent treatments and actions will be recorded.

9.6.2 Data on Concomitant Medication

Data on concomitant medications will not be recorded for drugs other than those meeting the criteria below. The drug, reason for use and route of administration will be recorded for the below:

- Concomitant medication implicated in an interaction with trial treatment and resulting in a Serious Adverse Event (SAE) or Suspected Unexpected Serious Adverse Reaction (SUSAR);
- Additional uterotonic use before and after the IMP;
- Blood products (with units given);
- PPH prophylactics given;
- Intrapartum oxytocin infusion used

9.7 Unblinding

Unblinding pressure-sealed envelopes are to be provided to each site. They are to be stored in a secure location accessible to trial staff, for example in a controlled drug cabinet. These are to be used to unblind the participant during the emergency, where knowledge of COPE treatment is absolutely essential for further management.

If unblinding is considered appropriate, the unblinding envelope with the randomisation number matching the participant's randomisation number should be accessed ideally after discussing with the original recruiter or if not available with a delegated member of the research team, ideally a recruiter (prescriber). Following on, LCTC should be informed initially by telephone, and then by completion and return of an unblinding CRF.

For peer review only

For peer review only - http://bmjopen.bmj.com/site/about/guidelines.xhtml

10 SAFETY REPORTING

10.1 Time Period for Safety Reporting

Safety reporting of Serious Adverse Events and Serious Adverse Reactions will be actively monitored and reported during the clinical trial from the time of randomisation until hospital discharge or 4 weeks, whichever is sooner.

IMPORTANT: Any safety events related to the trial treatment (see section 10.7) occurring after the end of this "active monitoring" period which meet the definition of serious (see section 10.4), related (see section 10.7) and are recorded for this study (see section 10.10) must continue to be reported by sites to the LCTC in accordance with the timeframes and procedures described in section 10.10. The same processes established for SAEs within the active monitoring period should be followed for these events.

Non-serious adverse reactions of particular interest (listed below) will be collected on a separate CRF.

Adverse reactions of particular interest:

1. Hypotension - A fall in blood pressure requiring treatment, or maternal symptoms of hypotension, developed within 2 minutes of IMP administration.

Those listed below, occurring within 2 hrs of IMP administration:

2. Vomiting
3. Pyrexia; temperature of >38°C
4. Headache
5. Hot flushes
6. Diarrhoea

Non-serious adverse events and non-serious adverse reactions (not listed within reactions of particular interest) do not require reporting.

10.2 Reference Safety Information

The Reference Safety Information (RSI) in COPE used to assess expectedness is section 4.8 of the respective Summary of Product Characteristics (SmPC):

- Oxytocin - Oxytocin 10IU/ml Concentrate for Solution for Infusion, Hameln Pharmaceuticals Ltd
- Carboprost - Hemabate Sterile Solution, Pfizer Limited

As the placebo is inert there are no expected adverse reactions, therefore any serious adverse reactions thought to be related to the placebo would be unexpected and reported as a potential SUSAR.

For peer review only

10.3 Flowchart of Adverse Events Reporting Requirements

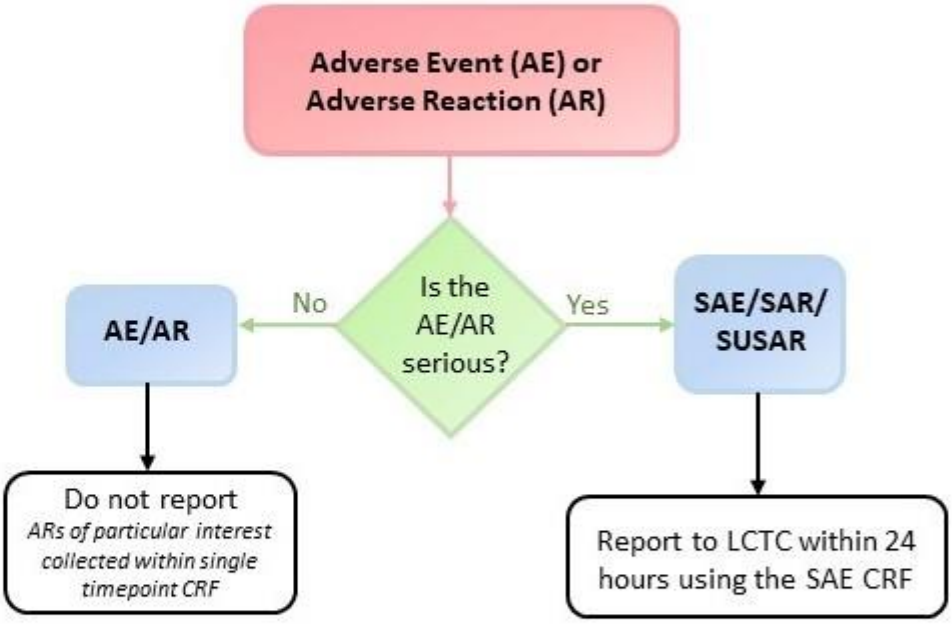

10.4 Terms and Definitions

**Adverse Event (AE)**	Any untoward medical occurrence in a subject to whom a medicinal product has been administered, including occurrences which are not necessarily caused by or related to that product.<br><br>Therefore an AE is any unfavourable or unintended change in the structure (signs), function (symptoms) or chemistry (laboratory data) in a subject to whom an IMP has been administered, including occurrences which are not necessarily caused by or related to that product.
**Adverse Reaction (AR)**	Any untoward and unintended response in a subject to an IMP which is related to any dose administered to that subject.<br><br>Therefore an AR is any unfavourable or unintended change in the structure (signs), function (symptoms) or chemistry (laboratory data) in a subject to an IMP which is related to any dose administered to that subject.

For peer review only - http://bmjopen.bmj.com/site/about/guidelines.xhtml

**Unexpected Adverse Reaction (UAR)**	An adverse reaction, the nature and severity of which is not consistent with the information about the medicinal product in question set out in the investigator's brochure or the SPC, which may be referenced where the IMP in question is a product with a marketing authorisation.
**Serious Adverse Event (SAE), Serious Adverse Reaction, or Unexpected Serious Adverse Reaction**	An adverse event, adverse reaction or unexpected adverse reaction respectively that:<br>• results in death;<br>• is life threatening (places the subject, in the view of the investigator, at immediate risk of death from the experience as it occurred (this does not include an adverse experience that, had it occurred in a more severe form, might have caused death);<br>• requires hospitalisation or prolongation of existing hospitalisation (hospitalisation is defined as an inpatient admission, regardless of length of stay, even if the hospitalisation is a precautionary measure for continued observation; hospitalisations for a pre-existing condition, including elective procedures that have not worsened, do not constitute a SAE).<br>• results in persistent or significant disability or incapacity (substantial disruption of one's ability to conduct normal life functions);<br>• consists of a congenital anomaly or birth defect;<br>• other important medical events (these may not result in death, be life-threatening, or require hospitalisation, but may be considered a serious adverse event/experience when, based upon appropriate medical judgment, they may jeopardise the subject and may require medical or surgical intervention to prevent one of the outcomes listed in this definition).
**Suspected Serious Adverse Reaction (SSAR)**	An adverse reaction that is classed in nature as serious and which is consistent with the information about the medicinal product in question set out in the case of a licensed product, in the SPC for that product.

**Suspected Unexpected Serious Adverse Reaction (SUSAR)**	An adverse reaction that is classed in nature as serious and which is not consistent with the information about the medicinal product in question set out in the Reference Safety Information.

N.B. "seriousness" assessment must be performed by an appropriately delegated, medically qualified member of the site research team.

10.5 Notification of deaths

All deaths in participants from the time of randomisation until hospital discharge or 4 weeks, whichever is earlier, should be reported to the LCTC using the SAE CRF **within 24 hours** of the clinical research team becoming aware of the event.

Death should be considered an outcome and not a distinct event. The event or condition that caused or contributed to the fatal outcome should be recorded as the single medical concept on the SAE CRF. Generally, only one such event should be reported. The term "sudden death" should only be used for the occurrence of an abrupt and unexpected death due to presumed cardiac causes in a participant with or without pre-existing heart disease, within 1 hour of the onset of acute symptoms or, in the case of an unwitnessed death, within 24 hours after the participant was last seen alive and stable. If the cause of death is unknown and cannot be ascertained at the time of reporting, "unexplained death" should be recorded on the SAE CRF. If the cause of death subsequently becomes available (e.g., after autopsy), "unexplained death" should be replaced by the established cause of death.

10.6 Severity / Grading of Adverse Events

The assignment of the severity/grading should be made by the investigator responsible for the care of the woman using the definitions below.

Mild	Does not interfere with routine activities
Moderate	Interferes with routine activities
Severe	Impossible to perform routine activities

A distinction is drawn between serious and severe events. Severity is a measure of intensity (see above) whereas seriousness is defined using the criteria in section 10.4, hence, a severe AR need not necessarily be a Serious Adverse Reaction.

10.7 Relationship to Trial Treatment

An assessment of the causality will be made by the investigator responsible for the care of the participant using the definitions below.

If any doubt about the causality exists, the local investigator should inform the LCTC who will notify the Chief Investigator. In the case of discrepant views on causality between the investigator and others, the opinion of the treating investigator will never be downgraded and the Medicines for Healthcare products Regulatory Agency (MHRA) will be informed of both points of view.

**Unrelated**	There is no evidence of any causal relationship. N.B. An alternative cause for the AE should be given.

For peer review only - http://bmjopen.bmj.com/site/about/guidelines.xhtml

**Unlikely**	There is little evidence to suggest there is a causal relationship (e.g. the event did not occur within a reasonable time after administration of the trial medication). There is another reasonable explanation for the event (e.g. the participant's clinical condition, other concomitant treatment).
**Possibly**	There is some evidence to suggest a causal relationship (e.g. because the event occurs within a reasonable time after administration of the trial medication). However, the influence of other factors may have contributed to the event (e.g. the participant's clinical condition, other concomitant treatments).
**Probably**	There is evidence to suggest a causal relationship and the influence of other factors is unlikely.
**Almost certainly**	There is clear evidence to suggest a causal relationship and other possible contributing factors can be ruled out.

An AE whose causal relationship to the study drug is assessed by the investigator as "possible", "probable", or "almost certainly" is an Adverse Reaction (AR).

10.8 Expectedness

There is no requirement for a reporting investigator to make an assessment of expectedness. For all SARs, the CI (or delegate) will assess for expectedness, determined by the COPE RSI (see section 10.2 for RSI). The nature, severity, or frequency of the event will be considered – if this is not consistent with that described for the type of event in the RSI current and approved at the time of the event's onset, the event will be assessed as unexpected. If **unexpected** the SAR will be reported as a SUSAR.

10.9 Follow-up After Adverse Events

All reportable adverse events should be followed until satisfactory resolution or until the investigator responsible for the care of the participant deems the event to be chronic or the participant to be stable.

When reporting SAEs, SARs or SUSARs the investigator responsible for the care of the woman should apply the following criteria to provide information relating to event outcomes: resolved; resolved with sequelae (specifying with additional narrative); not resolved/ongoing; ongoing at final follow-up; fatal or unknown.

10.10 Reporting Procedures

All SAEs and adverse reactions of particular interest should be reported for the durations described in section 10.1. Depending on the nature of the event the reporting procedures below should be followed. Any questions concerning adverse event reporting should be directed to the LCTC in the first instance. A flowchart is provided (see section 10.3) to aid in determining reporting requirements.

10.10.1 Non serious AEs

These events are not reportable for the purposes of COPE pharmacovigilance.

For peer review only - http://bmjopen.bmj.com/site/about/guidelines.xhtml

10.10.2 Non serious ARs

Only ARs of particular interest (see section 10.1), whether expected or not, should be recorded on the appropriate CRF, which should be transmitted to the LCTC within 21 days of the form being completed.

10.10.3 SAEs/ SARs/ SUSARs

All SAEs, SARs and SUSARs should be reported within 24 hours of the local site becoming aware of the event. The SAE CRF asks for the nature of event, date of onset, severity, corrective therapies given, outcome and causality. Additional information should be sent within 5 days if the reaction has not resolved at the time of reporting.

Local investigators should report any SUSARs and/or SAEs as required locally.

All deaths and overdoses should be reported as described in sections 10.5 and 9.4, respectively.

Minimum information required for reporting (CT-3)

- Valid EudraCT number (if applicable)
- Sponsor study number
- One identifiable coded subject
- One identifiable reporter
- One SUSAR
- One suspect IMP (including active substance name- code)
- A causality assessment

i. The SAE form should be completed by a designated investigator, a physician named on the 'delegation of authority and signature log' as responsible for safety reporting and making trial related medical decisions. The investigator should assess the SAE for the likelihood that it is a response to an IMP. In the absence of the designated investigator the SAE form should be completed and signed by an alternative member of the research site trial team and submitted to the LCTC. As soon as possible thereafter the responsible investigator should check the SAE form, make amendments as appropriate, sign and re-send to the LCTC. The initial report shall be followed by detailed reports as appropriate.

ii. When submitting an SAE form to the LCTC, research sites should also telephone the appropriate trial co-ordinator/data manager on telephone number **0151 795 8760** to advise that a SAE form has been submitted. The LCTC trial team should ensure that the number to be used to report SAEs in this way is manned during office hours, and is notified to research site personnel during the site initiation process.

iii. The SAE CRF should be sent by **secure** email (within 24 hours) to the LCTC:

Email: lctcsafe@liverpool.ac.uk

iv. The responsible investigator must **notify** their R&D department of the event (as per standard local governance procedures).

v. In the case of an SAE the participant must be followed-up until clinical recovery is complete and laboratory results have returned to normal, or until the event has stabilised. Follow-up may continue after completion of protocol treatment, if necessary. A final report will always be expected.

For peer review only - http://bmjopen.bmj.com/site/about/guidelines.xhtml

vi. Follow-up information is noted on another SAE form by ticking the box marked 'follow-up' and **securely** emailing to the LCTC as information becomes available. Extra, annotated information and/or copies of test results may be provided separately.

vii. The participant **must** be identified by trial number only. The participant's name **must not** be used on any correspondence.

Patient safety incidents that take place in the course of research should be reported locally by each participating NHS Trust in accordance with Trust reporting procedures.

10.10.4 Maintenance of Blinding

Systems for SUSAR and SAE reporting should, as far as possible, maintain blinding of individual clinicians and of trials staff involved in the day-to-day running of the trial. Unblinding of clinicians may be unavoidable if the information is necessary for the medical management of particular participant. The safety of participant in the trial always takes priority. In each report, seriousness, causality and expectedness should be evaluated for all of the trial treatments unless criteria have been fulfilled and unblinding has taken place (section 9.7). Cases that are considered serious, unexpected and possibly, probably or almost certainly related to one of the trial therapies (i.e. possible SUSARs) would have to be unblinded at LCTC prior to reporting to the MHRA.

10.11 Responsibilities – LCTC

The LCTC is undertaking duties delegated by the trial Sponsor, the University of Liverpool, and is responsible for the reporting of SAEs, SARs and SUSARs to the regulatory authorities (MHRA and REC) as follows:

- SUSARs which are fatal or life-threatening must be reported not later than 7 days after the LCTC is first aware of the reaction. Any additional relevant information must be reported within a further 8 days.
- SUSARs that are not fatal or life-threatening must be reported within 15 days of the LCTC first becoming aware of the reaction.
- A list of all SARs (expected and unexpected) and SAEs must be reported annually.

It is recommended that the following safety issues should also be reported in an expedited fashion:

- An increase in the rate of occurrence or a qualitative change of an expected serious adverse reaction, which is judged to be clinically important.
- Post-study SUSARs that occur after the patient has completed a clinical trial and are notified by the investigator to the LCTC/Sponsor.
- New events related to the conduct of the trial or the development of the IMPs and likely to affect the safety of the participants, such as:
    a. A SAR which could be associated with the trial procedures and which could modify the conduct of the trial;
    b. A significant hazard to the participant population, such as lack of efficacy of an IMP used for the treatment of a life-threatening disease;
    c. A major safety finding from a newly completed animal study (such as carcinogenicity).
    d. Any anticipated end or temporary halt of a trial for safety reasons and conducted with the same IMP in another country by the same sponsor;

For peer review only - http://bmjopen.bmj.com/site/about/guidelines.xhtml

- Recommendations of the IDSMC, if any, where relevant for the safety of the participants.

Staff at the LCTC will liaise with the Chief Investigator (or designated other specified in the protocol) who will review all SAEs received for seriousness and causality, and assess expectedness. Investigator reports of SAEs will be reviewed immediately and those that are SUSARs identified and reported to MHRA and REC. The causality assessment given by the Local Investigator at the hospital cannot be downgraded and in the case of disagreement, both opinions will be provided with the report.

The PIs at all institutions participating in the trial will be notified of any SUSARs.

10.12 Safety reports

Safety reports will be generated during the course of the trial which allows for monitoring of SAE and ARs of particular interest reporting rates across sites. The LCTC will send annual developmental safety update reports (DSUR) to MHRA and REC. Any concerns raised by the IDSMC or inconsistencies noted at a given site may prompt additional training at sites, with the potential for the LCTC to carry out site visits if there is suspicion of unreported SAEs/ ARs of particular interest in patient case notes. Additional training will also be provided if unacceptable delay in safety reporting timelines. If any safety reports identify issues that have implications for the safety of trial participants, the PIs at all institutions participating in the trial will be notified.

10.13 Urgent Safety Measures

An urgent safety measure (USM) is a procedure not defined by the protocol, which is put in place prior to authorisation by the MHRA and REC in order to protect clinical trial participants from any immediate hazard to their health and safety.

The LCTC will notify the MHRA and REC immediately and, in any event, within 3 days that such a measure has been taken and the reasons why it has been taken. The initial notification to the MHRA will be by telephone (ideally within 24 hours) and a notice in writing will be sent within 3 days, setting out the reasons for the USM and the plan for further action. After discussion with the MHRA and REC, further action will be agreed, which may include submission of a substantial amendment, a temporary halt, or permanent termination of the trial.

If the study is temporarily halted it may not recommence until authorised to do so by the MHRA and REC. If the study is permanently terminated before the date specified for its conclusion (in the original applications to MHRA and REC), the sponsor should notify the MHRA and REC within 15 days of the date of termination by submitting the formal End of Trial Notification.

10.14 Out-of-hours Medical Cover

Out-of-hours medical cover will use the standard out-of-hours care available within the hospital.

For peer review only - http://bmjopen.bmj.com/site/about/guidelines.xhtml

11 STATISTICAL CONSIDERATIONS

11.1 Introduction

A full and detailed statistical analysis plan (SAP), following ICH E9 and the CONSORT guidelines, will be developed prior to the first comparative monitoring report to be presented to the IDSMC (22). The main features of these planned statistical analyses are detailed below.

11.2 Method of Randomisation

The randomisation list will be generated by a statistician at the LCTC (independent to the COPE trial). Sequentially numbered treatment kits, detailing the randomisation number, will be stored in a secure location in the delivery areas. Upon randomisation the research team will select the next sequentially numbered kit for the particular mode of birth.

Participants will be randomised to carboprost or oxytocin in a 1:1 ratio.

11.3 Sample Size calculation

To detect a 2.3% reduction in a 5.8% transfusion rate (relative risk 0.60) using a Fishers exact test with 90% power (alpha 0.05), we would require 1,880 participants per group, increasing to 3,948 allowing for 5% loss to follow-up.

The magnitude of treatment effect for the primary outcome is needed in order to change long established clinical practice, and to represent a significant advantage, given the carboprost cost and side effects.

Data from sites and published literature provide a range of transfusion rates, the variation of which may be explained by the time point of randomisation. The table below demonstrates the impact of varying the event rate within this range on the study power, all other parameters including the relative effect size are maintained. Therefore, even if the transfusion rate was the lowest observed the study would still have good power. However, the most relevant and accurate data is from the Liverpool audit and we have based our sample size calculations accordingly.

It is planned to recruit equal numbers of caesarean and vaginal births to ensure that study results are convincing across both subgroups. This is to ensure that the study has the potential to impact clinical practice across both modes of delivery. Within each subgroup we would be able to detect a decrease in transfusion rate from 5.8% to 3.0% with 85% power (alpha=0.05, fishers exact test).

Transfusion rate (%)	Power (%)
3.5	71
4.0	77
5.8	90
7.5	96
10	99

Relative effect size 0.60, alpha=0.05, fishers exact test

For peer review only - http://bmjopen.bmj.com/site/about/guidelines.xhtml

Repeat uterotonic is an important secondary outcome and this sample size will provide 87% power, using a chi-square test, to detect a 4.2% reduction from a control group rate of 23.7%.

11.3.1 Feasibility (attaining recruitment targets)

In order to continue to the main trial, certain criteria should be satisfied as specified in section 3.

11.4 Interim Monitoring and Analyses

Formal interim analyses of the accumulating data, overall and split by delivery method, will be performed at regular intervals (at least annually) for review by the IDSMC. These analyses will be performed at the LCTC. The IDSMC will be asked to give advice to the Trial Steering Committee (TSC) and Trial Management Group (TMG) on whether the accumulated data from the trial, together with results from other relevant trials, justifies continuing recruitment of further patients or further follow-up. A decision to discontinue recruitment, in all participants or in selected subgroups will be made only if the result is likely to convince a broad range of clinicians including participants in the trial and the general clinical community.

11.5 Analysis Plan

The primary analysis will follow the intention to treat principle as far as practically possible, such that for all women receiving a drug intervention for PPH and for whom the outcome is known will be included in their randomly allocated group regardless of intervention received. All analyses will be conducted using a 5% two-sided level of statistical significance and 95% confidence intervals throughout.

The primary analysis will use negative binomial regression with treatment group and mode of delivery as fixed effects, and site fitted as a random effect. While the primary analysis will include all women with an indicator for mode of delivery, secondary subgroup analyses will be presented for each mode.

The secondary outcomes will be analysed using negative binomial regression for binary outcomes and linear regression for continuous outcomes. The CEQ will be scored according to its scoring manual and analysed using linear regression. A blind review will be undertaken prior to database lock which will assess the suitability of the planned analysis methods.

Baseline characteristics will be presented using descriptive statistics only; there will be no statistical tests between randomised groups.

Adverse Reactions (ARs) and Serious Adverse Reactions/Suspected Unexpected Serious Adverse Reactions (SARs/SUSARs) will also be presented using descriptive statistics only.

11.5.1 Health Economic Analysis

Total costs from the perspective of the NHS and Personal Social Services will be combined with the measure of health outcome to calculate the incremental cost-effectiveness (utility) ratios (ICER) of carboprost vs oxytocin. Where appropriate, missing resource use or health outcome data will be imputed. The number of QALYs experienced by each patient will be calculated as the area under the curve, using the trapezoidal rule, and corrected for 24-hour measurement. We will employ parametric approaches for analysing cost and QALY data that assume normal distributions given the large samples where the near-normality of sample means is approximated. Stratified cost-effectiveness analyses will be conducted on important, pre-specified patient subgroups. Estimates of Incremental cost-effectiveness ratios (ICERs)

For peer review only - http://bmjopen.bmj.com/site/about/guidelines.xhtml

will be compared with the £20,000 to £30,000 per QALY threshold of cost-effectiveness specified by NICE, and a range of sensitivity analyses will be conducted to assess the robustness of the analysis. The joint uncertainty in costs and benefits will be presented as cost-effectiveness acceptability curves.

A full Health Economics Analysis Plan will be completed prior to conducting the analysis.

11.5.2 Qualitative data analysis

Interviews and audio recordings will be transcribed, checked and anonymised as the study progresses. Qualitative researchers will lead the analysis of data. QSR NVivo software will be used to assist in the organisation and indexing of qualitative data. Framework analysis will be used to assist the management and interpretation of data (23). The focus will be modified to fit with the criterion of catalytic validity, whereby findings should be relevant to future research and trial recruitment practice (24, 25).

For peer review only

12 REGULATORY AND ETHICAL APPROVALS

12.1 Statement of Compliance

Statement of compliance: The study will be carried out in accordance with:

- The World Medical Association Declaration of Helsinki (1996)
- LCTC SOPs
- The template content structure is consistent with the SPIRIT (Standard Protocol Item: Recommendations for Interventional Trials 2013) (26)
- UK Policy Framework for Health and Social Care Research 2017.
- Medicines for Human use (Clinical Trials) Regulations 2004 (as amended)

12.2 Regulatory Approval

This trial falls within the remit of applicable trials legislation. This trial has been registered with the MHRA and has been granted a Clinical Trial Authorisation (CTA). The EudraCT number and CTA reference are displayed on the protocol cover page.

12.3 Ethical Approval

The trial protocol has received the favourable opinion of the Research Ethics Committee (REC) but must undergo independent review by the Health Research Authority and capacity and capability assessment at the R&D offices at participating sites.

12.3.1 Ethical Considerations

The study will abide by the principles of the World Medical Association Declaration of Helsinki (1964) and subsequent revisions from 1975 (Tokyo, Japan), 1983 (Venice, Italy), 1989 (Hong Kong), 1996 (Republic of South Africa).

Two key ethical issues have been identified in COPE and our approach has been informed by our Patient & Public Involvement (PPI) activities. These are the consent process and the administration of a placebo intramuscular injection to those allocated to the active oxytocin arm or a placebo intravenous injection for those allocated to the active carboprost arm.

We have undertaken discussions with consumers on the use of placebo injections. These discussions support our study design with a clear consumer preference to the use of a placebo injection to increase the reliability of the answer to the research question. The approaches undertaken are compliant with the required regulations and national guidelines, and fully justifiable. We therefore are cognisant of these issues but believe that the approaches will not raise ethical concerns.

Consent procedures for this emergency study follow the recommendations of consumer groups, the Royal Colleges, and are in compliance with ethical and regulatory frameworks. Information about the study will be distributed to target women during pregnancy through posters, leaflets, websites and on social media. As described in section 6.3, those women who bleed after childbirth and meet the eligibility criteria will be randomised into the trial in the emergency situation where treatment needs to be given urgently and there is no time for prior consent. There will also be the opportunity for prior consent for those at high risk. This approach has been successfully used in several recent trials of emergencies in labour (6, 8, 9).

For peer review only - http://bmjopen.bmj.com/site/about/guidelines.xhtml

12.4 Health Research Authority (HRA) Approval

In order for the study to take place within NHS, an approval from the Health Research Authority (HRA) will be required. This is coordinated by LCTC.

12.4.1 Capacity and capability assessment

The local Research & Development (R&D) offices should be sent the appropriate site-specific information packs complete with the necessary authorisation signatures, plus any other documentation requested for review. This review can take place at the same time as the HRA review. A copy of local R&D capacity and capability assessment should be forwarded to LCTC before the site is initiated and patients recruited.

12.5 Protocol Deviation and Serious Breaches

A breach of the protocol or GCP is 'serious' if it meets the regulatory definition of being "likely to affect to a significant degree the safety or physical or mental integrity of the trial participants, or the scientific value of the trial". All serious breaches of GCP or protocol will be reported to the MHRA and REC in an expedited manner by the LCTC.

If any persons involved in the conduct of the trial become aware of a potential serious breach, they must immediately report this to the LCTC who will in turn notify the sponsor. The sponsor will assess the breach and determine if is meets the criteria of a 'serious' breach of GCP or protocol and therefore requires expedited reporting to the MHRA and REC.

In determining whether or not the breach is likely to affect to a significant degree the safety, physical or mental integrity of participants, the sponsor may seek advice from medical expert members of the TMG and/or of the independent oversight committees (IDSMC and TSC). In determining whether or not the breach is likely to significantly affect the scientific value of the trial, the Sponsor may seek advice form the Trial Statistician. However, the sponsor retains responsibility for the assessment of whether or not a breach meets the definition of 'serious' and is subject to expedited reporting to MHRA and REC.

Breaches confirmed as 'serious' will be reported to the MHRA and REC within 7 days by the LCTC and notified to the TMG, IDSMC and TSC at their next meeting.

Any requests for additional information from the sponsor, TMG, TSC, IDSMC, REC or MHRA, will be promptly actioned by the relevant member(s) of the research team and open communication will be maintained to ensure appropriate corrective actions are taken and documented.

Incidence of protocol non-compliance are recorded as protocol deviations, the incidence of which are monitored and reported to trial oversight committees.

12.6 Study Discontinuation

In the event that the study is discontinued, participating sites will be advised on any actions that are required with regards to individual women's follow-up.

Women will otherwise be treated according to the usual standard clinical care. The process for women who withdraw early from trial is described in section 7.5.

For peer review only - http://bmjopen.bmj.com/site/about/guidelines.xhtml

13 DATA MANAGEMENT AND TRIAL MONITORING

Trial monitoring is carried out to ensure that the rights and well-being of human participants are protected during the course of a clinical trial. A risk assessment is performed for each trial coordinated by the LCTC to determine the level and type of monitoring required for specific hazards. The nature and extent of monitoring will be specific to the individual trial. Monitoring can take the form of on-site visits or central monitoring. A detailed monitoring plan will be developed to describe who will conduct the monitoring, at what frequency monitoring will be done, and what level of detail monitoring will be conducted.

Details of the monitoring to be carried out for the COPE study are included in the COPE Trial Monitoring Plan.

Trial Oversight Committees related to the monitoring of the trial are detailed in section 15.4.

13.1 Source Documents

Source data: *All information in original records and certified copies of original records of clinical findings, observations, or other activities in a clinical trial necessary for the reconstruction and evaluation of the trial. Source data are contained in source documents (original records or certified copies). (ICH E6, 1.51).*

Source documents: *Original documents, data, and records (e.g., hospital records, clinical and office charts, laboratory notes, memoranda, participants diaries or evaluation checklists, pharmacy dispensing records, recorded data from automated instruments, copies or transcriptions certified after verification as being accurate copies, microfiches, photographic negatives, microfilm or magnetic media, x-rays, participant files, and records kept at the pharmacy, at the laboratories and at medico-technical departments involved in the clinical trial). (ICH E6, 1.52).*

In order to resolve possible discrepancies between information appearing in the case report form (CRF) and any other patient related documents, it is important to know what constitutes the source document and therefore the source data for all information in the CRF.

The CRF will be considered the source document for data where no prior record exists and which is recorded directly in the CRF. A COPE source document list will be completed by each site.

Date(s) of conducting informed consent process including date of provision of patient information, randomisation number, confirmation of full eligibility by an authorised medical doctor, and the fact that the patient is participating in a clinical trial should be added to the patient's medical record chronologically.

13.2 Data Capture Methods

The study CRF is the primary data collection instrument for the study. Guidelines regarding data collection requirements, CRF completion and responding to data queries will be provided to sites.

For peer review only

13.3 Monitoring

13.3.1 Central Monitoring

Data stored at LCTC will be checked in accordance with data management and monitoring SOPs. Data clarification forms will be produced and provided to a named individual at a site (as listed on the site delegation log) in order to query data. Returned data clarification forms are filed along with the relevant CRFs after the appropriate corrections made on the database. There are a number of monitoring features in place at the LCTC, detailed in the trial monitoring plan, to ensure reliability and validity of the trial data.

13.3.2 Clinical Site Monitoring

In order to perform their role effectively, the trial coordinator (or other LCTC personnel) and persons involved in Quality Assurance and Inspection may need direct access to primary data, e.g. patient records, laboratory reports, appointment books, etc. Since this affects the patient's confidentiality, this fact is included on the Patient Information Sheet and Informed Consent Form.

13.4 Confidentiality

Individual participant medical information obtained as a result of this study is considered confidential and disclosure to third parties is prohibited without prior agreement in accordance with the Common Law Duty of Confidentiality. Agreement will be achieved during the trial consent process for disclosure by the clinical care staff to the COPE research team, and for sharing of information between the COPE team and NHS Digital and devolved nation equivalents. Medical information may also be given to the participant's wider medical team and all appropriate medical personnel responsible for the participant's welfare.

Data processing will be performed in accordance with applicable data protection legislation. The University of Liverpool and Bangor University are registered with the Information Commissioner's Office; as Data Controllers for this study, they will process data under the legal basis of performing a task in the public interest for research purposes.

The LCTC will be undertaking activities requiring the transfer of personal identifiers (e.g. name):

- Verification that appropriate informed consent for trial participation is obtained will be enabled by the provision of copies of participant's signed informed consent forms to the LCTC by recruiting centres, this requires the transfer of name data to the LCTC.
- Obtaining medical data from NHS Digital and devolved nation equivalents will require LCTC to collect NHS (or CHI) numbers and transfer them to the applicable organisations.

This transfer of identifiable data is disclosed in the Patient Information Sheet.

With the exception of the trial consent form, all other CRFs will be pseudonymised: labelled with the participant's initials and unique trial randomisation number, except for the screening CRFs, which will use participant's screening number.

Sites must ensure other trial documents are not posted in the same envelope as the consent form as there is a risk to patient confidentiality.

For peer review only - http://bmjopen.bmj.com/site/about/guidelines.xhtml

13.4.1 Hospital Episode Statistics (HES)

To obtain HES data and SMR data from NHS Digital, the NHS Wales Informatics Service information Centre and the electronic Data Research and Innovation Service, identifiable patient information will be transferred from LCTC to these NHS organisations as per section 7.4.2. This transfer is going to be disclosed in the trial patient information sheet and consent.

13.5 Quality Assurance and Control

Quality Assurance (QA) includes all the planned and systematic actions established to ensure the trial is performed and data generated, documented/recorded and reported in compliance with applicable regulatory requirements. Quality Control (QC) includes the operational techniques and activities done within the QA system to verify that the requirements for quality of the trial-related activities are fulfilled e.g. state what clinical site monitoring (and audit) is planned, if any. In accordance with the monitoring plan site visits will or will not be conducted and source verification performed, if indicated to be require as a result of central monitoring processes.

- The Trial Coordinator at the LCTC will verify appropriate approvals are in place prior to initiation of a site and the relevant personnel have attended trial specific training. A greenlight checklist will verify all approvals are in place prior to trial initiation at LCTC and the individual site.
- The trial will be conducted in accordance with procedures identified in the protocol.
- The TMG will determine the minimum key staff required to be recorded on the delegation log in order for the site to be eligible to be initiated and will monitor screening, randomisation and consent rates between centres.
- Independent oversight of the trial will be provided by the IDSMC and independent members of the TSC.
- The PI and other key staff from each centre will attend site initiation training, coordinated by the LCTC, which will incorporate elements of trial-specific training necessary to fulfil the requirements of the protocol.
- The process for consent, recruitment and randomisation will be evaluated for compliance with the protocol.
- Data quality checks and monitoring procedures will be undertaken in line with LCTC SOPs.

13.6 Records Retention

The retention period for COPE data and information is 25 years from the official End of Trial date (defined in section 7.7 above).

The PI at each investigational site must make arrangements to store the essential trial documents (as defined by ICH GCP guidelines) including the Investigator Site File, the applicable participant medical records and Pharmacy Site File, for the full length of the trial's retention period and will arrange for confidential destruction at the end of this period as instructed by the LCTC.
The PI is also responsible for archiving all relevant source documents so that the trial data can be compared against source data after completion of the trial (e.g. in case of inspection from authorities). They must ensure the continued storage of the documents, even if they, for example, leave the clinic/practice or retire before the end of required storage period. Delegation of responsibility for this must be documented in writing.

All other persons and organisations involved in the trial will be responsible for storing and archiving the parts of the TMF relevant to their delegated duties (e.g. laboratories, IMP

For peer review only - http://bmjopen.bmj.com/site/about/guidelines.xhtml

manufacturers and distributors, third-party vendors providing randomisation and IMP allocation systems, etc.).

The LCTC undertakes to archive as per their contractual requirements; documents will be archived in compliance with the principles of GCP. All electronic CRFs and trial data will be archived onto an appropriate media for long term accessible storage. Hard copies of data will be boxed and transferred to secure premises where unique reference numbers are applied to enable confidentiality, tracking and retrieval.

For peer review only

For peer review only - http://bmjopen.bmj.com/site/about/guidelines.xhtml

14 INDEMNITY

COPE is sponsored by the University of Liverpool and co-ordinated by the LCTC, University of Liverpool. The University of Liverpool has vicarious liability for the actions of its staff, when through the course of their employment they are involved in the design and initiation of clinical research, including but not limited to the authorship of the Protocol. The University of Liverpool has appropriate insurance in place to cover this liability.

As this is an investigator-initiated study, The Association of the British Pharmaceutical Industry (ABPI) guidelines for patient compensation by the pharmaceutical industry do not apply. However, in terms of liability, NHS Trust and Non-Trust Hospitals have a duty of care to patients treated, whether or not the patient is taking part in a clinical trial, and they are legally liable for the negligent acts and omission of their employees. Compensation is therefore available in the event of clinical negligence being proven.

Clinical negligence is defined as:
"A breach of duty of care by members of the health care professions employed by NHS bodies or by others consequent on decisions or judgments made by members of those professions acting in their professional capacity in the course of their employment, and which are admitted as negligent by the employer or are determined as such through the legal process".

For peer review only

15 ROLES AND RESPONSIBILITIES

15.1 Role of Study Sponsor and Study Funder

This study is funded by the Health Technology Assessment programme of National Institute for Health Research (HTA NIHR) and sponsored by the University of Liverpool. The sponsor's roles and responsibilities are outlined in the COPE collaborators agreement.

15.2 Funding and Support in Kind

Contractual agreements will be in place between sponsor and participating centres that will describe financial arrangements.

Funder(s)	Financial and Non-financial Support Given	Role
NIHR HTA	Financial support is described in the main contract.<br><br>NIHR HTA provides on-going help and support to the CI to ensure that the project progresses smoothly. Trial design, conduct, data analysis and interpretation, manuscript writing and dissemination of results are the responsibility of sponsor and their delegates, though the funder will monitor progress against key milestones via the submission of regular progress reports.<br>The funder requires to approve the initial protocol and amendments prior to submission for ethical/regulatory approval.<br>The funder requires to be provided with copies of project outputs at least 28 days before publication or presentation.	Funder

15.2.1 NHS Research costs

15.2.1.1 Per site

To reflect site personnel time on the study to fulfil the needs of the research, e.g. preparing for an attending initiation/ monitoring meetings, a per-site payment is included in the budget and detailed in the contract between sponsor and site.

15.2.1.2 Per patient recruited

Research related activity, including data collection, will be supported by a per-patient payment. In COPE the patient care pathway mirrors usual care in this acute emergency situation and data collection forms will be formatted to ensure that critical variables can be collected in the

For peer review only - http://bmjopen.bmj.com/site/about/guidelines.xhtml

busy emergency setting. However in order to support the robust recruitment and follow-up of participants, a per-patient payment is budgeted and detailed in contracts. The per-patient payment for transferred participants will be given to the recruiting hospital (see section 7.5.3).

15.2.2 NHS Support costs

This trial will be entered into the NIHR CRN portfolio and infrastructure support will be available through the CRN network.

15.2.3 Treatment costs

Study treatments will be supplied to participating sites by a central manufacturing facility.

15.3 Protocol Contributors

Name	Affiliations (organisation address)	Contribution to protocol
Professor Andrew Weeks	Sanyu Research Unit, University of Liverpool	Clinical aspects, trial design and conduct
Dr Shireen Meher	Birmingham Women's Hospital	Clinical aspects, trial design and conduct
Helen Hickey	Liverpool Clinical Trials Centre, University of Liverpool	Governance arrangements and trial design and conduct
Carrol Gamble	Liverpool Clinical Trials Centre, University of Liverpool	Statistical lead in trial design and conduct including monitoring, and analysis
Charlotte Van Netten	Liverpool Clinical Trials Centre, University of Liverpool	Governance arrangements and trial conduct
Katie Neville	Liverpool Clinical Trials Centre, University of Liverpool	QA oversight
Kerry Woolfall	University of Liverpool	Embedded study design and trial consent process
Louise Roper	University of Liverpool	Embedded study design and trial consent process
Clare Jackson	Liverpool Clinical Trials Centre, University of Liverpool	Data Management and trial monitoring oversight
Sylvia Balabanova	Liverpool Clinical Trials Centre, University of Liverpool	Governance arrangements and trial conduct
Dyfrig Hughes	Bangor University	Health Economics
Catrin Plumpton	Bangor University	Health Economics
Anna Rosala-Hallas	Liverpool Clinical Trials Centre, University of Liverpool	Statistical analyses under the supervision of the statistical team leader
Ben Hardwick	Liverpool Clinical Trials Centre, University of Liverpool	Governance arrangements and trial design and conduct

For peer review only - http://bmjopen.bmj.com/site/about/guidelines.xhtml

15.4 Trial Committees

15.4.1 Trial Management Group (TMG)

A TMG will be formed comprising the Chief Investigator, other lead investigators (clinical and non-clinical) and members of the LCTC. The TMG will meet regularly and is responsible for the day-to-day running and management of the trial. Refer to the TMG terms of reference and trial oversight committee membership document for further details.

15.4.2 Trial Steering Committee (TSC)

The TSC will consist of an independent chairperson, independent experts in the field of clinical obstetrics and a biostatistician. The role of the TSC is to provide overall supervision for the trial and provide advice through its independent Chairman. The ultimate decision for the continuation of the trial lies with the TSC. Refer to the TSC terms of reference and trial oversight committee membership document for further details.

15.4.3 Independent Data and Safety Monitoring Committee (IDSMC)

The IDSMC consists of an independent chairperson, plus 2 independent members: one who is an expert in the field of obstetrics, and one who is an expert in medical statistics.

The IDSMC will be responsible for reviewing and assessing recruitment, interim monitoring of safety and effectiveness, trial conduct and external data. The IDSMC will first convene prior to the start of recruitment and will then define frequency of subsequent meetings (at least annually).

The IDSMC will provide a recommendation to the TSC concerning the continuation of the study. Refer to the IDSMC charter and trial oversight committee membership document for further details.

For peer review only

For peer review only - http://bmjopen.bmj.com/site/about/guidelines.xhtml

16 PUBLICATION AND DISSEMINATION

16.1 Publication Policy

The results from different centres will be analysed together and published as soon as possible. Individual Clinicians must undertake not to submit any part of their individual data for publication without the prior consent of the TMG.

The TMG will form the basis of the Writing Committee and advise on the nature of publications. The Uniform Requirements for Manuscripts Submitted to Biomedical Journals (http://www.icmje.org/) will be respected. The ISRCTN allocated to this trial should be attached to any publications resulting from this trial.

The members of the TSC and IDSMC should be listed with their affiliations in the Acknowledgements/Appendix of the main publication.

16.1.1 Authorship

All publications shall include a list of contributors, and if there are named authors, these should include the trial's Chief Investigator, Statistician(s), Health Economist(s) and Trial Manager(s) involved at least. If there are no named authors (i.e. group authorship) then a writing committee will be identified that would usually include these people, at least.

The members of the TSC and IDSMC should be listed with their affiliations in the Acknowledgements/Appendix of the main publication.

16.2 Dissemination to Key Stakeholders

Trial results will be published in highly reputable peer-review journals and disseminated via scientific meetings and conferences, as well as through the trial website and any professional networks. Trial results will be disseminated regardless of the magnitude or direction of effect. The clinical trial report will be submitted within 12 months of the end of trial.

16.3 Data Sharing

Requests for data sharing following trial closure should be made to the LCTC for consideration.

For peer review only

For peer review only - http://bmjopen.bmj.com/site/about/guidelines.xhtml

17 CHRONOLOGY OF PROTOCOL AMENDMENTS

17.1 Version 1.0 (10/07/2018)

- Original approved version.

17.2 Version 2.0 (10/09/2018)

Summary of Amendments from Protocol V1.0 to Protocol V2.0		
**Protocol Section Number**	**Protocol Section Title**	**Summary of Changes**
10.8	Unblinding	• Removal of the requirement for the investigator to contact the Sponsor before unblinding.
11	Safety Reporting	• Reference to type A trial removed.<br>• Inclusion of all serious adverse events to be recorded from the time of randomisation until hospital discharge or 4 weeks, whichever is sooner.<br>• Removal of the requirement for the investigator to contact the Sponsor before unblinding.

17.3 Version 3.0 (01/05/2019)

Summary of Amendments from Protocol V2.0 to Protocol V3.0		
**Protocol Section Number**	**Protocol Section Title**	**Summary of Changes**
Front Page	N/A	• Additional reference numbers inserted.
Throughout	N/A	• References to vial amended to ampoule.<br>• All references to faxing amended to securely emailing.<br>• Other minor typographical corrections and clarifications in order to ensure consistency throughout.
2	Protocol Summary	• Addition of exclusion criterion “Has already received carboprost prophylactically for postpartum haemorrhage”.<br>• Inclusion of assessment of adverse events at 24 hours, 48 hours/ hospital discharge if sooner and 4 weeks post randomisation.
3.2	Rationale	• Inclusion of reference to IaBOR study: “The only risk of this is hypotension that occurs with rapid injection, however this was not seen in a recent randomised controlled trial in which 517 women were given 10 international units of oxytocin as an i.v. bolus over 1 minute.”<br>• Inclusion of reference to confidential enquiries into maternal deaths.

For peer review only - http://bmjopen.bmj.com/site/about/guidelines.xhtml

3.4	Outcome measures	• Clarification of pharmacological interventions included within footnote 4.
6.2	Exclusion Criteria	• Addition of exclusion criterion "Has already received carboprost prophylactically for postpartum haemorrhage".
7.5	Who is blinded to allocations	• Responsibility of Pharmacy amended to site team for periodic checks of unblinding envelopes.
8.1	Schedule for Follow-up	• Inclusion of reference to footnote 4 within schedule for follow-up table.<br>• Update to footnote 7 in line with update to section 11.1.
9.3	Embedded Mixed Methods Research – Enrolment & Procedure	• Update to upload of audio recordings to online folder for transcription purposes.<br>• Clarification of management of interviews with women and birth partners.
10.2	Formulation, Packaging, Labelling, Storage and Stability	• Amendment of wording from "COPE trial active treatments (carboprost and oxytocin) are market-authorised drugs being used within the terms of their marketing authorisation" to "COPE trial active treatments (carboprost and oxytocin) are market-authorised drugs".<br>• Reference to oxytocin as "aqueous solution for infusion" amended to "aqueous solution for intravenous injection".<br>• Storage and stability process clarified.<br>• Inclusion of more generic reference to clinical services company.
10.3	Distribution, Dispensing, Dosage and Administration of Study Treatment/s	• Distribution and Dispensing process clarified.<br>• Amendment of wording from "Both ampoules should be administered at randomisation in accordance with the respective Summary of Product Characteristics (SPC) accessed via the electronic medicines compendium (eMC)" to "Both ampoules should be administered at randomisation in accordance with local practice".
10.7	Concomitant medications/Treatments	• Removal of recording of dose and dose duration for concomitant medications.
11.1	Time Period for Safety Reporting	• Update to reportable adverse events. "Reporting of adverse reactions considered to be "possibly", "probably", or "almost certainly" related to the randomised trial treatment" amended to "adverse reactions of particular interest (safety reports that do not meet the "serious" criteria and are listed within reactions of particular interest)".<br>• Inclusion of "adverse reaction of particular interest" list.<br>• Figure 4 updated in line with safety reporting.
11.3	Notes on Adverse Reaction Inclusions and Exclusions	• Update to reportable adverse events. "Reporting of adverse reactions considered to be "possibly", "probably", or "almost certainly" related to the randomised trial treatment" amended to "adverse reactions of particular interest (safety reports that do not meet the "serious" criteria and are listed within reactions of particular interest)."
11.8	Reporting procedures	• Clarification of safety reporting procedures.

For peer review only - http://bmjopen.bmj.com/site/about/guidelines.xhtml

11.9	Responsibilities - Investigator	• Clarification of safety reporting procedures.
11.12	Safety reports	• Clarification of safety reporting procedures.
12.5	Analysis plan	• Inclusion of more detailed analysis plan.

17.4 Version 4.0 (13/07/2020)

Summary of Amendments from Protocol V3.0 to Protocol V4.0		
**Protocol Section Number**	**Protocol Section Title**	**Summary of Changes**
Front Page	N/A	• Title clarified.<br>• Addition of LCTC logo and CTRC and LCTU merger text.
Throughout	N/A	• All references to and addresses associated with Clinical Trials Research Centre (CTRC) updated to Liverpool Clinical Trials Centre (LCTC).<br>• Section numbering updated.<br>• "Oxytocin 10IU by slow intravenous injection over 2 minutes and 1ml placebo by deep intramuscular injection" amended to "Oxytocin (5 international units (IU) for caesarean section or 10IU for vaginal delivery) by slow intravenous injection over 2 minutes and 1ml placebo by deep intramuscular injection".<br>• All references to administration of intravenous injection updated to administration over 2 minutes.<br>• All references to the internal pilot "using data from the first five hospitals opened" amended to "using data collected during the first 6 months of recruitment"<br>• Other minor typographical corrections and clarifications in order to ensure consistency throughout.<br>• All references to "participant" amended to "woman", where necessary.<br>• All references to "emergency consent" amended to "postnatal (emergency pathway) consent".<br>• General formatting changes.
Protocol Approval	N/A	• Separation of approval signature pages.
1.0	Protocol Summary	• Embedded mixed methods research timelines updated.
1.1	Schematic of Study Design	• Schematic updated in line with oxytocin dosage changes and changes to data collection.
2.0	Introduction	• Removal of duplicated information.<br>• Embedded mixed methods research timelines updated.
3.0	Trial Design	• Pilot study clarified by addition of "Other sites may be started during this first 6 months so as to accelerate the overall recruitment timetable, but data from these will be included in the main study analysis only, not the pilot study analysis."
6.2	COPE recruitment pathways	• Schematic design updated and clarified.

For peer review only - http://bmjopen.bmj.com/site/about/guidelines.xhtml

6.3	Consent Procedures	• Clarification that postnatal consent (emergency pathway) is sought for disclosure of identifiable data and for follow-up.<br>• Grouping of information provision and consent in each of the recruitment pathways.
6.4	Randomisation Procedures	• Addition of: "All women randomised in to COPE (treatment kit removed from fridge following confirmation of eligibility) who go on to receive the COPE treatment or an alternative medicinal treatment for PPH should be approached for their consent and followed up as a COPE participant as per COPE protocol. If a treatment kit was removed from the fridge but a medicinal treatment for PPH was not administered (COPE treatment kit or other) then consent does not need to be sought and they should not be followed up as a COPE participant."
7.1	Schedule for follow-up	• Table updated to clarify follow-up schedule and group outcomes by CRF.
7.4.1	Childbirth Experience Questionnaire (CEQ)	• Inclusion of option to use telephone version of CEQ at 4 weeks, as well as paper version.
7.4.2	Health Economics Assessments	• "cost questionnaire" amended to "resource use questionnaire"<br>• Explanation of HES/ SMR data process simplified.<br>• "Once the analysis has been completed the HES and SMR data will be securely disposed in accordance with the CHEME and NHS organisations Data Services Commissioners (DSfC)" Amended to "Electronic HES and SMR records will be stored on secure Bangor University computer servers which meet NHS data security standards".
8.0	Embedded mixed methods research	• Clarification of timing of embedded study: "For at least the first 9 months of recruitment, we will use interviews, and questionnaires to explore the views and experiences of women recruited to the trial and their birth partners. In the five pilot sites (or alternative site if a pilot site is not open to recruitment within two months of recruitment of the first woman to the trial) we will also conduct focus groups with practitioners and audio-recorded recruitment discussions."
9.3.2	Distribution, Dispensing, Dosage and Administration of Study Treatment/s – Dosage and Administration	• Intervention use during the 15 minute post IMP administration clarified; "Once the IMP is administered, treating clinicians should not use further carboprost or oxytocin bolus within 15 minutes, this time represents the half-life of intravenous oxytocin, and is the time interval specified for repeat doses of carboprost. Additional pharmacological treatments should be avoided within the 15 minutes, where necessary. However, if a woman continues to experience heavy bleeding, the managing clinician may use the below treatments during this period"<br><br>Treatments that can be used during the 15 minute period clarified.<br><br>"After the 15 minute period, further management should be conducted in accordance with NICE guidance and local practice for the management of PPH, including the use of further oxytocin (no more than 10 additional units applied as a bolus) or carboprost (no more than 7 additional doses)."

		Section 7.5.1 – Discontinuation and section 9.6.1 – Medications Permitted/Not Permitted/ Precautions Required, have also been updated in line with this change.
9.5	Overdose	• Amendment of wording from "As part of the COPE protocol women will be administered a single dose of carboprost or oxytocin that are each within the parameters of the dose advised in their respective SPC. If administered in accordance with the relevant SPC and this protocol, overdose is unlikely." to "As part of the COPE protocol women will be administered a single dose of carboprost or oxytocin. If administered in accordance with this protocol, overdose is unlikely."
9.7	Unblinding	• Unblinding process clarified.
10.0	Safety Reporting	• Removal of non-serious adverse reactions of particular interest from monitoring and reporting period of randomisation until hospital discharge or 4 weeks, whichever is sooner. All such reactions appear within 2 hours of IMP administration, therefore will be collected on a separate single timepoint CRF.<br>• Flowchart of Adverse Events reporting requirements updated in line with the above.
10.2	Reference Safety Information	• Addition of "As the placebo is inert there are no expected adverse reactions, therefore any serious adverse reactions thought to be related to the placebo would be unexpected and reported as a potential SUSAR".
11.2	Method of Randomisation	• "Upon randomisation the research team will select the next sequentially numbered kit" amended to "Upon randomisation the research team will select the .next sequentially numbered kit for the particular mode of birth"
11.5.1	Health Economic Analysis	• "The joint uncertainty in costs and benefits will be considered through the application of bootstrapping and the estimation of cost-effectiveness acceptability curves" amended to "The joint uncertainty in costs and benefits will be presented as cost-effectiveness acceptability curves".
13.6	Records Retention	• Section simplified by the removal of ICH GCP essential document guidance.
15.3	Protocol Contributors	• Dyfrig Hughes added as a protocol contributor.
Appendix	COPE Outcomes	• Blood loss outcome: amended from "Estimated or measured vaginal blood loss in mls commencing in the first 24 hours from randomisation, up to cessation of active bleeding. The estimated blood loss will be recorded, and supported by blood loss collection and/or weighing where possible." to "Estimated or measured vaginal blood loss in mls from randomisation to cessation of first active bleeding. The estimated blood loss will be recorded, and supported by blood loss collection and / or weighing where possible."<br>• Blood loss ≥ 1000 mls outcome: Amended to "Volume of blood loss ≥ 1000 mls, as described above."<br>• Maternal death outcome: Amended from "Death of the mother within 4 weeks of the birth where postpartum haemorrhage was a contributing factor (it does not need to be the primary cause)" to

For peer review only - http://bmjopen.bmj.com/site/about/guidelines.xhtml

		"All deaths in participants from the time of randomisation until hospital discharge or 4 weeks, whichever is earlier."<br>• Hypotension definition clarified: "A fall in blood pressure requiring treatment, or maternal symptoms of hypotension, developed within 2 minutes of IMP administration".<br>• Resource use clarified "Resource use will include direct medical costs using questionnaire and hospital episode statistics. Quality-adjusted life years will be estimated using the using EQ-5D-5L; and the incremental cost effectiveness ratio calculated as the economic outcome of interest"

17.5 Version 5.0 (14/04/2021)

**Summary of Amendments from Protocol V4.0 to Protocol V5.0**		
**Protocol Section Number**	**Protocol Section Title**	**Summary of Changes**
N/A	Protocol Approval	• Sponsor signatory amended from Alex Astor to Dr Neil French.
N/A	Contact Details - Institutions	• Sponsor contact details updated.
N/A	Contact Details - Individuals	• Sponsor signatory amended from Alex Astor to Dr Neil French.<br>• Individual addresses/contact details updated, as required.
8.3	Embedded Mixed Methods Research – Enrolment & Procedure	• **Part B: Questionnaires** - Addition of "If questionnaires are not returned the embedded study team will ask women and birth partners to complete the questionnaire online at the point of arranging interviews (see below).<br>• **Part C: Interviews with women and their birth partner** - Inclusion of the option to carry out embedded study interviews online, in addition to via telephone or face-face (for participants located in the North West).<br>• Clarification that qualitative researchers will provide interviewees with a thank you letter, a high street voucher, and contact participants by email, not site staff.<br>• **Part D: Focus groups and/or interviews with the COPE practitioners** -<br>– Clarification of interviews/ focus groups format and addition of online option: "online focus group or remote interview (telephone or online via Zoom or Microsoft Teams) (whichever is possible to arrange around clinical practice).<br>– Clarification of the arrangement of interviews/ focus groups with practitioners.
10.1	Safety Reporting – Time Period for Safety Reporting	• Addition of: "IMPORTANT: Any safety events related to the trial treatment (see section 10.7) occurring after the end of this "active monitoring" period which meet the definition of serious (see section 10.4), related (see section 10.7) and are recorded for this study

For peer review only - http://bmjopen.bmj.com/site/about/guidelines.xhtml

		(see section 10.10) must continue to be reported by sites to the LCTC in accordance with the timeframes and procedures described in section 10.10. The same processes established for SAEs within the active monitoring period should be followed for these events."<br>• Clarification that "Non-serious adverse events and non-serious adverse reactions (not listed within reactions of particular interest) do not require reporting."
10.4	Safety Reporting – Terms and Definitions	• Serious Adverse Event (SAE), Serious Adverse Reaction, or Unexpected Serious Adverse Reaction definition clarification – Removal of "in offspring of subjects, or their partners, taking the IMP regardless of time of diagnosis" from "consists of congenital anomaly or birth defect" item.<br>• Addition of "N.B. "seriousness" assessment must be performed by an appropriately delegated, medically qualified member of the site research team."
10.7	Safety Reporting – Relationship to Trial Treatment	• Inclusion of "the opinion of the treating investigator will never be downgraded"<br>• "An AE whose causal relationship to the study drug is assessed by the investigator as "possible", "probable", or "almost certainly" is an Adverse Reaction (AR)." Moved from section 10.8 to section 10.7.
10.8	Safety Reporting – Expectedness	• Clarification of expectedness processes carried out by CI.
10.10	Safety Reporting – Reporting Procedures	• Email address updated from cope@liverpool.ac.uk to lctcsafe@liverpool.ac.uk for transfer of SAE reports from site to LCTC.

17.6 Version 6.0 (11/05/2022)

Summary of Amendments from Protocol V5.0 to Protocol V6.0		
**Protocol Section Number**	**Protocol Section Title**	**Summary of Changes**
Throughout	N/A	• Removal of the antenatal recruitment pathway
N/A	Protocol Approval	• Sponsor signatory amended from Dr Neil French to Karen Wilding
N/A	Contact Details - Institutions	• Sponsor contact details updated.
N/A	Contact Details - Individuals	• Sponsor signatory amended from Dr Neil French to Karen Wilding<br>• Individual addresses/contact details updated, as required.
3.0	Trial Design	• Clarification of the pilot study timing.
6.3	Consent Procedures	• Clarification of the use of data "Anonymous data, for the purposes of the recipients, will be sent to the LCTC for processing under public interest to allow monitoring of safety. Personal identifiable

For peer review only - http://bmjopen.bmj.com/site/about/guidelines.xhtml

		information will be subsequently sent to LCTC following written informed consent.”
6.3.3	Consent Procedures – Discharge/ transfer to another hospital prior to postnatal consent	• Clarification of processes option following discharge/transfer to another hospital prior to postnatal consent and the addition of e-consent as an option.
8.1	Embedded mixed methods research	• Confirmation that recruitment to embedded mixed methods research has closed.
9.2.3	Storage and stability	• Addition of procedures for re-labelling stock that experiences an excursion over 8°C.

For peer review only

For peer review only - http://bmjopen.bmj.com/site/about/guidelines.xhtml

18 REFERENCES

1. Protocol guidance and template for use in a Clinical Trial of an Investigational Medicinal Product (CTIMP). NHS Health Research Authority Guidance 2016
2. Organization WH. Evaluating the quality of care for severe pregnancy complications. The WHO near-miss approach for maternal health. 2011.
3. Weeks A. The prevention and treatment of postpartum haemorrhage: what do we know, and where do we go to next? BJOG. 2015;122(2):202-10.
4. Knight M NM, Tuffnell D, Shakespeare J, Kenyon S, Kurinczuk JJ (Eds.) on behalf of MBRRACE-UK. Saving Lives, Improving Mothers' Care - Lessons learned to inform maternity care from the UK and Ireland Confidential Enquiries into Maternal Deaths and Morbidity 2013–15. Oxford: National Perinatal Epidemiology Unit, University of Oxford. 2017.
5. (NICE) NIfHaCE. NICE 2014. Intrapartum care: care of healthy women and their babies during childbirth. NICE Clinical guideline CG190. . 2014.
6. Pushpa-Rajah A, Bradshaw L, Dorling J, Gyte G, Mitchell EJ, Thornton J, et al. Cord pilot trial - immediate versus deferred cord clamping for very preterm birth (before 32 weeks gestation): study protocol for a randomized controlled trial. Trials. 2014;15:258.
7. Gynaecologists RCoOa. Obtaining Valid Consent to Participate in Perinatal Research Where Consent is Time Critical. Clinical Governance Advice No. 6a. London RCOG; 2016. 2016.
8. Shakur H, Elbourne D, Gulmezoglu M, Alfirevic Z, Ronsmans C, Allen E, et al. The WOMAN Trial (World Maternal Antifibrinolytic Trial): tranexamic acid for the treatment of postpartum haemorrhage: an international randomised, double blind placebo controlled trial. Trials. 2010;11:40.
9. Vernon G, Alfirevic Z, Weeks A. Issues of informed consent for intrapartum trials: a suggested consent pathway from the experience of the Release trial [ISRCTN13204258]. Trials. 2006;7:13.
10. POSTPARTUM HAEMORRHAGE, PREVENTION AND MANAGEMENT (Green-top Guideline 52) [Online]. London. [Internet]. 2009.
11. Knight M KS, Brocklehurst P, Neilson J, Shakespeare J, Kurinszuk JJ (Eds) on behlf od MBRRACE-UK. Saving Lives, Improving Mothers' Care - Lessons learned to inform future maternity care from the UK and Ireland Confidential Enquiries into Maternal Deaths and Morbidity 2009-12. Oxford: National Perinatal Epidemiology Unit, University of Oxford 2014. 2014.
12. Carvalho JC, Balki M, Kingdom J, Windrim R. Oxytocin requirements at elective cesarean delivery: a dose-finding study. Obstet Gynecol. 2004;104(5 Pt 1):1005-10.
13. Balki M, Ronayne M, Davies S, Fallah S, Kingdom J, Windrim R, et al. Minimum oxytocin dose requirement after cesarean delivery for labor arrest. Obstet Gynecol. 2006;107(1):45-50.
14. Adnan N, Conlan-Trant R, McCormick C, Boland F, Murphy DJ. Intramuscular versus intravenous oxytocin to prevent postpartum haemorrhage at vaginal delivery: randomised controlled trial. BMJ. 2018;362:k3546.
15. Balki M, Erik-Soussi M, Kingdom J, Carvalho JC. Oxytocin pretreatment attenuates oxytocin-induced contractions in human myometrium in vitro. Anesthesiology. 2013;119(3):552-61.
16. Widmer M, Blum J, Hofmeyr GJ, Carroli G, Abdel-Aleem H, Lumbiganon P, et al. Misoprostol as an adjunct to standard uterotonics for treatment of post-partum haemorrhage: a multicentre, double-blind randomised trial. Lancet. 2010;375(9728):1808-13.
17. Tuncalp O, Hofmeyr GJ, Gulmezoglu AM. Prostaglandins for preventing postpartum haemorrhage. Cochrane Database Syst Rev. 2012(8):CD000494.
18. Mousa HA, Blum J, Abou El Senoun G, Shakur H, Alfirevic Z. Treatment for primary postpartum haemorrhage. Cochrane Database Syst Rev. 2014(2):CD003249.
19. Thomas JS, Koh SH, Cooper GM. Haemodynamic effects of oxytocin given as i.v. bolus or infusion on women undergoing Caesarean section. Br J Anaesth. 2007;98(1):116-9.

For peer review only - http://bmjopen.bmj.com/site/about/guidelines.xhtml

20. Why Mothers die 1997 - 1999. The Confidential Enquiries into Maternal Deaths in the United Kingdom. 2001.
21. Mavrides E AS, Chandraharan E, Collins P, Green L, Hunt BJ, Riris S, Thomson AJ on behalf of the Royal College of Obstetricians and Gynaecologists. . Prevention and management of postpartum haemorrhage. BJOG. 2016;124:e106–e49.
22. Gamble C, Krishan A, Stocken D, Lewis S, Juszczak E, Dore C, et al. Guidelines for the Content of Statistical Analysis Plans in Clinical Trials. Jama. 2017;318(23):2337-43.
23. RITCHIE J, LEWIS J, NICHOLLS CM, ORMSTON R. Qualitative Research Practice. SAGE L, editor2013.
24. B. G. The Constant Comparative Method of Qualitative Analysis. Social Problems. 1965;12(4):436-45.
25. Strauss A CJ. Basics of qualitative research: techniques and procedures for developing grounded theory 2nd edition. Thousand Oaks, CA: Sage, 1998.
26. The SPIRIT Statement: http://www.spirit-statement.org/ [Accessed on 12/12/2017]. 2013.

For peer review only

APPENDIX 1. COPE OUTCOMES

Objectives	Outcome Measure	Definition, time frame of assessment and procedure		Timepoint(s) of data collection
**Primary Objective**	Primary outcome			
To compare carboprost with oxytocin as initial treatments for women with clinically diagnosed PPH after giving birth in British hospitals.	Blood transfusion	Any RBC blood transfusion or cell salvage of ≥ 300mls commenced any time between randomisation and 48 hours after randomisation (or hospital discharge if earlier than 48 hrs)		After hospital discharge
	Secondary outcomes			
	1. Volume of Blood transfusion	Data will be collected on total units of blood transfusion given, from randomisation up to 48 hrs (or hospital discharge if earlier).		After hospital discharge
	2. Use of a further uterotonic drug	Use of any uterotonic agent additional to IMP administered to control ongoing bleeding, from time of randomisation up to 24 hrs after randomisation.		After hospital discharge
	3. Composite outcome of any organ dysfunction	The composite includes any woman who develops one or more of the following from randomisation up to hospital discharge or 4 weeks, whichever is earlier. Composite based on the definitions used in the WHO Near-miss approach for maternal health' (2). Each of the items will also be reported separately.		After hospital discharge or 4 wks, whichever is earlier
		Renal dysfunction		
		Acute renal failure	Dialysis for renal failure or severe acute azotemia: creatinine ≥ 300 µmol/l or ≥ 3.5mg/dl	
		Oliguria non-responsive to fluids or diuretics:	A urinary output <30ml/h for 4 hours or <400ml/24h non-responsive to fluids or diuretics	
		Cardiovascular dysfunction		
		Cardiac arrest	Sudden absence of pulse and loss of consciousness or cardio-pulmonary resuscitation	
		Cardiopulmonary resuscitation	A set of emergency procedures including chest compressions and lung ventilation applied in cardiac arrest victims	
		Use of continuous vasoactive drugs:	The continuous use of any dose of dopamine, epinephrine or norepinephrine. In the context of vasoactive drugs infusion, continuous use refers to the uninterrupted infusion of a solution	

For peer review only - http://bmjopen.bmj.com/site/about/guidelines.xhtml

			containing a vasoactive drug. It is opposed to the intermittent or in bolus injection of a vasoactive drug.	
		Persistent shock	Systolic blood pressure <80mmHg that fails to respond to treatment with a fluid challenge	
		Severe hypoperfusion	Lactate >5mmol/l or 45mg/dl	
		Severe acidosis	A blood pH <7.1	
		Coagulation/haematologic dysfunction		
		Coagulopathy	Severe Acute thrombocytopenia (<50 000 platelets/ml), low fibrinogen (<100 mg/dl), prolonged prothrombin time (≥1.5x normal), or Fibtem A5 <6mm.	
		Massive transfusion	Transfusion of ≥5 units of blood or red blood cells.	
		Neurologic dysfunction		
		Prolonged unconsciousness	Any loss of consciousness lasting more than 12 hours, involving complete or almost complete lack of responsiveness to external stimuli / Glasgow Coma Scale <10.	
		Stroke	Acute death of brain cells in a localised area due to inadequate blood flow, diagnosed clinically.	
		Uncontrollable fits	Refractory, persistent convulsions. Status epilepticus.	
		Total paralysis	The complete or partial paralysis of both sides of the body. Usually, an extreme neuromuscular global weakness associated with critical illness. This condition is also known as critical illness polyneuromyopathy.	
		Respiratory dysfunction		
		Severe tachypnoea	Respiratory rate of more than 40 breaths per minute.	
		Severe bradypnea	Respiratory rate of less than 6 breaths per minute	
		Severe hypoxemia	Oxygen saturation <90% for ≥60min or $PaO_2/FiO_2<200$	
		Ventilation	Intubation and ventilation not related to anaesthesia	
		Acute cyanosis	Acute onset of bluish discolouration of mucous membranes and lips	
		Hepatic dysfunction		
		Severe acute hyperbilirubinaemia	Bilirubin >100 µmol/L or >6.0 mg/dL	
		Jaundice in the presence of pre-eclampsia	Acute onset yellowish discolouration of skin and sclera occurring in the presence of pre-eclampsia [blood pressure of greater than 140/90mmHg in the presence of proteinuria (+) or more on urinary dipstick or Protein-creatinine ratio > 30mg/mmol)]	

For peer review only - http://bmjopen.bmj.com/site/about/guidelines.xhtml

		Hysterectomy	Surgical removal of the uterus to treat postpartum haemorrhage or infection from any time after randomisation up to hospital discharge (or 4 weeks, whichever is earlier).	
	4. Hysterectomy	Surgical removal of the uterus any time after randomisation up to hospital discharge (or 4 weeks, whichever is earlier)		After hospital discharge
	5. Blood loss	Estimated or measured vaginal blood loss in mls from randomisation to cessation of first active bleeding. The estimated blood loss will be recorded, and supported by blood loss collection and / or weighing where possible.		After hospital discharge
	6. Blood loss ≥ 1000mls	Volume of blood loss ≥ 1000 mls, as described above.		After hospital discharge
	7. Haemoglobin	Postnatal Haemoglobin (Hb) closest to 24 hours after randomisation<br><br>Hb in non-transfused women-will be ideally obtained postnatally on the day following birth (12-36 hours post-randomisation) or at discharge, whichever is soonest. If repeated Hb measures have been obtained in the 12-36 hour window then the value obtained closest to 24 hours will be used		After hospital discharge,
	8. Shock	The presence of systolic blood pressure <80mmHg within 24 hours of randomisation		After hospital discharge
	9. Maternal death	All deaths in participants from the time of randomisation until hospital discharge or 4 weeks, whichever is earlier.		After hospital discharge
	10. Non pharmacological approach to treat or investigate bleeding	Use of any non-pharmacological approach to treat bleeding, from randomisation up to hospital discharge.<br>Such non-pharmacological interventions include: laparotomy, internal uterine tamponade with balloon or uterine packing, arterial embolization, removal of retained products.		After hospital discharge
	11. Manual removal of placenta	Manual removal of placenta required post randomisation up to hospital discharge.		After hospital discharge
	12. Adverse reactions of particular interest	1. Hypotension - A fall in blood pressure requiring treatment, or maternal symptoms of hypotension, developed within 2 minutes of IMP administration.<br><br>Adverse reactions of particular interest as listed below, occurring within 2 hrs of IMP administration:<br><br>2. Vomiting<br>3. Pyrexia; temperature of >38°C		From randomisation until hospital discharge.

For peer review only - http://bmjopen.bmj.com/site/about/guidelines.xhtml

		4. Headache<br>5. Hot flushes<br>6. Diarrhoea		
	13. Skin to skin care with baby within the first hour after birth	Where the newborn is placed unwrapped against the mother's bare chest or belly from any time after randomisation up to 1 hour after birth.		At 24 hrs
	14. Separation from new-born in first hour after birth	Separation of the newborn from the mother post randomisation up to the first hour after birth.<br>.		At 24 hrs
	15. Breastfeeding	'Breastfeeding' refers to feeding of the baby with the mother's breast milk, even if this is expressed breast milk given by cup or bottle		At 24 hrs, 48 hrs (or hospital discharge if sooner) and 4 wks
		Initiation	Breastfeeding initiated within the first 24 hours after birth.	
		Exclusively at hospital discharge	Exclusive breastfeeding at hospital discharge (i.e. no formula milk, other liquids, or food)	
		Exclusively at 4 weeks post-birth	Exclusive breastfeeding at 4 weeks post birth (i.e. no formula milk, other liquids, or food)	
	16. Childbirth experience questionnaire	Questionnaire on childbirth experience administered to women at 4 weeks postnatally.		At 4 wks
**Secondary Objective**<br>To assess relative cost-effectiveness of use of carboprost or oxytocin as initial treatments for women with clinically diagnosed primary PPH.	17. Resource use	Resource use will include direct medical costs using questionnaire and hospital episode statistics. Quality-adjusted life years will be estimated using the using EQ-5D-5L; and the incremental cost effectiveness ratio calculated as the economic outcome of interest.		At 24 hrs and 4 wks.
		QALY measured using EQ-5D-5L		
		Bespoke self-report instrument designed to capture participants' resource use.		

For peer review only

		Electronic data records of hospitalization will be requested from NHS Digital (England) and devolved equivalents, based on NHS (or equivalent) Number.<br><br>**This will include data on transfer to a higher level of care**<br>This is the transfer of the place of care **due to** a postpartum haemorrhage. This refers to any transfer for more specialist care, but examples would be a transfer from home to hospital, from one hospital to another for specialist input, from a high dependency care unit to an intensive care unit, or from a midwifery led unit to an intensive care unit.	Data will be requested following completion of follow-up from NHS Digital and devolved equivalents.

For peer review only

For peer review only - http://bmjopen.bmj.com/site/about/guidelines.xhtml

COPE
The Carboprost or Oxytocin Postpartum haemorrhage Effectiveness Study

<Trust address 1>
<Trust address 2>
<Trust address 3>
<postcode>

<telephone number>

COPE: Carboprost vs Oxytocin as the First Line Treatment of Primary Postpartum Haemorrhage; A phase IV, double-blind, double-dummy, randomised controlled trial.

Emergency Patient Information Sheet and Consent Form

Version 1.0: 12-07-2018

Contents — Page

Contents	Page
1) Why am I being asked after I was given the medicine?	2
2) Why are we doing the study?	2
3) What happens if I agree for you to use my information?	2
4) Do I have to take part?	3
5) How was it decided which medicine I received?	3
6) Timeline of visits	3
7) What are the alternatives for treatment?	3
8) What are the benefits and risk of taking part?	4
9) Will my participation be kept confidential?	4
10) What if there is a problem?	4
11) What will happen to the results of the study?	4
12) What are my electronic hospitalisation records?	4
13) Who is running the study?	5

Contact

Principal Investigator: **<PI NAME>**
Research Midwife: **<RM NAME>**
Telephone: **<number>**

Or visit the website: www.copestudy.uk

Information about the study

Excessive bleeding after childbirth (also known as postpartum haemorrhage or PPH) is a common problem which affects 1 in 20 women.

COPE is a research study to compare two drugs currently used to treat PPH, to decide which is better. The two drugs are **carboprost** and **oxytocin**.

Before you decide if you want to give your permission for your information to be used in this study, it is important for you to understand why the study is being done and what is involved.

Please take time to read the following information carefully and discuss it with friends or relatives if you wish. Taking part is voluntary. If you don't want to take part then you don't need to give a reason.

<u>Your decision will not affect how the doctors and nurses will look after you.</u>

Please ask a member of your clinical team if there is anything that is not clear, or if you would like more information.

Important things that you need to know

PPH is a medical emergency and needs to be treated without delay; it is important to prevent excessive bleeding as quickly as possible.

After you had given birth, you experienced a PPH. As this is a medical emergency, the priority is to treat the PPH and there is not time to properly discuss whether you would like to take part in COPE before giving treatment for PPH.

The ethical committee and medicines regulator have approved that women can be treated as part of the COPE research study and then approached after the medical emergency has passed to see if you are happy for us to collect some information for the research study.

For peer review only - http://bmjopen.bmj.com/site/about/guidelines.xhtml

Thank you for taking the time to read this information sheet. We hope you will find this information helpful.

Why am I being asked after I was given the medicine rather than before?

After you had given birth, you experienced a PPH.

PPH is a medical emergency and needed to be treated without delay; it was important to prevent excessive bleeding as quickly as possible. As there wasn't time to properly discuss the COPE research study we have approval from the ethical committee and medicines regulator to treat women as part of the COPE research study and then to ask for consent afterwards. This is research without prior consent (sometimes called deferred consent).

As part of the COPE study, when you experienced a PPH you were given two injections – one into the muscle and one into the vein. You were given either carboprost and placebo or oxytocin and placebo. A placebo is a salt solution containing no active drug – acting as a "dummy drug". Carboprost is injected into the muscle and oxytocin injected into the vein, therefore including a placebo as well as carboprost or oxytocin, in this way, means that neither you nor your doctor know what treatment you were given. This ensures a fair test.
We have therefore come to talk to you about the study as soon as possible after the medical emergency to ask for your permission to use information we have collected about your hospital stay.

Why are we doing the COPE study?

It is important to stop excessive bleeding after birth (PPH) as quickly as possible, as the blood loss can leave women feeling extremely weak and tired. They can then find it hard to care for their newborn baby. This hospital is one of about 40 hospitals in the UK involved in the COPE Study. About 4000 women will take part. We want to know if it is better to use carboprost or oxytocin as the first drug for treatment if a woman has a PPH.

The results from COPE will help doctors know which treatment is better for women with PPH.

What happens if I agree that you can use my information?

A member of the clinical team will talk to you in detail and you will be able to ask any questions that you have. If you have had all of your questions answered and are happy for your information to be collected then you will be asked to sign a consent form to confirm this. You will be given a copy of the consent form and the information sheet to keep.

We will collect some information about your health and we will ask you some questions about your experience in the study, your experience of childbirth and your general well-being. These questions will be asked before you leave the hospital and then again at four weeks after the birth. We will contact you via telephone, email or text to follow-up.

<u>Embedded study: Your views on how we ask people to take part in COPE are important</u>

You and your birth partner (if applicable) may be asked to take part in a telephone interview or face to face interview if you live in the North West and/or complete a questionnaire. Interview discussions will last about 60 minutes and will include how you felt about the COPE study. This study will inform how we ask other parents/ relatives about COPE and future related research. Again, it is up to you whether you take part or not, and your care will not be affected if you do not take part.

Do I have to take part?

No. Taking part is voluntary. If you choose to take part you can also choose to stop at any time without giving a reason. The standard of care you receive now and in the future will be the same whether you take part or not.

For peer review only - http://bmjopen.bmj.com/site/about/guidelines.xhtml

COPE
The Carboprost or Oxytocin Postpartum haemorrhage Effectiveness Study

NHS

If at any point you decide to stop taking part in the study, you will receive the care usually offered by your hospital following PPH.

We will use any study information collected up until the time you stop taking part; this information will be pseudo-anonymised, meaning that identifying details, (e. g. your name) will only be accessed by people working on the study.

How was it decided which medicine I received?

In research studies we often split people up into groups to look at how different treatments work. People in one group get a different treatment than patients in another group. In the COPE study there are two treatment groups:

Group A: Women will receive oxytocin and placebo

Group B: Women will receive carboprost and placebo

It is really important that each group in the COPE study has a similar mix of patients in it so we know that if one group of patients does better than the other it is because of the treatment and not because there are differences in the types of women in each group.

We use a computer programme that puts women 'at random' into one of the groups – you might hear this described as 'randomisation' or 'random allocation', but they all mean the same thing. So, neither you nor your doctor chose your treatment. In the COPE study, you are equally as likely to be in the group receiving oxytocin as you are in the group receiving carboprost.

COPE is a 'blinded study' which means neither you nor your doctor will know what your treatment is. In the event of an emergency, the name of your treatment can be found out quickly.

Timeline of visits

Bleeding after childbirth → After the emergency is over, a member of the research team will talk to you as soon as possible about the study and the process of deferred consent. If you consent that you are happy to take part, we will use information we have collected about your hospital stay.

4 weeks after treatment → We will contact you using your preferred method of communication (telephone, text or email) to follow-up completion of a questionnaire about your quality of life and your childbirth experience.

Your views and experiences → If you have opted in, yours and your birth partner's views and experiences of the study treatment and consent process will be explored after the emergency and before you leave the hospital, and again 4 weeks later.

What are the alternatives for treatment?

If you were not treated as part of COPE, you would have usually been given oxytocin first and then carboprost if the bleeding continued. Both are common treatments for PPH, but we do not know which is better.

What are the benefits and risks of taking part?

The results from the study will help doctors know what treatment is best to use first for PPH in the future.

The common side effects of both treatments are as follows:

Oxytocin	Carboprost
Headache	Diarrhoea
Nausea (feeling sick)	Vomiting
Vomiting	High temperature
Low blood pressure	Increased blood pressure
Water Retention	

For peer review only - http://bmjopen.bmj.com/site/about/guidelines.xhtml

Will my participation be kept confidential?

Yes. We will follow strict ethical and legal practice so that all information collected about you during the study is kept strictly confidential. With your permission, we will send a letter to your GP to let them know you are taking part and use your NHS number to access anonymised data about your hospital admissions.

Any information with your name on it (such as the consent form) that has to be transferred to the coordinating centre in Liverpool, will be sent securely and only accessed by people working on the study or working to ensure the study is being run correctly.

When you agree to take part in a research study, the information about your health and care may be provided to researchers running other research studies in this organisation and in other organisations. These organisations may be universities, NHS organisations or companies involved in health and care research in this country or abroad. Your information will only be used by organisations and researchers to conduct research in accordance with the UK Policy Framework for Health and Social Care Research.

This information will not identify you and will not be combined with other information in a way that could identify you. The information will only be used for the purpose of health and care research, and cannot be used to contact you or to affect your care. It will not be used to make decisions about future services available to you, such as insurance.

What if there is a problem?

If you have any concerns about this study, you should ask to speak with one of your research team who will do their best to answer your questions.

If you remain unhappy and wish to complain formally, you can do this through the NHS Complaints Procedure. Details can be obtained from your hospital.

Every care will be taken in the course of this research study. However, in the unlikely event that you are injured as a result of the managing organisation (University of Liverpool), compensation may be available and you may have to pay your related legal costs. Your hospital where you receive your treatment has a duty of care to you whether or not you agree to participate in the trial and the University of Liverpool accepts no liability for negligence on the part of your hospital's employees. If you are harmed and this is due to someone's negligence, then you may have grounds for a legal action for compensation against the NHS Trust where you are being treated, but you may have to pay for your legal costs.

What will happen to the results of the study?

Our results will guide clinical practice for PPH. We plan to present the results of the study at conferences and publish them in medical journals so that we can explain to the medical community what our research results have shown. Confidentiality will be ensured at all times and you will not be identified in any publication.

What are my electronic hospitalisation records?

The NHS organisations routinely collect information about your inpatient, outpatient and any Accident & Emergency hospital care. In England and Wales the data are referred to as 'Hospital Episode Statistics' or HES data, whilst in Scotland the data are referred to as 'Scottish Morbidity Records' or SMR data; these are regarded as a special category of information.

The study team will retrieve information from your electronic hospitalisation records held by NHS Digital (https://digital.nhs.uk) for patients in England; NHS Wales Informatics Service Information Services (http://www.infoandstats.wales.nhs.uk/) for patients in Wales; and the electronic Data Research and Innovation Service (http://www.isdscotland.org/Products-and-Services/EDRIS/) for patients in Scotland.

Researchers at Bangor University, who are part of the COPE study, want to use your HES and SMR data to

For peer review only - http://bmjopen.bmj.com/site/about/guidelines.xhtml

calculate the overall costs of care. These data will be collected for the period from three months prior to the start of the study and for the duration of the study. To retrieve your HES records, information to identify you including your name, date of birth, NHS (or CHI) Number and COPE Study Number, will be securely transferred by the COPE research team to each of the organisations listed above. Towards the end of the study, they will in turn, securely transfer your pseudo-anonymised HES or SMR records to Bangor University using an encrypted electronic transfer system. The data is referred to as 'pseudo-anonymised' data because whilst all your identifying details (name, date of birth, postcode, NHS (or CHI) Number) will have been removed, there is still a unique COPE Study Number present in the data should COPE researchers need to refer back to your identifying details and contact you for any reason.

Your electronic HES and SMR records will be stored on secure Bangor University computer servers which meet NHS data security standards. Researchers at Bangor University will not be able to access your identifying details and will not keep the pseudo-anonymised HES or SMR data longer than 12 months. It will then be returned to the University of Liverpool for long term storage.

Who is running the study?

The University of Liverpool is the sponsor for this study based in the United Kingdom. The University is responsible for managing this study whilst the University's Clinical Trials Research Centre (CTRC) runs it on a day-to-day basis. This study is funded by the National Institute for Health Research (NIHR)'s Health Technology Assessment (HTA) programme.

The study has been reviewed by the funder and has been approved by the Health Research Authority, National Research Ethics Service Committee (<MAIN REC>) and the Medicines and Healthcare products Regulatory Agency.

The University of Liverpool will be using information from you and your medical records in order to undertake this study and will act as the data controller for this study. This means that we are responsible for looking after your information and using it properly. The University of Liverpool will keep identifiable information about you for 25 years after the study has finished.

Your rights to access, change or move your information are limited, as we need to manage your information in specific ways in order for the research to be reliable and accurate. If you withdraw from the study, we will keep the information about you that we have already obtained. To safeguard your rights, we will use the minimum personally-identifiable information possible.

You can find out more about how we use your information at www.copestudy.uk

Your NHS hospital will collect information from you and your medical records for this research study in accordance with our instructions.

Your NHS hospital will use your name, NHS number and contact details to contact you about the research study, and make sure that relevant information about the study is recorded for your care, and to oversee the quality of the study. Individuals from the University of Liverpool and regulatory organisations may look at your medical and research records to check the accuracy of the research study. Your NHS hospital will pass these details to the University of Liverpool along with the information collected from you and your medical records. The only people in the University of Liverpool who will have access to information that identifies you will be people who need to contact you to in relation to the study or audit the data collection process. The people who analyse the information will not be able to identify you and will not be able to find out your name, NHS number or contact details.

Your NHS hospital will keep identifiable information about you from this study for 25 years after the study has finished.

For peer review only - http://bmjopen.bmj.com/site/about/guidelines.xhtml

Thank you for reading this information sheet.

Contacts for further information

If you would like more information or have any questions about the COPE study please talk to:

Principal Investigator: **<PI NAME>**
Research Midwife: **<RM NAME>**

Telephone: **<number>**
Or visit the website: www.copestudy.uk

If you wish to discuss the study with someone independent of the research team you can contact the local NHS Patient Advice and Liaison Service (PALS) on: <telephone number>

For peer review only - http://bmjopen.bmj.com/site/about/guidelines.xhtml

COPE
The Carboprost or Oxytocin Postpartum haemorrhage Effectiveness Study

NHS

Consent Form

To be completed by the Researcher:

Site Name:			
Participant Study Number			
Participant Initials:		Participant DOB:	/ /
NHS / CHI Number			
Postcode			

To be completed by the patient:

	Tick	Initial
**Once you have read and understood each statement please tick (✓) and initial.**<br>Example: I confirm that I have read and understand the Participant Information Sheet.	✓	JS
1. I have read and understood the information sheet for this study. I have had the opportunity to ask questions and have these answered satisfactorily.		
2. I understand that I have already received the study treatment for PPH.		
3. I understand that participation is voluntary and that I am free to withdraw from the study at any time, without giving a reason, and without my care or legal rights being affected.		
4. I understand that my data will be retained for a maximum of 25 years by the site and by the Clinical Trials Research Centre (CTRC), part of the University of Liverpool, and that they will be stored in a confidential manner.		
5. I understand that relevant written and electronically-recorded sections of my medical notes, records and any data collected during the study may be looked at by authorised individuals from the research team and those listed under ‘Who is running the study?’ section above, NHS Trust and Regulatory Authorities where it is relevant to my taking part in research. I give permission for these individuals to have access to these notes, my records and data.		
6. I understand that the data collected for this study may be used in a pseudo-anonymised form for related research.		
7. I give permission for a copy of this consent form which will include my name, date of birth and NHS number to be sent to the CTRC (where it will be kept in a secure location), to allow confirmation that my consent was given.		
8. I agree to my GP being informed of my participation in the study.		
9. I agree for my name, date of birth, NHS (or CHI) Number, COPE Study Number and a copy of this consent form to be shared with NHS Digital or their UK devolved equivalent so they can link this information to Hospital Episode Statistics (HES), or Scottish Morbidity Records (SMR) containing my details and provide members of the COPE study team working on the trial with HES and SMR data regarding my hospital attendances.		
10. I agree to take part in the above study.<br>My preferred method of communication for follow – up is (please tick as appropriate):<br>**Telephone** ☐ **Email** ☐ **Text** ☐		

For peer review only

For peer review only - http://bmjopen.bmj.com/site/about/guidelines.xhtml

Consent Form

To be completed by the Researcher:

Site Name:	
Participant Study Number	
Participant Initials:	Participant DOB: / /
NHS / CHI Number	
Postcode	

**Contact details:**	
**Telephone number:**	
**Email address:**	

Below are embedded study statements:

11. I agree to being contacted to take part in an embedded study interview.
12. I agree to completing an embedded study questionnaire.
13. I agree for the audio recording of my consent discussion with the COPE midwife/doctor to be used in the embedded study.

Below are optional study statements:

14. I agree that I may be contacted in the future in relation to this or other relates studies.

Your **full** name (please print):			
Your signature:		Date:	dd / mm / yyyy

To be completed by the Researcher:			
Researcher **full** name (please print):			
Researcher signature:		Date:	dd / mm / yyyy

To be completed by the Translator (if used):			
Translator **full** name (please print):			
Translator signature:		Date:	

For peer review only

For peer review only - http://bmjopen.bmj.com/site/about/guidelines.xhtml

COPE
The Carboprost or Oxytocin Postpartum haemorrhage Effectiveness Study

Trial number: ☐☐ ☐☐

COPE Study: postnatal questionnaire

Carboprost vs Oxytocin as the First Line Treatment of Primary Postpartum Haemorrhage.

The following questions are about the COPE study discussion and consent process that you/your partner took part in. We refer to people agreeing to take part in research as 'consenting'. We are interested in when you/ your partner were approached to discuss COPE and what you thought of the information you were given.

a. Date of birth: ☐☐/☐☐/☐☐☐☐

b. Today's date: ☐☐/☐☐/☐☐☐☐

c. First part of your postcode: ___ ___ ___ ___

d. Are you:

Male ☐ or female ☐

Patient (Mum) ☐ or Birth partner ☐ Please describe your relationship to the patient in the box below (e.g. husband, wife, partner, mother, friend)

☐

e. Have you/your partner had a postpartum haemorrhage in a previous pregnancy

Yes ☐ No ☐ I don't know ☐ Not applicable ☐

f. When did a doctor or midwife first speak to you about COPE? (Please tick all that apply)

1) During an antenatal appointment ☐

2) When I was bleeding before treatment was given ☐

3) After I had have been given treatment for bleeding ☐

4) I can't remember ☐

For peer review only - http://bmjopen.bmj.com/site/about/guidelines.xhtml

1. *Please indicate how strongly you agree or disagree with the following statements by placing a circle around the answer that best fits your opinion*

**Statements**	**Agree**	**Neither agree nor disagree**	**Disagree**
a. The doctor or midwife checked that it was a convenient time to discuss research before discussing COPE	1	2	3
b. The information I received about COPE was clear and straightforward to understand	1	2	3
c. I had enough opportunity to ask questions about COPE	1	2	3
d. I was satisfied with the consent process for COPE	1	2	3
e. It was difficult to take in the information I was given about COPE	1	2	3
f. It was difficult to make a decision about COPE	1	2	3
g. I made this decision	1	2	3
h. It was a joint decision (e.g. between patient and birth partner)	1	2	3
i. Someone took this decision away from me	1	2	3
j. I was not in control of this decision	1	2	3
k. The decision about the research was inappropriately influenced by others	1	2	3

If the answer to this question is ‘Agree’, please state who you think influenced the decision about the research:

____

For peer review only - http://bmjopen.bmj.com/site/about/guidelines.xhtml

2. *Did you/your partner consent for your participation in COPE?*

◯ Yes (Go to Question 3) ◯ No (Go to Question 4)

3. *What were your reasons for providing consent for your participation in COPE?*

Please tick all that apply and then circle your main reason (e.g. ✔)

a. To help me/my partner ☐

b. To help other women in the future ☐

c. I felt that medical studies like COPE are important ☐

d. Because I trusted the doctor or midwife who explained COPE ☐

e. The treatment had already been given to me/my partner ☐

f. The treatment worked ☐

g. I didn't feel comfortable saying no to the doctor or midwife who explained the study ☐

h. Other (Please state): ☐

____

4. *If you did not provide consent, please provide your reasons for deciding that you/your partner would not take part in COPE*
(If you do not wish to do so, please leave this space blank)

5. *Please tell us any comments or suggestions you have to improve the recruitment and consent process for COPE:*

We would like to thank you for taking the time to complete this questionnaire.

Please place the questionnaire in the envelope provided, seal it and give it back to the doctor or study midwife.

For peer review only - http://bmjopen.bmj.com/site/about/guidelines.xhtml

Version 1.0 19.01.2018
IRAS ID: 235254

COPE: Interview Topic Guide- Patient

Introduce self, thank participant for taking part and confirm happy to take part in an interview. **Remind participants of broad aims: We are interviewing women and their partners who have been asked to participation in the COPE study to find out experiences of women, their birth partners and practitioners involved in recruitment and consent in COPE. Findings from interviews will be used to inform how we conduct the rest of the trial.**

Section 1: Introduction, setting ground rules

Discuss the following issues:

- Review the nature and purpose of the research.
- No right or wrong answers, aim to understand experiences of recruitment and consent.
- Confidentiality, use of data.
- Explain the use of data recorder, transcription, use of pseudonym (invite to choose), use of verbatim quotes, will be taking field notes.
- Researcher aware that discussion might bring up difficult memories, explain can decline to answer any question or prompt; can ask to stop at any time if feels need to.
- Expected duration of interview.
- Check consent form signed
- Ask if any questions.

Bereaved mothers: I have some idea about your circumstances, if there is anything that you find difficult to talk about please don't feel that you have to. If you want to stop the interview at any point, then please let me know.

I will start with some questions about you if that's ok and then I will ask you about your experience of being invited to take part in the COPE study?

Check recorder working

Introduce and switch on tape recorder

Section 2: demographics

To start we have a couple of general questions about you if that's ok?

1. Could you please tell me your age?
2. Before your maternity leave were you in employed or unemployed? If employed, what was your occupation?

For peer review only - http://bmjopen.bmj.com/site/about/guidelines.xhtml

Version 1.0 19.01.2018
IRAS ID: 235254

3. What is the first part of post code?
4. What would you describe as your ethnic background?
5. How many children do you have?

Section 3: Antenatal awareness and COPE recruitment

1. **When did you first hear about the COPE study?**

2. **Were you provided with information about the COPE study during an antenatal visit?**

-IF NO

- **Did you see any leaflets or posters about the COPE Study during your pregnancy?** Explore location, views on the information; whether they requested further details, who from, what questions did you have, where they answered sufficiently.

Go to Section 4

IF YES

- Who discussed COPE with you (role)
- Could you tell me what they explained about the COPE study?
- Did they go through any of the potential risks or benefits of taking part in the study at that point? If yes, how did they describe these?
- Was there anything that you found: a) unclear b) surprising?
- Is there anything else that sticks out in your mind about the discussion?
- (*Prompt- if study aims not described by participant in response to the second point above*) This next question is not a test. Just so we can gage whether the trial is being explained clearly to women. Could you tell me what you understood the study was about?
- Do you have any suggestions about how this discussion could be improved in the future? If yes how?
- Could you tell me about any written information you were given about the COPE study? Explore whether they were given the information leaflet – short version and/or the full patient information sheet
- When did you receive this information? (Prompt: explore written and verbal and time point)
- Did you read the information leaflet/sheet? (Prompt: If they read the short information PIS leaflet or the full information PIS)
- What did you think about the information leaflet/sheet?
- Was there anything that you found: a) unclear? b) surprising?

For peer review only - http://bmjopen.bmj.com/site/about/guidelines.xhtml

Version 1.0 19.01.2018
IRAS ID: 235254

- Could the information leaflet be improved in any way? (Prompt: If so, how?)

Section 4: birth and PPH experience (for bereaved mothers introduce this section and restate that if there is anything that you find difficult to talk about please don't feel that you have to).

1. Please could you tell me about your labour and birth? (Explore: mode of delivery- vaginal birth, caesarean birth, emergency, planned, water birth, general anaesthetic etc.)
2. How did you feel during labour and birth?
3. After birth were you aware that you were losing a lot of blood.
   - *Explore what alerted you to the problem?*
   - *What were you told, by whom about the loss of blood.*
   - *Could you tell me what happened?, how long after birth?*
   - *How did you feel, any worries or fears, how did you manage your emotions?*
4. Can you remember if you were given any treatments to stop the bleeding? *(If can recall explore awareness of what drug, if it did stop the bleeding, other treatments given)*
5. (if applicable) Did you have a PPH with other births?

Section 5. COPE first approach (birth)

1. **Could you tell me at what point did someone first mention the COPE study? For example, did a midwife or doctor briefly speak to you about COPE before a treatment was given to help stop your bleeding?**

IF YES (e.g. verbal consent at time of PPH)

- Could you tell me a bit more about that? *Explore who and when,* did they speak to you or (partner name) or was this a joint discussion?
- Could you tell me how were you feeling at that point in time?
- Can you remember how you responded? Explore if consented, declined, did you ask any questions?
- Is there anything that sticks out in your mind about the discussion?
- Do you think that this was the best time to discuss the study with you?
- If not, when would you have preferred to have the discussion about COPE? *Explore views on seeking consent after the trial drugs have been given.*
- *Did someone come and speak to you a second time about COPE after your birth?*

IF NO (e.g. consent within 24 hours or can't remember)

- How would you have felt if the doctor or midwife had spoken to you at that point in time? (*Explore acceptability of seeking verbal consent during PPH, would you have been able to make a decision about taking part in COPE at that point in time?)*
- When do you remember a midwife or doctor first speaking to you about the COPE study? *(Explore who discussed COPE and whether person familiar to participant, whether it*

For peer review only - http://bmjopen.bmj.com/site/about/guidelines.xhtml

matters to the participant who approached her, timing of approach, whether this was an appropriate time and if timing was checked).

Section 6. COPE full recruitment conversation

1. **Could you tell me what they explained to you about the COPE study? (explore level of involvement in trial discussions) If already consented antenatally explore level of discussion- this could have just been a checking consent so questions below may be too detailed).**
2. **Did they go through any of the potential risks or benefits of taking part in the study at that point?** *If yes, how did they describe these?*
3. **Was there anything that you found: a) unclear b) surprising?**
4. **Is there anything else that sticks out in your mind about the discussion?**
5. **This next question is not a test. Just so we can gage whether the trial is being explained clearly to women. Could you tell me what you understood the study was about?**
6. **Do you have any suggestions about how this discussion could be improved in the future?** *If yes how?*
7. **Did you discuss the decision to be part of COPE with anyone else?** *Explore who, did their views influence your decision? Shared decision making*

Section 7. Written information (as above, if antenatally see if information given again)

1. **Could you tell me about any written information you were given about the COPE study?** *Explore whether they were given the information leaflet – short version and/or the full patient information sheet*
2. **When did you receive this information?** *(Prompt: explore written and verbal and time point)*
3. **Did you read the information leaflet/sheet?** (*Prompt:* If they read the short information PIS leaflet or the full information PIS)
4. **What did you think about the information leaflet/sheet?**
5. **Was there anything that you found: a) unclear? b) surprising?**
6. **Could the information leaflet be improved in any way?** *(Prompt: If so, how?)*

Section 8. Research without prior consent

Explain: Some women were not provided with COPE study information and invited to consent during an antenatal visit as they were not anticipated to be at high risk of a PPH. This meant that when they had a PPH they were included in the study without having a full discussion about the trial and invited to provide consent. This is research without prior consent, also known as deferred consent. Legislation allows research without prior consent in emergency situations as there is no time to have a discussion about the research and that actually having that discussion might delay vital treatment.

For peer review only - http://bmjopen.bmj.com/site/about/guidelines.xhtml

1. **What do you think of the use of research without prior content in emergency situations?**
2. **What do you think of the use of RWPC in the COPE study?**

ONLY IF RWPC approach used

3. **How long did you get to think about whether you wanted your information to be used in COPE?** *Do you think this was long enough?*
4. **Did you have the opportunity to ask any other questions about the study?**
5. **Did you ask any?** *What questions did you ask?, if not, why not*
6. **How long do you think people should be given to think about consenting to the use of their information in the trial?**
7. **In making the decision about your participation in COPE, what sort of things went through your mind?**
8. **Was there anything you found particularly helpful in making up your mind?**
9. **Was there anything you found unhelpful?**
10. **How hard was this decision?**
11. **Was there anything specific that influenced your decision**?
12. **Would you mind telling me what were your reasons for (providing consent/not providing consent)?**
13. **How do you feel about participating in this research?**
    - *Explore experiences of the research process e.g. recruitment, completing questionnaires, contact with research team*
    - *Ask how they feel about participating in this interview*

    Is there anything else you might want to add?

At the close of the interview briefly summarise the main points to confirm interpretation with the participant. Ask if they wish to expand any responses or add anything else to the discussion.

- *Ensure participant has contact details for the local research team should they wish to discuss any aspect of the study.*
- *Complete reflexive diary/field notes.*

For peer review only - http://bmjopen.bmj.com/site/about/guidelines.xhtml

COREQ (COnsolidated criteria for REporting Qualitative research) Checklist

A checklist of items that should be included in reports of qualitative research. You must report the page number in your manuscript where you consider each of the items listed in this checklist. If you have not included this information, either revise your manuscript accordingly before submitting or note N/A.

Topic	Item No.	Guide Questions/Description	Reported on Page No.
**Domain 1: Research team and reflexivity**			
Personal characteristics			
Interviewer/facilitator	1	Which author/s conducted the interview or focus group?	
Credentials	2	What were the researcher's credentials? E.g. PhD, MD	
Occupation	3	What was their occupation at the time of the study?	
Gender	4	Was the researcher male or female?	
Experience and training	5	What experience or training did the researcher have?	
Relationship with participants			
Relationship established	6	Was a relationship established prior to study commencement?	
Participant knowledge of the interviewer	7	What did the participants know about the researcher? e.g. personal goals, reasons for doing the research	
Interviewer characteristics	8	What characteristics were reported about the inter viewer/facilitator? e.g. Bias, assumptions, reasons and interests in the research topic	
**Domain 2: Study design**			
Theoretical framework			
Methodological orientation and Theory	9	What methodological orientation was stated to underpin the study? e.g. grounded theory, discourse analysis, ethnography, phenomenology, content analysis	
Participant selection			
Sampling	10	How were participants selected? e.g. purposive, convenience, consecutive, snowball	
Method of approach	11	How were participants approached? e.g. face-to-face, telephone, mail, email	
Sample size	12	How many participants were in the study?	
Non-participation	13	How many people refused to participate or dropped out? Reasons?	
Setting			
Setting of data collection	14	Where was the data collected? e.g. home, clinic, workplace	
Presence of non-participants	15	Was anyone else present besides the participants and researchers?	
Description of sample	16	What are the important characteristics of the sample? e.g. demographic data, date	
Data collection			
Interview guide	17	Were questions, prompts, guides provided by the authors? Was it pilot tested?	
Repeat interviews	18	Were repeat inter views carried out? If yes, how many?	
Audio/visual recording	19	Did the research use audio or visual recording to collect the data?	
Field notes	20	Were field notes made during and/or after the inter view or focus group?	
Duration	21	What was the duration of the inter views or focus group?	
Data saturation	22	Was data saturation discussed?	
Transcripts returned	23	Were transcripts returned to participants for comment and/or	

For peer review only

Topic	Item No.	Guide Questions/Description	Reported on Page No.
		correction?	
**Domain 3: analysis and findings**			
Data analysis			
Number of data coders	24	How many data coders coded the data?	
Description of the coding tree	25	Did authors provide a description of the coding tree?	
Derivation of themes	26	Were themes identified in advance or derived from the data?	
Software	27	What software, if applicable, was used to manage the data?	
Participant checking	28	Did participants provide feedback on the findings?	
Reporting			
Quotations presented	29	Were participant quotations presented to illustrate the themes/findings? Was each quotation identified? e.g. participant number	
Data and findings consistent	30	Was there consistency between the data presented and the findings?	
Clarity of major themes	31	Were major themes clearly presented in the findings?	
Clarity of minor themes	32	Is there a description of diverse cases or discussion of minor themes?	

Developed from: Tong A, Sainsbury P, Craig J. Consolidated criteria for reporting qualitative research (COREQ): a 32-item checklist for interviews and focus groups. *International Journal for Quality in Health Care*. 2007. Volume 19, Number 6: pp. 349 – 357

Once you have completed this checklist, please save a copy and upload it as part of your submission. DO NOT include this checklist as part of the main manuscript document. It must be uploaded as a separate file.

For peer review only

BMJ Open

Questioning approaches to consent in time critical obstetric trials: findings from a mixed methods study

Journal:	*BMJ Open*
Manuscript ID	bmjopen-2023-081874.R1
Article Type:	Original research
Date Submitted by the Author:	18-Dec-2023
Complete List of Authors:	Deja, Elizabeth; University of Liverpool, Department of Public Health, Policy and Systems<br>Weeks, Andrew; University of Liverpool, Department of Women's and Children's Health<br>Van Netten, Charlotte; University of Liverpool, Liverpool Clinical Trials Centre<br>Gamble, Carrol; University of Liverpool, Health Data Science<br>Meher, Shireen; Birmingham Women's Hospital<br>Gyte, Gillian; National Childbirth Trust,<br>Lavender, Tina; Liverpool School of Tropical Medicine Department of Clinical Sciences,<br>Woolfall, Kerry; University of Liverpool,
**Primary Subject Heading**:	Obstetrics and gynaecology
Secondary Subject Heading:	Qualitative research, Research methods, Ethics
Keywords:	Postpartum Women < Postpartum Period, OBSTETRICS, QUALITATIVE RESEARCH, Clinical Trial

SCHOLARONE™
Manuscripts

I, the Submitting Author has the right to grant and does grant on behalf of all authors of the Work (as defined in the below author licence), an exclusive licence and/or a non-exclusive licence for contributions from authors who are: i) UK Crown employees; ii) where BMJ has agreed a CC-BY licence shall apply, and/or iii) in accordance with the terms applicable for US Federal Government officers or employees acting as part of their official duties; on a worldwide, perpetual, irrevocable, royalty-free basis to BMJ Publishing Group Ltd ("BMJ") its licensees and where the relevant Journal is co-owned by BMJ to the co-owners of the Journal, to publish the Work in this journal and any other BMJ products and to exploit all rights, as set out in our licence.

The Submitting Author accepts and understands that any supply made under these terms is made by BMJ to the Submitting Author unless you are acting as an employee on behalf of your employer or a postgraduate student of an affiliated institution which is paying any applicable article publishing charge ("APC") for Open Access articles. Where the Submitting Author wishes to make the Work available on an Open Access basis (and intends to pay the relevant APC), the terms of reuse of such Open Access shall be governed by a Creative Commons licence – details of these licences and which Creative Commons licence will apply to this Work are set out in our licence referred to above.

Other than as permitted in any relevant BMJ Author's Self Archiving Policies, I confirm this Work has not been accepted for publication elsewhere, is not being considered for publication elsewhere and does not duplicate material already published. I confirm all authors consent to publication of this Work and authorise the granting of this licence.

For peer review only

Questioning approaches to consent in time critical obstetric trials: findings from a mixed methods study

Deja, E., Weeks, AD. Van Netten, C., Gamble, C., Meher, S., Gyte, G., Lavender, T and Woolfall, K.

Author details:

Dr Elizabeth Deja, PhD, Research Associate, University of Liverpool, Department of Public Health, Policy and Systems

Weeks, AD. MD FRCOG, Professor of International Maternal Health, Department of Women's and Children's Health, University of Liverpool and Liverpool Women's NHS Foundation Trust, members of Liverpool Health Partners.

Van Netten, C., Trial Manager, Liverpool Clinical Trials Centre, University of Liverpool.

Gamble, Carrol., Professor of Medical Statistics, Health Data Science, University of Liverpool.

Meher, S., MD, MRCOG, Consultant in Maternal Fetal Medicine, Birmingham Women's Hospital, Birmingham, UK

Gyte, G., BSc, MPhil. NCT PPI Consultant. NCT, Brunel House, 11 The Promenade, Clifton Down, Bristol. BS8 3NG

Lavender, T, PhD, Professor of Maternal and Newborn Health, Department of International Public Health, Liverpool School of Tropical Medicine, Pembroke Place, Liverpool L3 5QA.

Dr Woolfall, K., PhD, Reader in Health Research Methodology, University of Liverpool, Department of Public Health, Policy and Systems

Correspondence to Dr Elizabeth Deja, e.deja@Liverpool.ac.uk

Twitter

Kerry Woolfall @kerry_woolfall; Andrew Weeks @adweeks; Elizabeth Deja @BethDeja; Tina Lavender @DameTina1; COPE trial account @COPETrial; Carrol Gamble @Carrol_Gamble; LCTC @livuni_LCTC; IPHS @livuniIPH

Acknowledgements Special thanks to all the families who took part in the research and to our patient partners for their contribution to the design, conduct and study findings

For peer review only

Author contributions

KW led the study, which was embedded within the COPE Trial led by AW. ED, CG, GG, LT and AW were involved in the design of the study; or the acquisition, analysis or interpretation of data. ED collected the data. ED analysed the data with oversight from KW. ED and KW drafted the article and AW, LT, and CG critically revised it for important intellectual content. All authors gave approval for the version to be published. KW and ED agree to act as guarantors and to be accountable for all aspects of the work in ensuring that questions related to the accuracy or integrity of any part of the work are appropriately investigated and resolved.

Funding: The COPE Trial, including this embedded study, was funded by The National Institute for Health Research (NIHR) Health Technology Assessment (HTA) (Project number 16/16/06) and coordinated by the University of Liverpool.

Disclaimer: The views and opinions expressed herein are those of the authors and do not necessarily reflect those of the HTA Programme, NIHR, NHS or the Department of Health. The funders had no role in the collection, analysis and interpretation of data or in the decision to submit this article for publication.

Competing interests: NIHR, HTA Programme grant payments were made to the institutions of all authors to support the conduct of this study. ED and CVN employment roles were funded by the NIHR HTA programme grant payments made to their institutions.

Patient and public involvement: Patients and/or the public were involved in the design, conduct, reporting, or dissemination plans of this research. Refer to the Methods section for further details.

Ethics approval: This study involves human participants and was approved by West Midlands - Coventry & Warwickshire Research Ethics Committee (18/WM/0227)

Provenance and peer review: Not commissioned; Grant externally peer-reviewed.

Data availability statement: No data are available. Although data relevant to the study are included in the article, no raw data are available. The datasets generated and analysed for this study are not publicly available due to participants being from a small, specialised population, which creates ethical and privacy concerns about being too identifiable.

ORDID IDs

Elizabeth Deja https://orcid.org/0000-0002-3626-4927

Andrew Weeks https://orcid.org/0000-0002-1909-337X

Kerry Woolfall https://orcid.org/0000-0002-5726-5304

Gill Gyte https://orcid.org/0000-0003-4266-3585

Shireen Meher https://orcid.org/0000-0002-9866-7932

Carrol Gamble https://orcid.org/0000-0002-3021-1955

For peer review only

Conflicts of interest

ED None

KW None

SM None

CVN None

ADW Is the inventor of the PPH Butterfly a device to facilitate uterine compression for the first-line management of PPH. The patent is held by the University of Liverpool, but ADW would receive a share of any royalties if commercialised.

CG None

Key words

Postpartum haemorrhage, Consent, Trials, Labour, Mixed-methods

For peer review only

ABSTRACT

Objective: Trial legislation enables research to be conducted without prior consent (RWPC) in emergency situations, yet this approach has rarely been used in time-critical obstetric trials. This study explored views and experiences of antenatal recruitment and consent and RWPC in an emergency intrapartum randomised clinical trial.

Design: Embedded, Mixed methods study within a trial, involving questionnaires, recorded recruitment discussions, interviews and focus groups in the first year of trial recruitment (12/2020-01/2022).

Setting: COPE is a double-blind randomised controlled trial, comparing the effectiveness of Carboprost or Oxytocin as first-line treatment of postpartum haemorrhage.

Participants: Two hundred and eighty-six people (190 women/ 96 birth partners), linked to 198/380 (52.1%) COPE recruits participated in the embedded study. Of these, 272 completed a questionnaire (178 women/ 94 birth partners), 22 were interviewed (19 women/ 3 birth partners) and 16 consent discussions with 12 women were recorded. Twenty-seven staff took part in three focus groups and 9 staff were interviewed.

Results: Participants recommended that information about the study should be more accessible antenatally for those who wish to be informed. Most women and staff did not think it would be appropriate to seek consent during pregnancy or early labour as it may cause 'unnecessary panic' and lead to research waste, as most women would not become eligible. There was support for the use of RWPC as COPE interventions are used in standard clinical practice and viewed as low risk. Women who were approached about the trial whilst having a postpartum haemorrhage also supported RWPC as they could not recall research discussions.

Conclusions: Findings support the use of RWPC for time-critical interventions, and raise questions about the appropriateness of other commonly used consent pathways, including antenatal consent and verbal assent.

For peer review only

Strengths and limitations of this study

Strengths

- A comprehensive mixed methods design including questionnaires and in depth interviews from key stakeholders including trial participants, birth partners and staff; this allowed for triangulation of data and depth of insight.
- Insight was gained from women and birth partners who experienced informed consent through antenatal recruitment, as well as verbal assent during PPH and RWPC through emergency recruitment.
- Data were collected until there was thematic saturation and to the point of information power, which is the point when data is deemed to address the study aims; sample specificity, such as experience relevant to the study aims, and sample diversity.

Limitations

- The consent rate for the COPE trial was high (n= 363/380, 96%) which limited opportunities to gain insight into the views and experiences of women who declined to take part.
- Interviews were limited to participants who could speak English.

The original protocol for the study, see supplementary material 1.

For peer review only

INTRODUCTION

The Royal College of Obstetricians and Gynaecologists (RCOG) provides guidance on obtaining consent in time-critical perinatal research and recommends that the approach should vary according to the individual underlying risk of being eligible for participation (1). However, there is no evidence to support this strategy, or the quoted levels of risk, which means the design of clinical trial consent processes may not be in line with the expectations or priorities of women and stakeholders.

Recruiting women to intrapartum research studies is complex (2, 3). Several strategies have been used to balance the need to gain truly informed consent with the desire not to unnecessarily distress or over-medicalise a natural process (4). Predominantly, informed consent is sought from women before trial enrolment, however, this may not always be possible in intrapartum research for example if a woman completely lacks capacity (e.g. is unconscious). Legislation makes provisions for an alternative decision maker to be involved in enrolment decisions. This is usually a relative (e.g. legal representative) or doctor who is independent of the research. However, involving an alternative decision-maker requires time for information exchange and discussion and thereby this is not possible where there is an urgent need to treat within a medical emergency. In such urgent care situations, women or their representative may have very little time or capacity to consider study information and make an informed decision about participating in research. Research has shown staff may have ethical and legal concerns about enrolling women into a trial without written informed consent (5). Some studies include an antenatal consent process to help offset such concerns. For uncommon conditions or events, this means approaching potentially large numbers of women who may never become eligible. This can be costly and may overburden women with information or cause unnecessary worry for women who may never experience the trial condition (6, 7).

Houghton et al (2018) conducted a qualitative study exploring women's views and experiences of consent in an obstetric emergency in The WOMAN Trial, which explored treatments for postpartum haemorrhage (PPH) (8). The WOMAN trial included options for informed consent and to randomise women without their prior consent if a relative or doctor was not present to consent on their behalf. Regardless of the consent approach, and whether or not they consented or declined, women struggled to recall recruitment conversations and were often unable to differentiate between clinical and research discussions. No single consent pathway was preferred. Findings may have been limited by poor recall due to a stressful birth experience and interviews being conducted more than a year after trial recruitment took place.

Trials legislation enables research to be conducted without prior consent (RWPC, also known as deferred consent) when there is an urgent need to treat a patient for the purposes of the trial (9). This involves administering trial interventions to eligible patients without delay and then, once the emergency situation has passed, researchers seek consent for ongoing involvement in the study (10, 11). Although research has shown support for RWPC in paediatric and neonatal settings (12-15) there has been limited use of this approach in time critical obstetric trials. One pilot study (ACROBAT) (7) explored women's views and experiences of RWPC within a postpartum cluster trial and showed support for the approach in a small sample of women

COPE is a double-blind, randomised controlled trial (RCT) comparing the effectiveness of carboprost and oxytocin as first line treatments of PPH. As part of an embedded study, our research aimed to explore women, birth partner and clinician views on the various approaches to recruitment and consent to inform the ongoing RCT and future time critical obstetric trials.

For peer review only - http://bmjopen.bmj.com/site/about/guidelines.xhtml

Methods

Setting

The COPE Trial was designed with two consent processes: researchers could seek prospective antenatal consent for women at increased risk of PPH, or women could be recruited without prior consent during a PPH (see Figure 1). The latter method is unusual in a labour setting and required assessment of feasibility and patient acceptability.

[Insert Figure 1. COPE RCT consent process]

Study Design

We conducted an embedded mixed-methods study in all sites open during the first year of COPE Trial recruitment (12/2020- 01/2022). We used previous research (16-20) to develop participant information sheets (Supplementary material 2), questionnaires (Supplementary Material 3) and topic guides (Supplementary Material 4).

We collected questionnaires and conducted interviews with women and birth partners approached for participation in COPE. The questionnaire followed the same format as those used in similar studies (9, 11, 13) consisting of demographic questions, Likert scale questions from the Decision Making Control Instrument (14) and free text responses, taking on average five minutes to complete. Interview topic guides included questions on birth experiences, COPE information, approaches to recruitment, willingness to participate and views on trial acceptability

For clinical and research staff we undertook focus groups and interviews and recorded trial consent discussions with patients and birth partners (if applicable). The Staff focus group and interview topic guide explored experiences of recruitment and consent during the first year of recruitment, acceptability of the COPE trial, site training, screening, administering the COPE intervention, documentation, medication and logistics of running the trial.

Patient and public involvement

Design: Before finalizing the research protocol, the consent process went through extensive consultation with parents and public both in person and online with consumer groups.

Conduct: A trial consumer panel was formed, led by an experienced consumer representative and Trial Management Group member (GG), who provided critical input and represented the participants' views throughout the study

Dissemination: GG is an author on this paper. The paper will be disseminated to participants through the COPE online media. In addition an infographic of the findings will be sent directly to participants who requested a copy of the findings on their consent forms.

Women and birth partner recruitment and conduct

All women and their birth partners approached to take part in the COPE trial during its first year were eligible to take part in the embedded study if they spoke English. This included those who declined trial participation. When the COPE trial was broached by site staff, women and birth partners were informed of the embedded study and written consent was sought to complete a

For peer review only

questionnaire and/ or take part in a telephone/ online interview with the COPE researcher (ED) at a convenient time within approximately a month. Two pilot sites also sought consent to audio record consent discussions which took place on a hospital ward within 24 hours of randomisation.

Eleven English sites recruited to COPE during the first year. Staff at all sites provided women and birth partners with questionnaires to complete immediately following the consent discussion with pre-addressed envelopes to be returned to ED (PhD, female, Psychologist, Research Associate). ED contacted those who consented to interview in sequential order (by receipt of a consent form), stratifying by site, and role (women/ birth partner). Priority was given to those who had declined consent for the COPE trial and/ or agreed to audio recordings. Based on previous studies (21-23), we anticipated that 20-25 interviews would be needed to reach thematic saturation, where additional data does not lead to any new major themes identified during analysis. Researchers were also looking for high levels of 'information redundancy' (24-26) and information power, the point when data is deemed to address the study aims; sample specificity, such as experience relevant to the study aims, and sample diversity (24, 27).

Due to COVID-19 restrictions, all interviews took place over the telephone. Respondent validation was used to add unanticipated topics to the topic guide as interviewing and analysis progressed [16]. A £30 shopping voucher was given to participants after the interview to thank them for their time.

Staff recruitment and conduct

Initial findings from women and birth partner interviews and questionnaires were used to develop staff focus groups and interview topic guides (see supplementary material). Co-investigators at the first four open sites were contacted regarding the capacity to hold an online focus group using Microsoft Teams or Zoom. The research nurse and co- investigators disseminated invitations to all staff involved in the conduct of the trial, including Participant Information Sheets and consent forms to be completed before the focus group. Staff members that could not make the focus groups and/or were at other sites were invited to take part in an online interview.

Analysis

Digital audio recordings were transcribed verbatim by a professional transcription company (UK Transcription, Brighton) and anonymised. Qualitative analysis of interviews, focus groups, audio-recorded recruitment discussion data and open response questionnaire data were interpretive and iterative (28) utilising a reflexive thematic analysis approach (28, 29) (see table 1) . NVivo 12 software (QSR International Pty Ltd., Melbourne, Australia) was used to assist in the organisation and coding of data. Data from the parent and staff questionnaires were cleaned and entered into SPSS Version 24.0 (IBM Corp., Armonk, NY, US). Descriptive statistics are presented with percentages. ED and KW (PhD, female, social scientist, Reader) analysed and synthesised the data, drawing on the constant comparative approach (30, 31).

For peer review only

Table 1 Approach to qualitative data analysis

Phase	Description
**1. Familiarising with data**	ED read and re-read transcripts noting down initial ideas on themes.
**2. Generating initial codes**	Data-driven themes and concepts were identified by ED through line-by-line coding, discussions with KW and notes from phase 1. ED also developed the data-coding framework using *a priori* codes identified from the project aims, topic guides and past relevant experience of conducting trials methodology research
**3. Developing the coding framework**	KW coded 20% of the interview transcripts using and refining the initial coding frame.
**4. Defining and naming themes**	Following review by ED and KW coding frames were subsequently developed and ordered into themes (codes) within the *NVivo* Database. (Investigator triangulation)
**5. Completion of coding of transcripts**	ED completed coding interview transcripts in preparation for write-up.
**6. Quantitative data analysis**	Descriptive statistics were conducted on questionnaire and survey data.
**7. Data synthesis**	ED and KW synthesised qualitative and qualitative data drawing on a constant comparative approach. This involved identifying themes in each data set and reflecting on the study aims ensuring key findings and recommendations were relevant to the COPE trial design (catalytic validity).
**8. Stakeholder feedback**	ED presented initial findings and sought feedback from a variety of key stakeholders; Including 'how to COPE" research nurse working groups, The study advisory group and Management groups both of which have PPI representation (respondent validation).
**9. Producing the report**	Final discussion and development of selected themes occurred during the write-up phase.

Results

Participants

During the first year of COPE Trial recruitment, 382 women were screened for trial participation. A total of 366 individuals (n=260 women and 106 birth partners) gave consent to at least one part of the embedded study. Of these, 191 women and 96 birth partners provided data, linked to 199/ 380 (52.1%) COPE recruits at 9/11 (75%) sites (see Figure 2). Within these, 16 consent discussions with 12 women (mean 9 minutes per woman, range 4.1-19.6) on the emergency pathway were recorded, 272 questionnaires (178 women/ 94 birth partners) and 22 telephone interviews (19 women/ 3 birth partners) (mean of 31.6 minutes, SD 9.3, range 17 -49) were competed.

For peer review only

The women that completed the questionnaires had a mean age of 30 years old (range 18.4- 42.9 years) and 41/178 (23%) had experienced a PPH in a previous pregnancy. The birth partners that competed the questionnaire had a mean age of 31.9 years (range 21.9-64.9 years) and 17/94 (18%) had themselves or had a partner who had experienced a PPH in a previous pregnancy. The eleven sites opened at different times across the 12 months of data collection. Four sites were open during the first six months, with the others opening between months seven and twelve, two in the last month. To prevent over-representation, the initial two sites were closed to the embedded study after seven months as thematic saturation had been reached for both questionnaires and interviews. The other sites remained open to ensure information power was achieved.

The first four open sites took part in the staff component of the embedded study. A total of 36 staff self-identified as research midwives (n=10), midwives (n=4), anaesthetists (n=2), obstetricians (n=14), principal Investigators (n=2), ward managers (n=1) and research leads (n=3). Of these, 27 took part in three focus groups at two sites (mean 28 minutes, range 31-52) and 9 in interviews (mean 50 minutes, range 38-103).

[Instert Figure 2: Embeded study recruitment]

Of the women and birth partners who completed a questionnaire, most 162/272 (60.7 %) stated they first heard about COPE after treatment for PPH, whilst 42/ 272 (15.7%) indicated they heard about the trial when bleeding prior to treatment and 24/272 (9%) heard during an antenatal appointment. The remaining 15% 'could not remember' (29/272, 10.9%), 'other' (4/272, 1.5%) or did not respond to this question (11/272, 4%)

Antenatal recruitment - the importance of awareness

Women, birth partners and practitioners had mixed views on the use of antenatal recruitment to the COPE trial. All of the women and birth partners who were recruited antenatally (n=16) and/ or indicated in the questionnaire that they *"first heard about COPE during an antenatal appointment"* (questionnaire item) (n=8) stated they were *"satisfied with the consent process"* (questionnaire item, 24/24, 100%).

The majority of women who were recruited in the emergency pathway, as well as staff involved in recruitment, believed that antenatal COPE recruitment may: "*be a bit overwhelming and cause mams to panic unnecessarily*" (P05, Women, interview) for women who do not have a PPH and those that do:

> "No, definitely after. Because you don't need that extra worry on top. And isn't it only 5% of women that haemorrhage anyway? It's such a low percentage. Even if you're telling the high-risk women, they're already concerned. Yes, I would say afterwards."
>
> P12, Women, Interview

> "We didn't want to worry the women about PPH before the event because it might not happen to them. I think there was that fear of causing alarm about what might happen before it actually happens. You want her to look forward to the birth of her baby and not be worried about it. So, I think we were a little bit concerned that it might cause a bit of upset in the women unnecessarily"
>
> P01, Staff, Interview

Staff highlighted that alongside the potential emotional burden, antenatal recruitment was practically and conceptually difficult. A couple of the staff described how the definition of high risk for PPH varied between hospitals, with conflicting definitions considered to be confusing and a barrier for antenatal recruitment:

> "Some of our ladies that were classed as high-risk for a PPH weren't on your list as being high-risk for antenatal approaching. So, that caused a bit of confusion sometimes"
>
> P01, Staff, Interview

Practical issues to antenatal recruitment also included the COVID-19 pandemic restrictions limiting the: "*number of face-to-face consultations… it decreases our chances of meeting people*" (P01, Focus Group 1), as well as being "*really time-consuming*" (P05, Staff, Interview) and wasteful as recruitment may not lead to randomisation as: "*actually they did not have postpartum haemorrhage"* (P02, Staff, Focus group 1). They were also concerned with the possibility, that for those who did have a PPH, there may not be a COPE trained member of staff available to administer the drugs. These challenges were reflected in trial recruitment data (see Figure 2) with 22 antenatal consents resulting in just two randomisations.

Some staff were concerned about the possibility of accidentally recruiting a woman through the RWPC route who had previously declined consent antenatally. Despite processes to mitigate this risk being in place within the protocol, this possibility made some staff reluctant to recruit antenatally:

> "Because you could accidentally randomise someone who maybe they will be a decline, but that person maybe hasn't quite realised that, several months down the line, or weeks or whatever, when they then come to the Labour Ward."
>
> P06, Staff, interview

Both women and staff reflected that the main benefit of antenatal recruitment: "*is that it increases awareness for the women*" (P04, Staff, Interview). Women recruited through the emergency route felt that raising awareness of the COPE trial antenatally would be sufficient rather than consent being sought at that point in time. Both women and staff felt that this could be achieved through parent-facing advertising: "*whether it's just posters and things like that*" (P04, Staff, Focus group 2) or an "*information leaflet on Badger* [electronic notes]" (P09, Staff, Interview). As the information would provide: "*the opportunity to know about it..[..].. they could read up more into for themselves.*" (P17, Women, Interview) for those that wish to do so.

Assent discussion during PPH - situational incapacity

Some staff discussed broaching the COPE trial with women and/or birth partners during PPH when they felt it was appropriate, despite verbal assent not being specified within the COPE protocol. A total of 42/272 (15.4%) women and birth partners indicated in the questionnaire that 'a doctor or midwife first spoke to them about COPE while they were bleeding before treatment was given'. When describing their experience of labour and PPH, women's experiences varied greatly based on levels of blood loss, trauma, medical interventions, physical health, sedation and consciousness. All of these impacted their level of situational capacity and in turn their view on seeking verbal assent in COPE:

For peer review only

"I think I lost two and a half pints of blood. I just remember being very cold, very uncomfortable and frankly a little bit scared. Then I pretty much woke up four hours later and she [the baby] was already here."

P07, Women, Interview

"I was nervous beforehand, but the staff were great. They just put my mind at rest and, yes, it just felt like it went really quickly. Everything seemed to go well. Yes, it was a really calm and positive experience."

P15, Women, Interview

During interviews, the few women who recalled the COPE trial being briefly discussed during their PPH were largely indifferent to the research discussion. Due to the consequences of going through labour and having just given birth, they often stated they had not taken in any study information due to lack of capacity:

"Yes, I was fine with it. [being told about COPE] it was more beneficial that they came round after rather than explaining it fully in the [Caesarean] section room because in the theatre I wasn't thinking straight, so I couldn't tell you, I couldn't relay anything that they said in there.."

P13, Women, interview

In contrast, one woman who stated she wanted to be 'in control of her body' clearly recalled asking questions about the research and felt reassured to have all the information about what was going on, including information about COPE:

"For me personally, I ask questions because I like to feel a little bit in control of body, I like to know exactly what's happening. So, me asking questions and them being able to explain it there and then, that helped me come to terms with it, that helped calm me down really because I like to know step by step what is exactly happening."

P05, Women, interview

Women who did not hear about the COPE trial during their PPH were asked their views on seeking verbal assent at that point in time. Most felt that discussing research would be inappropriate, particularly if there was a lot of blood lost or in the context of a traumatic birth. As the quotations below illustrate, one woman felt that she was dying during her PPH. Most stated they would have been emotionally and physically unable to understand or retain research information and make an informed decision:

"There was one point in theatre when I thought, "I actually am going to die." But aside from that they were amazing. Everyone in the theatre was cheering me on, being like, "Push. I dare you. Push." It was a bit like being at a football match."

P20, Women, Interview

"In my opinion, labour is one of the most unplannable events that you can have. You can be completely low risk and end up requiring litres of blood. I think nobody is going to be retain that information at the time if they are haemorrhaging or having somebody place metal forceps in their vagina."

P18, Women, Interview

For peer review only

Not all women were aware that they had a PPH and some felt that broaching the trial to seek verbal assent could negatively affect women's birth experiences and cause panic by drawing attention to the bleeding:

> "I was completely unaware that I was having a bleed, whereas I think if they had made me aware in theatre that I was actually having a bleed and asked for my consent, I think I would have started panicking. I think that would have completely changed that really nice, calm memory I have got of giving birth. I think it is a good thing that they didn't ask for my consent right there and then, and then put me onto it."
>
> P15, Women, Interview

Seeking consent when the emergency situation has passed

The majority (351/373, 94%) of all COPE participants during the first year were recruited through the RWPC route. In our sample, 96.3% of women and birth partners indicated they were satisfied (n= 144/161) or indifferent (n=11/161) with 'the consent process for COPE' (Questionnaire Item). They described how it: "*made sense when I read the information why it was done that way*." (P21, women, interview) as they would not have been able to take in the information at the time and on the whole would prefer not to have it mentioned to them to before, or during labour. This support for RWPC appeared to be underpinned by their: "*trust in the people that are looking after you*" (P19, Women, Interview), that staff: "*wouldn't do it if it's going to put you at risk or anything, so it's fine*." (P01, Women Interview). RWPC in COPE was also viewed as acceptable because both treatment arms were: "*approved in practice in its own right*" (P18, Women, Interview).

Whilst all those interviewed were happy with the use of RWPC, interview and audio-recorded recruitment discussion data indicated that at the time of consent, commonly within 24 hours after birth, they were: "*shattered*" (P10, women, recruitment discussion recording), "*I'm really tired. I've just come over all tired*" (P12, Women, recruitment discussion recording); "*My whole body feels really weak and lethargic*." (P05, Women, recruitment discussion recording) and: "*in quite a lot of pain still*" (P16, Women, Interview). Alongside their physical condition: "*there was just too much happening*" (P10, Women, Interview) with people: "*just popping in to make sure you're okay* (P02, staff, recruitment discussion recording), women and baby checks, clinical care and looking after the baby. Consequently, they commented on how they had asked fewer questions than they might have done in another context. This was reflected in the audio recordings, which were short in length (mean 9 minutes, range 4.1-19.6) and predominantly consisted of the staff talking.

Despite the personal discomfort experienced at the point that the COPE trial was broached, during interviews women accurately recalled the purpose of the COPE trial and stated they had made an informed decision about taking part, with multiple opportunities to ask staff questions if they had wished. Recruitment discussion data indicated those who did ask questions were focused on clarifying details of their PPH: "*How much blood loss compared to…*? (P05, Women, recruitment discussion recording) and whether or not they had only received a placebo. During interviews, women reflected that their interactions with staff were clear and well-handled:

> "She came back and we still hadn't had a chance to read it. So, she left and then she came back later that day. She was great. It was all very clear. And she was very conscientious and understanding of the situation, just to give us space and time."

For peer review only

P12, Women, Interview

After discussing all the possible types of consent with women and birth partners during interviews, many concluded that if there had been an option between antenatal and RWPC recruitment for COPE, their preference was for the RWPC/ emergency route where: *"there was no consent given and they just gave it to me*" (P15, Women, Interview) due to the reasons outlined above.

Of the 6/161 (3.7%) who stated they were "not satisfied" with the use of RWPC in the questionnaire, four were birth partners and two were mothers. They used the open response section of the questionnaire to explain that they would have preferred to give consent in advance because: "*Seeking consent after the fact is disconcerting*" (P60, Women, Matched, Questionnaire) and: "*consent takes away some of the good will we have towards the programme*" (P101, Birth Partner, Questionnaire). One birth partner felt that: "*it didn't seem an emergency and more of a 'just in case' treatment* (P60, Birth partner, Matched, questionnaire) therefore not meeting the criteria for RWPC. One mother was not happy to have received an unnecessary intervention (the placebo): "*Tell people what is going in their bodies, don't get me wrong it helped but do not like the dummy needle*" (P199, Women, Questionnaire).

Staff described how they initially had concerns about the use of RWPC as their: "*knee-jerk response was the ethics of how can you give drugs to somebody without their consent?"* (P01, Staff, Interview). It was also considered: "*a really daunting approach for us because we really didn't know how women would take it*" (P04, Staff, Interview). However, experience with this approach appeared to change opinions about the acceptability of RWPC in this setting: "*I think the more we got through the trial, I think, the better we all have felt about it*." (P03, Staff, Focus group 3). Staff found RWPC acceptable in COPE because: *"the fact that the women are okay with it, puts to bed any worries that I had about how it would be perceived."* (P09, Staff, Interview). As the trial progressed, staff also concluded that giving the PPH treatment without prior consent was closer to standard practice as they do not ask for consent when administering PPH drugs in emergency situations:

> "If you have got a situation as a PPH, you don't go through all the different drugs. You don't say to a woman, 'We are giving this drug and this does this and that does that.' You don't go into great detail. You just say you are giving some medication and debrief them afterwards because, in that emergency situation, you haven't got time" (P08, Staff, Interview)

As also highlighted by women, Staff described how RWPC was acceptable in the COPE trial because it involved approved medications and actively treating the PPH: "*it is not a placebo medication, they are getting an active medication as well*" (P01, Staff, Focus group 1). Therefore: "*we not exposing the patient to unnecessary harm because of the trial*" (P01, Staff, Focus group 3) and: "*In essence, they are going to get their usual care*" (P11, Staff, Focus group 1).

The main issue staff experienced with RWPC was timing. They needed to obtain consent for the 24-hour main COPE trial questionnaire as close to that time point as possible. This was only If women were not busy and had the capacity to do so.

Trial screening logs indicated that the 17 women who declined consent were due to 'not receiving treatment for PPH' (n=2); women 'not wishing to take part in research' (n=7), no translation services being available (n=2), too much paper work' (n=1) and 'being too tired' (n=1). Or women providing verbal agreement, however, leaving hospital before written consent being obtained and then not returning the consent forms (n=4);'

For peer review only

Discussion

Establishing the most appropriate consent pathway for intrapartum research with women who may be vulnerable, have fluctuating capacity and need emergency treatment for PPH is ethically and practically challenging (2-4, 6-8). This mixed methods study provides insight into the experiences of women, birth partners and clinician and their views on approaches to recruitment and consent in the COPE trial, including the use of RWPC, which is novel in obstetric trials.

Our findings suggest that seeking informed consent antenatally was considered acceptable to those women and birth partners who experienced this pathway in COPE. Despite the COPE trial being designed in accordance with RCOG guidance (1), staff involved in recruitment, and women randomised to the trial through the emergency consent pathway questioned the ethics of consenting women who may never become eligible for the trial. They were concerned about causing unnecessary anxiety and information burden for women by asking them to consider something going wrong with their pregnancy, particularly for those that do not go on to have a PPH. This finding was also reported by Alvarez et al (32), who raised concerns about how a desire to not cause distress may lead to selection bias, with staff choosing who to approach. Women and birth partners in our study prioritised information provision, such as leaflets or posters, about the trial in antenatal settings over an informed consent process. Providing such information to women at increased risk of PPH would provide the opportunity for consideration by those who wish to do so (6) and therefore, in line with the bioethical principle of autonomy, protecting their right to make their own decision about research participation (33, 34). Staff were also concerned about the potential for research waste, a concern validated when the first 22 research consent discussions in COPE led to just two randomisations. After the first year of the COPE trial, taking into consideration the findings of the embedded study and recruitment rates, the antenatal consent pathway route was removed from the COPE protocol.

Despite verbal assent not being specified as part of the recruitment process in the COPE protocol, we found some clinicians did seek verbal assent for participation in the trial when women were having a PPH. The majority of women stated they were not in a position to have, or understand research discussions at that point in time. As reported by Houghton et al 2018 (8), recall of such recruitment discussions was poor. We found that due to situational incapacity, verbal assent at the time of PPH was only viewed as acceptable in a minority of cases when women are seeking out information and appeared to have capacity. However, assessing such capacity is extremely difficult (2, 3) and, as our data suggests, clinicians did not always correctly judge if a woman had the capacity to discuss research. Women in our study expressed a clear preference to not be approached about research at this time.

To our knowledge, this is the first study to explore experiences of RWPC in an obstetric RCT. RWPC was viewed by women and birth partners as being logical, acceptable and appropriate as COPE interventions were considered low-risk and time critical. This is in line with views of RWPC in paediatric emergency situations (13, 21, 23) and the ACROBAT Pilot trial (7). Staff had some initial apprehension about the use of RWPC. However, they were reassured by women's positive responses. Roper et al's (35) seven-step framework to enhance practitioner explanations and parental understandings of RWPC in paediatric emergency and critical care trials highlights the importance of explaining the condition before the consent discussion. This is particularly relevant to obstetric trials where women may have fluctuating, or no capacity, and therefore not be aware of what has happened during birth. Indeed, there were both women and birth partners in our study who were unaware that a PPH had occurred when COPE was broached by staff. In line with guidance on RWPC (36), we recommend that research staff should check the appropriate timing of the

For peer review only

research discussion with the bedside staff and ensure the patient is aware of their clinical condition and history during childbirth before explaining how they were entered into a clinical trial. Research staff should also explain the reasons why consent could not have been sought prospectively.

Strengths and limitations of this study

The study was strengthened by its mixed methods design including multiple data collection methods at various time points from all key stakeholders including birth partners. Data triangulation is considered to increase confidence in the findings from the research (37, 38). The study included all sites open within the first year which provided sample variance (75% of sites) and data from all sites that opened in the first 11 months of COPE retirement. Giving insight into views on consent processes from a large proportion of COPE participants and birth partners linked to half (52%) of the COPE recruits.

This study has several limitations. Firstly, we had restricted insight into the views and experiences of women who declined to take part in the COPE trial. However, 100% of women approached antenatally proved consent and 97.5% of women recruited via the emergency pathway provided consent, so there was limited opportunity to access the minority who declined. Screening logs indicated that reasons for declining were not related to the approach to recruitment and more about not wanting to take part in any research. An exception was one birth partner, whose questionnaire indicated they had declined due to not being satisfied with the RWPC process. Secondly, audio-recorded recruitment conversations were restricted to two sites. Although limited in quantity, the insight gained from the data we did obtain suggested they were often very brief conversations led by clinicians and corroborated the accounts given by both women and staff. These data further highlight the importance of assessing appropriate timing of RWPC discussion with women who have just had a traumatic childbirth. Thirdly, there were no interviews with antenatal consent pathway recruits because of delays in obtaining and/or processing consent due to staffing shortages during the initial phase of the COVID-19 pandemic. Questionnaire data linked to 14 COPE recruits 14/22 (63%) who experienced this pathway provided insight into their satisfaction with the recruitment process they experienced, but this did not provide the depth of understating that would be gained through an interview.

Conclusion

Recruiting women to intrapartum research studies is practically and ethically challenging. Our findings support the use of RWPC for time-critical, low-risk interventions and it raises questions about the appropriateness of other commonly used consent pathways, including seeking informed consent antenatally and verbal assent during an obstetric emergency.

For peer review only

Figures and supplementary Materials

Figure 1. COPE RCT consent process

Table 1: Approach to qualitative data analysis

Figure 2: Embedded study recruitment

SM 1 COPE Protocol

SM 2 Example Information Sheet and Consent Form - Emergency Patient

SM 3 Example Embedded Study Questionnaire- Postnatal

SM 4 Example Embedded Study topic guide - patient interview

References

1. RCOG. Obtaining Valid Consent to Participate in Perinatal Research Where Consent is Time Critical. Royal College of Obstetrics and Gynaecologists2016.
2. Vernon G, Alfirevic Z, Weeks A. Issues of informed consent for intrapartum trials: a suggested consent pathway from the experience of the Release trial [ISRCTN13204258]. Trials. 2006;7(1):13.
3. Russell AM, Shepherd V, Woolfall K, Young B, Gillies K, Volkmer A, et al. Complex and alternate consent pathways in clinical trials: methodological and ethical challenges encountered by underserved groups and a call to action. Trials. 2023;24(1):151.
4. Patel D, Nasir S, Elati A, Vernon G, Weeks A. Historical trends in the timing of informed consent for research into intrapartum complications. BJOG: An International Journal of Obstetrics & Gynaecology. 2012;119(3):361-5.
5. Lawton J, Hallowell N, Snowdon C, Norman J, Carruthers K, Denison F. Written versus verbal consent: a qualitative study of stakeholder views of consent procedures used at the time of recruitment into a peripartum trial conducted in an emergency setting. BMC Medical Ethics. 2017;18(1):1-13.
6. Lawton J, Snowdon C, Morrow S, Norman JE, Denison FC, Hallowell N. Recruiting and consenting into a peripartum trial in an emergency setting: a qualitative study of the experiences and views of women and healthcare professionals. Trials. 2016;17:195.
7. Sweeney L, Lanz D, Daru J, Rasijeff AM, Khanom F, Thomas A, et al. Deferred consent in emergency obstetric research: findings from qualitative interviews with women and recruiters in the ACROBAT pilot trial for severe postpartum haemorrhage. BMJ open. 2022;12(5):e054787.
8. Houghton G, Kingdon C, Dower M, Shakur-Still H, Alfirevic Z. What women think about consent to research at the time of an obstetric emergency: a qualitative study of the views of a cohort of World Maternal Antifibrinolytic Trial participants. BJOG: An International Journal of Obstetrics & Gynaecology. 2018;125(13):1744-53.
9. The Medicines for Human Use (Clinical Trials) Regulations, (2004).
10. Woolfall K, Frith L, Gamble C, Gilbert R, Mok Q, The CONNECT advisory group, et al. How parents and practitioners experience research without prior consent (deferred consent) for emergency research involving children with life threatening conditions: a mixed method study. BMJ Open *2015*;5(9):e008522
11. Woolfall K, Frith L, Dawson A, Gamble C, Lyttle MD, The CONNECT Advisory Group, et al. 15 minute consultation: an evidence-based approach to research without prior consent (deferred consent) in neonatal and paediatric critical care trials. Archives of disease in childhood - Education & practice edition. 2015;101(1):49-53.

For peer review only

12. Woolfall K, Young B, Frith L, Appleton R, Iyer A, Messahel S, et al. Doing challenging research studies in a patient-centred way: a qualitative study to inform a randomised controlled trial in the paediatric emergency care setting. BMJ Open. 2014;4(5).
13. Deja E, Peters MJ, Khan I, Mouncey PR, Agbeko R, Fenn B, et al. Establishing and augmenting views on the acceptability of a paediatric critical care randomised controlled trial (the FEVER trial): a mixed methods study. BMJ Open. 2021;11(3):e041952.
14. O'Hara CB, Canter RR, Mouncey PR, Carter A, Jones N, Nadel S, et al. A qualitative feasibility study to inform a randomised controlled trial of fluid bolus therapy in septic shock. Archives of Disease in Childhood. 2017.
15. Woolfall K, Frith L, Dawson A, Gamble C, group TCa, Young B. Research without prior consent (deferred consent) in trials investigating the emergency treatmemt of critically ill children: CONNECT study guidance. Liverpool: University of Liverpol; 2015 July 2015.
16. Molyneux S, Njue M, Boga M, Akello L, Olupot-Olupot P, Engoru C, et al. 'The Words Will Pass with the Blowing Wind': Staff and Parent Views of the Deferred Consent Process, with Prior Assent, Used in an Emergency Fluids Trial in Two African Hospitals. PLoS ONE. 2013;8(2):e54894.
17. O'Hara CB, Canter RR, Mouncey PR, Carter A, Jones N, Nadel S, et al. A qualitative feasibility study to inform a randomised controlled trial of fluid bolus therapy in septic shock. Arch Dis Child. 2017:archdischild-2016-312515.
18. Woolfall K, Frith L, Gamble C, Gilbert R, Mok Q, Young B. How parents and practitioners experience research without prior consent (deferred consent) for emergency research involving children with life threatening conditions: a mixed method study. BMJ Open. 2015;5(9):e008522.
19. Peters MJ, Woolfall K, Khan I, Deja E, Mouncey PR, Wulff J, et al. Different temperature thresholds for antipyretic intervention in critically ill children with fever due to infection: the FEVER feasibility RCT. Health Technology Assessment 2019;23(5):1 - 148.
20. Roper L, Lyttle MD, Gamble C, Humphreys A, Messahel S, Lee ED, et al. Planning for success: overcoming challenges to recruitment and conduct of an open-label emergency department–led paediatric trial. Emergency Medicine Journal. 2021;38(3):191-7.
21. Woolfall K, Frith L, Gamble C, Gilbert R, Mok Q, Young B, et al. How parents and practitioners experience research without prior consent (deferred consent) for emergency research involving children with life threatening conditions: a mixed method study. BMJ Open. 2015;5.
22. O'Hara CB, Canter RR, Mouncey PR, Carter A, Jones N, Nadel S, et al. A qualitative feasibility study to inform a randomised controlled trial of fluid bolus therapy in septic shock. Arch Dis Child. 2018;103(1):28-32.
23. Woolfall K FL, Gamble C, Young B. How experience makes a difference: practitioners' views on the use of deferred consent in paediatric and neonatal emergency care trials. BMC Med Ethics. 2013;14(45).
24. Saunders B, Sim J, Kingstone T, Baker S, Waterfield J, Bartlam B, et al. Saturation in qualitative research: exploring its conceptualization and operationalization. Quality & Quantity. 2018;52(4):1893-907.
25. Baker SE, Edwards R. How many qualitative interviews is enough? National Centre for Research Methods (NCRM): . 2012.
26. Mays N, Pope C. Assessing quality in qualitative research. Bmj. 2000;320(7226):50-2.
27. Malterud K, Siersma V, Guassora A. Sample Size in Qualitative Interview Studies. Qualitative Health Research. Sage Journals. 2016;26(13):1753-60.
28. Braun V, Clarke V. What can "thematic analysis''offer health and wellbeing researchers? International journal of qualitative studies on health and well-being. 2014;9:26152.
29. Byrne D. A worked example of Braun and Clarke's approach to reflexive thematic analysis. Quality & Quantity. 2021.
30. Glaser BG. The constant comparative method of qualitative analysis. Social Problems. 1965;12(4):436-45.
31. Stiles WB. Evaluating qualitative research. Evidence-Based Mental Health. 1999;2(4):99-101.

For peer review only

32. Alvarez M, Hotton EJ, Harding S, Ives J, Crofts JF, Wade J. Women's and midwives' views on the optimum process for informed consent for research in a feasibility study involving an intrapartum intervention: a qualitative study. Pilot and Feasibility Studies. 2023;9(1):98.
33. Entwistle VA, Carter SM, Cribb A, McCaffery K. Supporting Patient Autonomy: The Importance of Clinician-patient Relationships. Journal of General Internal Medicine. 2010;25(7):741-5.
34. Kihlbom U. Autonomy and negatively informed consent. Journal of Medical Ethics. 2008;34(3):146-9.
35. Roper L, Lyttle MD, Gamble C, Humphreys A, Messahel S, Lee ED, et al. Seven-step framework to enhance practitioner explanations and parental understandings of research without prior consent in paediatric emergency and critical care trials. Emergency Medicine Journal. 2021;38(3):198-204.
36. Woolfall K, Frith L, Dawson A, Gamble C, Lyttle MD, Young B. Fifteen-minute consultation: an evidence-based approach to research without prior consent (deferred consent) in neonatal and paediatric critical care trials. Archives of disease in childhood Education and practice edition. 2016;101(1):49-53.
37. Thurmond VA. The Point of Triangulation. Journal of Nursing Scholarship. 2001;33(3):253-8.
38. Flick U. Triangulation in qualitative research. A companion to qualitative research. 2004;3:178-83.

For peer review only

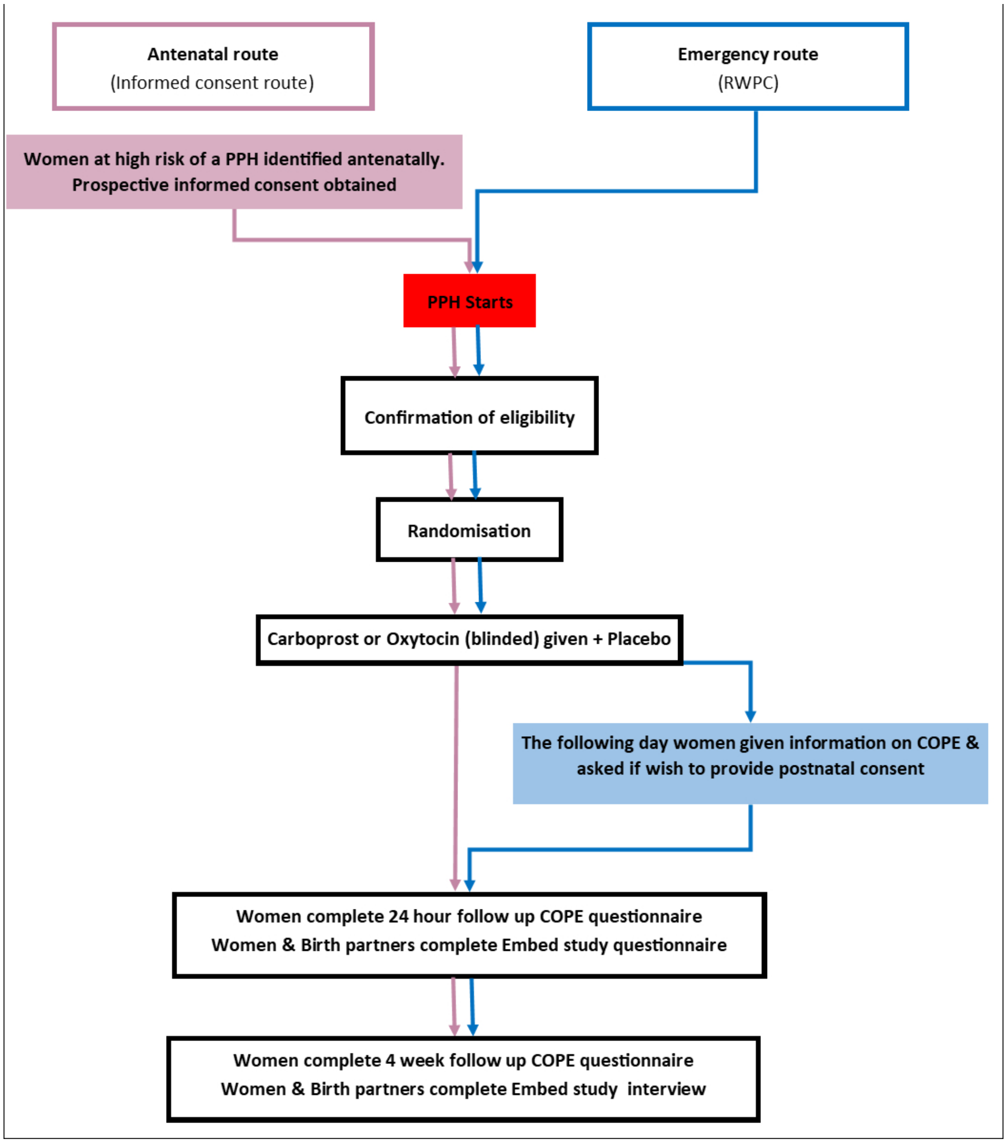

Figure 1. COPE RCT consent process

177x201mm (150 x 150 DPI)

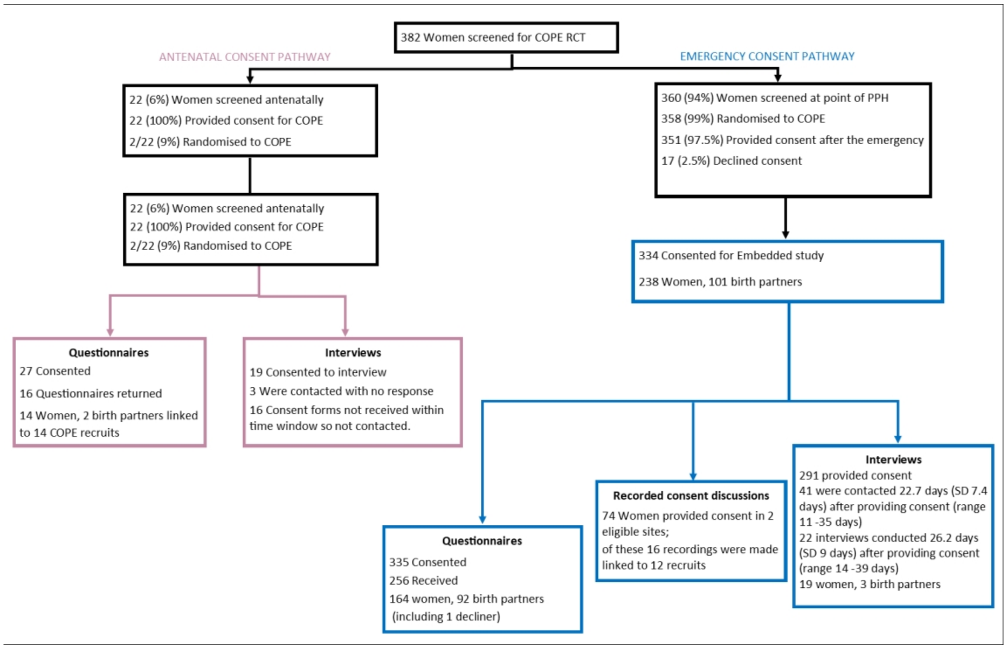

Figure 2: Embedded study recruitment

277x177mm (150 x 150 DPI)

COPE

The **C**arboprost or **O**xytocin **P**ostpartum haemorrhage **E**ffectiveness Study

Full explanatory title: Carboprost vs Oxytocin as the First Line Treatment of Primary Postpartum Haemorrhage. A Phase IV, double-blind, double-dummy, randomised controlled trial.

COPE Protocol v6.0 11/05/2022

Study Sponsor:

University of Liverpool Clinical Directorate,
Thompson Yates Building,
The Quadrangle,
Brownlow Hill,
Liverpool,
L3 5RB

EudraCT number: 2018-001829-11

CTA Reference Number: 04196/0052/001-0001

ISRCTN: 16416766

Research Ethics Ref: 18/WM/0227

Sponsor Ref: UoL001337

During the management of the COPE study, the Clinical Trials Research Centre (CTRC) and the Liverpool Cancer Trials Unit (LCTU) have merged to become the Liverpool Clinical Trials Centre (LCTC). The University of Liverpool is responsible for managing this study whilst the Liverpool Clinical Trials Centre (LCTC) runs it on a day-to-day basis. This study is funded by the National Institute for Health Research (NIHR) Health Technology Assessment (HTA) programme (16/16/06).

For peer review only

For peer review only - http://bmjopen.bmj.com/site/about/guidelines.xhtml

PROTOCOL APPROVAL

I, the undersigned, hereby approve this clinical study protocol:

Authorised by Chief Investigator:

Signature: ______________________________ **Date:** ____________

Prof Andrew Weeks
Professor of International Maternal Health
Sanyu Research Unit, Department of Women's and Children's Health
University of Liverpool
c/o University Department, First Floor
Liverpool Women's Hospital
Crown Street
Liverpool, L8 7SS

For peer review only

For peer review only - http://bmjopen.bmj.com/site/about/guidelines.xhtml

PROTOCOL APPROVAL

I, the undersigned, hereby approve this clinical study protocol:

Authorised on behalf of Sponsor:

Signature: ________________________________ **Date:** ____________

Karen Wilding
Senior Clinical Research Governance Manager
University of Liverpool Clinical Directorate
Thompson Yates Building
The Quadrangle, Brownlow Hill,
Liverpool
L3 5RB

For peer review only

PROTOCOL APPROVAL

I, the undersigned, hereby approve this clinical study protocol:

Authorised by Lead Statistician:

Signature: ____ **Date:** ______________

Prof Carrol Gamble
LCTC Director
Liverpool Clinical Trials Centre
University of Liverpool
2nd Floor, Institute in the Park
Alder Hey Children's NHS Foundation Trust
Liverpool, L12 2AP

For peer review only

For peer review only - http://bmjopen.bmj.com/site/about/guidelines.xhtml

General Information

This document describes the COPE trial including detailed information about procedures and recruitment. The protocol should not be used as an aide-memoir or guide for the treatment of other patients; every care was taken in its drafting, but corrections or amendments may be necessary. These will be circulated to the registered investigators in the trial, but centres entering patients for the first time are advised to contact the coordinating centre (Liverpool Clinical Trials Centre, University of Liverpool) to confirm they have the most up to date version. Clinical questions relating to this trial should be referred to the Chief Investigator, Professor Andrew Weeks, via the LCTC.

This protocol defines the participant characteristics required for study entry and the schedule of treatment and follow-up. Participant recruitment will be undertaken in compliance with this document and applicable regulatory and governance requirements and waivers to authorise non-compliance are not permitted.

Incidence of protocol non-compliance, whether reported prospectively (e.g. where a treatment cannot be administered on a scheduled date as a result of public holidays) or retrospectively noted (e.g. as a result of central monitoring) are recorded as protocol deviations, the incidence of which are monitored and reported to trial oversight committees.

The template content structure is consistent with the SPIRIT (22) and has regard for the Health Research Authority guidance (1). Regulatory and ethical compliance information is located in section 12.

Relationship Statements

Roles and responsibilities are fully described in section 15.

The sponsor name is University of Liverpool who will formally delegate specific sponsoring roles to the Chief Investigator and Clinical Trials Unit, but remains legally responsible for the trial.

Clinical Trials Unit (CTU): LCTC at the University of Liverpool in collaboration with the chief investigator, Professor Andrew Weeks, will have overall management responsibility for the trial from a CTU perspective and will be responsible for the co-ordination of centres.

LCTC as part of the Liverpool Clinical Trials Collaborative has achieved full registration by the UK Clinical Research Collaboration (www.ukcrc.org) as their standards and systems were assessed by an international review panel as reaching the highest quality. LCTC has a diverse trial portfolio underpinned by methodological rigour, a GCP compliant data management system, and core standard operating procedures.

For peer review only - http://bmjopen.bmj.com/site/about/guidelines.xhtml

CONTACT DETAILS

Institutions

**Sponsor:**	**Trial Management, Monitoring and Analysis:**
The University of Liverpool<br>Clinical Directorate<br>Thompson Yates Building<br>The Quadrangle, Brownlow Hill,<br>Liverpool<br>L3 5RB<br><br>Tel: 00 44 (0) 7717 863747<br>Email: sponsor@liverpool.ac.uk	Liverpool Clinical Trials Centre<br>University of Liverpool<br>2nd Floor, Institute in the Park<br>Alder Hey Children's NHS Foundation Trust<br>Eaton Road<br>Liverpool<br>L12 2AP<br><br>Tel: +44(0)151 795 8760<br>E-mail: cope@liverpool.ac.uk

For peer review only

For peer review only - http://bmjopen.bmj.com/site/about/guidelines.xhtml

CONTACT DETAILS

Individuals

**Individual Authorised to Sign the Protocol and Protocol Amendments on behalf of the Sponsor:**	**Chief Investigator (CI):**
Karen Wilding<br>Senior Clinical Research Governance Manager<br>The University of Liverpool Clinical Directorate<br>Thompson Yates Building<br>The Quadrangle, Brownlow Hill,<br>Liverpool<br>L3 5RB<br><br>Tel: 00 44 (0) 7717 863747T: +44(0)151 794 8373<br>E-mail: sponsor@liverpool.ac.uk	Professor Andrew Weeks<br>Professor of International Maternal Health<br>Sanyu Research Unit, Department of Women's and Children's Health<br>University of Liverpool<br>c/o University Department, First Floor<br>Liverpool Women's Hospital<br>Crown Street<br>Liverpool, L8 7SS<br><br>Tel: +44(0)151 7959578<br>Fax: +44(0)151 7959553<br>E-mail: aweeks@liverpool.ac.uk
**Medical Expert who will Advise on Protocol Related Clinical Queries (If other than CI):**	**Medical Expert who will Evaluate SAE Reports (If other than CI):**
Dr Shireen Meher<br>Consultant in Maternal Fetal Medicine<br>Birmingham Women's & Children's NHS Foundation Trust<br>Birmingham Women's Hospital<br>Mindelsohn Way<br>Edgbaston<br>Birmingham, B15 2TG<br><br>Tel: 07734694660<br>E-mail: smeher@liverpool.ac.uk	Dr Dimitrios Siassakos<br>Honorary Consultant in Obstetrics<br>UCL EGA Institute for Women's Health<br>University College Hospital<br>Medical School Building<br>74 Huntley Street<br>London WC1E 6AU<br><br>Tel: 07930 624 894<br>E-mail: jsiasakos@me.com
**Medical Expert who will Evaluate SAE Reports (If other than CI or cross cover):**	**Statistical Team Lead:**
Mr Kim Hinshaw<br>Consultant Obstetrician & Gynaecologist<br>Obstetrics & Gynaecology<br>City Hospitals Sunderland NHS Foundation Trust<br>Sunderland Royal Hospital<br>Kayll Road<br>Sunderland, SR4 7TP<br><br>Tel: 0191 5410051<br>Fax: 0191 5699792<br>E-mail: kim.hinshaw@chsft.nhs.uk	Professor Carrol Gamble<br>Head of Biostatistics, LCTC Director<br>Liverpool Clinical Trials Centre<br>University of Liverpool<br>2nd Floor, Institute in the Park<br>Alder Hey Children's NHS Foundation Trust<br>Liverpool<br>L12 2AP<br><br>Tel: 0151 794 9759<br>E-mail: c.gamble@liverpool.ac.uk

For pe

**Embedded study:**	**Embedded Study Lead and Consent Lead:**
Professor Dame Tina Lavender<br>Professor of Maternal and Newborn Health<br>Department of International Public Health<br>Liverpool School of Tropical Medicine<br>Pembroke Place<br>Liverpool, L3 5QA<br><br>E-mail: Tina.Lavender@lstmed.ac.uk	Kerry Woolfall, PhD, Dr, Psychological Sciences<br>Senior Lecturer<br>MRC North West Hub for Trials Methodology Research<br>Institute of Psychology, Health and Society<br>Block B, Room B112, 1st Floor Waterhouse Building<br>Liverpool, L69 3GL<br><br>Tel: 0151 794 4634<br>E-mail: woolfall@liverpool.ac.uk
**Lead for Economic Evaluation:**	
Prof Dyfrig Hughes<br>Professor of Pharmacoeconomics<br>Centre for Health Economics and Medicines Evaluation<br>Ardudwy, Normal Site,<br>Bangor University<br>Holyhead Road<br>Bangor, LL57 2PZ<br><br>Tel: 01248 382950<br>E-mail: d.a.hughes@bangor.ac.uk	

Additional Contacts:

The contact details of other individuals involved in the trial are detailed in documents supplementary to the protocol and stored in the Trial Master File (TMF):

Contact	Document Title
Trial Management Group (TMG)<br>Trial Steering Committee (TSC)<br>Independent Data and Safety Monitoring Committee (IDSMC)	COPE Trial Oversight Committee Membership
Principal Investigators (PIs)	COPE Participating Centres

For peer review only - http://bmjopen.bmj.com/site/about/guidelines.xhtml

TABLE OF CONTENTS

PROTOCOL APPROVAL 2
CONTACT DETAILS 6
TABLE OF CONTENTS 9
GLOSSARY 12
1 PROTOCOL SUMMARY 13
1.1 Schematic of Study Design 15
2 INTRODUCTION 16
2.1 Background 16
2.2 Rationale 16
2.3 Risk and Benefits 17
2.4 Objectives 17
2.4.1 Primary Objective 17
2.4.2 Secondary Objective(s) 17
2.5 Outcome measures 17
2.5.1 Primary Outcome 17
2.5.2 Secondary Outcome 18
3 TRIAL DESIGN 19
4 STUDY SETTING AND SELECTION OF CENTRES / CLINICIANS 20
5 STUDY POPULATION 21
5.1 Inclusion Criteria 21
5.2 Exclusion Criteria 21
5.3 Co-enrolment Guidelines 21
6 SCREENING, RECRUITMENT AND RANDOMISATION 22
6.1 Screening 22
6.2 Recruitment 22
6.3 Consent Procedures 22
6.3.1 Information provision and postnatal consent 22
6.3.2 Principles of informed consent 23
6.3.3 Discharge/transfer to another hospital prior to postnatal consent 24
6.3.4 Maternal death prior to postnatal consent 26
6.3.5 Neonatal death prior to postnatal consent 26
6.4 Randomisation Procedures 26
6.5 Who is Blinded to Allocations 27
7 PARTICIPANT TIMELINE, ASSESSMENTS AND PROCEDURES 28
7.1 Schedule for Follow-up 28
7.2 Procedures for Assessing Efficacy 29
7.3 Procedures for Assessing Safety 29
7.4 Other Assessments 29
7.4.1 Childbirth Experience Questionnaire (CEQ) 29
7.4.2 Health Economics assessments 29
7.5 Patient Transfer and Withdrawal 30
7.5.1 Discontinuation 30
7.5.2 Withdrawal of Consent 30
7.5.3 Participant Transfers 30
7.6 Loss to Follow-up 31

For peer review only

For peer review only - http://bmjopen.bmj.com/site/about/guidelines.xhtml

7.7 Trial Closure 31
8 EMBEDDED MIXED METHODS RESEARCH 32
8.1 Design 32
8.2 Selection of participants 32
8.3 Enrolment and procedure 33
8.3.1 Part A: Audio-recordings 33
8.3.2 Part B: Questionnaires 34
8.3.3 Part C: Interviews with women and their birth partner 34
8.3.4 Part D: Focus groups and/or interviews with the COPE practitioners 35
9 TRIAL TREATMENTS 36
9.1 Introduction 36
9.2 Formulation, Packaging, Labelling, Storage and Stability 36
9.2.1 Formulation 36
9.2.2 Packaging and Labelling 36
9.2.3 Storage and Stability 37
9.3 Distribution, Dispensing, Dosage and Administration of Study Treatment/s 37
9.3.1 Distribution and Dispensing 37
9.3.2 Dosage and Administration 37
9.4 Overdose 39
9.5 Delivery and Accountability of IMP at Trial Sites 39
9.5.1 Delivery 39
9.5.2 Accountability Procedures for Study Treatment/s 39
9.5.3 Expired and unused IMP stock 39
9.6 Concomitant Medications/Treatments 40
9.6.1 Medications Permitted/Not Permitted/ Precautions Required 40
9.6.2 Data on Concomitant Medication 40
9.7 Unblinding 40
10 SAFETY REPORTING 41
10.1 Time Period for Safety Reporting 41
10.2 Reference Safety Information 41
10.3 Flowchart of Adverse Events Reporting Requirements 42
10.4 Terms and Definitions 42
10.5 Notification of deaths 44
10.6 Severity / Grading of Adverse Events 44
10.7 Relationship to Trial Treatment 44
10.8 Expectedness 45
10.9 Follow-up After Adverse Events 45
10.10 Reporting Procedures 45
10.10.1 Non serious AEs 45
10.10.2 Non serious ARs 46
10.10.3 SAEs/ SARs/ SUSARs 46
10.10.4 Maintenance of Blinding 47
10.11 Responsibilities – LCTC 47
10.12 Safety reports 48
10.13 Urgent Safety Measures 48
10.14 Out-of-hours Medical Cover 48
11 STATISTICAL CONSIDERATIONS 49
11.1 Introduction 49
11.2 Method of Randomisation 49
11.3 Sample Size calculation 49
11.3.1 Feasibility (attaining recruitment targets) 50
11.4 Interim Monitoring and Analyses 50

For peer review only - http://bmjopen.bmj.com/site/about/guidelines.xhtml

11.5 Analysis Plan 50
11.5.1 Health Economic Analysis 50
11.5.2 Qualitative data analysis 51

12 REGULATORY AND ETHICAL APPROVALS 52
12.1 Statement of Compliance 52
12.2 Regulatory Approval 52
12.3 Ethical Approval 52
12.3.1 Ethical Considerations 52
12.4 Health Research Authority (HRA) Approval 53
12.4.1 Capacity and capability assessment 53
12.5 Protocol Deviation and Serious Breaches 53
12.6 Study Discontinuation 53

13 DATA MANAGEMENT AND TRIAL MONITORING 54
13.1 Source Documents 54
13.2 Data Capture Methods 54
13.3 Monitoring 55
13.3.1 Central Monitoring 55
13.3.2 Clinical Site Monitoring 55
13.4 Confidentiality 55
13.4.1 Hospital Episode Statistics (HES) 56
13.5 Quality Assurance and Control 56
13.6 Records Retention 56

14 INDEMNITY 58

15 ROLES AND RESPONSIBILITIES 59
15.1 Role of Study Sponsor and Study Funder 59
15.2 Funding and Support in Kind 59
15.2.1 NHS Research costs 59
15.2.2 NHS Support costs 60
15.2.3 Treatment costs 60
15.3 Protocol Contributors 60
15.4 Trial Committees 61
15.4.1 Trial Management Group (TMG) 61
15.4.2 Trial Steering Committee (TSC) 61
15.4.3 Independent Data and Safety Monitoring Committee (IDSMC) 61

16 PUBLICATION AND DISSEMINATION 62
16.1 Publication Policy 62
16.1.1 Authorship 62
16.2 Dissemination to Key Stakeholders 62
16.3 Data Sharing 62

17 CHRONOLOGY OF PROTOCOL AMENDMENTS 63
17.1 Version 1.0 (10/07/2018) 63
17.2 Version 2.0 (10/09/2018) 63
17.3 Version 3.0 (01/05/2019) 63
17.4 Version 4.0 (13/07/2020) 65
17.5 Version 5.0 (14/04/2021) 68
17.6 Version 6.0 (11/05/2022) 69

18 REFERENCES 71

Appendix 1. COPE Outcomes 73

For peer review only

GLOSSARY

AE	Adverse Event
AR	Adverse Reaction
CI	Chief Investigator
COS	Core outcome set
CRF	Case Report Form
CTIMP	Clinical Trial of an Investigational Medicinal Product
CTU	Clinical Trials Unit
EUDRACT	European Clinical Trials Database
GP	General Practitioner
HRA	Health Research Authority
IDSMC	Independent Data and Safety and Monitoring Committee
IMP	Investigational Medicinal Product
LCTC	Liverpool Clinical Trials Centre
MHRA	Medicines and Health Care Products Regulatory Agency
NRES	National Research Ethics Service
NIHR CRN	National Institute for Health Research Clinical Research Network
PI	Principal Investigator
QALY	Quality Adjusted Life Years
RCOG	Royal College of Obstetricians and Gynaecologists
R&D	Research & Development
REC	Research Ethics Committee
RM	Research Midwife (Registered)
RSI	Reference Safety Information
RSO	Research Support Office
SAE	Serious Adverse Event
SAR	Serious Adverse Reaction
SDV	Source Data Verification
SMPC	Summary of Product Characteristics
SMR	Scottish Morbidity Records
SOP	Standard Operating Procedure
SSAR	Suspected Serious Adverse Reaction
SUSAR	Suspected Unexpected Serious Adverse Reaction
TMG	Trial Management Group
TSC	Trial Steering Committee
UAR	Unexpected Adverse Reaction
WHO	World Health Organisation

For peer review only

1 PROTOCOL SUMMARY

**Full Title:**	Carboprost vs Oxytocin as the first line treatment of primary postpartum haemorrhage. A Phase IV, double-blind, double-dummy, randomised controlled trial.
**Acronym:**	COPE
**Phase:**	IV
**Target Condition:**	Primary Postpartum haemorrhage (PPH)
**Sample size**	3,948 participants
**Main Inclusion Criteria:**	• ≥ 16 years of age<br>• Requirement for medical treatment for primary PPH
**Main Exclusion Criteria:**	• Known to have opted out of participation antenatally<br>• Known oxytocin or carboprost hypersensitivity<br>• Known active cardiac or pulmonary disease<br>• Known to have previously been treated as part of COPE<br>• Has already received carboprost prophylactically for postpartum haemorrhage<br>• Has already received uterotonic drug treatment for postpartum haemorrhage (this does not include PPH prophylaxis)<br>• Stillbirth
**Study Centres and Distribution:**	NHS hospital maternal units in the UK
**Patient Study Duration:**	Duration per participant: 4 weeks
**Overall Study duration:**	48 months
**Agent/ Intervention:**	**Intervention:** Carboprost 250 micrograms by deep intramuscular injection and 1 ml placebo by slow intravenous injection over 2 minutes<br><br>**Control:** Oxytocin (5 international units (IU) for caesarean section or 10 IU for vaginal delivery) by slow intravenous injection over 2 minutes and 1 ml placebo by deep intramuscular injection
**Primary objective:**	To compare carboprost with oxytocin as initial treatments for women with clinically diagnosed PPH after giving birth in UK hospitals.

For peer review only

**Secondary objective:**	1. To assess the relative cost-effectiveness of the use of carboprost and oxytocin as initial treatments for women with clinically diagnosed PPH.<br>2. To explore the views of participants and their carers about their experiences of the two treatments and the consent process.
**Primary outcome measures:**	Blood transfusion defined as "Any red blood cell transfusion or cell salvage of ≥300mls commenced any time between randomisation and 48 hours after randomisation (or hospital discharge if earlier than 48 hrs)"
**Secondary outcome measures:**	1. Volume of blood transfusion<br>2. Use of a further uterotonic drug<br>3. Composite outcome of any organ dysfunction based on WHO near-miss approach for maternal health (2)<br>4. Hysterectomy<br>5. Blood loss<br>6. Blood loss ≥ 1000 mls<br>7. Haemoglobin<br>8. Shock<br>9. Maternal death<br>10. Non pharmacological approach to treat or investigate bleeding<br>11. Manual removal of placenta<br>12. Adverse reactions of particular interest (as listed in section 10.1)<br>13. 'Skin to skin' care with baby within the first hour after birth<br>14. Separation from new-born in first hour after birth<br>15. Breastfeeding<br>16. Childbirth Experience Questionnaire (CEQ)<br>17. Resource use
**Embedded Mixed Methods Research**	For at least the first 9 months of recruitment, we will conduct qualitative research to explore the views and experiences of women recruited to the trial, their partners, and practitioners involved in recruitment and consent in COPE.

For peer review only - http://bmjopen.bmj.com/site/about/guidelines.xhtml

1.1 Schematic of Study Design

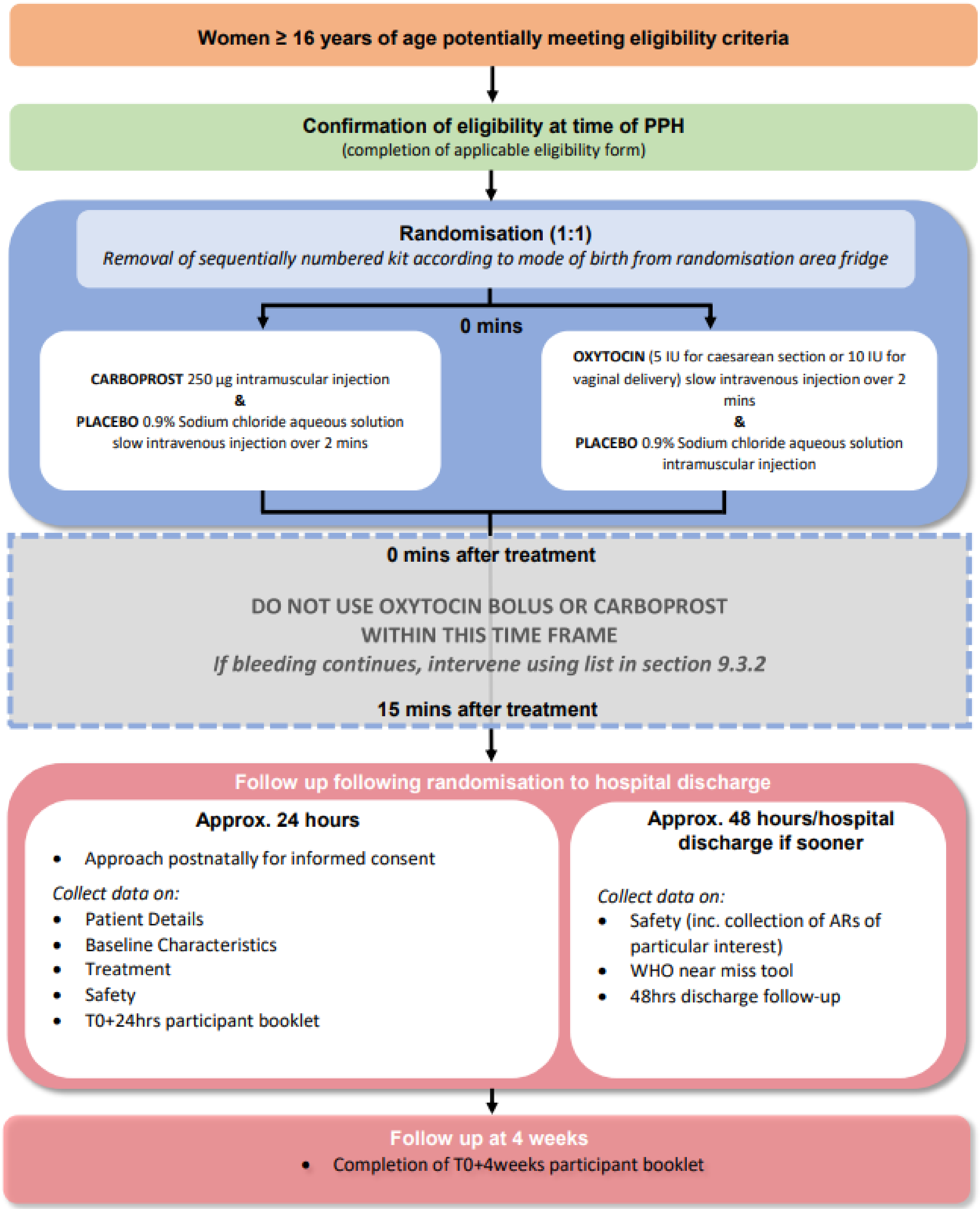

2 INTRODUCTION

2.1 Background

Bleeding after childbirth (postpartum haemorrhage, PPH) occurs after around 5% of births (depending on definition) and causes the death of 75,000 women worldwide each year (3). It is the second leading cause of direct maternal deaths in the UK (19 deaths between 2013-2015) (4). Significant maternal morbidity may also result from weakness secondary to anaemia, delayed recovery, psychological trauma, difficulties with breastfeeding and poor bonding with the newborn. Available treatments, including drugs with known side effect profiles, blood transfusion and invasive or surgical procedures such as hysterectomy can have substantial negative impact on the woman's recovery, long-term health and sense of wellbeing.

PPH is a clinical emergency. The bleeding in PPH is unpredictable and difficult to quantify, and so most clinicians treat early, as soon as they are unhappy with the bleeding. As a result of the unpredictability of PPH and difficulties in gaining emergency consent, there are few randomised trials of PPH treatments. The evidence used in guidelines is therefore based largely on studies of prophylaxis and small observational studies (5). The National Institute for Health Research (NIHR) Health Technology Assessment (HTA) programme has therefore called for research into PPH treatments as one of its research priorities. Recent advances in emergency intrapartum consent pathways, developed in part by applicants in partnership with consumer groups, have facilitated recruitment (6-9).

2.2 Rationale

As uterine atony is the most common cause, intravenous (i.v.) oxytocin is universally recommended as first line therapy. However, the recommended dosage varies. Whilst the National Institute for Health and Care Excellence (NICE) recommends 10 international units i.v.; the Royal College of Obstetricians and Gynaecologists (RCOG) guidelines and *Mothers and Babies: Reducing Risk through Audits and Confidential Enquiries across the UK* (MBRRACE-UK) suggest 5 international units (5, 10, 11). There are no direct comparisons of the two doses. Although studies suggest that 3 international units is adequate (12, 13), we will use a higher dose in this study (5-10IU) to prevent criticism of inadequate dosing. The only risk of this is hypotension that occurs with rapid injection, however this was not seen in a recent randomised controlled trial in which 517 women were given 10 international units of oxytocin as an i.v. bolus over 1 minute (14). The need for slow intravenous injection will be emphasised in training (see section 2.3 below). In women who have a caesarean section (who usually have intravenous oxytocin prophylaxis, have usually just had a spinal anaesthetic inserted and who have greater blood loss) we will give only 5IU IV in line with anaesthetic recommendations.

The PPH rate is increasing, and this may be partially related to the overuse of oxytocics in labour (11). Laboratory studies on myometrium suggests that repeated use of oxytocics leads to the saturation of oxytocin receptors and reduced efficacy of oxytocin as a therapy (15). Attention has therefore turned to the use of prostaglandins as an alternative mechanism for improving the strength of uterine contractions in the event of uterine atony. Misoprostol is a prostaglandin E1 analogue, which is convenient for both storage and administration. It has been studied extensively for both prophylaxis and treatment of PPH, and found to be less effective than oxytocin (3) and of no additional benefit when given in addition to standard drugs for PPH treatment (16). Carboprost is a prostaglandin F2a analogue that is given intramuscularly (i.m.). There are 13 small studies of carboprost for PPH prophylaxis. They show a reduction in blood loss compared with conventional uterotonics, but diarrhoea,

For peer review only - http://bmjopen.bmj.com/site/about/guidelines.xhtml

vomiting, fever or hypertension affects about 15% of those treated (17). Carboprost is also more expensive than oxytocin. There are no studies of carboprost for PPH treatment (18), but NICE recommends carboprost as a second line treatment. NICE, and others, have suggested the need for a major randomised trial to ascertain both the effectiveness of carboprost and its optimal position in a PPH treatment pathway (3, 5).

2.3 Risk and Benefits

Oxytocin is the standard first line treatment for atonic postpartum haemorrhage. It has been shown to cause effective uterine contraction, is low cost and relatively free from side-effects. Oxytocin can commonly cause headache, nausea and vomiting. The benefit of repeated doses (for example giving 10IU prophylaxis and then 10IU treatment shortly after) has however been questioned (3, 15), especially given that pharmacokinetic studies suggest that the optimal dosage is just 3IU (12). Furthermore, a bolus dose of intravenous oxytocin causes a rapid but transient fall in blood pressure by around 20mmHg (19) and has been implicated in maternal death during PPH (20). Repeated doses of oxytocin also cause water retention. It is therefore of uncertain benefit, and not without risks.

Carboprost, a prostaglandin F2α analogue, is usually reserved for second line management of atonic PPH. A systematic review of 13 small randomised trials comparing the efficacy of carboprost and conventional uterotonics for PPH prophylaxis found that it was associated with less blood loss, but around 15% of women suffered side effects including diarrhoea, vomiting, fever or hypertension (17).

2.4 Objectives

2.4.1 Primary Objective

The aim of the research is to compare intramuscular carboprost 250 micrograms with intravenous oxytocin (5 international units (IU) for caesarean section or 10 IU for vaginal birth) by slow injection over 2 minutes for the initial treatment for women with clinically diagnosed postpartum haemorrhage after giving birth in UK hospitals.

2.4.2 Secondary Objective(s)

1. To assess the relative cost-effectiveness of the use of carboprost and oxytocin as initial treatments for women with clinically diagnosed PPH.
2. To explore the views of participants and their carers about their experiences of the two treatments and the consent process.

2.5 Outcome measures

The outcomes selected for this study include the recently developed Core Outcome Set (COS) for PPH treatment trials (Appendix 1).

2.5.1 Primary Outcome

Blood transfusion defined as ‘any red blood cell transfusion or cell salvage of ≥ 300 mls commenced any time between randomisation and 48 hours after randomisation (or hospital discharge if earlier than 48 hours’.

For peer review only - http://bmjopen.bmj.com/site/about/guidelines.xhtml

2.5.2 Secondary Outcome

All secondary outcomes are collected prior to discharge or 4 weeks whichever is earliest unless otherwise specified.

1. Volume of blood transfusion
2. Use of a further uterotonic drug
3. Composite outcome of any organ dysfunction[1]
4. Hysterectomy
5. Blood loss
6. Blood loss ≥ 1000mls
7. Haemoglobin
8. Shock
9. Maternal death
10. Non-pharmacological approach to treat or investigate bleeding[2]
11. Manual removal of placenta
12. Adverse reactions of particular interest (as listed in section 10.1)
13. Skin to skin care with baby within the first hour after birth
14. Separation from new-born in first hour after birth
15. Breastfeeding
16. Childbirth Experience Questionnaire (CEQ)
17. Resource use[3]

For at least the first 9 months of recruitment, we will conduct qualitative research to explore the views and experiences of women recruited to the trial, their partners, and practitioners involved in recruitment and consent in COPE.

[1] Based on definitions within the WHO Maternal 'near-miss' Approach for maternal health (2).

[2] Interventions include: laparotomy, internal uterine tamponade with balloon or packing with gauze, arterial embolisation, removal of retained placenta or placental tissue, examination in theatre under anaesthetic and external uterine compression (bimanual or other).

[3] Number of units of blood transfused will be collected for resource use; transfers to higher care units will be collected within resource use.

For peer review only

3 TRIAL DESIGN

This is a double-blind, double-dummy, randomised controlled trial.

To determine the feasibility of answering the clinical question, an internal pilot examining rates of recruitment and consent, data completeness and sample size assumptions will be carried out. The timing of the internal pilot will be informed by the decision of the IDSMC and correspond to ability to reasonably estimate the primary outcome event rate with reasonable precision.

Criteria for progression from internal pilot to full study are:

Recruitment
Taking into account the pilot study and the ratio of vaginal to caesarean births, we will estimate the total recruitment period required to achieve the target sample size. If this is achievable within the recruitment period agreed with the funders then we will proceed to the main trial. If it exceeds this duration we will introduce ways to reduce this, and seek approval from the funder. If no obvious solutions exist and the funder is unable to support the time extension required then we will abandon the main trial.

Consent
If the rate of informed consent for follow-up and data use is 80% or more, proceed to main trial. If 60% to 80%, then review qualitative data to identify any aspects amenable to change, and proceed. If less than 60%, and no obvious solutions exist, then abandon the main trial.

Completeness of primary outcome data
If primary outcome data are available for over 95% of participants, then proceed. If 75-95%, identify aspects amenable to change and proceed to main trial as amended. If available for less than 75% of participants randomised, and no obvious solutions exist, abandon the main trial.

Sample Size estimation
The Independent Data Safety and Monitoring Committee (IDSMC) will be asked to review the assumptions made in the sample size calculation. As a result the numbers required may be increased but not decreased. If an increase in sample size is recommended, then this will be discussed with funders and informed by ability to deliver within time and budget.

For peer review only

For peer review only - http://bmjopen.bmj.com/site/about/guidelines.xhtml

4 STUDY SETTING AND SELECTION OF CENTRES / CLINICIANS

The study will take place in approximately 40 UK NHS hospital maternity units.

Criteria for the selection of centres will be determined by the Trial Management Group (TMG) and will be described in the supplementary document 'COPE Site Suitability Assessment'.

Initiation of centres will be undertaken in compliance with LCTC Standard Operating Procedures (SOPs). Centres fulfilling the criteria will be selected to be recruitment centres for the COPE trial and will be opened to recruitment upon successful completion of all global (e.g. Research Ethics Committee (REC) and Medicines and Healthcare Products Regulatory Agency (MHRA)) and study-specific conditions (e.g. site personnel training requirements) and once all necessary documents have been returned to LCTC as detailed in the trial 'greenlight' checklist.

Participating centres will be listed in a log, maintained separately to the protocol.

For peer review only

5 STUDY POPULATION

The requirements of Good Clinical Practice (GCP) are that a doctor should either recruit or supervise the recruitment of individuals to a Clinical Trial of an Investigational Medicinal Product (CTIMP). Thus women giving birth at home or in stand-alone midwifery units will not be eligible to participate.

5.1 Inclusion Criteria

- ≥ 16 years of age
- Requirement for medical treatment for primary PPH

5.2 Exclusion Criteria

- Known to have opted out of participation antenatally
- Known oxytocin or carboprost hypersensitivity
- Known active cardiac or pulmonary disease
- Known to have previously been treated as part of COPE
- Has already received carboprost prophylactically for postpartum haemorrhage
- Has already received uterotonic drug treatment for postpartum haemorrhage (this does not include PPH prophylaxis)
- Stillbirth

5.3 Co-enrolment Guidelines

Individuals who are currently recruited into other intrapartum interventional studies may be approached for the COPE trial upon agreement with the study Chief Investigator. Where recruitment into another trial is considered to be appropriate and without having any detrimental effect on the COPE trial this must first be discussed with the LCTC who will contact the Chief Investigator, Prof Andrew Weeks.

For peer review only

For peer review only - http://bmjopen.bmj.com/site/about/guidelines.xhtml

6 SCREENING, RECRUITMENT AND RANDOMISATION

6.1 Screening

Screening and eligibility information will be collected by completion of participant screening and eligibility forms. These forms will provide important information for monitoring purposes.

6.2 Recruitment

PPH is an unpredictable clinical emergency occurring after around 5% of births. COPE participants will be recruited via the following emergency pathway:

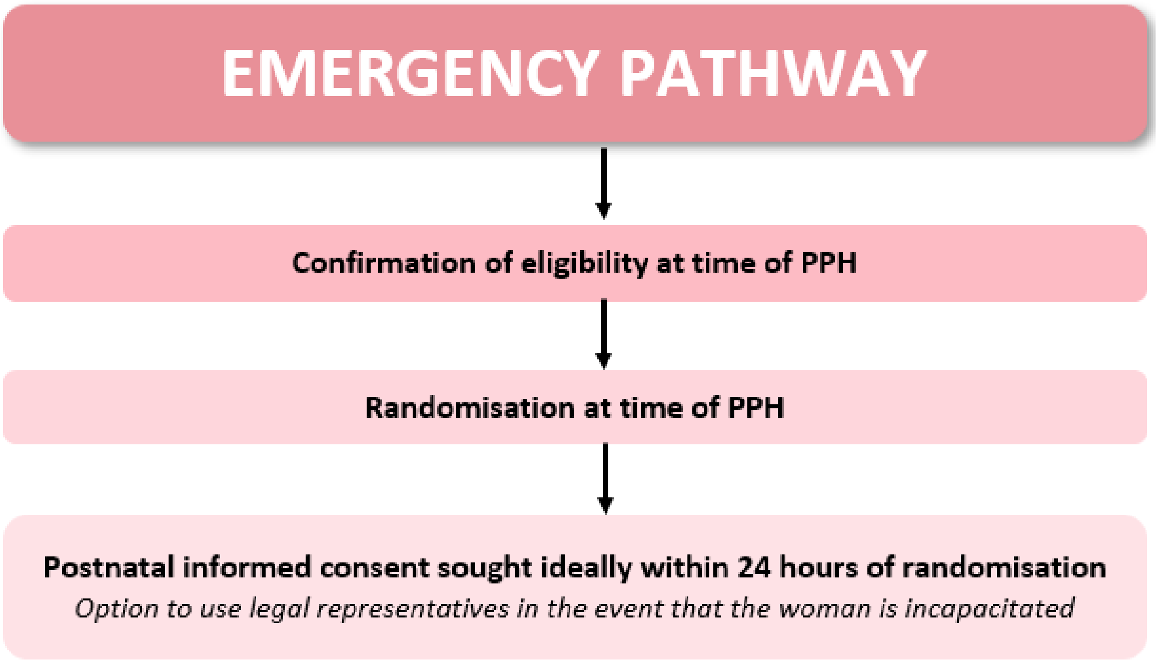

6.3 Consent Procedures

Randomisation and treatment will occur if eligibility criteria are met after child birth. Anonymous data, for the purposes of the recipients, will be sent to the LCTC for processing under public interest to allow monitoring of safety. Personal identifiable information will be subsequently sent to LCTC following written informed consent.

6.3.1 Information provision and postnatal consent

A variety of strategies will be used to advertise the study to raise awareness amongst women antenatally, including: patient posters, antenatal clinical discussions, and social media campaigns. Study advertising will include details of how women can contact the local study team to discuss the study.

Once a woman has been diagnosed with PPH, a critical clinical emergency situation exists. Eligible women have a life threatening condition and risk of death is highest early after delivery.

For peer review only - http://bmjopen.bmj.com/site/about/guidelines.xhtml

Their mental capacity, physical and emotional state may be diminished as a result of blood loss, pain, or by drugs administered during the labour.

Eligible women will be randomised into the trial in the emergency situation where treatment needs to be given urgently and there is no time for prior consent from a Legal Representative. An overview of the emergency pathway and postnatal consent procedure is provided in section 6.2. The treating clinician may briefly explain the study to the woman if it is deemed appropriate. Note that it is not the intention to obtain verbal consent in this emergency situation. For women who are randomised, but they or their legal representatives do not provide consent postnatally, anonymous data, for the purposes of the recipients (LCTC) will be sent for processing under public interest to allow monitoring of safety. The provision of anonymous data from sites to the LCTC is in the line with the General Data Protection Regulations (2018) and Common Law. Where consent is not provided no contact will be made with the woman's GP.

6.3.1.1 Consent provision where capacity is regained promptly

Postnatally, once the woman is stable and ideally within 24 hours or prior to discharge/transfer to another hospital, full study information will be provided to the woman. Before approaching women, the trial recruiter will firstly check whether the timing is appropriate with the clinical team. Permission will be sought for disclosure of identifiable data and continued follow-up. The woman will be asked to sign the "Postnatal (emergency pathway) Patient Information Sheet and Consent Form".

6.3.1.2 Consent provision where capacity is not regained or is delayed

Before approaching women, the trial recruiter will firstly check whether the timing is appropriate with the clinical team. Should it be apparent that incapacity is likely to continue, full study information will be provided as soon as practicable to her Personal Legal Representative (PeR) or Professional Legal Representative (PrR), if PeR is not available. Permission will be sought for disclosure of identifiable data and continued follow-up. The PeR or PrR will be asked to sign the "Postnatal (emergency pathway) Personal Legal Representative Information Sheet and Consent Form" or "Postnatal (emergency pathway) Professional Legal Representative Information Sheet and Consent Form", respectively.

The consent given by an appropriate legal representative on behalf of a woman lacking capacity to do so for themselves shall represent the presumed will of the incapacitated woman and as such it is not mandatory to re-approach women recruited in such a manner should they regain their capacity to give consent during the course of their participation in COPE.

Legal representatives will be made aware that, should this circumstance arise during the trial participation period (4 weeks), the woman will be provided with information about COPE but will not be approached to provide their own written consent. The woman will be made aware that they can withdraw from the trial at any time by revoking the informed consent provided by their legal representative as stated in section 7.5.2.

6.3.2 Principles of informed consent

In obtaining and documenting informed consent, the investigator/delegate should comply with applicable regulatory requirements and should adhere to the principles of GCP and to the ethical principles that have their origin in the Declaration of Helsinki.

At the appropriate time, the objectives, risks and inconveniences of the trial and the conditions under which it is to be conducted are to be provided to the woman or their legal representative by staff with experience in obtaining informed consent. A Patient Information Sheet and

For peer review only

Consent form, describing in detail the trial interventions, trial procedures and risks will be approved by a REC, and the woman (or their Legal Representative) will be asked to read and review the document. Upon reviewing the document, the investigator will explain the research study to the woman (or their Legal Representative). This information will emphasise that participation in the trial is voluntary and that the woman may withdraw from the trial at any time and for any reason. All women (or their Legal Representative) will be given opportunity to ask any questions that may arise, and should have the opportunity to discuss the study with their friends and family, and time to consider the information prior to agreeing to consent. A contact point where further information about the trial may be obtained will be provided.

Women will have as long as possible to consider participation in COPE. Research staff should aim to meet the woman to seek consent within 24 hours, or prior to discharge or transfer to another hospital. The woman or their Legal Representative will be asked to sign and date the informed consent document. Both the person obtaining consent and the woman (or their Legal Representative) must personally sign and date the form. A copy of the consent form will be given to the woman (or their Legal Representative) for their records. The original copy will be filed in the Investigator Site File, a copy filed in the woman's notes and a further copy should be sent to the LCTC. Following provision of consent, a COPE GP letter will be sent to the woman's GP by the recruiting site.

The woman (or their Legal Representative) may, without being subject to any resulting detriment, withdraw consent for trial participation any time by revoking their informed consent as stated in section 7.5.2. In this circumstance or where the woman (or their Legal Representative) declines to provide consent, the rights and welfare of the woman will be protected and the quality of medical care will not be adversely affected.

6.3.2.1 Definitions of legal representatives

England and Wales:

- Personal Legal Representative i.e. a person not connected with the conduct of the trial who is suitable to act as the legal representative by virtue of their relationship with the adult, and is available and willing to do so. If one is not available:
- Professional Legal Representative i.e. a person not connected with the conduct of the trial who is the doctor primarily responsible for the medical treatment of the adult, or a person nominated by the healthcare provider.

Scotland:

- Personal Legal Representative i.e.
  - Adult's Welfare Guardian or Welfare Attorney, or if not appointed:
  - The adult's nearest relative, if neither are reasonably contactable:
- Professional Legal Representative i.e. a person not connected with the conduct of the trial who is the doctor primarily responsible for the medical treatment of the adult, or a person nominated by the healthcare provider.

6.3.3 Discharge/transfer to another hospital prior to postnatal consent

It is expected that consent will be sought for all women prior to discharge/transfer to another hospital (in the event that the woman dies prior to consent being sought refer to section 6.3.4).

Where consent is not sought prior to discharge/transfer the following should occur:

For peer review only - http://bmjopen.bmj.com/site/about/guidelines.xhtml

The Research Midwife (RM), or other designated member of the research team at site, will call the woman within 5 working days of randomisation to inform them of their involvement in the trial and provide details. If the woman cannot be contacted, then the process for postal consent should be followed.

If the woman is willing to provide consent, the RM will outline the two different ways in which consent could be provided, as per the below, and ask the woman which way they would prefer. If during the conversation the woman is clear that they do not wish to participate then no trial documentation/correspondence or follow up documentation will be sent.

Two methods are provided for confirming consent:

1. **Electronic-consent:** If the woman would prefer to use Electronic-consent then their email address must be provided to the site. The person speaking with the woman must ensure that the woman is fully informed that their email address will be used for this purpose. Full guidance on obtaining consent electronically can be found in COPE Electronic-consent guidance document.

   LCTC will facilitate the collection of validated E-consent. The E-consent request will expire after 4 weeks from the date sent. Once complete, all signatories and LCTC will receive a copy of the final signed consent form.

2. **Postal consent:** the RM, or other designated member of the research team at site, will post the below items to the woman's address within 5 working days:
   - Participant covering letter
   - Participant Information Sheet and Consent form (Home version)

   The covering letter will confirm that the woman has 4 weeks from the date of the letter to return the consent form confirming whether they would like to continue participation in the trial.

   When the consent form is received by the RM, they will sign or organise for an authorised individual to sign the consent form. When signed, the RM will send a copy to the LCTC.

Note: Audio-recordings of telephone discussions are not required.

If no response is received within 2 weeks (14 days), the RM, or other designated member of the research team at site will make a follow up call to the woman to check that they have received the information. Postal consent documents/e-consent email will be re-sent/forwarded, respectively, if the site team member is unable to contact the woman or information has not been received.

If no response is received via E-consent or postal consent within 4 weeks (28 days), the woman's identifiable data will not be included within the trial as confirmed in the telephone conversation/correspondence. Consent is being sought in this scenario for the disclosure of confidential information in order to avoid a breach of the common law duty of confidentiality. The four-week questionnaire will be sent by post with another consent form to give the woman another opportunity to provide consent. If we find that consent in this scenario is not practicable and results in informative missing data (e.g. linked to severity of condition or duration of hospital stay) a future Section 251 application may be made.

For peer review only - http://bmjopen.bmj.com/site/about/guidelines.xhtml

Collection of personal identifiable information is restricted to the data requested on the consent form. All other data are anonymous to the purposes of the recipients (LCTC) and processed under public interest and are necessary for monitoring of safety.

6.3.4 Maternal death prior to postnatal consent

This is likely to be a very rare occurrence. However, in the event that a woman dies before consent has been sought, the attending RM will obtain information from colleagues and bereavement counsellors to establish the most appropriate practitioner to notify the Personal Legal Representative of the research involvement.

Consent can be sought from Personal legal representatives following the death of their relative and prior to the Personal Legal Representative's departure from the hospital. However, it is at the discretion of the site staff to determine if this is appropriate for each individual family. In this situation, the "Bereaved Postnatal (emergency pathway) Personal Legal Representative Information Sheet and Consent Form" would be used.

If consent is not sought prior to the Personal Legal Representative's departure from hospital then the Personal Legal Representative will be notified and consent can be sought by an appropriate practitioner at a bereavement follow-up visit. If it is not possible to obtain Personal Legal Representative consent then Professional Legal Representative consent form can be completed.

In addition, all deaths, irrespective of the consent status, should be reported on a Serious Adverse Event Case Report Form (CRF) and securely emailed to the LCTC within 24 hours of the research team becoming aware (see section 10.5).

6.3.5 Neonatal death prior to postnatal consent

This is likely to be a rare occurrence. However, if a participant experiences the death of their baby before consent has been sought, the attending RM will obtain information from colleagues and bereavement counsellors to establish the most appropriate practitioner to notify the participant of the research involvement.

Consent will be sought if considered appropriate; however, it is at the discretion of the site staff to determine if this is appropriate for each individual participant. In this situation, the usual information sheet and consent form would be used.

6.4 Randomisation Procedures

Participants will be randomised in a 1:1 ratio using random variable block size, stratified by mode of birth (caesarean section or vaginal birth).

Centres will be provided with a series of sequentially numbered, sealed treatment kits to be received by the pharmacy department and distributed to an appropriate secure location within randomisation areas i.e. delivery suite for ready access upon presentation of eligible patients.

Each kit will contain either:

a) An ampoule of carboprost and an ampoule of placebo or
b) An ampoule of oxytocin and an ampoule of placebo.

Departments will be provided with case report forms (CRFs) to be completed promptly at the time of recruitment (to document key screening/eligibility and baseline data).

For peer review only - http://bmjopen.bmj.com/site/about/guidelines.xhtml

After eligibility is confirmed by an authorised medical doctor, the next sequentially numbered treatment kit for the particular mode of birth (vaginal or caesarean section) should be selected and administered. The treatment kit's identification number is the randomisation number for the woman and should be recorded in their medical notes and on the trial CRFs.

All women randomised in to COPE (treatment kit removed from fridge following confirmation of eligibility) who go on to receive the COPE treatment or an alternative medicinal treatment for PPH should be approached for their consent and followed up as a COPE participant as per COPE protocol. If a treatment kit was removed from the fridge but a medicinal treatment for PPH was not administered (COPE treatment kit or other) then consent does not need to be sought and they should not be followed up as a COPE participant.

A member of the trial site team as delegated on the delegation log will periodically check to ensure that there are adequate stocks of trial treatment kits, that the correct number of treatment kits are present and intact and that the sequential numbering system is maintained. Any discrepancies will be immediately reported to the LCTC.

6.5 Who is Blinded to Allocations

This is a double-blind study and all individuals involved in the conduct and delivery of the trial, except for the randomising statistician, or those unblinded to individual cases as a requirement (e.g. for safety reporting), will be blinded to treatment allocations. Statisticians involved in monitoring will be unblinded following determination of participant inclusion within each analysis population.

In case of the need of emergency unblinding, unblinding envelopes will be provided and stored at an agreed location within the site that is readily accessible at time of need. The construction of these envelopes is resistant to accidental damage or tampering and contents cannot be viewed without fully opening.

The site team will periodically check to ensure that the appropriate number of unblinding envelopes are present and intact. Any discrepancies will be immediately reported to the LCTC.

For unblinding procedures see section 9.7.

For peer review only - http://bmjopen.bmj.com/site/about/guidelines.xhtml

7 PARTICIPANT TIMELINE, ASSESSMENTS AND PROCEDURES

7.1 Schedule for Follow-up

Participants will be recruited and followed up to a maximum of 4 weeks postnatally.

Procedures		Follow-Up Schedule: Screening	Follow-Up Schedule: Baseline (T0)[1]	Follow-Up Schedule: T0 - 48 hrs or hospital discharge if sooner[3]	Follow-Up Schedule: T0 + 4 wks[4]
Assessment of Eligibility Criteria		X	X		
Confirmation of Full Eligibility by an authorised medical doctor			X		
Randomisation			X		
Administration of Study Intervention			X		
Signed Postnatal Consent Form[2]				X*	
Baseline Characteristics			X		
Clinical Outcomes	The use and timing of additional uterotonics			X	
	Manual removal of placenta			X	
	Hysterectomy			X	
	Blood loss at birth			X	
	Non-pharmacological approach			X	
	Skin to skin care with baby			X*	
	Separation from new-born in first hour after birth			X*	
	Breastfeeding initiation (first 24hrs)			X*	
	Blood transfusion or cell salvage			X	
	Volume of blood transfusion			X	
	Haemoglobin[3]			(X)*	
	Shock			X	
	Exclusive breastfeeding (at 48hrs/ discharge if sooner)			X	
	Adverse Reactions of particular interest		X	X	
	Outcome of any organ dysfunction			X	(X)
	Maternal death[5]		(X)	(X)	(X)
	Exclusive breastfeeding (at 4 weeks)				X
Assessment of Serious Adverse Events[6]			X	X	(X)
Childbirth Experience Questionnaire					X
EQ-5D-5L questionnaire and EQ-VAS				X*	X
Healthcare Resource Utilisation Questionnaire					X

For peer review only

For peer review only - http://bmjopen.bmj.com/site/about/guidelines.xhtml

(X) – As indicated/appropriate.
[1] At baseline, all procedures should be done before study intervention.
[2] Postnatal (emergency pathway) consent is obtained after childbirth ideally within 24 hrs or prior to discharge / transfer to another hospital. If consent is not obtained prior to discharge, the appropriate consent procedures should be followed as per section 6.3.
[3] Haemoglobin (in non-transfused women only) will be ideally obtained postnatally on the day following birth (12-36 hours post birth) or at discharge, whichever is soonest. For more details, see Appendix 1.
[4] Participant to be contacted initially using her favoured mode of communication (letter or telephone), with a letter or telephone reminder if no response within 2 weeks
[5] All deaths collected from the time of randomisation until hospital discharge or 4 weeks, whichever is earlier.

[6]Serious Adverse Events and Serious Adverse Reactions will be actively monitored and reported from the time of randomisation until hospital discharge or 4 weeks, whichever is sooner. ARs of particular interest collected on a separate single timepoint CRF, as per section 10.
* To be completed at 24 hours after randomisation.

7.2 Procedures for Assessing Efficacy

Clinical efficacy outcomes will be collected at 24 hours and at 48 hours or at hospital discharge, if sooner. Details of the outcomes (with the timing of assessment and definitions) are included in Appendix 1. COPE OUTCOMES.

7.3 Procedures for Assessing Safety

The Principal Investigator (PI) or delegated research staff will be monitoring and reporting all relevant adverse events (see section 10) from randomisation until hospital discharge or 4 weeks, whichever is sooner.

7.4 Other Assessments

7.4.1 Childbirth Experience Questionnaire (CEQ)

The CEQ was developed in 2010 to measure the impact of an intervention on a woman's childbirth experience. It includes four main aspects of the childbirth experience: Own capacity, Professional support, Perceived safety and Participation.

In COPE, the UK-validated CEQ will be completed by the participants as a paper or telephone questionnaire at 4 weeks.

7.4.2 Health Economics assessments

7.4.2.1 Resource use and costs

Resource use will be based on:

(1) Entries made in designated sections of participants' CRFs. The CRF will be used to record data on procedures (surgical) and interventions (including units of blood products transfused) as well as dates of patient admission and discharge. This will be completed prior to discharge.

(2) A resource use questionnaire, administered at the 4-week follow-up, using the participant's favoured mode of communication (letter, telephone) with a letter or telephone reminder if no response provided within 2 weeks.

(3) Hospital Episode Statistics (HES) and Scottish Morbidity Records (SMR) data sourced from i) NHS Digital for participants recruited in England; ii) NHS Wales Informatics Service for

For peer review only - http://bmjopen.bmj.com/site/about/guidelines.xhtml

participants recruited in Wales; and iii) the electronic Data Research and Innovation Service for participants recruited in Scotland.

- Data will be requested from the above listed NHS organisations pertaining to outpatient, inpatient, critical care and A&E attendances from 3 months prior to randomisation, to 4 weeks post randomisation (following completion of follow-up). Participant information (postcode, date of birth, NHS (or CHI) number and screening/randomisation number) will be securely transferred to authorised personnel at the NHS organisations and the HES and SMR data with the screening/randomisation number will be securely transferred to health economists at Centre for Health Economics and Medicines Evaluation (CHEME), Bangor University, who will be conducting the economic analysis.
- The only identifier present in the HES / SMR dataset will be the trial randomisation identifier, and health economists at CHEME will not have any access to keys linking this to participant personal data. Electronic HES and SMR records will be stored on secure Bangor University computer servers which meet NHS data security standards. Access will be restricted only to health economists working on the trial.

Unit costs will be obtained from NHS reference costs and other standard NHS sources.

7.4.2.2 Health outcomes

Quality Adjusted Life Years (QALYs) will be estimated from utilities derived from trial participant responses to EQ-5D-5L questionnaires (and applying recommended methods for generating UK relevant utilities) administered for completion at 24 hours and at 4-weeks post randomisation.

7.5 Patient Transfer and Withdrawal

7.5.1 Discontinuation

Upon completion of trial intervention, subsequent treatments and actions will be recorded (see section 9.3.2). This would **not** necessitate the participant discontinuing the study.

7.5.2 Withdrawal of Consent

Generally, follow-up will continue unless the woman/Legal Representative explicitly withdraws consent for follow-up.

The woman/Legal Representative is free to withdraw consent at any time without providing a reason and without being subject to any resulting detriment. The rights and welfare of the woman will be protected by emphasising to them throughout the trial that the quality of medical care will not be adversely affected if they decline to participate in the trial, or withdraw consent.

Where the woman/Legal Representative wishes to withdraw consent, a withdrawal CRF should be completed documenting the reason for withdrawal. Centres should explain the importance of remaining in trial follow-up and of the importance of contributing data. Data collected prior to withdrawal will be analysed and data relevant to safety events will continue to be collected to meet regulatory requirements.

7.5.3 Participant Transfers

A minority of women may be randomised and treated at a COPE site and then transferred to another hospital for further treatment before the 48-hour follow-up period is completed (e.g. the participant needs to be transferred to a hospital with intensive care facilities).

For peer review only - http://bmjopen.bmj.com/site/about/guidelines.xhtml

In these cases, the treating hospital should contact the transfer hospital to collect the follow-up information and consent (where possible).

Note: Only transfer sites that are also a recruiting site for COPE can assist with the consent process, however, regardless of where consent was sought, the participant would be classified as a recruited participant for the treating site only.

7.6 Loss to Follow-up

Follow-up contact will be made via the PI or designated research staff at each centre. After discharge, participants will be contacted via their preferred means of communication for the purposes of the 4-week follow-up.

Non-responders will be contacted again after 2 weeks. Failure to respond to the second contact within another 2 weeks will deem the participant “loss to follow-up”.

Wherever possible, information on the reason for loss to follow-up will be recorded.

7.7 Trial Closure

The end of the trial is defined to be the date on which data for all participants is locked and data entry privileges are withdrawn from the trial database. However, the trial may be closed prematurely by the Trial Steering Committee (TSC), on the recommendation of the Independent Data and Safety Monitoring Committee (IDSMC).

For peer review only

8 EMBEDDED MIXED METHODS RESEARCH

8.1 Design

Recruitment to the embedded mixed methods research has now closed.

For at least the first 9 months of recruitment, we will use interviews, and questionnaires to explore the views and experiences of women recruited to the trial and their birth partners. In the five pilot sites (or alternative site if a pilot site is not open to recruitment within two months of recruitment of the first woman to the trial) we will also conduct focus groups with practitioners and audio-recorded recruitment discussions. This embedded research will explore:

- experience and acceptability of the trial and its interventions;
- antenatal and postnatal information provision;
- practitioner experience of recruitment;
- the interaction and exchange of information about the trial between staff and potential participants;
- emergency recruitment at time of PPH and postnatal consent;
- antenatal consent amongst both those recruited and not recruited;
- postnatal consent for the disclosure of identifiable data and follow-up.

Interim findings from research conducted in the internal pilot will be used to inform approaches to recruitment and consent procedures for the remainder of the COPE trial.

Methods will include:

A: Audio recording of COPE antenatal and postnatal consent discussions between women and their birth partner (if applicable) and trial recruiters (only at the internal pilot sites).

B. A questionnaire with women and their birth partners (if applicable) who consent or decline consent for the COPE trial (all sites).

C: Interviews with women and their birth partners (if applicable) who consent or decline consent for the COPE trial, including bereaved parents (all sites)

D: Focus groups and/or interviews with COPE trial recruiters at each pilot site (only at the internal pilot sites)

8.2 Selection of participants

<u>Women and birth partners</u>

Inclusion criteria

- Women and their birth partners who are recruited to COPE

Exclusion criteria

- Women and their birth partners who do not speak English or lack the capacity to consent

For peer review only - http://bmjopen.bmj.com/site/about/guidelines.xhtml

COPE practitioners

Inclusion criteria

- Practitioners who are involved in screening, recruiting, randomising and consenting participants/ legal representatives

Exclusion criteria

- None

Consent sought from	Location consent was sought	A: Audio-recording	B: Questionnaire	C: Interview
Woman and birth partner (if present) who do and do not give consent for COPE trial	On-site (antenatal and postnatal following birth)	✔ (at pilot sites only)	✔	✔
Bereaved woman and birth partner (if applicable)	On-site (postnatal)			✔
Woman and birth partner (if applicable)	Home (postnatal)		✔	✔
Bereaved women and birth partner (if applicable)	Home (postnatal)			✔

8.3 Enrolment and procedure

8.3.1 Part A: Audio-recordings

Delegated COPE recruiters at pilot sites will seek permission to audio-record recruitment consultations when they first approach potential women and their birth partners about COPE in postnatal settings. If permission for audio-recording is declined the recruitment consultation will not be recorded. If permission is given, the recruiter will record this on the relevant COPE information sheet and consent form and activate an audio-recorder. If there is more than one trial discussion (e.g. an initial discussion followed by a full trial discussion after woman has considered the trial information) then all discussions should be recorded.

Completed audio-recordings will be uploaded to a secure online file store for professional transcription purposes. The 'Guidance for the management of audio recordings' should be used to ensure that the recordings are correctly stored and uploaded in accordance with the Data Protection Act.

Audio-recording of trial consultations will take place at each of the five pilot sites (or alternative site if a pilot site is not open to recruitment within two months of recruitment of the first woman to the trial) until the sample target (data saturation point) is achieved. Sites will be informed when audio-recordings can stop.

Note: Please refer to section 8.2 for audio recordings applicability.

For peer review only - http://bmjopen.bmj.com/site/about/guidelines.xhtml

8.3.2 Part B: Questionnaires

At postnatal recruitment discussions COPE recruiters will ask all women and their birth partners (including those who decline consent for the COPE trial, but not including bereaved women and their birth partners) to complete the COPE recruitment questionnaire, place it in a sealed stamped addressed envelope and return it to the recruiter to post to qualitative researcher.

If consent has been given to complete a questionnaire within the COPE information sheet and consent form, but a questionnaire has not been received, the questionnaire will be sent via post. In the rare instance that COPE trial consent is not sought prior to discharge/transfer to another hospital, the site team should send the questionnaire by post to women and their birth partners along with the COPE information sheet and consent form to complete. If questionnaires are not returned the embedded study team will ask women and birth partners to complete the questionnaire online at the point of arranging interviews (see below).

Recruitment for questionnaires will be conducted for the internal pilot and for at least the first 9 months of recruitment. We anticipate to collect approximately 500 questionnaires.

Note: Refer to section 8.2 for questionnaire applicability.

8.3.3 Part C: Interviews with women and their birth partner

COPE recruiters will ask women and their birth partners if they wish to take part in a telephone interview or face-to-face interview (if they live in the Northwest of England). Their permission to be contacted to arrange an interview will be recorded on the relevant COPE information sheet and consent form. They will be contacted using their contact details provided. We aim to interview a selection of women and their birth partners, who decline consent and provide consent.

Following consent to do so, the University of Liverpool qualitative researchers will make contact with women and, their birth partner (if applicable) after the birth to arrange remote interviews (telephone or online) or face-to-face interviews (Northwest only) at their home or location of choice (e.g. hospital, University) approximately 2 to 4 weeks after they have given birth. Telephone interviews with women and their birth partners will be conducted separately. Those involved in face-to-face interviews can choose to be interviewed together. Bereaved women will be contacted no less than six weeks after birth. Such calls/interviews will be handled with sensitivity and will be arranged within a timeframe tailored to individual women.

All interviews will be conducted by qualitative researchers who have proven skills in the conduct of research in sensitive settings. Any distress during the interviews will be managed with care and compassion and participants will be free to decline to answer any questions that they do not wish to answer or to stop the interviews at any point. Such participants will be supported in obtaining appropriate help.

Interviews will be conducted until point of sample or data saturation (approximately 15-35), whereby no new themes are emerging in the analysis of data). It is expected that the interviews will take on average between 30 to 60 minutes.

After the interviews are complete, qualitative researchers will send participants a letter and a £30 high street shopping voucher to thank them for their time. All participants who express an

For peer review only - http://bmjopen.bmj.com/site/about/guidelines.xhtml

interest in taking part but are not selected for an interview will be contacted via email by a qualitative researcher (if address provided) to thank them for their interest in the study.

8.3.4 Part D: Focus groups and/or interviews with the COPE practitioners

COPE practitioners (e.g. recruiters, PI or lead RM) at the five pilot sites (or alternative site if a pilot site is not open to recruitment within two months of recruitment of the first woman to the trial) will be sent an email invitation to participate in an online focus group or remote interview (telephone or online via Zoom or Microsoft Teams) (whichever is possible to arrange around clinical practice). Interviews and focus groups will take place between 3 and five months after site opening (depending upon sufficient experience of recruitment and staff availability)

Those who register interest will be sent a practitioner information sheet including details of topics that will be discussed in the focus group or telephone interview including:

- acceptability of the trial;
- experience of recruitment and the approach to consent;
- potential barriers to recruitment and any potential solutions;
- how information about the trial is communicated to women;
- how women respond to trial information and any suggested improvements to trial information and consent processes.

All interviews will be conducted by the qualitative researcher:

Consent will be sought from participants before the focus group or telephone interview begins. This will involve the qualitative researcher explaining the aims of the study to the practitioner and providing an opportunity for questions. For focus groups, PIS and consent forms will be sent to practitioners via email. Practitioners will be asked to complete and return the consent form to the embedded study researcher before the scheduled focus group. For telephone or online interviews, the qualitative researcher will read each aspect of the consent form, including consent for audio recording of the interview and ask whether the participant would like to receive a copy of the findings when the study is complete. The qualitative researcher will tick each box on the consent form when the participant provides verbal consent. Informed consent discussions will be audio recorded for analysis.

Participation will be entirely voluntary and practitioners will be able to withdraw at any time without giving a reason.

After the interviews are complete, site research staff will be sent a thank you letter.

For peer review only - http://bmjopen.bmj.com/site/about/guidelines.xhtml

9 TRIAL TREATMENTS

9.1 Introduction

This is a randomised, double-blind, double-dummy trial. The investigational medicinal products (IMPs) in this trial are carboprost and oxytocin. Participants will be randomised to one of the following treatment arms:

Intervention: Carboprost 250 micrograms by deep intramuscular injection and placebo by slow intravenous injection over 2 minutes.

Control: Oxytocin (5 international units (IU) for caesarean section or 10 IU for vaginal delivery) by slow intravenous injection over 2 minutes and placebo by deep intramuscular injection.

9.2 Formulation, Packaging, Labelling, Storage and Stability

9.2.1 Formulation

COPE trial active treatments (carboprost and oxytocin) are market-authorised drugs. The formulations will be supplied in their marketed form and will not be manipulated other than labelling and packaging to maintain double-blind, carried out by clinical supply company.

9.2.1.1 Carboprost

Active carboprost 250 micrograms (Pfizer Limited) is presented as 1 ml of a colourless, sterile, aqueous solution for intramuscular injection.

9.2.1.2 Oxytocin

Active oxytocin 10 international units (Hameln Pharmaceuticals Ltd) is presented as 1 ml of a colourless, sterile, aqueous solution for intravenous injection.

9.2.1.3 Placebo

The matching placebo is presented as 0.9% Sodium Chloride in 1 ml colourless, sterile, aqueous solution for intramuscular injection or intravenous injection.

9.2.2 Packaging and Labelling

IMP supplies will be secured via a clinical services company, who will procure, over-label and package into blinded treatment kits in accordance with regulation 46 SI 2004/1031 and the detailed guidance provided in annex 13 of the EU Good Manufacturing Practice (GMP) guide. Kits will be sequentially numbered and their number will become the participant's randomisation number.

Each kit will contain two ampoules in an outer carton. Both ampoules and the outer carton will be labelled. Each ampoule is intended for a single dose for a single participant.

For peer review only

9.2.3 Storage and Stability

Treatment kits will be temperature monitored whilst stored at the central manufacturing facility and during shipment to participating centres. Treatment kits will be received and stored by the site pharmacy prior to being distributed in batches to the participating department where they will be stored until randomisation in an appropriate, secure refrigerator (randomisation area). Randomisation represents the point at which a kit is removed from the fridge, following confirmation of woman's eligibility. The fridge temperature should be maintained at 2-8°C. Kits should not be frozen and ampoules should be kept refrigerated and in their outer carton until required.

The temperature of storage locations will be monitored and documented daily in the temperature log. In the event of storage temperatures falling outside the 2-8°C range permitted, stock should be immediately quarantined in a monitored refrigerator and the trial coordinator at the LCTC contacted for further actions. Stock should not be removed from quarantine until instructed to do so by the trial co-ordinator. Stock that falls below 2°C should be quarantined and should not be used in the trial; the trial co-ordinator will confirm when this stock can be destroyed. Some stock that goes above 8°C may be used again with a shortened expiry date (12 weeks from excursion). This stock must remain in quarantine until the trial co-ordinator has confirmed that it can be used again. If the trial co-ordinator confirms that this can be used again then the site must apply a new label to the stock with the new expiry date and batch number (labels will be provided by the LCTC). Full guidance on re-labelling activities can be found in COPE guidance for re-labelling. Re-labelling is to be performed under GMP conditions with exemption 37 for labelling. Two people at site (one of whom should be a pharmacist) will complete re-labelling following the guidance document and record on the COPE re-labelling log when completed. Once re-labelling has been completed the trial co-ordinator will confirm when the stock can be removed from quarantine.

9.3 Distribution, Dispensing, Dosage and Administration of Study Treatment/s

9.3.1 Distribution and Dispensing

IMPs will be supplied as bulk stock from the distributor to pharmacy in kits containing two ampoules. Authorised, trial-trained pharmacy staff should distribute sufficient kits to the designated randomisation area(s) where they should be stored in accordance with section 9.2. Pharmacy staff at site will sign and date an accountability log documenting receipt of IMP and recording the distributed location.

Kits should be dispensed at time of randomisation following confirmation of eligibility. Dispense represents the point at which kits are removed from the randomisation area fridge. Staff at site will sign and date accountability logs documenting receipt in randomisation area and dispense for randomisation.

9.3.2 Dosage and Administration

Study interventions are supplied as ready-for-use, 1 millilitre formulations as single doses for a single participant. Both ampoules in each kit should be administered following randomisation in accordance with COPE protocol and local practice (this includes option to dilute intravenous ampoule contents in 10 millilitres saline to facilitate slow injection):

- Carboprost 250 micrograms by deep intramuscular injection (Pfizer Limited; Hemabate Sterile Solution) and placebo by slow intravenous injection over 2 minutes

For peer review only - http://bmjopen.bmj.com/site/about/guidelines.xhtml

OR

- Oxytocin (5 IU for caesarean section or 10 IU for vaginal delivery) by slow intravenous injection over 2 minutes (Hameln Pharmaceuticals Ltd; Oxytocin 10 IU/ml Concentrate for Solution for Infusion) and placebo by deep intramuscular injection.

Note: To achieve 5 IU oxytocin (caesarean section) draw up 0.5 ml of intravenous ampoule contents and discard the remaining 0.5 ml.

As far as packaging allows, **both** ampoules should be visually inspected for particulate matter and discoloration prior to administration. Should this be observed, the whole kit (two ampoules) should be quarantined, segregated from the other IMP and pharmacy notified. If neither ampoule has been administered the next sequential kit may be selected to administer COPE treatment. If one ampoule has already been administered and a problem is noted with the second ampoule a further COPE kit **should not** be selected. Care should continue as if both ampoules had been administered, with the use of the emergency procedures and / or unblinding as required. Both ampoules should be quarantined, segregated from the other IMP and pharmacy notified.

The quarantined kit/s should be returned to pharmacy as soon as they are next open to await further instruction (pharmacy staff will liaise with LCTC).

Once the IMP is administered, treating clinicians should **not** use further carboprost or oxytocin bolus within 15 minutes, this time represents the half-life of intravenous oxytocin, and is the time interval specified for repeat doses of carboprost. Additional pharmacological treatments, if needed, should be avoided within the 15 minutes. However, if a woman continues to experience heavy bleeding, the managing clinician may use the below treatments during this period:

PHYSICAL

- Empty the bladder, massage the uterus and check for genital tract trauma;
- Put the baby to the breast to stimulate natural oxytocin release;
- Use bimanual compression, or aortic compression.

DRUGS

- Give ergometrine (if not hypertensive);
- Start an oxytocin infusion;
- Administer intravenous tranexamic acid 1g; or
- Give misoprostol 800 micrograms sublingual or per rectum.

After the 15 minute period, further management should be conducted in accordance with NICE guidance and local practice for the management of PPH, including the use of further oxytocin (no more than 10 additional units applied as a bolus) or carboprost (no more than 7 additional doses).

Both of the ampoules in a kit should be administered so that all women are administered a deep intramuscular injection and a slow intravenous injection. For women who are recruited following caesarean section, 0.5 ml of intravenous ampoule contents will be drawn up for injection and the remaining 0.5 ml discarded. This will achieve the 5 IU oxytocin. For women recruited following vaginal birth, the entirety of the 1 ml of intravenous ampoule contents will be drawn up for injection.

For peer review only - http://bmjopen.bmj.com/site/about/guidelines.xhtml

Given the nature of the interventions in this protocol it is not anticipated a further modification in dose will be required or feasible. If, as a last resort, unblinding is necessary, this will be conducted in accordance with section 9.7.

9.4 Overdose

As part of the COPE protocol, women will be administered a single dose of carboprost or oxytocin. If administered in accordance with this protocol, overdose is unlikely. In the event that overdose is suspected, treatment should be symptomatic and supportive and there is the option to unblind if absolutely essential for further management (see also Section 9.7).

Although overdose of medication without signs or symptoms may be excluded from AE reporting this may still require investigation to ensure the protocol and regulatory requirements are met e.g. for IMP management and administration to ensure participant safety. Therefore, in such circumstances site should report immediately (within 24 hours) using the SAE form to LCTC (see section 10) and a root cause investigation should be initiated.

9.5 Delivery and Accountability of IMP at Trial Sites

9.5.1 Delivery

Trial IMP will only be delivered to an investigator site once the site has been initiated by LCTC, acting on behalf of Sponsor to ensure full ethical and regulatory approvals have been granted. The size of the shipments to each site will be pre-determined based on the participant recruitment target for that individual site.

Recruitment will be monitored centrally and drug shipment dates will be tailored accordingly to ensure that pharmacies hold adequate supplies of trial treatment. Pharmacies must document all shipment receipts and will provide copies of this documentation to the LCTC.

IMP stock must be received by a designated member of the pharmacy department and must be stored at 2-8°C with temperature monitoring as described in section 9.2.3.

9.5.2 Accountability Procedures for Study Treatment/s

Drug accountability logs will be maintained throughout (accountability and temperature monitoring records will be maintained in the receiving department within site as well as within pharmacy).

Records of all shipments must be kept in the drug accountability log.

If IMP stock received from the distributor is unexpected, wrong, damaged or out of temperature range, the stock should be quarantined and LCTC contacted for further actions.

9.5.3 Expired and unused IMP stock

Any stock that has expired at the trial site during the trial and any stock remaining at trial closedown must be notified to the LCTC, who will authorise destruction. Stock will be destroyed locally according to site policy and records made in the applicable accountability log.

For peer review only - http://bmjopen.bmj.com/site/about/guidelines.xhtml

9.6 Concomitant Medications/Treatments

9.6.1 Medications Permitted/Not Permitted/ Precautions Required

Once the IMP is administered, treating clinicians should **not** use further carboprost or oxytocin bolus within 15 minutes, this time represents the half-life of intravenous oxytocin, and is the time interval specified for repeat doses of carboprost. Additional pharmacological treatments, if needed, should be avoided within the 15 minutes. However, if a woman continues to experience heavy bleeding, the managing clinician may use other treatments (see section 9.3.2). After the 15 minute period, further management should be conducted in accordance with NICE guidance and local practice for the management of PPH, including the use of further oxytocin (no more than 10 additional units applied as a bolus) or carboprost (no more than 7 additional doses). Subsequent treatments and actions will be recorded.

9.6.2 Data on Concomitant Medication

Data on concomitant medications will not be recorded for drugs other than those meeting the criteria below. The drug, reason for use and route of administration will be recorded for the below:

- Concomitant medication implicated in an interaction with trial treatment and resulting in a Serious Adverse Event (SAE) or Suspected Unexpected Serious Adverse Reaction (SUSAR);
- Additional uterotonic use before and after the IMP;
- Blood products (with units given);
- PPH prophylactics given;
- Intrapartum oxytocin infusion used

9.7 Unblinding

Unblinding pressure-sealed envelopes are to be provided to each site. They are to be stored in a secure location accessible to trial staff, for example in a controlled drug cabinet. These are to be used to unblind the participant during the emergency, where knowledge of COPE treatment is absolutely essential for further management.

If unblinding is considered appropriate, the unblinding envelope with the randomisation number matching the participant's randomisation number should be accessed ideally after discussing with the original recruiter or if not available with a delegated member of the research team, ideally a recruiter (prescriber). Following on, LCTC should be informed initially by telephone, and then by completion and return of an unblinding CRF.

For peer review only - http://bmjopen.bmj.com/site/about/guidelines.xhtml

10 SAFETY REPORTING

10.1 Time Period for Safety Reporting

Safety reporting of Serious Adverse Events and Serious Adverse Reactions will be actively monitored and reported during the clinical trial from the time of randomisation until hospital discharge or 4 weeks, whichever is sooner.

IMPORTANT: Any safety events related to the trial treatment (see section 10.7) occurring after the end of this "active monitoring" period which meet the definition of serious (see section 10.4), related (see section 10.7) and are recorded for this study (see section 10.10) must continue to be reported by sites to the LCTC in accordance with the timeframes and procedures described in section 10.10. The same processes established for SAEs within the active monitoring period should be followed for these events.

Non-serious adverse reactions of particular interest (listed below) will be collected on a separate CRF.

Adverse reactions of particular interest:

1. Hypotension - A fall in blood pressure requiring treatment, or maternal symptoms of hypotension, developed within 2 minutes of IMP administration.

Those listed below, occurring within 2 hrs of IMP administration:

2. Vomiting
3. Pyrexia; temperature of >38°C
4. Headache
5. Hot flushes
6. Diarrhoea

Non-serious adverse events and non-serious adverse reactions (not listed within reactions of particular interest) do not require reporting.

10.2 Reference Safety Information

The Reference Safety Information (RSI) in COPE used to assess expectedness is section 4.8 of the respective Summary of Product Characteristics (SmPC):

- Oxytocin - Oxytocin 10IU/ml Concentrate for Solution for Infusion, Hameln Pharmaceuticals Ltd
- Carboprost - Hemabate Sterile Solution, Pfizer Limited

As the placebo is inert there are no expected adverse reactions, therefore any serious adverse reactions thought to be related to the placebo would be unexpected and reported as a potential SUSAR.

For peer review only

For peer review only - http://bmjopen.bmj.com/site/about/guidelines.xhtml

10.3 Flowchart of Adverse Events Reporting Requirements

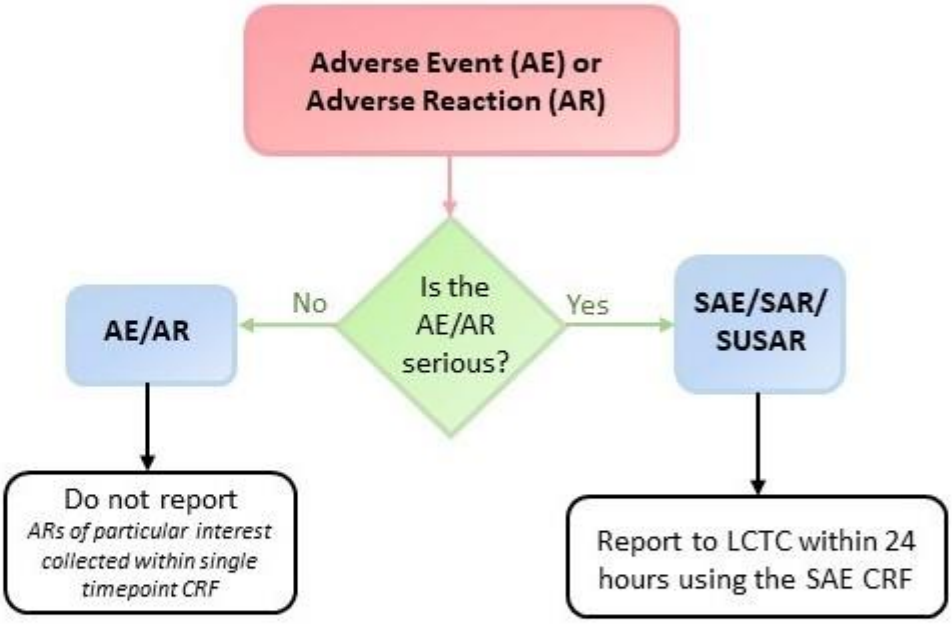

10.4 Terms and Definitions

**Adverse Event (AE)**	Any untoward medical occurrence in a subject to whom a medicinal product has been administered, including occurrences which are not necessarily caused by or related to that product.<br><br>Therefore an AE is any unfavourable or unintended change in the structure (signs), function (symptoms) or chemistry (laboratory data) in a subject to whom an IMP has been administered, including occurrences which are not necessarily caused by or related to that product.
**Adverse Reaction (AR)**	Any untoward and unintended response in a subject to an IMP which is related to any dose administered to that subject.<br><br>Therefore an AR is any unfavourable or unintended change in the structure (signs), function (symptoms) or chemistry (laboratory data) in a subject to an IMP which is related to any dose administered to that subject.

For peer review only - http://bmjopen.bmj.com/site/about/guidelines.xhtml

**Unexpected Adverse Reaction (UAR)**	An adverse reaction, the nature and severity of which is not consistent with the information about the medicinal product in question set out in the investigator's brochure or the SPC, which may be referenced where the IMP in question is a product with a marketing authorisation.
**Serious Adverse Event (SAE), Serious Adverse Reaction, or Unexpected Serious Adverse Reaction**	An adverse event, adverse reaction or unexpected adverse reaction respectively that:<br>• results in death;<br>• is life threatening (places the subject, in the view of the investigator, at immediate risk of death from the experience as it occurred (this does not include an adverse experience that, had it occurred in a more severe form, might have caused death);<br>• requires hospitalisation or prolongation of existing hospitalisation (hospitalisation is defined as an inpatient admission, regardless of length of stay, even if the hospitalisation is a precautionary measure for continued observation; hospitalisations for a pre-existing condition, including elective procedures that have not worsened, do not constitute a SAE).<br>• results in persistent or significant disability or incapacity (substantial disruption of one's ability to conduct normal life functions);<br>• consists of a congenital anomaly or birth defect;<br>• other important medical events (these may not result in death, be life-threatening, or require hospitalisation, but may be considered a serious adverse event/experience when, based upon appropriate medical judgment, they may jeopardise the subject and may require medical or surgical intervention to prevent one of the outcomes listed in this definition).
**Suspected Serious Adverse Reaction (SSAR)**	An adverse reaction that is classed in nature as serious and which is consistent with the information about the medicinal product in question set out in the case of a licensed product, in the SPC for that product.

For peer review only - http://bmjopen.bmj.com/site/about/guidelines.xhtml

**Suspected Unexpected Serious Adverse Reaction (SUSAR)**	An adverse reaction that is classed in nature as serious and which is not consistent with the information about the medicinal product in question set out in the Reference Safety Information.

N.B. “seriousness” assessment must be performed by an appropriately delegated, medically qualified member of the site research team.

10.5 Notification of deaths

All deaths in participants from the time of randomisation until hospital discharge or 4 weeks, whichever is earlier, should be reported to the LCTC using the SAE CRF **within 24 hours** of the clinical research team becoming aware of the event.

Death should be considered an outcome and not a distinct event. The event or condition that caused or contributed to the fatal outcome should be recorded as the single medical concept on the SAE CRF. Generally, only one such event should be reported. The term “sudden death” should only be used for the occurrence of an abrupt and unexpected death due to presumed cardiac causes in a participant with or without pre-existing heart disease, within 1 hour of the onset of acute symptoms or, in the case of an unwitnessed death, within 24 hours after the participant was last seen alive and stable. If the cause of death is unknown and cannot be ascertained at the time of reporting, “unexplained death” should be recorded on the SAE CRF. If the cause of death subsequently becomes available (e.g., after autopsy), “unexplained death” should be replaced by the established cause of death.

10.6 Severity / Grading of Adverse Events

The assignment of the severity/grading should be made by the investigator responsible for the care of the woman using the definitions below.

Mild	Does not interfere with routine activities
Moderate	Interferes with routine activities
Severe	Impossible to perform routine activities

A distinction is drawn between serious and severe events. Severity is a measure of intensity (see above) whereas seriousness is defined using the criteria in section 10.4, hence, a severe AR need not necessarily be a Serious Adverse Reaction.

10.7 Relationship to Trial Treatment

An assessment of the causality will be made by the investigator responsible for the care of the participant using the definitions below.

If any doubt about the causality exists, the local investigator should inform the LCTC who will notify the Chief Investigator. In the case of discrepant views on causality between the investigator and others, the opinion of the treating investigator will never be downgraded and the Medicines for Healthcare products Regulatory Agency (MHRA) will be informed of both points of view.

**Unrelated**	There is no evidence of any causal relationship. N.B. An alternative cause for the AE should be given.

For peer review only - http://bmjopen.bmj.com/site/about/guidelines.xhtml

**Unlikely**	There is little evidence to suggest there is a causal relationship (e.g. the event did not occur within a reasonable time after administration of the trial medication). There is another reasonable explanation for the event (e.g. the participant's clinical condition, other concomitant treatment).
**Possibly**	There is some evidence to suggest a causal relationship (e.g. because the event occurs within a reasonable time after administration of the trial medication). However, the influence of other factors may have contributed to the event (e.g. the participant's clinical condition, other concomitant treatments).
**Probably**	There is evidence to suggest a causal relationship and the influence of other factors is unlikely.
**Almost certainly**	There is clear evidence to suggest a causal relationship and other possible contributing factors can be ruled out.

An AE whose causal relationship to the study drug is assessed by the investigator as "possible", "probable", or "almost certainly" is an Adverse Reaction (AR).

10.8 Expectedness

There is no requirement for a reporting investigator to make an assessment of expectedness. For all SARs, the CI (or delegate) will assess for expectedness, determined by the COPE RSI (see section 10.2 for RSI). The nature, severity, or frequency of the event will be considered – if this is not consistent with that described for the type of event in the RSI current and approved at the time of the event's onset, the event will be assessed as unexpected. If **unexpected** the SAR will be reported as a SUSAR.

10.9 Follow-up After Adverse Events

All reportable adverse events should be followed until satisfactory resolution or until the investigator responsible for the care of the participant deems the event to be chronic or the participant to be stable.

When reporting SAEs, SARs or SUSARs the investigator responsible for the care of the woman should apply the following criteria to provide information relating to event outcomes: resolved; resolved with sequelae (specifying with additional narrative); not resolved/ongoing; ongoing at final follow-up; fatal or unknown.

10.10 Reporting Procedures

All SAEs and adverse reactions of particular interest should be reported for the durations described in section 10.1. Depending on the nature of the event the reporting procedures below should be followed. Any questions concerning adverse event reporting should be directed to the LCTC in the first instance. A flowchart is provided (see section 10.3) to aid in determining reporting requirements.

10.10.1 Non serious AEs

These events are not reportable for the purposes of COPE pharmacovigilance.

For peer review only - http://bmjopen.bmj.com/site/about/guidelines.xhtml

10.10.2 Non serious ARs

Only ARs of particular interest (see section 10.1), whether expected or not, should be recorded on the appropriate CRF, which should be transmitted to the LCTC within 21 days of the form being completed.

10.10.3 SAEs/ SARs/ SUSARs

All SAEs, SARs and SUSARs should be reported within 24 hours of the local site becoming aware of the event. The SAE CRF asks for the nature of event, date of onset, severity, corrective therapies given, outcome and causality. Additional information should be sent within 5 days if the reaction has not resolved at the time of reporting.

Local investigators should report any SUSARs and/or SAEs as required locally.

All deaths and overdoses should be reported as described in sections 10.5 and 9.4, respectively.

Minimum information required for reporting (CT-3)

- Valid EudraCT number (if applicable)
- Sponsor study number
- One identifiable coded subject
- One identifiable reporter
- One SUSAR
- One suspect IMP (including active substance name- code)
- A causality assessment

i. The SAE form should be completed by a designated investigator, a physician named on the 'delegation of authority and signature log' as responsible for safety reporting and making trial related medical decisions. The investigator should assess the SAE for the likelihood that it is a response to an IMP. In the absence of the designated investigator the SAE form should be completed and signed by an alternative member of the research site trial team and submitted to the LCTC. As soon as possible thereafter the responsible investigator should check the SAE form, make amendments as appropriate, sign and re-send to the LCTC. The initial report shall be followed by detailed reports as appropriate.

ii. When submitting an SAE form to the LCTC, research sites should also telephone the appropriate trial co-ordinator/data manager on telephone number **0151 795 8760** to advise that a SAE form has been submitted. The LCTC trial team should ensure that the number to be used to report SAEs in this way is manned during office hours, and is notified to research site personnel during the site initiation process.

iii. The SAE CRF should be sent by **secure** email (within 24 hours) to the LCTC:

Email: lctcsafe@liverpool.ac.uk

iv. The responsible investigator must **notify** their R&D department of the event (as per standard local governance procedures).

v. In the case of an SAE the participant must be followed-up until clinical recovery is complete and laboratory results have returned to normal, or until the event has stabilised. Follow-up may continue after completion of protocol treatment, if necessary. A final report will always be expected.

For peer review only - http://bmjopen.bmj.com/site/about/guidelines.xhtml

vi. Follow-up information is noted on another SAE form by ticking the box marked 'follow-up' and **securely** emailing to the LCTC as information becomes available. Extra, annotated information and/or copies of test results may be provided separately.

vii. The participant **must** be identified by trial number only. The participant's name **must not** be used on any correspondence.

Patient safety incidents that take place in the course of research should be reported locally by each participating NHS Trust in accordance with Trust reporting procedures.

10.10.4 Maintenance of Blinding

Systems for SUSAR and SAE reporting should, as far as possible, maintain blinding of individual clinicians and of trials staff involved in the day-to-day running of the trial. Unblinding of clinicians may be unavoidable if the information is necessary for the medical management of particular participant. The safety of participant in the trial always takes priority. In each report, seriousness, causality and expectedness should be evaluated for all of the trial treatments unless criteria have been fulfilled and unblinding has taken place (section 9.7). Cases that are considered serious, unexpected and possibly, probably or almost certainly related to one of the trial therapies (i.e. possible SUSARs) would have to be unblinded at LCTC prior to reporting to the MHRA.

10.11 Responsibilities – LCTC

The LCTC is undertaking duties delegated by the trial Sponsor, the University of Liverpool, and is responsible for the reporting of SAEs, SARs and SUSARs to the regulatory authorities (MHRA and REC) as follows:

- SUSARs which are fatal or life-threatening must be reported not later than 7 days after the LCTC is first aware of the reaction. Any additional relevant information must be reported within a further 8 days.
- SUSARs that are not fatal or life-threatening must be reported within 15 days of the LCTC first becoming aware of the reaction.
- A list of all SARs (expected and unexpected) and SAEs must be reported annually.

It is recommended that the following safety issues should also be reported in an expedited fashion:

- An increase in the rate of occurrence or a qualitative change of an expected serious adverse reaction, which is judged to be clinically important.
- Post-study SUSARs that occur after the patient has completed a clinical trial and are notified by the investigator to the LCTC/Sponsor.
- New events related to the conduct of the trial or the development of the IMPs and likely to affect the safety of the participants, such as:
    a. A SAR which could be associated with the trial procedures and which could modify the conduct of the trial;
    b. A significant hazard to the participant population, such as lack of efficacy of an IMP used for the treatment of a life-threatening disease;
    c. A major safety finding from a newly completed animal study (such as carcinogenicity).
    d. Any anticipated end or temporary halt of a trial for safety reasons and conducted with the same IMP in another country by the same sponsor;

For peer review only - http://bmjopen.bmj.com/site/about/guidelines.xhtml

- Recommendations of the IDSMC, if any, where relevant for the safety of the participants.

Staff at the LCTC will liaise with the Chief Investigator (or designated other specified in the protocol) who will review all SAEs received for seriousness and causality, and assess expectedness. Investigator reports of SAEs will be reviewed immediately and those that are SUSARs identified and reported to MHRA and REC. The causality assessment given by the Local Investigator at the hospital cannot be downgraded and in the case of disagreement, both opinions will be provided with the report.

The PIs at all institutions participating in the trial will be notified of any SUSARs.

10.12 Safety reports

Safety reports will be generated during the course of the trial which allows for monitoring of SAE and ARs of particular interest reporting rates across sites. The LCTC will send annual developmental safety update reports (DSUR) to MHRA and REC. Any concerns raised by the IDSMC or inconsistencies noted at a given site may prompt additional training at sites, with the potential for the LCTC to carry out site visits if there is suspicion of unreported SAEs/ ARs of particular interest in patient case notes. Additional training will also be provided if unacceptable delay in safety reporting timelines. If any safety reports identify issues that have implications for the safety of trial participants, the PIs at all institutions participating in the trial will be notified.

10.13 Urgent Safety Measures

An urgent safety measure (USM) is a procedure not defined by the protocol, which is put in place prior to authorisation by the MHRA and REC in order to protect clinical trial participants from any immediate hazard to their health and safety.

The LCTC will notify the MHRA and REC immediately and, in any event, within 3 days that such a measure has been taken and the reasons why it has been taken. The initial notification to the MHRA will be by telephone (ideally within 24 hours) and a notice in writing will be sent within 3 days, setting out the reasons for the USM and the plan for further action. After discussion with the MHRA and REC, further action will be agreed, which may include submission of a substantial amendment, a temporary halt, or permanent termination of the trial.

If the study is temporarily halted it may not recommence until authorised to do so by the MHRA and REC. If the study is permanently terminated before the date specified for its conclusion (in the original applications to MHRA and REC), the sponsor should notify the MHRA and REC within 15 days of the date of termination by submitting the formal End of Trial Notification.

10.14 Out-of-hours Medical Cover

Out-of-hours medical cover will use the standard out-of-hours care available within the hospital.

For peer review only - http://bmjopen.bmj.com/site/about/guidelines.xhtml

11 STATISTICAL CONSIDERATIONS

11.1 Introduction

A full and detailed statistical analysis plan (SAP), following ICH E9 and the CONSORT guidelines, will be developed prior to the first comparative monitoring report to be presented to the IDSMC (22). The main features of these planned statistical analyses are detailed below.

11.2 Method of Randomisation

The randomisation list will be generated by a statistician at the LCTC (independent to the COPE trial). Sequentially numbered treatment kits, detailing the randomisation number, will be stored in a secure location in the delivery areas. Upon randomisation the research team will select the next sequentially numbered kit for the particular mode of birth.

Participants will be randomised to carboprost or oxytocin in a 1:1 ratio.

11.3 Sample Size calculation

To detect a 2.3% reduction in a 5.8% transfusion rate (relative risk 0.60) using a Fishers exact test with 90% power (alpha 0.05), we would require 1,880 participants per group, increasing to 3,948 allowing for 5% loss to follow-up.

The magnitude of treatment effect for the primary outcome is needed in order to change long established clinical practice, and to represent a significant advantage, given the carboprost cost and side effects.

Data from sites and published literature provide a range of transfusion rates, the variation of which may be explained by the time point of randomisation. The table below demonstrates the impact of varying the event rate within this range on the study power, all other parameters including the relative effect size are maintained. Therefore, even if the transfusion rate was the lowest observed the study would still have good power. However, the most relevant and accurate data is from the Liverpool audit and we have based our sample size calculations accordingly.

It is planned to recruit equal numbers of caesarean and vaginal births to ensure that study results are convincing across both subgroups. This is to ensure that the study has the potential to impact clinical practice across both modes of delivery. Within each subgroup we would be able to detect a decrease in transfusion rate from 5.8% to 3.0% with 85% power (alpha=0.05, fishers exact test).

Transfusion rate (%)	Power (%)
3.5	71
4.0	77
5.8	90
7.5	96
10	99

Relative effect size 0.60, alpha=0.05, fishers exact test

For peer review only - http://bmjopen.bmj.com/site/about/guidelines.xhtml

Repeat uterotonic is an important secondary outcome and this sample size will provide 87% power, using a chi-square test, to detect a 4.2% reduction from a control group rate of 23.7%.

11.3.1 Feasibility (attaining recruitment targets)

In order to continue to the main trial, certain criteria should be satisfied as specified in section 3.

11.4 Interim Monitoring and Analyses

Formal interim analyses of the accumulating data, overall and split by delivery method, will be performed at regular intervals (at least annually) for review by the IDSMC. These analyses will be performed at the LCTC. The IDSMC will be asked to give advice to the Trial Steering Committee (TSC) and Trial Management Group (TMG) on whether the accumulated data from the trial, together with results from other relevant trials, justifies continuing recruitment of further patients or further follow-up. A decision to discontinue recruitment, in all participants or in selected subgroups will be made only if the result is likely to convince a broad range of clinicians including participants in the trial and the general clinical community.

11.5 Analysis Plan

The primary analysis will follow the intention to treat principle as far as practically possible, such that for all women receiving a drug intervention for PPH and for whom the outcome is known will be included in their randomly allocated group regardless of intervention received. All analyses will be conducted using a 5% two-sided level of statistical significance and 95% confidence intervals throughout.

The primary analysis will use negative binomial regression with treatment group and mode of delivery as fixed effects, and site fitted as a random effect. While the primary analysis will include all women with an indicator for mode of delivery, secondary subgroup analyses will be presented for each mode.

The secondary outcomes will be analysed using negative binomial regression for binary outcomes and linear regression for continuous outcomes. The CEQ will be scored according to its scoring manual and analysed using linear regression. A blind review will be undertaken prior to database lock which will assess the suitability of the planned analysis methods.

Baseline characteristics will be presented using descriptive statistics only; there will be no statistical tests between randomised groups.

Adverse Reactions (ARs) and Serious Adverse Reactions/Suspected Unexpected Serious Adverse Reactions (SARs/SUSARs) will also be presented using descriptive statistics only.

11.5.1 Health Economic Analysis

Total costs from the perspective of the NHS and Personal Social Services will be combined with the measure of health outcome to calculate the incremental cost-effectiveness (utility) ratios (ICER) of carboprost vs oxytocin. Where appropriate, missing resource use or health outcome data will be imputed. The number of QALYs experienced by each patient will be calculated as the area under the curve, using the trapezoidal rule, and corrected for 24-hour measurement. We will employ parametric approaches for analysing cost and QALY data that assume normal distributions given the large samples where the near-normality of sample means is approximated. Stratified cost-effectiveness analyses will be conducted on important, pre-specified patient subgroups. Estimates of Incremental cost-effectiveness ratios (ICERs)

For peer review only - http://bmjopen.bmj.com/site/about/guidelines.xhtml

will be compared with the £20,000 to £30,000 per QALY threshold of cost-effectiveness specified by NICE, and a range of sensitivity analyses will be conducted to assess the robustness of the analysis. The joint uncertainty in costs and benefits will be presented as cost-effectiveness acceptability curves.

A full Health Economics Analysis Plan will be completed prior to conducting the analysis.

11.5.2 Qualitative data analysis

Interviews and audio recordings will be transcribed, checked and anonymised as the study progresses. Qualitative researchers will lead the analysis of data. QSR NVivo software will be used to assist in the organisation and indexing of qualitative data. Framework analysis will be used to assist the management and interpretation of data (23). The focus will be modified to fit with the criterion of catalytic validity, whereby findings should be relevant to future research and trial recruitment practice (24, 25).

For peer review only

12 REGULATORY AND ETHICAL APPROVALS

12.1 Statement of Compliance

Statement of compliance: The study will be carried out in accordance with:

- The World Medical Association Declaration of Helsinki (1996)
- LCTC SOPs
- The template content structure is consistent with the SPIRIT (Standard Protocol Item: Recommendations for Interventional Trials 2013) (26)
- UK Policy Framework for Health and Social Care Research 2017.
- Medicines for Human use (Clinical Trials) Regulations 2004 (as amended)

12.2 Regulatory Approval

This trial falls within the remit of applicable trials legislation. This trial has been registered with the MHRA and has been granted a Clinical Trial Authorisation (CTA). The EudraCT number and CTA reference are displayed on the protocol cover page.

12.3 Ethical Approval

The trial protocol has received the favourable opinion of the Research Ethics Committee (REC) but must undergo independent review by the Health Research Authority and capacity and capability assessment at the R&D offices at participating sites.

12.3.1 Ethical Considerations

The study will abide by the principles of the World Medical Association Declaration of Helsinki (1964) and subsequent revisions from 1975 (Tokyo, Japan), 1983 (Venice, Italy), 1989 (Hong Kong), 1996 (Republic of South Africa).

Two key ethical issues have been identified in COPE and our approach has been informed by our Patient & Public Involvement (PPI) activities. These are the consent process and the administration of a placebo intramuscular injection to those allocated to the active oxytocin arm or a placebo intravenous injection for those allocated to the active carboprost arm.

We have undertaken discussions with consumers on the use of placebo injections. These discussions support our study design with a clear consumer preference to the use of a placebo injection to increase the reliability of the answer to the research question. The approaches undertaken are compliant with the required regulations and national guidelines, and fully justifiable. We therefore are cognisant of these issues but believe that the approaches will not raise ethical concerns.

Consent procedures for this emergency study follow the recommendations of consumer groups, the Royal Colleges, and are in compliance with ethical and regulatory frameworks. Information about the study will be distributed to target women during pregnancy through posters, leaflets, websites and on social media. As described in section 6.3, those women who bleed after childbirth and meet the eligibility criteria will be randomised into the trial in the emergency situation where treatment needs to be given urgently and there is no time for prior consent. There will also be the opportunity for prior consent for those at high risk. This approach has been successfully used in several recent trials of emergencies in labour (6, 8, 9).

For peer review only - http://bmjopen.bmj.com/site/about/guidelines.xhtml

12.4 Health Research Authority (HRA) Approval

In order for the study to take place within NHS, an approval from the Health Research Authority (HRA) will be required. This is coordinated by LCTC.

12.4.1 Capacity and capability assessment

The local Research & Development (R&D) offices should be sent the appropriate site-specific information packs complete with the necessary authorisation signatures, plus any other documentation requested for review. This review can take place at the same time as the HRA review. A copy of local R&D capacity and capability assessment should be forwarded to LCTC before the site is initiated and patients recruited.

12.5 Protocol Deviation and Serious Breaches

A breach of the protocol or GCP is ‘serious’ if it meets the regulatory definition of being “likely to affect to a significant degree the safety or physical or mental integrity of the trial participants, or the scientific value of the trial”. All serious breaches of GCP or protocol will be reported to the MHRA and REC in an expedited manner by the LCTC.

If any persons involved in the conduct of the trial become aware of a potential serious breach, they must immediately report this to the LCTC who will in turn notify the sponsor. The sponsor will assess the breach and determine if is meets the criteria of a ‘serious’ breach of GCP or protocol and therefore requires expedited reporting to the MHRA and REC.

In determining whether or not the breach is likely to affect to a significant degree the safety, physical or mental integrity of participants, the sponsor may seek advice from medical expert members of the TMG and/or of the independent oversight committees (IDSMC and TSC). In determining whether or not the breach is likely to significantly affect the scientific value of the trial, the Sponsor may seek advice form the Trial Statistician. However, the sponsor retains responsibility for the assessment of whether or not a breach meets the definition of ‘serious’ and is subject to expedited reporting to MHRA and REC.

Breaches confirmed as ‘serious’ will be reported to the MHRA and REC within 7 days by the LCTC and notified to the TMG, IDSMC and TSC at their next meeting.

Any requests for additional information from the sponsor, TMG, TSC, IDSMC, REC or MHRA, will be promptly actioned by the relevant member(s) of the research team and open communication will be maintained to ensure appropriate corrective actions are taken and documented.

Incidence of protocol non-compliance are recorded as protocol deviations, the incidence of which are monitored and reported to trial oversight committees.

12.6 Study Discontinuation

In the event that the study is discontinued, participating sites will be advised on any actions that are required with regards to individual women’s follow-up.

Women will otherwise be treated according to the usual standard clinical care. The process for women who withdraw early from trial is described in section 7.5.

For peer review only - http://bmjopen.bmj.com/site/about/guidelines.xhtml

13 DATA MANAGEMENT AND TRIAL MONITORING

Trial monitoring is carried out to ensure that the rights and well-being of human participants are protected during the course of a clinical trial. A risk assessment is performed for each trial coordinated by the LCTC to determine the level and type of monitoring required for specific hazards. The nature and extent of monitoring will be specific to the individual trial. Monitoring can take the form of on-site visits or central monitoring. A detailed monitoring plan will be developed to describe who will conduct the monitoring, at what frequency monitoring will be done, and what level of detail monitoring will be conducted.

Details of the monitoring to be carried out for the COPE study are included in the COPE Trial Monitoring Plan.

Trial Oversight Committees related to the monitoring of the trial are detailed in section 15.4.

13.1 Source Documents

Source data: *All information in original records and certified copies of original records of clinical findings, observations, or other activities in a clinical trial necessary for the reconstruction and evaluation of the trial. Source data are contained in source documents (original records or certified copies). (ICH E6, 1.51).*

Source documents: *Original documents, data, and records (e.g., hospital records, clinical and office charts, laboratory notes, memoranda, participants diaries or evaluation checklists, pharmacy dispensing records, recorded data from automated instruments, copies or transcriptions certified after verification as being accurate copies, microfiches, photographic negatives, microfilm or magnetic media, x-rays, participant files, and records kept at the pharmacy, at the laboratories and at medico-technical departments involved in the clinical trial). (ICH E6, 1.52).*

In order to resolve possible discrepancies between information appearing in the case report form (CRF) and any other patient related documents, it is important to know what constitutes the source document and therefore the source data for all information in the CRF.

The CRF will be considered the source document for data where no prior record exists and which is recorded directly in the CRF. A COPE source document list will be completed by each site.

Date(s) of conducting informed consent process including date of provision of patient information, randomisation number, confirmation of full eligibility by an authorised medical doctor, and the fact that the patient is participating in a clinical trial should be added to the patient's medical record chronologically.

13.2 Data Capture Methods

The study CRF is the primary data collection instrument for the study. Guidelines regarding data collection requirements, CRF completion and responding to data queries will be provided to sites.

For peer review only

13.3 Monitoring

13.3.1 Central Monitoring

Data stored at LCTC will be checked in accordance with data management and monitoring SOPs. Data clarification forms will be produced and provided to a named individual at a site (as listed on the site delegation log) in order to query data. Returned data clarification forms are filed along with the relevant CRFs after the appropriate corrections made on the database. There are a number of monitoring features in place at the LCTC, detailed in the trial monitoring plan, to ensure reliability and validity of the trial data.

13.3.2 Clinical Site Monitoring

In order to perform their role effectively, the trial coordinator (or other LCTC personnel) and persons involved in Quality Assurance and Inspection may need direct access to primary data, e.g. patient records, laboratory reports, appointment books, etc. Since this affects the patient's confidentiality, this fact is included on the Patient Information Sheet and Informed Consent Form.

13.4 Confidentiality

Individual participant medical information obtained as a result of this study is considered confidential and disclosure to third parties is prohibited without prior agreement in accordance with the Common Law Duty of Confidentiality. Agreement will be achieved during the trial consent process for disclosure by the clinical care staff to the COPE research team, and for sharing of information between the COPE team and NHS Digital and devolved nation equivalents. Medical information may also be given to the participant's wider medical team and all appropriate medical personnel responsible for the participant's welfare.

Data processing will be performed in accordance with applicable data protection legislation. The University of Liverpool and Bangor University are registered with the Information Commissioner's Office; as Data Controllers for this study, they will process data under the legal basis of performing a task in the public interest for research purposes.

The LCTC will be undertaking activities requiring the transfer of personal identifiers (e.g. name):

- Verification that appropriate informed consent for trial participation is obtained will be enabled by the provision of copies of participant's signed informed consent forms to the LCTC by recruiting centres, this requires the transfer of name data to the LCTC.
- Obtaining medical data from NHS Digital and devolved nation equivalents will require LCTC to collect NHS (or CHI) numbers and transfer them to the applicable organisations.

This transfer of identifiable data is disclosed in the Patient Information Sheet.

With the exception of the trial consent form, all other CRFs will be pseudonymised: labelled with the participant's initials and unique trial randomisation number, except for the screening CRFs, which will use participant's screening number.

Sites must ensure other trial documents are not posted in the same envelope as the consent form as there is a risk to patient confidentiality.

For peer review only - http://bmjopen.bmj.com/site/about/guidelines.xhtml

13.4.1 Hospital Episode Statistics (HES)

To obtain HES data and SMR data from NHS Digital, the NHS Wales Informatics Service information Centre and the electronic Data Research and Innovation Service, identifiable patient information will be transferred from LCTC to these NHS organisations as per section 7.4.2. This transfer is going to be disclosed in the trial patient information sheet and consent.

13.5 Quality Assurance and Control

Quality Assurance (QA) includes all the planned and systematic actions established to ensure the trial is performed and data generated, documented/recorded and reported in compliance with applicable regulatory requirements. Quality Control (QC) includes the operational techniques and activities done within the QA system to verify that the requirements for quality of the trial-related activities are fulfilled e.g. state what clinical site monitoring (and audit) is planned, if any. In accordance with the monitoring plan site visits will or will not be conducted and source verification performed, if indicated to be require as a result of central monitoring processes.

- The Trial Coordinator at the LCTC will verify appropriate approvals are in place prior to initiation of a site and the relevant personnel have attended trial specific training. A greenlight checklist will verify all approvals are in place prior to trial initiation at LCTC and the individual site.
- The trial will be conducted in accordance with procedures identified in the protocol.
- The TMG will determine the minimum key staff required to be recorded on the delegation log in order for the site to be eligible to be initiated and will monitor screening, randomisation and consent rates between centres.
- Independent oversight of the trial will be provided by the IDSMC and independent members of the TSC.
- The PI and other key staff from each centre will attend site initiation training, coordinated by the LCTC, which will incorporate elements of trial-specific training necessary to fulfil the requirements of the protocol.
- The process for consent, recruitment and randomisation will be evaluated for compliance with the protocol.
- Data quality checks and monitoring procedures will be undertaken in line with LCTC SOPs.

13.6 Records Retention

The retention period for COPE data and information is 25 years from the official End of Trial date (defined in section 7.7 above).

The PI at each investigational site must make arrangements to store the essential trial documents (as defined by ICH GCP guidelines) including the Investigator Site File, the applicable participant medical records and Pharmacy Site File, for the full length of the trial's retention period and will arrange for confidential destruction at the end of this period as instructed by the LCTC.
The PI is also responsible for archiving all relevant source documents so that the trial data can be compared against source data after completion of the trial (e.g. in case of inspection from authorities). They must ensure the continued storage of the documents, even if they, for example, leave the clinic/practice or retire before the end of required storage period. Delegation of responsibility for this must be documented in writing.

All other persons and organisations involved in the trial will be responsible for storing and archiving the parts of the TMF relevant to their delegated duties (e.g. laboratories, IMP

For peer review only - http://bmjopen.bmj.com/site/about/guidelines.xhtml

manufacturers and distributors, third-party vendors providing randomisation and IMP allocation systems, etc.).

The LCTC undertakes to archive as per their contractual requirements; documents will be archived in compliance with the principles of GCP. All electronic CRFs and trial data will be archived onto an appropriate media for long term accessible storage. Hard copies of data will be boxed and transferred to secure premises where unique reference numbers are applied to enable confidentiality, tracking and retrieval.

For peer review only

For peer review only - http://bmjopen.bmj.com/site/about/guidelines.xhtml

14 INDEMNITY

COPE is sponsored by the University of Liverpool and co-ordinated by the LCTC, University of Liverpool. The University of Liverpool has vicarious liability for the actions of its staff, when through the course of their employment they are involved in the design and initiation of clinical research, including but not limited to the authorship of the Protocol. The University of Liverpool has appropriate insurance in place to cover this liability.

As this is an investigator-initiated study, The Association of the British Pharmaceutical Industry (ABPI) guidelines for patient compensation by the pharmaceutical industry do not apply. However, in terms of liability, NHS Trust and Non-Trust Hospitals have a duty of care to patients treated, whether or not the patient is taking part in a clinical trial, and they are legally liable for the negligent acts and omission of their employees. Compensation is therefore available in the event of clinical negligence being proven.

Clinical negligence is defined as:
"A breach of duty of care by members of the health care professions employed by NHS bodies or by others consequent on decisions or judgments made by members of those professions acting in their professional capacity in the course of their employment, and which are admitted as negligent by the employer or are determined as such through the legal process".

For peer review only

15 ROLES AND RESPONSIBILITIES

15.1 Role of Study Sponsor and Study Funder

This study is funded by the Health Technology Assessment programme of National Institute for Health Research (HTA NIHR) and sponsored by the University of Liverpool. The sponsor's roles and responsibilities are outlined in the COPE collaborators agreement.

15.2 Funding and Support in Kind

Contractual agreements will be in place between sponsor and participating centres that will describe financial arrangements.

Funder(s)	Financial and Non-financial Support Given	Role
NIHR HTA	Financial support is described in the main contract.<br><br>NIHR HTA provides on-going help and support to the CI to ensure that the project progresses smoothly. Trial design, conduct, data analysis and interpretation, manuscript writing and dissemination of results are the responsibility of sponsor and their delegates, though the funder will monitor progress against key milestones via the submission of regular progress reports.<br>The funder requires to approve the initial protocol and amendments prior to submission for ethical/regulatory approval.<br>The funder requires to be provided with copies of project outputs at least 28 days before publication or presentation.	Funder

15.2.1 NHS Research costs

15.2.1.1 Per site

To reflect site personnel time on the study to fulfil the needs of the research, e.g. preparing for an attending initiation/ monitoring meetings, a per-site payment is included in the budget and detailed in the contract between sponsor and site.

15.2.1.2 Per patient recruited

Research related activity, including data collection, will be supported by a per-patient payment. In COPE the patient care pathway mirrors usual care in this acute emergency situation and data collection forms will be formatted to ensure that critical variables can be collected in the

For peer review only - http://bmjopen.bmj.com/site/about/guidelines.xhtml

busy emergency setting. However in order to support the robust recruitment and follow-up of participants, a per-patient payment is budgeted and detailed in contracts. The per-patient payment for transferred participants will be given to the recruiting hospital (see section 7.5.3).

15.2.2 NHS Support costs

This trial will be entered into the NIHR CRN portfolio and infrastructure support will be available through the CRN network.

15.2.3 Treatment costs

Study treatments will be supplied to participating sites by a central manufacturing facility.

15.3 Protocol Contributors

Name	Affiliations (organisation address)	Contribution to protocol
Professor Andrew Weeks	Sanyu Research Unit, University of Liverpool	Clinical aspects, trial design and conduct
Dr Shireen Meher	Birmingham Women's Hospital	Clinical aspects, trial design and conduct
Helen Hickey	Liverpool Clinical Trials Centre, University of Liverpool	Governance arrangements and trial design and conduct
Carrol Gamble	Liverpool Clinical Trials Centre, University of Liverpool	Statistical lead in trial design and conduct including monitoring, and analysis
Charlotte Van Netten	Liverpool Clinical Trials Centre, University of Liverpool	Governance arrangements and trial conduct
Katie Neville	Liverpool Clinical Trials Centre, University of Liverpool	QA oversight
Kerry Woolfall	University of Liverpool	Embedded study design and trial consent process
Louise Roper	University of Liverpool	Embedded study design and trial consent process
Clare Jackson	Liverpool Clinical Trials Centre, University of Liverpool	Data Management and trial monitoring oversight
Sylvia Balabanova	Liverpool Clinical Trials Centre, University of Liverpool	Governance arrangements and trial conduct
Dyfrig Hughes	Bangor University	Health Economics
Catrin Plumpton	Bangor University	Health Economics
Anna Rosala-Hallas	Liverpool Clinical Trials Centre, University of Liverpool	Statistical analyses under the supervision of the statistical team leader
Ben Hardwick	Liverpool Clinical Trials Centre, University of Liverpool	Governance arrangements and trial design and conduct

For peer review only - http://bmjopen.bmj.com/site/about/guidelines.xhtml

15.4 Trial Committees

15.4.1 Trial Management Group (TMG)

A TMG will be formed comprising the Chief Investigator, other lead investigators (clinical and non-clinical) and members of the LCTC. The TMG will meet regularly and is responsible for the day-to-day running and management of the trial. Refer to the TMG terms of reference and trial oversight committee membership document for further details.

15.4.2 Trial Steering Committee (TSC)

The TSC will consist of an independent chairperson, independent experts in the field of clinical obstetrics and a biostatistician. The role of the TSC is to provide overall supervision for the trial and provide advice through its independent Chairman. The ultimate decision for the continuation of the trial lies with the TSC. Refer to the TSC terms of reference and trial oversight committee membership document for further details.

15.4.3 Independent Data and Safety Monitoring Committee (IDSMC)

The IDSMC consists of an independent chairperson, plus 2 independent members: one who is an expert in the field of obstetrics, and one who is an expert in medical statistics.

The IDSMC will be responsible for reviewing and assessing recruitment, interim monitoring of safety and effectiveness, trial conduct and external data. The IDSMC will first convene prior to the start of recruitment and will then define frequency of subsequent meetings (at least annually).

The IDSMC will provide a recommendation to the TSC concerning the continuation of the study. Refer to the IDSMC charter and trial oversight committee membership document for further details.

For peer review only

For peer review only - http://bmjopen.bmj.com/site/about/guidelines.xhtml

16 PUBLICATION AND DISSEMINATION

16.1 Publication Policy

The results from different centres will be analysed together and published as soon as possible. Individual Clinicians must undertake not to submit any part of their individual data for publication without the prior consent of the TMG.

The TMG will form the basis of the Writing Committee and advise on the nature of publications. The Uniform Requirements for Manuscripts Submitted to Biomedical Journals (http://www.icmje.org/) will be respected. The ISRCTN allocated to this trial should be attached to any publications resulting from this trial.

The members of the TSC and IDSMC should be listed with their affiliations in the Acknowledgements/Appendix of the main publication.

16.1.1 Authorship

All publications shall include a list of contributors, and if there are named authors, these should include the trial's Chief Investigator, Statistician(s), Health Economist(s) and Trial Manager(s) involved at least. If there are no named authors (i.e. group authorship) then a writing committee will be identified that would usually include these people, at least.

The members of the TSC and IDSMC should be listed with their affiliations in the Acknowledgements/Appendix of the main publication.

16.2 Dissemination to Key Stakeholders

Trial results will be published in highly reputable peer-review journals and disseminated via scientific meetings and conferences, as well as through the trial website and any professional networks. Trial results will be disseminated regardless of the magnitude or direction of effect. The clinical trial report will be submitted within 12 months of the end of trial.

16.3 Data Sharing

Requests for data sharing following trial closure should be made to the LCTC for consideration.

For peer review only

For peer review only - http://bmjopen.bmj.com/site/about/guidelines.xhtml

17 CHRONOLOGY OF PROTOCOL AMENDMENTS

17.1 Version 1.0 (10/07/2018)

- Original approved version.

17.2 Version 2.0 (10/09/2018)

Summary of Amendments from Protocol V1.0 to Protocol V2.0		
**Protocol Section Number**	**Protocol Section Title**	**Summary of Changes**
10.8	Unblinding	• Removal of the requirement for the investigator to contact the Sponsor before unblinding.
11	Safety Reporting	• Reference to type A trial removed.<br>• Inclusion of all serious adverse events to be recorded from the time of randomisation until hospital discharge or 4 weeks, whichever is sooner.<br>• Removal of the requirement for the investigator to contact the Sponsor before unblinding.

17.3 Version 3.0 (01/05/2019)

Summary of Amendments from Protocol V2.0 to Protocol V3.0		
**Protocol Section Number**	**Protocol Section Title**	**Summary of Changes**
Front Page	N/A	• Additional reference numbers inserted.
Throughout	N/A	• References to vial amended to ampoule.<br>• All references to faxing amended to securely emailing.<br>• Other minor typographical corrections and clarifications in order to ensure consistency throughout.
2	Protocol Summary	• Addition of exclusion criterion "Has already received carboprost prophylactically for postpartum haemorrhage".<br>• Inclusion of assessment of adverse events at 24 hours, 48 hours/ hospital discharge if sooner and 4 weeks post randomisation.
3.2	Rationale	• Inclusion of reference to IaBOR study: "The only risk of this is hypotension that occurs with rapid injection, however this was not seen in a recent randomised controlled trial in which 517 women were given 10 international units of oxytocin as an i.v. bolus over 1 minute."<br>• Inclusion of reference to confidential enquiries into maternal deaths.

3.4	Outcome measures	• Clarification of pharmacological interventions included within footnote 4.
6.2	Exclusion Criteria	• Addition of exclusion criterion "Has already received carboprost prophylactically for postpartum haemorrhage".
7.5	Who is blinded to allocations	• Responsibility of Pharmacy amended to site team for periodic checks of unblinding envelopes.
8.1	Schedule for Follow-up	• Inclusion of reference to footnote 4 within schedule for follow-up table.<br>• Update to footnote 7 in line with update to section 11.1.
9.3	Embedded Mixed Methods Research – Enrolment & Procedure	• Update to upload of audio recordings to online folder for transcription purposes.<br>• Clarification of management of interviews with women and birth partners.
10.2	Formulation, Packaging, Labelling, Storage and Stability	• Amendment of wording from "COPE trial active treatments (carboprost and oxytocin) are market-authorised drugs being used within the terms of their marketing authorisation" to "COPE trial active treatments (carboprost and oxytocin) are market-authorised drugs".<br>• Reference to oxytocin as "aqueous solution for infusion" amended to "aqueous solution for intravenous injection".<br>• Storage and stability process clarified.<br>• Inclusion of more generic reference to clinical services company.
10.3	Distribution, Dispensing, Dosage and Administration of Study Treatment/s	• Distribution and Dispensing process clarified.<br>• Amendment of wording from "Both ampoules should be administered at randomisation in accordance with the respective Summary of Product Characteristics (SPC) accessed via the electronic medicines compendium (eMC)" to "Both ampoules should be administered at randomisation in accordance with local practice".
10.7	Concomitant medications/Treatments	• Removal of recording of dose and dose duration for concomitant medications.
11.1	Time Period for Safety Reporting	• Update to reportable adverse events. "Reporting of adverse reactions considered to be "possibly", "probably", or "almost certainly" related to the randomised trial treatment" amended to "adverse reactions of particular interest (safety reports that do not meet the "serious" criteria and are listed within reactions of particular interest)".<br>• Inclusion of "adverse reaction of particular interest" list.<br>• Figure 4 updated in line with safety reporting.
11.3	Notes on Adverse Reaction Inclusions and Exclusions	• Update to reportable adverse events. "Reporting of adverse reactions considered to be "possibly", "probably", or "almost certainly" related to the randomised trial treatment" amended to "adverse reactions of particular interest (safety reports that do not meet the "serious" criteria and are listed within reactions of particular interest)."
11.8	Reporting procedures	• Clarification of safety reporting procedures.

For peer review only - http://bmjopen.bmj.com/site/about/guidelines.xhtml

11.9	Responsibilities - Investigator	• Clarification of safety reporting procedures.
11.12	Safety reports	• Clarification of safety reporting procedures.
12.5	Analysis plan	• Inclusion of more detailed analysis plan.

17.4 Version 4.0 (13/07/2020)

**Summary of Amendments from Protocol V3.0 to Protocol V4.0**		
**Protocol Section Number**	**Protocol Section Title**	**Summary of Changes**
Front Page	N/A	• Title clarified.<br>• Addition of LCTC logo and CTRC and LCTU merger text.
Throughout	N/A	• All references to and addresses associated with Clinical Trials Research Centre (CTRC) updated to Liverpool Clinical Trials Centre (LCTC).<br>• Section numbering updated.<br>• "Oxytocin 10IU by slow intravenous injection over 2 minutes and 1ml placebo by deep intramuscular injection" amended to "Oxytocin (5 international units (IU) for caesarean section or 10IU for vaginal delivery) by slow intravenous injection over 2 minutes and 1ml placebo by deep intramuscular injection".<br>• All references to administration of intravenous injection updated to administration over 2 minutes.<br>• All references to the internal pilot "using data from the first five hospitals opened" amended to "using data collected during the first 6 months of recruitment"<br>• Other minor typographical corrections and clarifications in order to ensure consistency throughout.<br>• All references to "participant" amended to "woman", where necessary.<br>• All references to "emergency consent" amended to "postnatal (emergency pathway) consent".<br>• General formatting changes.
Protocol Approval	N/A	• Separation of approval signature pages.
1.0	Protocol Summary	• Embedded mixed methods research timelines updated.
1.1	Schematic of Study Design	• Schematic updated in line with oxytocin dosage changes and changes to data collection.
2.0	Introduction	• Removal of duplicated information.<br>• Embedded mixed methods research timelines updated.
3.0	Trial Design	• Pilot study clarified by addition of "Other sites may be started during this first 6 months so as to accelerate the overall recruitment timetable, but data from these will be included in the main study analysis only, not the pilot study analysis."
6.2	COPE recruitment pathways	• Schematic design updated and clarified.

6.3	Consent Procedures	• Clarification that postnatal consent (emergency pathway) is sought for disclosure of identifiable data and for follow-up.<br>• Grouping of information provision and consent in each of the recruitment pathways.
6.4	Randomisation Procedures	• Addition of: "All women randomised in to COPE (treatment kit removed from fridge following confirmation of eligibility) who go on to receive the COPE treatment or an alternative medicinal treatment for PPH should be approached for their consent and followed up as a COPE participant as per COPE protocol. If a treatment kit was removed from the fridge but a medicinal treatment for PPH was not administered (COPE treatment kit or other) then consent does not need to be sought and they should not be followed up as a COPE participant."
7.1	Schedule for follow-up	• Table updated to clarify follow-up schedule and group outcomes by CRF.
7.4.1	Childbirth Experience Questionnaire (CEQ)	• Inclusion of option to use telephone version of CEQ at 4 weeks, as well as paper version.
7.4.2	Health Economics Assessments	• "cost questionnaire" amended to "resource use questionnaire"<br>• Explanation of HES/ SMR data process simplified.<br>• "Once the analysis has been completed the HES and SMR data will be securely disposed in accordance with the CHEME and NHS organisations Data Services Commissioners (DSfC)" Amended to "Electronic HES and SMR records will be stored on secure Bangor University computer servers which meet NHS data security standards".
8.0	Embedded mixed methods research	• Clarification of timing of embedded study: "For at least the first 9 months of recruitment, we will use interviews, and questionnaires to explore the views and experiences of women recruited to the trial and their birth partners. In the five pilot sites (or alternative site if a pilot site is not open to recruitment within two months of recruitment of the first woman to the trial) we will also conduct focus groups with practitioners and audio-recorded recruitment discussions."
9.3.2	Distribution, Dispensing, Dosage and Administration of Study Treatment/s – Dosage and Administration	• Intervention use during the 15 minute post IMP administration clarified; "Once the IMP is administered, treating clinicians should not use further carboprost or oxytocin bolus within 15 minutes, this time represents the half-life of intravenous oxytocin, and is the time interval specified for repeat doses of carboprost. Additional pharmacological treatments should be avoided within the 15 minutes, where necessary. However, if a woman continues to experience heavy bleeding, the managing clinician may use the below treatments during this period"<br><br>Treatments that can be used during the 15 minute period clarified.<br><br>"After the 15 minute period, further management should be conducted in accordance with NICE guidance and local practice for the management of PPH, including the use of further oxytocin (no more than 10 additional units applied as a bolus) or carboprost (no more than 7 additional doses)."

		Section 7.5.1 – Discontinuation and section 9.6.1 – Medications Permitted/Not Permitted/ Precautions Required, have also been updated in line with this change.
9.5	Overdose	• Amendment of wording from "As part of the COPE protocol women will be administered a single dose of carboprost or oxytocin that are each within the parameters of the dose advised in their respective SPC. If administered in accordance with the relevant SPC and this protocol, overdose is unlikely." to "As part of the COPE protocol women will be administered a single dose of carboprost or oxytocin. If administered in accordance with this protocol, overdose is unlikely."
9.7	Unblinding	• Unblinding process clarified.
10.0	Safety Reporting	• Removal of non-serious adverse reactions of particular interest from monitoring and reporting period of randomisation until hospital discharge or 4 weeks, whichever is sooner. All such reactions appear within 2 hours of IMP administration, therefore will be collected on a separate single timepoint CRF.<br>• Flowchart of Adverse Events reporting requirements updated in line with the above.
10.2	Reference Safety Information	• Addition of "As the placebo is inert there are no expected adverse reactions, therefore any serious adverse reactions thought to be related to the placebo would be unexpected and reported as a potential SUSAR".
11.2	Method of Randomisation	• "Upon randomisation the research team will select the next sequentially numbered kit" amended to "Upon randomisation the research team will select the .next sequentially numbered kit for the particular mode of birth"
11.5.1	Health Economic Analysis	• "The joint uncertainty in costs and benefits will be considered through the application of bootstrapping and the estimation of cost-effectiveness acceptability curves" amended to "The joint uncertainty in costs and benefits will be presented as cost-effectiveness acceptability curves".
13.6	Records Retention	• Section simplified by the removal of ICH GCP essential document guidance.
15.3	Protocol Contributors	• Dyfrig Hughes added as a protocol contributor.
Appendix	COPE Outcomes	• Blood loss outcome: amended from "Estimated or measured vaginal blood loss in mls commencing in the first 24 hours from randomisation, up to cessation of active bleeding. The estimated blood loss will be recorded, and supported by blood loss collection and/or weighing where possible." to "Estimated or measured vaginal blood loss in mls from randomisation to cessation of first active bleeding. The estimated blood loss will be recorded, and supported by blood loss collection and / or weighing where possible."<br>• Blood loss ≥ 1000 mls outcome: Amended to "Volume of blood loss ≥ 1000 mls, as described above."<br>• Maternal death outcome: Amended from "Death of the mother within 4 weeks of the birth where postpartum haemorrhage was a contributing factor (it does not need to be the primary cause)" to

For peer review only - http://bmjopen.bmj.com/site/about/guidelines.xhtml

		"All deaths in participants from the time of randomisation until hospital discharge or 4 weeks, whichever is earlier."<br>• Hypotension definition clarified: "A fall in blood pressure requiring treatment, or maternal symptoms of hypotension, developed within 2 minutes of IMP administration".<br>• Resource use clarified "Resource use will include direct medical costs using questionnaire and hospital episode statistics. Quality-adjusted life years will be estimated using the using EQ-5D-5L; and the incremental cost effectiveness ratio calculated as the economic outcome of interest"

17.5 Version 5.0 (14/04/2021)

**Summary of Amendments from Protocol V4.0 to Protocol V5.0**		
**Protocol Section Number**	**Protocol Section Title**	**Summary of Changes**
N/A	Protocol Approval	• Sponsor signatory amended from Alex Astor to Dr Neil French.
N/A	Contact Details - Institutions	• Sponsor contact details updated.
N/A	Contact Details - Individuals	• Sponsor signatory amended from Alex Astor to Dr Neil French.<br>• Individual addresses/contact details updated, as required.
8.3	Embedded Mixed Methods Research – Enrolment & Procedure	• **Part B: Questionnaires** - Addition of "If questionnaires are not returned the embedded study team will ask women and birth partners to complete the questionnaire online at the point of arranging interviews (see below).<br>• **Part C: Interviews with women and their birth partner** - Inclusion of the option to carry out embedded study interviews online, in addition to via telephone or face-face (for participants located in the North West).<br>• Clarification that qualitative researchers will provide interviewees with a thank you letter, a high street voucher, and contact participants by email, not site staff.<br>• **Part D: Focus groups and/or interviews with the COPE practitioners** -<br>– Clarification of interviews/ focus groups format and addition of online option: "online focus group or remote interview (telephone or online via Zoom or Microsoft Teams) (whichever is possible to arrange around clinical practice).<br>– Clarification of the arrangement of interviews/ focus groups with practitioners.
10.1	Safety Reporting – Time Period for Safety Reporting	• Addition of: "IMPORTANT: Any safety events related to the trial treatment (see section 10.7) occurring after the end of this "active monitoring" period which meet the definition of serious (see section 10.4), related (see section 10.7) and are recorded for this study

		(see section 10.10) must continue to be reported by sites to the LCTC in accordance with the timeframes and procedures described in section 10.10. The same processes established for SAEs within the active monitoring period should be followed for these events."<br>• Clarification that "Non-serious adverse events and non-serious adverse reactions (not listed within reactions of particular interest) do not require reporting."
10.4	Safety Reporting – Terms and Definitions	• Serious Adverse Event (SAE), Serious Adverse Reaction, or Unexpected Serious Adverse Reaction definition clarification – Removal of "in offspring of subjects, or their partners, taking the IMP regardless of time of diagnosis" from "consists of congenital anomaly or birth defect" item.<br>• Addition of "N.B. "seriousness" assessment must be performed by an appropriately delegated, medically qualified member of the site research team."
10.7	Safety Reporting – Relationship to Trial Treatment	• Inclusion of "the opinion of the treating investigator will never be downgraded"<br>• "An AE whose causal relationship to the study drug is assessed by the investigator as "possible", "probable", or "almost certainly" is an Adverse Reaction (AR)." Moved from section 10.8 to section 10.7.
10.8	Safety Reporting – Expectedness	• Clarification of expectedness processes carried out by CI.
10.10	Safety Reporting – Reporting Procedures	• Email address updated from cope@liverpool.ac.uk to lctcsafe@liverpool.ac.uk for transfer of SAE reports from site to LCTC.

17.6 Version 6.0 (11/05/2022)

Summary of Amendments from Protocol V5.0 to Protocol V6.0		
**Protocol Section Number**	**Protocol Section Title**	**Summary of Changes**
Throughout	N/A	• Removal of the antenatal recruitment pathway
N/A	Protocol Approval	• Sponsor signatory amended from Dr Neil French to Karen Wilding
N/A	Contact Details - Institutions	• Sponsor contact details updated.
N/A	Contact Details - Individuals	• Sponsor signatory amended from Dr Neil French to Karen Wilding<br>• Individual addresses/contact details updated, as required.
3.0	Trial Design	• Clarification of the pilot study timing.
6.3	Consent Procedures	• Clarification of the use of data "Anonymous data, for the purposes of the recipients, will be sent to the LCTC for processing under public interest to allow monitoring of safety. Personal identifiable

For peer review only - http://bmjopen.bmj.com/site/about/guidelines.xhtml

		information will be subsequently sent to LCTC following written informed consent."
6.3.3	Consent Procedures – Discharge/ transfer to another hospital prior to postnatal consent	• Clarification of processes option following discharge/transfer to another hospital prior to postnatal consent and the addition of e-consent as an option.
8.1	Embedded mixed methods research	• Confirmation that recruitment to embedded mixed methods research has closed.
9.2.3	Storage and stability	• Addition of procedures for re-labelling stock that experiences an excursion over 8°C.

For peer review only

For peer review only - http://bmjopen.bmj.com/site/about/guidelines.xhtml

18 REFERENCES

1. Protocol guidance and template for use in a Clinical Trial of an Investigational Medicinal Product (CTIMP). NHS Health Research Authority Guidance 2016
2. Organization WH. Evaluating the quality of care for severe pregnancy complications. The WHO near-miss approach for maternal health. 2011.
3. Weeks A. The prevention and treatment of postpartum haemorrhage: what do we know, and where do we go to next? BJOG. 2015;122(2):202-10.
4. Knight M NM, Tuffnell D, Shakespeare J, Kenyon S, Kurinczuk JJ (Eds.) on behalf of MBRRACE-UK. Saving Lives, Improving Mothers' Care - Lessons learned to inform maternity care from the UK and Ireland Confidential Enquiries into Maternal Deaths and Morbidity 2013–15. Oxford: National Perinatal Epidemiology Unit, University of Oxford. 2017.
5. (NICE) NIfHaCE. NICE 2014. Intrapartum care: care of healthy women and their babies during childbirth. NICE Clinical guideline CG190. . 2014.
6. Pushpa-Rajah A, Bradshaw L, Dorling J, Gyte G, Mitchell EJ, Thornton J, et al. Cord pilot trial - immediate versus deferred cord clamping for very preterm birth (before 32 weeks gestation): study protocol for a randomized controlled trial. Trials. 2014;15:258.
7. Gynaecologists RCoOa. Obtaining Valid Consent to Participate in Perinatal Research Where Consent is Time Critical. Clinical Governance Advice No. 6a. London RCOG; 2016. 2016.
8. Shakur H, Elbourne D, Gulmezoglu M, Alfirevic Z, Ronsmans C, Allen E, et al. The WOMAN Trial (World Maternal Antifibrinolytic Trial): tranexamic acid for the treatment of postpartum haemorrhage: an international randomised, double blind placebo controlled trial. Trials. 2010;11:40.
9. Vernon G, Alfirevic Z, Weeks A. Issues of informed consent for intrapartum trials: a suggested consent pathway from the experience of the Release trial [ISRCTN13204258]. Trials. 2006;7:13.
10. POSTPARTUM HAEMORRHAGE, PREVENTION AND MANAGEMENT (Green-top Guideline 52) [Online]. London. [Internet]. 2009.
11. Knight M KS, Brocklehurst P, Neilson J, Shakespeare J, Kurinszuk JJ (Eds) on behlf od MBRRACE-UK. Saving Lives, Improving Mothers' Care - Lessons learned to inform future maternity care from the UK and Ireland Confidential Enquiries into Maternal Deaths and Morbidity 2009-12. Oxford: National Perinatal Epidemiology Unit, University of Oxford 2014. 2014.
12. Carvalho JC, Balki M, Kingdom J, Windrim R. Oxytocin requirements at elective cesarean delivery: a dose-finding study. Obstet Gynecol. 2004;104(5 Pt 1):1005-10.
13. Balki M, Ronayne M, Davies S, Fallah S, Kingdom J, Windrim R, et al. Minimum oxytocin dose requirement after cesarean delivery for labor arrest. Obstet Gynecol. 2006;107(1):45-50.
14. Adnan N, Conlan-Trant R, McCormick C, Boland F, Murphy DJ. Intramuscular versus intravenous oxytocin to prevent postpartum haemorrhage at vaginal delivery: randomised controlled trial. BMJ. 2018;362:k3546.
15. Balki M, Erik-Soussi M, Kingdom J, Carvalho JC. Oxytocin pretreatment attenuates oxytocin-induced contractions in human myometrium in vitro. Anesthesiology. 2013;119(3):552-61.
16. Widmer M, Blum J, Hofmeyr GJ, Carroli G, Abdel-Aleem H, Lumbiganon P, et al. Misoprostol as an adjunct to standard uterotonics for treatment of post-partum haemorrhage: a multicentre, double-blind randomised trial. Lancet. 2010;375(9728):1808-13.
17. Tuncalp O, Hofmeyr GJ, Gulmezoglu AM. Prostaglandins for preventing postpartum haemorrhage. Cochrane Database Syst Rev. 2012(8):CD000494.
18. Mousa HA, Blum J, Abou El Senoun G, Shakur H, Alfirevic Z. Treatment for primary postpartum haemorrhage. Cochrane Database Syst Rev. 2014(2):CD003249.
19. Thomas JS, Koh SH, Cooper GM. Haemodynamic effects of oxytocin given as i.v. bolus or infusion on women undergoing Caesarean section. Br J Anaesth. 2007;98(1):116-9.

For peer review only - http://bmjopen.bmj.com/site/about/guidelines.xhtml

20. Why Mothers die 1997 - 1999. The Confidential Enquiries into Maternal Deaths in the United Kingdom. 2001.
21. Mavrides E AS, Chandraharan E, Collins P, Green L, Hunt BJ, Riris S, Thomson AJ on behalf of the Royal College of Obstetricians and Gynaecologists. . Prevention and management of postpartum haemorrhage. BJOG. 2016;124:e106–e49.
22. Gamble C, Krishan A, Stocken D, Lewis S, Juszczak E, Dore C, et al. Guidelines for the Content of Statistical Analysis Plans in Clinical Trials. Jama. 2017;318(23):2337-43.
23. RITCHIE J, LEWIS J, NICHOLLS CM, ORMSTON R. Qualitative Research Practice. SAGE L, editor2013.
24. B. G. The Constant Comparative Method of Qualitative Analysis. Social Problems. 1965;12(4):436-45.
25. Strauss A CJ. Basics of qualitative research: techniques and procedures for developing grounded theory 2nd edition. Thousand Oaks, CA: Sage, 1998.
26. The SPIRIT Statement: http://www.spirit-statement.org/ [Accessed on 12/12/2017]. 2013.

For peer review only

APPENDIX 1. COPE OUTCOMES

**Objectives**	**Outcome Measure**	**Definition, time frame of assessment and procedure**		**Timepoint(s) of data collection**
**Primary Objective** To compare carboprost with oxytocin as initial treatments for women with clinically diagnosed PPH after giving birth in British hospitals.	Primary outcome			
	Blood transfusion	Any RBC blood transfusion or cell salvage of ≥ 300mls commenced any time between randomisation and 48 hours after randomisation (or hospital discharge if earlier than 48 hrs)		After hospital discharge
	Secondary outcomes			
	1. Volume of Blood transfusion	Data will be collected on total units of blood transfusion given, from randomisation up to 48 hrs (or hospital discharge if earlier).		After hospital discharge
	2. Use of a further uterotonic drug	Use of any uterotonic agent additional to IMP administered to control ongoing bleeding, from time of randomisation up to 24 hrs after randomisation.		After hospital discharge
	3. Composite outcome of any organ dysfunction	The composite includes any woman who develops one or more of the following from randomisation up to hospital discharge or 4 weeks, whichever is earlier. Composite based on the definitions used in the WHO Near-miss approach for maternal health' (2). Each of the items will also be reported separately.		After hospital discharge or 4 wks, whichever is earlier
		Renal dysfunction		
		Acute renal failure	Dialysis for renal failure or severe acute azotemia: creatinine ≥ 300 µmol/l or ≥ 3.5mg/dl	
		Oliguria non-responsive to fluids or diuretics:	A urinary output <30ml/h for 4 hours or <400ml/24h non-responsive to fluids or diuretics	
		Cardiovascular dysfunction		
		Cardiac arrest	Sudden absence of pulse and loss of consciousness or cardio-pulmonary resuscitation	
		Cardiopulmonary resuscitation	A set of emergency procedures including chest compressions and lung ventilation applied in cardiac arrest victims	
		Use of continuous vasoactive drugs:	The continuous use of any dose of dopamine, epinephrine or norepinephrine. In the context of vasoactive drugs infusion, continuous use refers to the uninterrupted infusion of a solution	

For peer review only - http://bmjopen.bmj.com/site/about/guidelines.xhtml

			containing a vasoactive drug. It is opposed to the intermittent or in bolus injection of a vasoactive drug.	
		Persistent shock	Systolic blood pressure <80mmHg that fails to respond to treatment with a fluid challenge	
		Severe hypoperfusion	Lactate >5mmol/l or 45mg/dl	
		Severe acidosis	A blood pH <7.1	
		Coagulation/haematologic dysfunction		
		Coagulopathy	Severe Acute thrombocytopenia (<50 000 platelets/ml), low fibrinogen (<100 mg/dl), prolonged prothrombin time (≥1.5x normal), or Fibtem A5 <6mm.	
		Massive transfusion	Transfusion of ≥5 units of blood or red blood cells.	
		Neurologic dysfunction		
		Prolonged unconsciousness	Any loss of consciousness lasting more than 12 hours, involving complete or almost complete lack of responsiveness to external stimuli / Glasgow Coma Scale <10.	
		Stroke	Acute death of brain cells in a localised area due to inadequate blood flow, diagnosed clinically.	
		Uncontrollable fits	Refractory, persistent convulsions. Status epilepticus.	
		Total paralysis	The complete or partial paralysis of both sides of the body. Usually, an extreme neuromuscular global weakness associated with critical illness. This condition is also known as critical illness polyneuromyopathy.	
		Respiratory dysfunction		
		Severe tachypnoea	Respiratory rate of more than 40 breaths per minute.	
		Severe bradypnea	Respiratory rate of less than 6 breaths per minute	
		Severe hypoxemia	Oxygen saturation <90% for ≥60min or PaO_2/FiO_2<200	
		Ventilation	Intubation and ventilation not related to anaesthesia	
		Acute cyanosis	Acute onset of bluish discolouration of mucous membranes and lips	
		Hepatic dysfunction		
		Severe acute hyperbilirubinaemia	Bilirubin >100 µmol/L or >6.0 mg/dL	
		Jaundice in the presence of pre-eclampsia	Acute onset yellowish discolouration of skin and sclera occurring in the presence of pre-eclampsia [blood pressure of greater than 140/90mmHg in the presence of proteinuria (+) or more on urinary dipstick or Protein-creatinine ratio > 30mg/mmol)]	

For peer review only - http://bmjopen.bmj.com/site/about/guidelines.xhtml

		Hysterectomy	Surgical removal of the uterus to treat postpartum haemorrhage or infection from any time after randomisation up to hospital discharge (or 4 weeks, whichever is earlier).	
	4. Hysterectomy	Surgical removal of the uterus any time after randomisation up to hospital discharge (or 4 weeks, whichever is earlier)		After hospital discharge
	5. Blood loss	Estimated or measured vaginal blood loss in mls from randomisation to cessation of first active bleeding. The estimated blood loss will be recorded, and supported by blood loss collection and / or weighing where possible.		After hospital discharge
	6. Blood loss $\geq$ 1000mls	Volume of blood loss $\geq$ 1000 mls, as described above.		After hospital discharge
	7. Haemoglobin	Postnatal Haemoglobin (Hb) closest to 24 hours after randomisation<br><br>Hb in non-transfused women-will be ideally obtained postnatally on the day following birth (12-36 hours post-randomisation) or at discharge, whichever is soonest. If repeated Hb measures have been obtained in the 12-36 hour window then the value obtained closest to 24 hours will be used		After hospital discharge,
	8. Shock	The presence of systolic blood pressure <80mmHg within 24 hours of randomisation		After hospital discharge
	9. Maternal death	All deaths in participants from the time of randomisation until hospital discharge or 4 weeks, whichever is earlier.		After hospital discharge
	10. Non pharmacological approach to treat or investigate bleeding	Use of any non-pharmacological approach to treat bleeding, from randomisation up to hospital discharge.<br>Such non-pharmacological interventions include: laparotomy, internal uterine tamponade with balloon or uterine packing, arterial embolization, removal of retained products.		After hospital discharge
	11. Manual removal of placenta	Manual removal of placenta required post randomisation up to hospital discharge.		After hospital discharge
	12. Adverse reactions of particular interest	1. Hypotension - A fall in blood pressure requiring treatment, or maternal symptoms of hypotension, developed within 2 minutes of IMP administration.<br><br>Adverse reactions of particular interest as listed below, occurring within 2 hrs of IMP administration:<br><br>2. Vomiting<br>3. Pyrexia; temperature of >38°C		From randomisation until hospital discharge.

For peer review only - http://bmjopen.bmj.com/site/about/guidelines.xhtml

		4. Headache<br>5. Hot flushes<br>6. Diarrhoea		
	13. Skin to skin care with baby within the first hour after birth	Where the newborn is placed unwrapped against the mother's bare chest or belly from any time after randomisation up to 1 hour after birth.		At 24 hrs
	14. Separation from new-born in first hour after birth	Separation of the newborn from the mother post randomisation up to the first hour after birth.<br>.		At 24 hrs
	15. Breastfeeding	'Breastfeeding' refers to feeding of the baby with the mother's breast milk, even if this is expressed breast milk given by cup or bottle		At 24 hrs, 48 hrs (or hospital discharge if sooner) and 4 wks
		Initiation	Breastfeeding initiated within the first 24 hours after birth.	
		Exclusively at hospital discharge	Exclusive breastfeeding at hospital discharge (i.e. no formula milk, other liquids, or food)	
		Exclusively at 4 weeks post-birth	Exclusive breastfeeding at 4 weeks post birth (i.e. no formula milk, other liquids, or food)	
	16. Childbirth experience questionnaire	Questionnaire on childbirth experience administered to women at 4 weeks postnatally.		At 4 wks
**Secondary Objective**<br>To assess relative cost-effectiveness of use of carboprost or oxytocin as initial treatments for women with clinically diagnosed primary PPH.	17. Resource use	Resource use will include direct medical costs using questionnaire and hospital episode statistics. Quality-adjusted life years will be estimated using the using EQ-5D-5L; and the incremental cost effectiveness ratio calculated as the economic outcome of interest.		At 24 hrs and 4 wks.
		QALY measured using EQ-5D-5L		
		Bespoke self-report instrument designed to capture participants' resource use.		

For peer review only

		Electronic data records of hospitalization will be requested from NHS Digital (England) and devolved equivalents, based on NHS (or equivalent) Number.<br><br>**This will include data on transfer to a higher level of care**<br>This is the transfer of the place of care **due to** a postpartum haemorrhage. This refers to any transfer for more specialist care, but examples would be a transfer from home to hospital, from one hospital to another for specialist input, from a high dependency care unit to an intensive care unit, or from a midwifery led unit to an intensive care unit.	Data will be requested following completion of follow-up from NHS Digital and devolved equivalents.

For peer review only

<Trust address 1>
<Trust address 2>
<Trust address 3>
<postcode>

<telephone number>

COPE: Carboprost vs Oxytocin as the First Line Treatment of Primary Postpartum Haemorrhage; A phase IV, double-blind, double-dummy, randomised controlled trial.

Emergency Patient Information Sheet and Consent Form

Version 1.0: 12-07-2018

Contents

Contents	Page
1) Why am I being asked after I was given the medicine?	2
2) Why are we doing the study?	2
3) What happens if I agree for you to use my information?	2
4) Do I have to take part?	3
5) How was it decided which medicine I received?	3
6) Timeline of visits	3
7) What are the alternatives for treatment?	3
8) What are the benefits and risk of taking part?	4
9) Will my participation be kept confidential?	4
10) What if there is a problem?	4
11) What will happen to the results of the study?	4
12) What are my electronic hospitalisation records?	4
13) Who is running the study?	5

Contact

Principal Investigator: **<PI NAME>**
Research Midwife: **<RM NAME>**
Telephone: **<number>**

Or visit the website: www.copestudy.uk

Information about the study

Excessive bleeding after childbirth (also known as postpartum haemorrhage or PPH) is a common problem which affects 1 in 20 women.

COPE is a research study to compare two drugs currently used to treat PPH, to decide which is better. The two drugs are **carboprost** and **oxytocin**.

Before you decide if you want to give your permission for your information to be used in this study, it is important for you to understand why the study is being done and what is involved.

Please take time to read the following information carefully and discuss it with friends or relatives if you wish. Taking part is voluntary. If you don't want to take part then you don't need to give a reason.

Your decision will not affect how the doctors and nurses will look after you.

Please ask a member of your clinical team if there is anything that is not clear, or if you would like more information.

Important things that you need to know

PPH is a medical emergency and needs to be treated without delay; it is important to prevent excessive bleeding as quickly as possible.

After you had given birth, you experienced a PPH. As this is a medical emergency, the priority is to treat the PPH and there is not time to properly discuss whether you would like to take part in COPE before giving treatment for PPH.

The ethical committee and medicines regulator have approved that women can be treated as part of the COPE research study and then approached after the medical emergency has passed to see if you are happy for us to collect some information for the research study.

For peer review only - http://bmjopen.bmj.com/site/about/guidelines.xhtml

Thank you for taking the time to read this information sheet. We hope you will find this information helpful.

Why am I being asked after I was given the medicine rather than before?

After you had given birth, you experienced a PPH.

PPH is a medical emergency and needed to be treated without delay; it was important to prevent excessive bleeding as quickly as possible. As there wasn't time to properly discuss the COPE research study we have approval from the ethical committee and medicines regulator to treat women as part of the COPE research study and then to ask for consent afterwards. This is research without prior consent (sometimes called deferred consent).

As part of the COPE study, when you experienced a PPH you were given two injections – one into the muscle and one into the vein. You were given either carboprost and placebo or oxytocin and placebo. A placebo is a salt solution containing no active drug – acting as a "dummy drug". Carboprost is injected into the muscle and oxytocin injected into the vein, therefore including a placebo as well as carboprost or oxytocin, in this way, means that neither you nor your doctor know what treatment you were given. This ensures a fair test.
We have therefore come to talk to you about the study as soon as possible after the medical emergency to ask for your permission to use information we have collected about your hospital stay.

Why are we doing the COPE study?

It is important to stop excessive bleeding after birth (PPH) as quickly as possible, as the blood loss can leave women feeling extremely weak and tired. They can then find it hard to care for their newborn baby. This hospital is one of about 40 hospitals in the UK involved in the COPE Study. About 4000 women will take part. We want to know if it is better to use carboprost or oxytocin as the first drug for treatment if a woman has a PPH.

The results from COPE will help doctors know which treatment is better for women with PPH.

What happens if I agree that you can use my information?

A member of the clinical team will talk to you in detail and you will be able to ask any questions that you have. If you have had all of your questions answered and are happy for your information to be collected then you will be asked to sign a consent form to confirm this. You will be given a copy of the consent form and the information sheet to keep.

We will collect some information about your health and we will ask you some questions about your experience in the study, your experience of childbirth and your general well-being. These questions will be asked before you leave the hospital and then again at four weeks after the birth. We will contact you via telephone, email or text to follow-up.

Embedded study: Your views on how we ask people to take part in COPE are important

You and your birth partner (if applicable) may be asked to take part in a telephone interview or face to face interview if you live in the North West and/or complete a questionnaire. Interview discussions will last about 60 minutes and will include how you felt about the COPE study. This study will inform how we ask other parents/ relatives about COPE and future related research. Again, it is up to you whether you take part or not, and your care will not be affected if you do not take part.

Do I have to take part?

No. Taking part is voluntary. If you choose to take part you can also choose to stop at any time without giving a reason. The standard of care you receive now and in the future will be the same whether you take part or not.

For peer review only - http://bmjopen.bmj.com/site/about/guidelines.xhtml

If at any point you decide to stop taking part in the study, you will receive the care usually offered by your hospital following PPH.

We will use any study information collected up until the time you stop taking part; this information will be pseudo-anonymised, meaning that identifying details, (e. g. your name) will only be accessed by people working on the study.

How was it decided which medicine I received?

In research studies we often split people up into groups to look at how different treatments work. People in one group get a different treatment than patients in another group. In the COPE study there are two treatment groups:

Group A: Women will receive oxytocin and placebo

Group B: Women will receive carboprost and placebo

It is really important that each group in the COPE study has a similar mix of patients in it so we know that if one group of patients does better than the other it is because of the treatment and not because there are differences in the types of women in each group.

We use a computer programme that puts women ‘at random’ into one of the groups – you might hear this described as ‘randomisation’ or ‘random allocation’, but they all mean the same thing. So, neither you nor your doctor chose your treatment. In the COPE study, you are equally as likely to be in the group receiving oxytocin as you are in the group receiving carboprost.

COPE is a ‘blinded study’ which means neither you nor your doctor will know what your treatment is. In the event of an emergency, the name of your treatment can be found out quickly.

Timeline of visits

Bleeding after childbirth: After the emergency is over, a member of the research team will talk to you as soon as possible about the study and the process of deferred consent. If you consent that you are happy to take part, we will use information we have collected about your hospital stay.

4 weeks after treatment: We will contact you using your preferred method of communication (telephone, text or email) to follow-up completion of a questionnaire about your quality of life and your childbirth experience.

Your views and experiences: If you have opted in, yours and your birth partner’s views and experiences of the study treatment and consent process will be explored after the emergency and before you leave the hospital, and again 4 weeks later.

What are the alternatives for treatment?

If you were not treated as part of COPE, you would have usually been given oxytocin first and then carboprost if the bleeding continued. Both are common treatments for PPH, but we do not know which is better.

What are the benefits and risks of taking part?

The results from the study will help doctors know what treatment is best to use first for PPH in the future.

The common side effects of both treatments are as follows:

Oxytocin	Carboprost
Headache	Diarrhoea
Nausea (feeling sick)	Vomiting
Vomiting	High temperature
Low blood pressure	Increased blood pressure
Water Retention	

For peer review only - http://bmjopen.bmj.com/site/about/guidelines.xhtml

Will my participation be kept confidential?

Yes. We will follow strict ethical and legal practice so that all information collected about you during the study is kept strictly confidential. With your permission, we will send a letter to your GP to let them know you are taking part and use your NHS number to access anonymised data about your hospital admissions.

Any information with your name on it (such as the consent form) that has to be transferred to the coordinating centre in Liverpool, will be sent securely and only accessed by people working on the study or working to ensure the study is being run correctly.

When you agree to take part in a research study, the information about your health and care may be provided to researchers running other research studies in this organisation and in other organisations. These organisations may be universities, NHS organisations or companies involved in health and care research in this country or abroad. Your information will only be used by organisations and researchers to conduct research in accordance with the UK Policy Framework for Health and Social Care Research.

This information will not identify you and will not be combined with other information in a way that could identify you. The information will only be used for the purpose of health and care research, and cannot be used to contact you or to affect your care. It will not be used to make decisions about future services available to you, such as insurance.

What if there is a problem?

If you have any concerns about this study, you should ask to speak with one of your research team who will do their best to answer your questions.

If you remain unhappy and wish to complain formally, you can do this through the NHS Complaints Procedure. Details can be obtained from your hospital.

Every care will be taken in the course of this research study. However, in the unlikely event that you are injured as a result of the managing organisation (University of Liverpool), compensation may be available and you may have to pay your related legal costs. Your hospital where you receive your treatment has a duty of care to you whether or not you agree to participate in the trial and the University of Liverpool accepts no liability for negligence on the part of your hospital's employees. If you are harmed and this is due to someone's negligence, then you may have grounds for a legal action for compensation against the NHS Trust where you are being treated, but you may have to pay for your legal costs.

What will happen to the results of the study?

Our results will guide clinical practice for PPH. We plan to present the results of the study at conferences and publish them in medical journals so that we can explain to the medical community what our research results have shown. Confidentiality will be ensured at all times and you will not be identified in any publication.

What are my electronic hospitalisation records?

The NHS organisations routinely collect information about your inpatient, outpatient and any Accident & Emergency hospital care. In England and Wales the data are referred to as 'Hospital Episode Statistics' or HES data, whilst in Scotland the data are referred to as 'Scottish Morbidity Records' or SMR data; these are regarded as a special category of information.

The study team will retrieve information from your electronic hospitalisation records held by NHS Digital (https://digital.nhs.uk) for patients in England; NHS Wales Informatics Service Information Services (http://www.infoandstats.wales.nhs.uk/) for patients in Wales; and the electronic Data Research and Innovation Service (http://www.isdscotland.org/Products-and-Services/EDRIS/) for patients in Scotland.

Researchers at Bangor University, who are part of the COPE study, want to use your HES and SMR data to

peer review

For peer review only - http://bmjopen.bmj.com/site/about/guidelines.xhtml

calculate the overall costs of care. These data will be collected for the period from three months prior to the start of the study and for the duration of the study. To retrieve your HES records, information to identify you including your name, date of birth, NHS (or CHI) Number and COPE Study Number, will be securely transferred by the COPE research team to each of the organisations listed above. Towards the end of the study, they will in turn, securely transfer your pseudo-anonymised HES or SMR records to Bangor University using an encrypted electronic transfer system. The data is referred to as 'pseudo-anonymised' data because whilst all your identifying details (name, date of birth, postcode, NHS (or CHI) Number) will have been removed, there is still a unique COPE Study Number present in the data should COPE researchers need to refer back to your identifying details and contact you for any reason.

Your electronic HES and SMR records will be stored on secure Bangor University computer servers which meet NHS data security standards. Researchers at Bangor University will not be able to access your identifying details and will not keep the pseudo-anonymised HES or SMR data longer than 12 months. It will then be returned to the University of Liverpool for long term storage.

Who is running the study?

The University of Liverpool is the sponsor for this study based in the United Kingdom. The University is responsible for managing this study whilst the University's Clinical Trials Research Centre (CTRC) runs it on a day-to-day basis. This study is funded by the National Institute for Health Research (NIHR)'s Health Technology Assessment (HTA) programme.

The study has been reviewed by the funder and has been approved by the Health Research Authority, National Research Ethics Service Committee (<MAIN REC>) and the Medicines and Healthcare products Regulatory Agency.

The University of Liverpool will be using information from you and your medical records in order to undertake this study and will act as the data controller for this study. This means that we are responsible for looking after your information and using it properly. The University of Liverpool will keep identifiable information about you for 25 years after the study has finished.

Your rights to access, change or move your information are limited, as we need to manage your information in specific ways in order for the research to be reliable and accurate. If you withdraw from the study, we will keep the information about you that we have already obtained. To safeguard your rights, we will use the minimum personally-identifiable information possible.

You can find out more about how we use your information at www.copestudy.uk

Your NHS hospital will collect information from you and your medical records for this research study in accordance with our instructions.

Your NHS hospital will use your name, NHS number and contact details to contact you about the research study, and make sure that relevant information about the study is recorded for your care, and to oversee the quality of the study. Individuals from the University of Liverpool and regulatory organisations may look at your medical and research records to check the accuracy of the research study. Your NHS hospital will pass these details to the University of Liverpool along with the information collected from you and your medical records. The only people in the University of Liverpool who will have access to information that identifies you will be people who need to contact you to in relation to the study or audit the data collection process. The people who analyse the information will not be able to identify you and will not be able to find out your name, NHS number or contact details.

Your NHS hospital will keep identifiable information about you from this study for 25 years after the study has finished.

For peer review only - http://bmjopen.bmj.com/site/about/guidelines.xhtml

Thank you for reading this information sheet.

Contacts for further information

If you would like more information or have any questions about the COPE study please talk to:

Principal Investigator: **<PI NAME>**
Research Midwife: **<RM NAME>**

Telephone: **<number>**
Or visit the website: www.copestudy.uk

If you wish to discuss the study with someone independent of the research team you can contact the local NHS Patient Advice and Liaison Service (PALS) on: <telephone number>

For peer review only

For peer review only - http://bmjopen.bmj.com/site/about/guidelines.xhtml

Consent Form

To be completed by the Researcher:

Site Name:	
Participant Study Number	
Participant Initials:	Participant DOB: / /
NHS / CHI Number	
Postcode	

To be completed by the patient:

	Tick	Initial
**Once you have read and understood each statement please tick (✓) and initial.**<br>Example: I confirm that I have read and understand the Participant Information Sheet.	✓	JS
1. I have read and understood the information sheet for this study. I have had the opportunity to ask questions and have these answered satisfactorily.		
2. I understand that I have already received the study treatment for PPH.		
3. I understand that participation is voluntary and that I am free to withdraw from the study at any time, without giving a reason, and without my care or legal rights being affected.		
4. I understand that my data will be retained for a maximum of 25 years by the site and by the Clinical Trials Research Centre (CTRC), part of the University of Liverpool, and that they will be stored in a confidential manner.		
5. I understand that relevant written and electronically-recorded sections of my medical notes, records and any data collected during the study may be looked at by authorised individuals from the research team and those listed under 'Who is running the study?' section above, NHS Trust and Regulatory Authorities where it is relevant to my taking part in research. I give permission for these individuals to have access to these notes, my records and data.		
6. I understand that the data collected for this study may be used in a pseudo-anonymised form for related research.		
7. I give permission for a copy of this consent form which will include my name, date of birth and NHS number to be sent to the CTRC (where it will be kept in a secure location), to allow confirmation that my consent was given.		
8. I agree to my GP being informed of my participation in the study.		
9. I agree for my name, date of birth, NHS (or CHI) Number, COPE Study Number and a copy of this consent form to be shared with NHS Digital or their UK devolved equivalent so they can link this information to Hospital Episode Statistics (HES), or Scottish Morbidity Records (SMR) containing my details and provide members of the COPE study team working on the trial with HES and SMR data regarding my hospital attendances.		
10. I agree to take part in the above study.<br>My preferred method of communication for follow – up is (please tick as appropriate):<br>**Telephone** ☐ **Email** ☐ **Text** ☐		

For peer review only

Consent Form

To be completed by the Researcher:

Site Name:			
Participant Study Number			
Participant Initials:		Participant DOB:	/ /
NHS / CHI Number			
Postcode			

**Contact details:**	
**Telephone number:**	
**Email address:**	

Below are embedded study statements:

11. I agree to being contacted to take part in an embedded study interview.
12. I agree to completing an embedded study questionnaire.
13. I agree for the audio recording of my consent discussion with the COPE midwife/doctor to be used in the embedded study.

Below are optional study statements:

14. I agree that I may be contacted in the future in relation to this or other relates studies.

Your **full** name (please print):			
Your signature:		Date:	dd / mm / yyyy

To be completed by the Researcher:			
Researcher **full** name (please print):			
Researcher signature:		Date:	dd / mm / yyyy

To be completed by the Translator (if used):			
Translator **full** name (please print):			
Translator signature:		Date:	

For peer review only

For peer review only - http://bmjopen.bmj.com/site/about/guidelines.xhtml

COPE
The Carboprost or Oxytocin Postpartum haemorrhage Effectiveness Study

Trial number: ☐☐ ☐☐

COPE Study: postnatal questionnaire

Carboprost vs Oxytocin as the First Line Treatment of Primary Postpartum Haemorrhage.

The following questions are about the COPE study discussion and consent process that you/your partner took part in. We refer to people agreeing to take part in research as 'consenting'. We are interested in when you/ your partner were approached to discuss COPE and what you thought of the information you were given.

a. Date of birth: ☐☐/☐☐/☐☐☐☐

b. Today's date: ☐☐/☐☐/☐☐☐☐

c. First part of your postcode: ___ ___ ___ ___

d. Are you:

Male ☐ or female ☐

Patient (Mum) ☐ or Birth partner ☐ Please describe your relationship to the patient in the box below (e.g. husband, wife, partner, mother, friend)

☐

e. Have you/your partner had a postpartum haemorrhage in a previous pregnancy

Yes ☐ No ☐ I don't know ☐ Not applicable ☐

f. When did a doctor or midwife first speak to you about COPE? (Please tick all that apply)

1) During an antenatal appointment ☐

2) When I was bleeding before treatment was given ☐

3) After I had have been given treatment for bleeding ☐

4) I can't remember ☐

For peer review only - http://bmjopen.bmj.com/site/about/guidelines.xhtml

1. *Please indicate how strongly you agree or disagree with the following statements by placing a circle around the answer that best fits your opinion*

Statements	Agree	Neither agree nor disagree	Disagree
a. The doctor or midwife checked that it was a convenient time to discuss research before discussing COPE	1	2	3
b. The information I received about COPE was clear and straightforward to understand	1	2	3
c. I had enough opportunity to ask questions about COPE	1	2	3
d. I was satisfied with the consent process for COPE	1	2	3
e. It was difficult to take in the information I was given about COPE	1	2	3
f. It was difficult to make a decision about COPE	1	2	3
g. I made this decision	1	2	3
h. It was a joint decision (e.g. between patient and birth partner)	1	2	3
i. Someone took this decision away from me	1	2	3
j. I was not in control of this decision	1	2	3
k. The decision about the research was inappropriately influenced by others	1	2	3

If the answer to this question is 'Agree', please state who you think influenced the decision about the research:

____

For peer review only

For peer review only - http://bmjopen.bmj.com/site/about/guidelines.xhtml

2. *Did you/your partner consent for your participation in COPE?*

◯ Yes (Go to Question 3) ◯ No (Go to Question 4)

3. *What were your reasons for providing consent for your participation in COPE?*

Please tick all that apply <u>and then circle your main reason</u> (e.g. ✔)

a. To help me/my partner	☐
b. To help other women in the future	☐
c. I felt that medical studies like COPE are important	☐
d. Because I trusted the doctor or midwife who explained COPE	☐
e. The treatment had already been given to me/my partner	☐
f. The treatment worked	☐
g. I didn't feel comfortable saying no to the doctor or midwife who explained the study	☐
h. Other (Please state): ______________________________	☐

4. *If you did not provide consent, please provide your reasons for deciding that you/your partner would not take part in COPE*
(If you do not wish to do so, please leave this space blank)

5. *Please tell us any comments or suggestions you have to improve the recruitment and consent process for COPE:*

We would like to thank you for taking the time to complete this questionnaire.

Please place the questionnaire in the envelope provided, <u>seal it and give it back to the doctor or study midwife.</u>

For peer review only - http://bmjopen.bmj.com/site/about/guidelines.xhtml

Version 1.0 19.01.2018
IRAS ID: 235254

COPE: Interview Topic Guide- Patient

Introduce self, thank participant for taking part and confirm happy to take part in an interview. **Remind participants of broad aims: We are interviewing women and their partners who have been asked to participation in the COPE study to find out experiences of women, their birth partners and practitioners involved in recruitment and consent in COPE. Findings from interviews will be used to inform how we conduct the rest of the trial.**

Section 1: Introduction, setting ground rules

Discuss the following issues:

- Review the nature and purpose of the research.
- No right or wrong answers, aim to understand experiences of recruitment and consent.
- Confidentiality, use of data.
- Explain the use of data recorder, transcription, use of pseudonym (invite to choose), use of verbatim quotes, will be taking field notes.
- Researcher aware that discussion might bring up difficult memories, explain can decline to answer any question or prompt; can ask to stop at any time if feels need to.
- Expected duration of interview.
- Check consent form signed
- Ask if any questions.

Bereaved mothers: I have some idea about your circumstances, if there is anything that you find difficult to talk about please don't feel that you have to. If you want to stop the interview at any point, then please let me know.

I will start with some questions about you if that's ok and then I will ask you about your experience of being invited to take part in the COPE study?

Check recorder working

Introduce and switch on tape recorder

Section 2: demographics

To start we have a couple of general questions about you if that's ok?

1. Could you please tell me your age?
2. Before your maternity leave were you in employed or unemployed? If employed, what was your occupation?

For peer review only - http://bmjopen.bmj.com/site/about/guidelines.xhtml

3. What is the first part of post code?
4. What would you describe as your ethnic background?
5. How many children do you have?

Section 3: Antenatal awareness and COPE recruitment

1. **When did you first hear about the COPE study?**

2. **Were you provided with information about the COPE study during an antenatal visit?**

-IF NO

- **Did you see any leaflets or posters about the COPE Study during your pregnancy?** Explore location, views on the information; whether they requested further details, who from, what questions did you have, where they answered sufficiently.

Go to Section 4

IF YES

- Who discussed COPE with you (role)
- Could you tell me what they explained about the COPE study?
- Did they go through any of the potential risks or benefits of taking part in the study at that point? If yes, how did they describe these?
- Was there anything that you found: a) unclear b) surprising?
- Is there anything else that sticks out in your mind about the discussion?
- (*Prompt- if study aims not described by participant in response to the second point above*) This next question is not a test. Just so we can gage whether the trial is being explained clearly to women. Could you tell me what you understood the study was about?
- Do you have any suggestions about how this discussion could be improved in the future? If yes how?
- Could you tell me about any written information you were given about the COPE study? Explore whether they were given the information leaflet – short version and/or the full patient information sheet
- When did you receive this information? (Prompt: explore written and verbal and time point)
- Did you read the information leaflet/sheet? (Prompt: If they read the short information PIS leaflet or the full information PIS)
- What did you think about the information leaflet/sheet?
- Was there anything that you found: a) unclear? b) surprising?

For peer review only - http://bmjopen.bmj.com/site/about/guidelines.xhtml

- Could the information leaflet be improved in any way? (Prompt: If so, how?)

<u>**Section 4: birth and PPH experience (**for bereaved mothers introduce this section and restate that</u> if there is anything that you find difficult to talk about please don't feel that you have to).

1. Please could you tell me about your labour and birth? (Explore: mode of delivery- vaginal birth, caesarean birth, emergency, planned, water birth, general anaesthetic etc.)
2. How did you feel during labour and birth?
3. After birth were you aware that you were losing a lot of blood.
   - *Explore what alerted you to the problem?*
   - *What were you told, by whom about the loss of blood.*
   - *Could you tell me what happened?, how long after birth?*
   - *How did you feel, any worries or fears, how did you manage your emotions?*
4. Can you remember if you were given any treatments to stop the bleeding? *(If can recall explore awareness of what drug, if it did stop the bleeding, other treatments given)*
5. (if applicable) Did you have a PPH with other births?

<u>**Section 5. COPE first approach (birth)**</u>

1. **Could you tell me at what point did someone first mention the COPE study? For example, did a midwife or doctor briefly speak to you about COPE before a treatment was given to help stop your bleeding?**

IF YES (e.g. verbal consent at time of PPH)

- Could you tell me a bit more about that? *Explore who and when,* did they speak to you or (partner name) or was this a joint discussion?
- Could you tell me how were you feeling at that point in time?
- Can you remember how you responded? Explore if consented, declined, did you ask any questions?
- Is there anything that sticks out in your mind about the discussion?
- Do you think that this was the best time to discuss the study with you?
- If not, when would you have preferred to have the discussion about COPE? *Explore views on seeking consent after the trial drugs have been given.*
- *Did someone come and speak to you a second time about COPE after your birth?*

IF NO (e.g. consent within 24 hours or can't remember)

- How would you have felt if the doctor or midwife had spoken to you at that point in time? (*Explore acceptability of seeking verbal consent during PPH, would you have been able to make a decision about taking part in COPE at that point in time?)*
- When do you remember a midwife or doctor first speaking to you about the COPE study? *(Explore who discussed COPE and whether person familiar to participant, whether it*

For peer review only - http://bmjopen.bmj.com/site/about/guidelines.xhtml

Version 1.0 19.01.2018
IRAS ID: 235254

matters to the participant who approached her, timing of approach, whether this was an appropriate time and if timing was checked).

Section 6. COPE full recruitment conversation

1. **Could you tell me what they explained to you about the COPE study? (explore level of involvement in trial discussions) If already consented antenatally explore level of discussion- this could have just been a checking consent so questions below may be too detailed).**
2. **Did they go through any of the potential risks or benefits of taking part in the study at that point?** *If yes, how did they describe these?*
3. **Was there anything that you found: a) unclear b) surprising?**
4. **Is there anything else that sticks out in your mind about the discussion?**
5. **This next question is not a test. Just so we can gage whether the trial is being explained clearly to women. Could you tell me what you understood the study was about?**
6. **Do you have any suggestions about how this discussion could be improved in the future?** *If yes how?*
7. **Did you discuss the decision to be part of COPE with anyone else?** *Explore who, did their views influence your decision? Shared decision making*

Section 7. Written information (as above, if antenatally see if information given again)

1. **Could you tell me about any written information you were given about the COPE study?** *Explore whether they were given the information leaflet – short version and/or the full patient information sheet*
2. **When did you receive this information?** *(Prompt: explore written and verbal and time point)*
3. **Did you read the information leaflet/sheet?** (*Prompt:* If they read the short information PIS leaflet or the full information PIS)
4. **What did you think about the information leaflet/sheet?**
5. **Was there anything that you found: a) unclear? b) surprising?**
6. **Could the information leaflet be improved in any way?** *(Prompt: If so, how?)*

Section 8. Research without prior consent

Explain: Some women were not provided with COPE study information and invited to consent during an antenatal visit as they were not anticipated to be at high risk of a PPH. This meant that when they had a PPH they were included in the study without having a full discussion about the trial and invited to provide consent. This is research without prior consent, also known as deferred consent. Legislation allows research without prior consent in emergency situations as there is no time to have a discussion about the research and that actually having that discussion might delay vital treatment.

For peer review only - http://bmjopen.bmj.com/site/about/guidelines.xhtml

1. **What do you think of the use of research without prior content in emergency situations?**
2. **What do you think of the use of RWPC in the COPE study?**

ONLY IF RWPC approach used

3. **How long did you get to think about whether you wanted your information to be used in COPE?** *Do you think this was long enough?*
4. **Did you have the opportunity to ask any other questions about the study?**
5. **Did you ask any?** *What questions did you ask?, if not, why not*
6. **How long do you think people should be given to think about consenting to the use of their information in the trial?**
7. **In making the decision about your participation in COPE, what sort of things went through your mind?**
8. **Was there anything you found particularly helpful in making up your mind?**
9. **Was there anything you found unhelpful?**
10. **How hard was this decision?**
11. **Was there anything specific that influenced your decision**?
12. **Would you mind telling me what were your reasons for (providing consent/not providing consent)?**
13. **How do you feel about participating in this research?**
    - *Explore experiences of the research process e.g. recruitment, completing questionnaires, contact with research team*
    - *Ask how they feel about participating in this interview*

    Is there anything else you might want to add?

At the close of the interview briefly summarise the main points to confirm interpretation with the participant. Ask if they wish to expand any responses or add anything else to the discussion.

- *Ensure participant has contact details for the local research team should they wish to discuss any aspect of the study.*
- *Complete reflexive diary/field notes.*

For peer review only - http://bmjopen.bmj.com/site/about/guidelines.xhtml

COREQ (COnsolidated criteria for REporting Qualitative research) Checklist

A checklist of items that should be included in reports of qualitative research. You must report the page number in your manuscript where you consider each of the items listed in this checklist. If you have not included this information, either revise your manuscript accordingly before submitting or note N/A.

Topic	Item No.	Guide Questions/Description	Reported on Page No.
**Domain 1: Research team and reflexivity**			
Personal characteristics			
Interviewer/facilitator	1	Which author/s conducted the interview or focus group?	
Credentials	2	What were the researcher's credentials? E.g. PhD, MD	
Occupation	3	What was their occupation at the time of the study?	
Gender	4	Was the researcher male or female?	
Experience and training	5	What experience or training did the researcher have?	
Relationship with participants			
Relationship established	6	Was a relationship established prior to study commencement?	
Participant knowledge of the interviewer	7	What did the participants know about the researcher? e.g. personal goals, reasons for doing the research	
Interviewer characteristics	8	What characteristics were reported about the inter viewer/facilitator? e.g. Bias, assumptions, reasons and interests in the research topic	
**Domain 2: Study design**			
Theoretical framework			
Methodological orientation and Theory	9	What methodological orientation was stated to underpin the study? e.g. grounded theory, discourse analysis, ethnography, phenomenology, content analysis	
Participant selection			
Sampling	10	How were participants selected? e.g. purposive, convenience, consecutive, snowball	
Method of approach	11	How were participants approached? e.g. face-to-face, telephone, mail, email	
Sample size	12	How many participants were in the study?	
Non-participation	13	How many people refused to participate or dropped out? Reasons?	
Setting			
Setting of data collection	14	Where was the data collected? e.g. home, clinic, workplace	
Presence of non-participants	15	Was anyone else present besides the participants and researchers?	
Description of sample	16	What are the important characteristics of the sample? e.g. demographic data, date	
Data collection			
Interview guide	17	Were questions, prompts, guides provided by the authors? Was it pilot tested?	
Repeat interviews	18	Were repeat inter views carried out? If yes, how many?	
Audio/visual recording	19	Did the research use audio or visual recording to collect the data?	
Field notes	20	Were field notes made during and/or after the inter view or focus group?	
Duration	21	What was the duration of the inter views or focus group?	
Data saturation	22	Was data saturation discussed?	
Transcripts returned	23	Were transcripts returned to participants for comment and/or	

For peer review only

Topic	Item No.	Guide Questions/Description	Reported on Page No.
		correction?	
**Domain 3: analysis and findings**			
Data analysis			
Number of data coders	24	How many data coders coded the data?	
Description of the coding tree	25	Did authors provide a description of the coding tree?	
Derivation of themes	26	Were themes identified in advance or derived from the data?	
Software	27	What software, if applicable, was used to manage the data?	
Participant checking	28	Did participants provide feedback on the findings?	
Reporting			
Quotations presented	29	Were participant quotations presented to illustrate the themes/findings? Was each quotation identified? e.g. participant number	
Data and findings consistent	30	Was there consistency between the data presented and the findings?	
Clarity of major themes	31	Were major themes clearly presented in the findings?	
Clarity of minor themes	32	Is there a description of diverse cases or discussion of minor themes?	

Developed from: Tong A, Sainsbury P, Craig J. Consolidated criteria for reporting qualitative research (COREQ): a 32-item checklist for interviews and focus groups. *International Journal for Quality in Health Care*. 2007. Volume 19, Number 6: pp. 349 – 357

Once you have completed this checklist, please save a copy and upload it as part of your submission. DO NOT include this checklist as part of the main manuscript document. It must be uploaded as a separate file.

For peer review only
